# Coding and decoding libraries of sequence-defined functional copolymers synthesized via photoligation

**DOI:** 10.1038/ncomms13672

**Published:** 2016-11-30

**Authors:** Nicolas Zydziak, Waldemar Konrad, Florian Feist, Sergii Afonin, Steffen Weidner, Christopher Barner-Kowollik

**Affiliations:** 1 Soft Matter Synthesis Laboratory, Institut für Biologische Grenzflächen, Karlsruhe Institute of Technology (KIT), Hermann-von-Helmholtz-Platz 1, 76344 Eggenstein-Leopoldshafen, Germany; 2 Preparative Macromolecular Chemistry, Institut für Technische Chemie und Polymerchemie, Karlsruhe Institute of Technology (KIT), Engesserstrasse 18, 76131 Karlsruhe, Germany; 3 Department of Molecular Biophysics (IGB-2), Institut für Biologische Grenzflächen, Karlsruhe Institute of Technology (KIT), Hermann-von-Helmholtz-Platz 1, 76344 Eggenstein-Leopoldshafen, Germany; 4 BAM-Federal Institute for Materials Research and Testing, Richard-Willstätter-Strasse 11, 12489 Berlin, Germany

## Abstract

Designing artificial macromolecules with absolute sequence order represents a considerable challenge. Here we report an advanced light-induced avenue to monodisperse sequence-defined functional linear macromolecules up to decamers via a unique photochemical approach. The versatility of the synthetic strategy—combining sequential and modular concepts—enables the synthesis of perfect macromolecules varying in chemical constitution and topology. Specific functions are placed at arbitrary positions along the chain via the successive addition of monomer units and blocks, leading to a library of functional homopolymers, alternating copolymers and block copolymers. The in-depth characterization of each sequence-defined chain confirms the precision nature of the macromolecules. Decoding of the functional information contained in the molecular structure is achieved via tandem mass spectrometry without recourse to their synthetic history, showing that the sequence information can be read. We submit that the presented photochemical strategy is a viable and advanced concept for coding individual monomer units along a macromolecular chain.

## Abstract (web summary)

Controlling the molecular sequence of polymers and oligomers is a challenging task. Here the authors use a photoligation approach to synthesise macromolecules with functionality at defined positions throughout the chain, and additionally decode the sequence information via tandem mass spectrometry.

## Graphical abstract

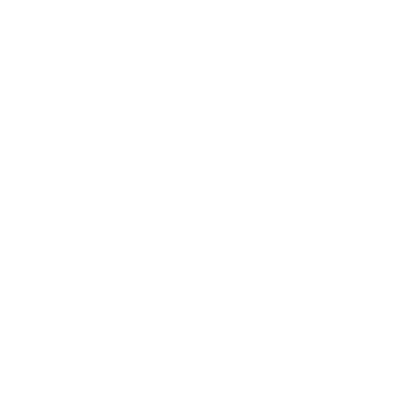

The coding and decoding of information into synthetic molecules is one of the key challenges of contemporary macromolecular chemistry. In the course of evolution, nature has developed finely adjusted tools for information coding, decoding, translation and correction. Despite the enormous progress made in the synthesis of defined macromolecular structures since the formulation of Staudinger's macromolecular hypothesis almost 100 years ago, macromolecular chemists have just started to develop the tools required to mimic nature's precision that is inherent to biomolecules such as DNA, proteins or glyco-sequences. Although these key molecules execute specific functions in biological processes—heredity and information storage for DNA^1^,^2^, enzymatic activity for proteins^3^ or cell communication for sugar motifs^4^—it is evident that perfect sequence order in an ensemble of identical macromolecules is their common defining element. Designing viable approaches to synthetic macromolecules with ‘sequence-controlled' (low dispersion coupled with some statistical fuzziness regarding the placement of the monomer units) or ‘sequence-defined' (no dispersion and 100% accurate placement of the monomers along the chain) order is thus the key to modern macromolecular chemistry. The synthesis of sequence-defined macromolecules—as defined above—represents the far greater challenge than the generation of sequence-controlled species^5^. Generating an ensemble of perfectly sequence-defined identical macromolecules containing exactly the same chemical information at the molecular scale—in contrast to existing avenues to disperse and irregularly coded species when compared with natural analogues—via a simple process constitutes a key technological gate for data storage, biological and material applications, yet requires reaction concepts that provide perfect yields and orthogonality under equimolar reaction conditions. Although solid-state peptide synthesis was introduced by Merrifield^6^—the first example of a true synthetic sequence-defined peptide—macromolecular chemists have initially focused on and developed a diverse number of solution-based polymerization strategies for imparting a certain level of control over statistical polymerization processes and exploited these for controlling the order of the building blocks (sequence-controlled polymers)^7^,^8^,^9^,^10^,^11^,^12^,^13^,^14^,^15^,^16^,^17^,^18^,^19^,^20^. In addition, the advent of modular synthetic strategies^21^,^22^,^23^,^24^ has contributed significantly towards achieving a high level of control over macromolecular formation processes.

Although these advances of limiting the statistical nature of polymerization processes are impressive and led to fine examples of sequence-controlled polymers^7^,^11^,^13^,^15^,^16^, only few sequence-defined examples have been reported that reach the precision level of nature^25^. Directly inspired by biologically accessible DNA systems—for example, nucleobase-coded chains—sequence-defined macromolecules have been synthesized employing template-based coding strategies^26^,^27^,^28^,^29^ or via the generation of regulated sequence alternating thymine hybrid polymers^30^, as well as the design of reactions with iterative protection/deprotection steps on supports^31^,^32^. Further, bulk reactions have been conducted in flow systems based on iterative synthesis^33^. In addition, iterative exponential growth can lead to sequence-defined macromolecules^34^,^35^,^36^. In parallel, strategies involving the consecutive insertion of single monomer units (vinyl or acrylate) based on radical processes started to emerge for the synthesis of sequence-defined polymers^37^,^38^. Further, notable synthetic strategies towards sequence-defined polymers conducted in bulk include the use of thiolactones^39^,^40^,^41^ and multi-component reactions^42^,^43^,^44^. Concomitant to the development of new synthetic approaches to sequence-defined polymers, selected applications of such molecules are emerging, for example, bio-inspired structures for which high sequence definition is essential^45^,^46^. For potential data storage applications, solid support-based systems have emerged featuring monomer units with unreactive variable alkyl groups^47^ and block copolymer phase separation based on sequence-defined oligomers has been demonstrated as well^36^. The herein proposed synthetic strategy based on photoreactive synthons offers significant applications of the functional sequence-defined macromolecules ranging from mild bioconjugation to base materials for advanced precision photoresists for, for example, direct laser writing^48^ or precision network formation for nuclear magnetic resonance (NMR) orientation media^49^,^50^. Critically, photochemistry offers the opportunity to broaden the chemical diversity in the design of sequence-defined macromolecules. Although we herein offer a proof-of-concept study, we submit that the synthetic protocols are amenable to scale up in photoflow systems^51^.

The synthesis of sequence-defined macromolecules in bulk requires highly efficient reaction systems. As a consequence, most reports rarely describe a variation of the sequence order and/or the incorporated functionalities to generate highly diverse libraries of sequence-defined macromolecules and subsequently exploit this diversity to decode the coded chemical information^52^. Herein, we demonstrate how highly efficient photochemical reactions combined with a simple macromolecular design concept can lead to functional sequence-defined linear macromolecules. To achieve this diversity, we built on our initial approach for photochemical sequence control^53^, simplifying the concept while concomitantly showing its power to generate an entire sequence-defined polymer library. The simplicity and versatility of the photoreactions are such that the sequence order and functionality within the chains can be arbitrarily varied. Our approach rests on a sole photochemical reaction relying on benzaldehyde species, which when irradiated lead to reactive dienes (so-called ‘photo-caged dienes')^54^ with an *ortho*-quinodimethane structure. Combined with ene reactive functional molecules, we reach diverse decamers from a library of six monomers. We evidence that the photochemical coding leads to a strictly monodisperse character after each synthetic step, evidenced via size-exclusion chromatography (SEC) and mass-spectrometric tools (matrix-assisted laser desorption/ionization–time-of-flight mass-spectrometry, MALDI–ToF). Owing to the absolute control over the sequence order, the chemical history—which the macromolecules experienced during the photochemical coding process—can be decoded via mass-spectrometric defragmentation experiments (MALDI–ToF–ToF). Such an in-depth characterization of selected macromolecules does not only evidence the successful synthesis of the desired structures but also constitutes a fundamental example of artificial sequence-defined macromolecules' ability to be employed for coding and decoding chemical information.

## Results

### Photochemical concepts for sequence-defined macromolecules

**Figure 1:**
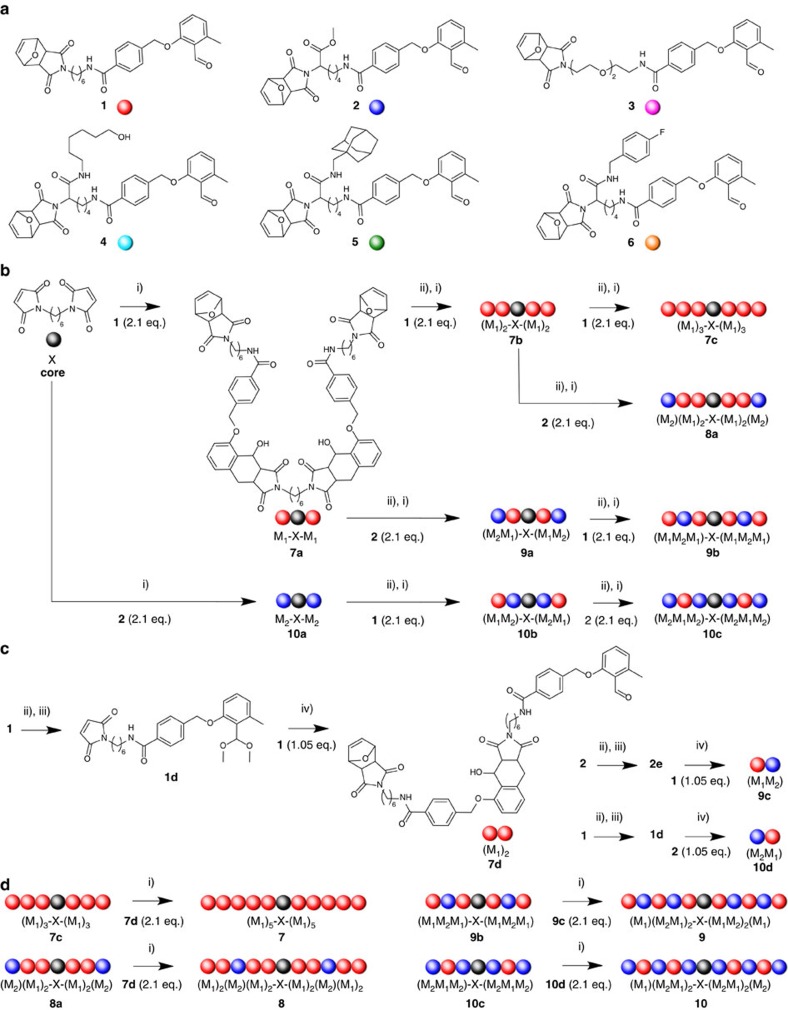
Strategies towards sequence-defined homopolymers and alternating block (co)polymers. (**a**) Synthesized monomers with diverse spacers: **1** (hexyl chain), **2** (lysine-methyl ester function), **3** (poly(ethylene glycol (PEG) chain), **4** (lysine-based monomer with alcohol function), **5** (lysine-based monomer with adamanthyl function), **6** (lysine-based monomer with fluorobenzyl function). (**b**) Sequential synthesis of symmetrical dimers **7a** and **10a**, tetramers **7b** (homopolymer), **9a** and **10b **(alternating polymers), and hexamers **7c** (homopolymer) and **8a**, **9b** and **10c** (copolymers). (**c**) Synthesis of dimer blocks **7d**, **9c** and **10d** with locked/unlocked function for modular ligation. (**d**) Synthesis of decamers as homopolymers **7** and copolymers **8**–**10** varying in composition and topology. (i) dry DCM, *λ*=365 nm, 45 min; (ii) 115 °C, vacuum, overnight, dark; (iii) *p*-TosOH, TMOF, 40 °C, overnight, dry MeOH, dark. Overall yield for monomer **1** (23.1%), **2** (20.8%), **3** (48.2%), **4** (32.4%), **5** (43.7%) and **6** (42.5%).

The photoreaction generating the sequence-defined macromolecules is based on a monomer library (Fig. 1a), in which each monomer carries a photoreactive benzaldehyde function as well as an ene functionality required for the photochemically induced Diels–Alder reaction. Contrary to our previous report—which employed two monomer types with separate diene and dienophile functions^53^—the herein presented monomers entail a dual diene/dienophile character. For example, monomer **1** (denoted M_1_) includes on the one hand the benzaldehyde moiety reacting as diene under ultraviolet irradiation from its *ortho*-quinodimethane state and on the other hand a furan-protected maleimide. The lysine-derived methyl ester monomer **2** (denoted M_2_) and the poly(ethylene glycol) containing monomer **3** (denoted M_3_) follow the same concept as monomer **1**. Monomers **4**–**6** (denoted M_4_, M_5_ and M_6_, respectively) introduce further functionalities for sequence-controlled coding, while resting on the same synthetic concept as monomers **1**, **2** and **3**. The synthetic strategy to introduce the different monomer functionalities is based on a lysine precursor featuring orthogonal protecting groups, that is, fluoromethoxycarbonyl and *tert*-butyloxycarbonyl, in the α- and ɛ-positions. After an initial condensation with the free carboxylic group of the protected amino acid with the corresponding amino derivatives, the different functional monomers are obtained with acceptable yields between ∼23 and 44% (refer to the Supplementary Methods section and the Supplementary Figs 1–32, as well as the caption of Fig. 1).

### Exploiting sequential and modular strategies

Our synthetic strategy to achieve high-molecular-weight molecules with defined sequence order with a limited number of synthetic steps rests on the combination of sequential and modular strategies based on locking and unlocking the diene and dienophile groups of each monomer on demand in an orthogonal manner. The use of a symmetrical starting core (1,6-hexylbismaleimide, denoted **X** in the polymer chain) contributes to a rapid molecular weight growth. For example, considering the synthesis of homopolymer **7** (denoted (M_1_)_5_-X-(M_1_)_5_), the first photoreaction conducted between 1,6-hexylbismaleimide (276.29 g mol^−1^) with two monomer units **1** (516.58 g mol^−1^) leads to the linear symmetrical molecule **7a** of molecular weight 1309.46 g mol^−1^ (Fig. 1b). A subsequent thermal treatment enables further photoligations with the monomer when the maleimide terminus is deprotected. Repeating this cycle to the third sequence order in a sequential manner, the polymer chain is extended monomer unit after monomer unit to generate the tetramer **7b** ((M_1_)_2_-X-(M_1_)_2_) and hexamer **7c** ((M_1_)_3_-X-(M_1_)_3_) of molecular weights 2206.48 and 3103.50 g mol^−1^, respectively. The concomitant modular strategy provides an efficient way for increasing the molecular weight of the polymer by ligating the symmetrical linear molecule with a previously synthesized dimer. One monomer unit **1** (with locked maleimide group) is ligated to a second monomer unit after the selected transformation of the end groups: the ultraviolet-active benzaldehyde moiety is locked as an acetal function, whereas the maleimide function is thermally unlocked (compound **1d**). The complementary unlocked groups of **1** (benzaldehyde) and **1d** (maleimide) react under light irradiation to form the dimer **7d** ((M_1_)_2_) bearing a furan-protected maleimide and a benzaldehyde group, after the mild hydrolysis of the labile acetal function (Fig. 1c). Consequently, the photoreaction between two dimer blocks **7d** (965.10 g mol^−1^) with the symmetrical chain **7c**, obtained after a preliminary thermal treatment, leads to the decamer **7** ((M_1_)_5_-X-(M_1_)_5_) of molecular weight 4897.54 g mol^−1^ after only four photoreactions (Fig. 1d) with an overall isolated yield of 1.0% from the monomer to the final decamer. The low yield results from necessary purification via column chromatography in the early sequence orders, because each photoreaction requires absolute stoichiometry and the bifunctional core undergoes an intermolecular photo-induced side reaction (maleimide–maleimide coupling under ultraviolet irradiation) for the first sequence order, leading to a loss of overall efficiency. However, for the fifth sequence order the difference in solubility of the homo-decamer **7** compared with the hexamer **7c** and dimer blocks **7d** enables purification via precipitation circumventing column purification.

Both sequential and modular strategies can be employed to design more complex copolymers, varying the position of monomer **2** within the homopolymer chain to provide the three additional decamers **8**, **9** and **10** (Fig. 1d). Here, the sequential alternation of **1** and **2** at the first, second and third sequence position diversifies the composition and topology of the symmetrical hexamers (corresponding respectively to **8a**, **9b** and **10c**) with molecular weights in the same order of magnitude as **7c**. The modular chain extension with the dimer blocks **7d**, **9c** and **10d** (the two last dimers resulting from the ligation of **1** and **2**, conducted with a locked benzaldehyde function as for **7d**) results in symmetrical decamers: **8** featuring monomer **2** at the third position, **9** and **10** as completely alternating structures with monomers **1** with **2** (**1** placed at first, third and fifth position for **9**, second and fourth position for **10**).

By inspecting the SEC traces at each synthetic step of the decamers **7**–**10** (Fig. 2), one can observe the expected decrease in retention volume (corresponding to a molecular weight increase) from the monomer units and core molecule to the successive symmetrical dimers, tetramers and hexamers resulting from the sequential approach, as well as the non-symmetrical dimer block following the modular strategy. The observed molecular weights relative to a polystyrene (PS) calibration and the chromatographically observed dispersity of the chains (1.01 for di-, tetra- and hexamers; 1.02–1.03 for decamers), as well as further characterization conducted via NMR spectroscopy and mass spectrometry (refer to Supplementary Figs 40–115) confirms the success of the photochemical synthetic strategy.

### Functional sequence-defined macromolecules

Importantly, our synthetic concept can be extended to monomers bearing functionalities attached at the α-position of the lysine-based monomer, leading to multifunctional sequence-defined macromolecules. The combination of monomers **2** and **4**–**6** was performed via the sequential and modular synthesis as for the decamer **7**. One type of sequence has been realized (Fig. 3a): starting from the symmetrical 1,6-bismaleimide core, the chain is extended sequentially with **2** (methyl ester function) via photoligation, followed by thermal deprotection and photoligation with **4** (alcohol function) to afford the tetramer **11a**, denoted (M_4_M_2_)-X-(M_2_M_4_). To perform the modular ligation, the dimer block **11b** was synthesized from **5** (adamanthyl function) and **6** (fluorobenzyl function). Subsequently, **5** underwent—before the photoreaction with **6**—a thermal treatment unlocking the maleimide group and the locking of the photoenol group via acetalization (**5e**). The evolution of retention time and the monodisperse character of the molecular weight distribution confirm the successful synthesis of the multifunctional octamer **11**, denoted (M_6_M_5_M_4_M_2_)-X-(M_2_M_4_M_5_M_6_) (Fig. 3b). The MALDI–ToF mass spectra (Fig. 3c) of the prepared species additionally evidence the monodisperse character of the synthesized sequence-defined macromolecules and the successful sequential/modular chain extension with an increase of the *m*/*z* values congruent with the addition of the functional monomer and dimer units.

### Non-symmetrical sequence-defined macromolecules

Further, the modular strategy was also exploited to generate non-symmetrical sequence-defined macromolecules (Fig. 4a). Following a similar locking/unlocking strategy to generate the dimer **7d** from monomer **1**, the dimer **12a** (denoted (M_3_)_2_) was synthesized from monomer **3** and the transformed compound **3d** (furan-deprotected and acetalized). The subsequent chain extension of the dimer with a third monomer unit **3**—in the form of **3d**—led to the trimer **12b** (denoted (M_3_)_3_). The photoligation of the transformed dimer from the lock/unlock strategy **12c** and unchanged trimer **12b**—both entities of respectively 1007.09 and 1509.60 g mol^−1^—succeeded in a single synthetic step resulting in the pentamer **12** (denoted (M_3_)_5_) with 2470.62 g mol^−1^ molecular weight. The monodisperse character of the different synthetic step as well as the modular ligation of dimer and trimer blocks was confirmed via SEC (Fig. 4b) and mass spectrometry (Fig. 4c), demonstrating the versatility of photoreactions and the feasibility in constituting with low synthetic efforts *α*, *ω*-functional linear macromolecules.

### Decoding sequence-defined macromolecules

**Figure 2:**
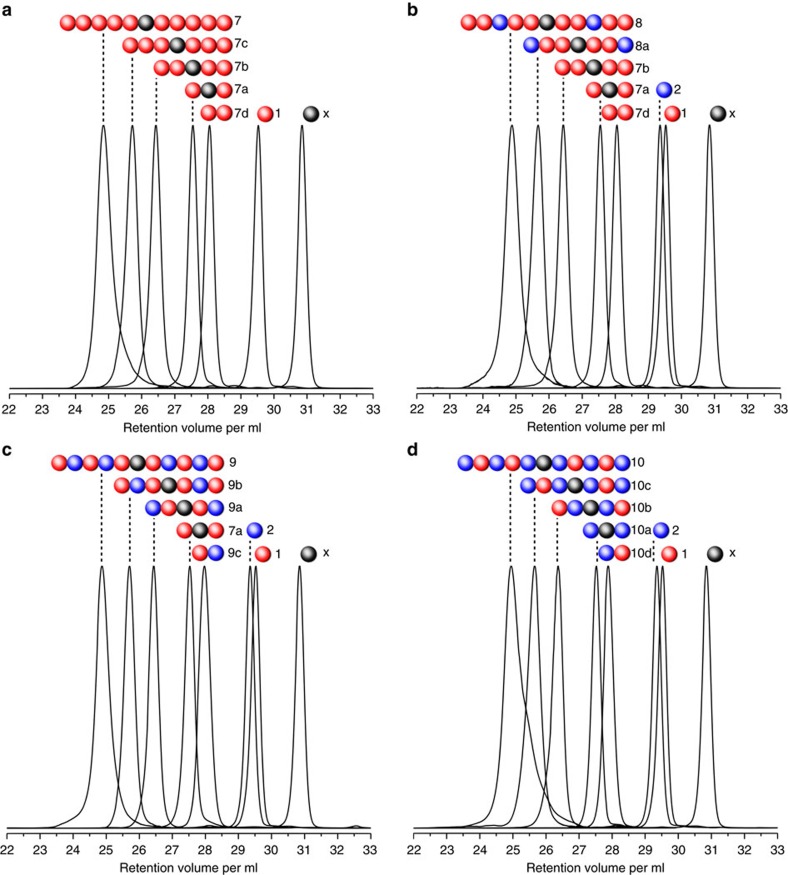
SEC traces of successively synthesized sequence-defined polymers. (**a**) Homopolymer **7**; (**b**) block copolymer **8** (monomer **2** placed at the third position); (**c**) alternating block copolymer **9** (monomer **2** placed at the second and fourth position); (**d**) alternating block copolymer **10** (monomer **2** placed at the first, third and fifth position). All SEC traces are recorded in THF. The core molecule (1,6-hexylbismaleimide **X**) as well as monomers **1** and **2** are also represented. The overall isolated yield is determined from the monomer to the respective decamer: **7** (1.0%), **8** (1.1%), **9** (1.4%) and **10** (4.0%). Shown here and in all figures are weight distributions, which are however identical to the number distributions given the monodisperse nature of the macromolecules.

**Figure 3:**
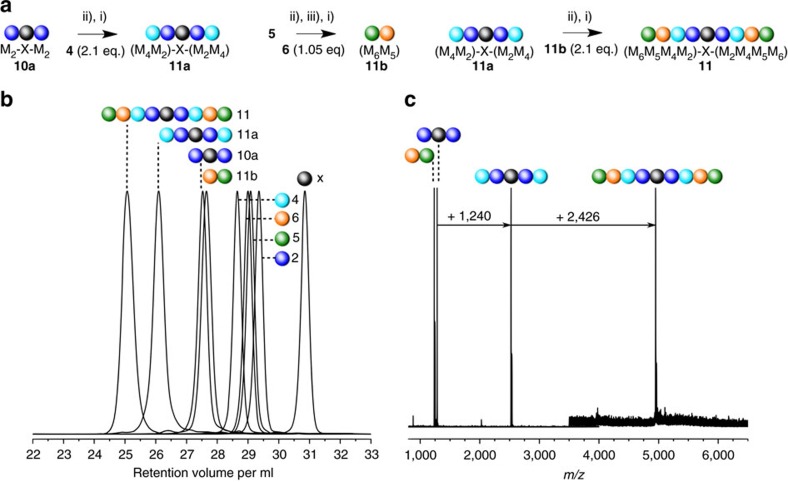
Design of sequence-defined multifunctional polymers. (**a**) Sequential and modular approach for the synthesis of the functional octamer **11**. (**b**) SEC traces (weight distribution) of the synthesized building blocks for the functional sequence-defined polymer. (**c**) MALDI–ToF spectra of the intermediates. The indicated increase of *m*/*z* corresponds to the addition of two fragments of molecule **4** (+1,240 *m/z*) and of the dimers **11b** (+2,426 *m/z*). The MALDI–ToF spectra are depicted in different *m*/*z* ranges: 800–4,000 (dimers **10a** and **11b**, tetramer **11a**), 3,500–6,500 (octamer **11**). (i) Dry DCM, *λ*=365 nm, 45 min, (ii) 115 °C, vacuum, overnight, dark; (iii) *p*-TosOH, TMOF, 40 °C, overnight, dry MeOH, dark.

**Figure 4:**
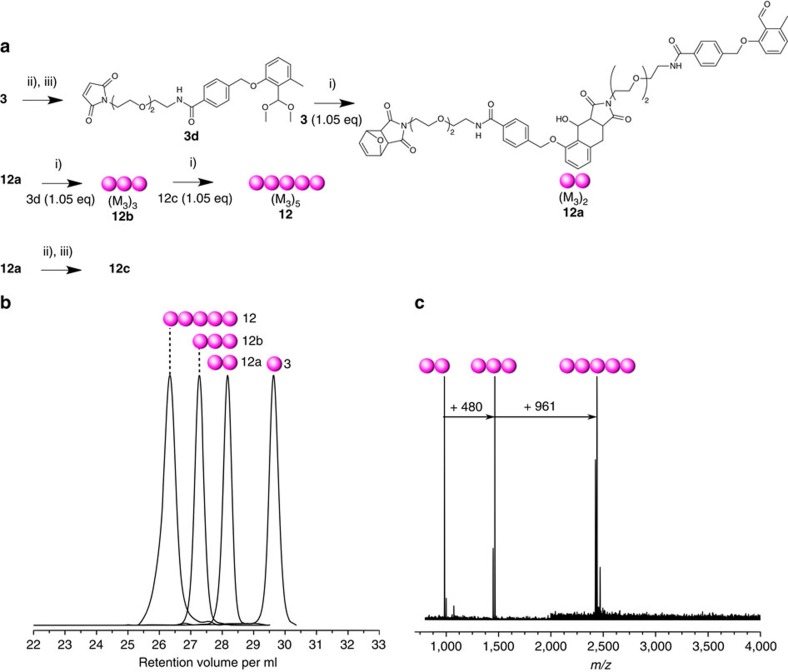
Modular synthesis of pentameric homopolymer. (**a**) Sequential and modular approach for the synthesis of the α,ω-functional pentamer **12**. (**b**) SEC traces (weight distribution) of the synthesized building blocks for the sequence-defined pentamer. (**c**) MALDI–ToF spectra of the intermediates. The indicated increase of *m*/*z* corresponds to the addition of one fragments of molecule **3** (+480 *m/z*) and of the dimer **12a** (+961 *m/z*). The spectrum from **12** results from different counterions (H^+^, Na^+^ and K^+^ adducts) and fragmentation pathways (loss of furan end group). The MALDI–ToF spectra are depicted in different *m*/*z* ranges: 800–4,000 (dimers **12a** and trimer **12b**) and 2,000–4,000 (pentamer **12**). More information can be found in the Supplementary Figs 130–147. (i) Dry DCM, *λ*=365 nm, 45 min; (ii) 115 °C, vacuum, overnight, dark; (iii) *p*-TosOH, TMOF, 40 °C, overnight, dry MeOH, dark. The overall isolated yield is determined from monomer to decamer **12** as close to 2.5%.

**Figure 5:**
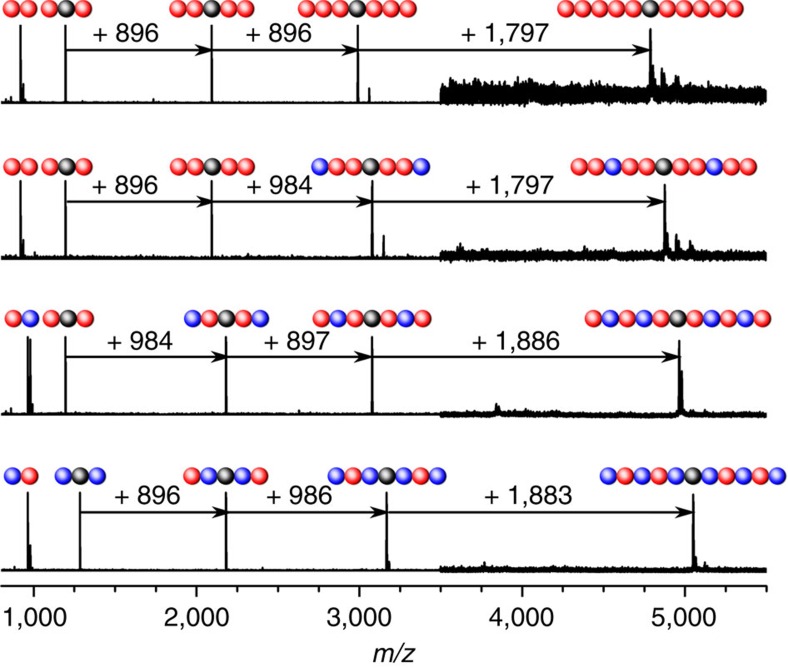
Coding chemical information into sequence-defined macromolecules. The *m*/*z* intervals between the intermediates are characteristic for the addition of two monomer units (896 *m/z* for **1** and 986 *m/z* for **2**) or two dimer blocks (1,797 *m/z* for **7d** and 1,883 *m/z* for **9c** or **10d**) at the chain termini. The MALDI–ToF spectra are depicted in different *m/z* ranges: 800–4,000 for the dimers (**7a**, **7d**, **9c** and **10d**), tetramers (**7b**, **9a** and **10b**), hexamers (**7c**, **8a**, **9b** and **10c**) and 3,500–5,500 for the decamers (**7**, **8**, **9** and **10**).

The verification of the sequence order represents no specific challenge as long as the chemical history of the sequence-defined macromolecules is known. However, with no access to the synthetic history of the sequence-defined macromolecules, decoding the chain structure becomes highly challenging, similar to what is required for naturally occurring biomolecules (see below). The use of mass spectrometric tools is essential to follow the evolution of the molecular weight increase along the defined polymer chain extension and to elucidate the composition of a given molecule. The overlaid MALDI–ToF spectra of the successive chain extension of the first-order dimers (**7a** and **10a**) to the copolymers **7**–**10** confirm the expected molecular weight increase—extending the chain monomer unit after monomer unit—as well as the monodispersity of each compound (Fig. 5). In this context it is interesting to note that the SEC trace of **10** (Fig. 2d) shows a tailing of the molecular weight distribution, leading to a slight increase of dispersity from the third to the fifth sequence (1.01 to 1.03). However, inspection of Fig. 5 indicates for the same compound a single molecular peak, attesting the species monodispersity. The tailing in the SEC trace may be associated with the particularly rich lysine composition of **10**, leading to an adsorption-driven column interaction.

**Figure 6:**
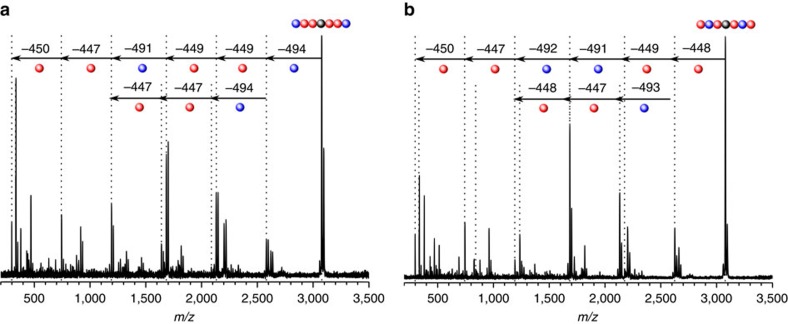
Decoding alternated sequence ordered macromolecules. (**a**) MALDI–ToF–ToF spectrum obtained for **8a**. (**b**) MALDI–ToF–ToF spectrum obtained for **9b** with similar fragments for the monomer units. The fragmentation pattern enables to decipher the monomer sequence of **8a** and **9b** with similar exact mass. Characteristic fragments from monomer **1** and **2** are assigned to 448 and 492 Da, respectively.

Nevertheless, decoding the chemical information stored in the sequence-defined molecules without relying on past-synthetic evidence is—in the current case—possible by MALDI–ToF–ToF experiments. Despite the limitation of this technique to relatively low mass (*ca*. 3,000 *m/z*), the identification of fragments from the hexamers **7c**, **8a**, **9b** and **10c** gives ready access to their composition and topology. Indeed, the fragmentation behaviour of the two hexamers **8a** and **9b** with identical mass is all the more interesting, as they differ in topology (monomer **2** is placed at the third position in the polymer chain in **8a**, whereas it can be found in the second position in **9b**). Despite the complex MALDI–ToF–ToF spectra, the sequence order can be decoded (Fig. 6) and a fragmentation mechanism for the hexamers (**7c**, **8a**, **9b** and **10c**) is proposed (refer to Supplementary Figs 148–159 for details). Fragments of the monomer units **1** and **2** can be identified as well as a specific fragmentation pattern. Indeed, owing to the symmetry of the hexamers, monomer units located at the chain termini fragment preferentially. The known symmetry of the molecules thus eases the interpretation of the spectra and leads to the identification of three consecutive monomer units present in one constituting arm. This behaviour enables to differentiate both hexamers **8a** and **9b**. For **8a**, the first fragment located at the third position (chain terminus) can be assigned to monomer **2**. For smaller *m*/*z* values, the molecule releases only fragments typical of **1**, corresponding to the presence of **1** at the second and first position. In the case of **9b**, the first fragment identified is derived from **1**, hence localized at the third position in the sequence, whereas monomer **2** is assigned at the second position due the symmetric structure of the molecule. Fragments appearing at smaller *m*/*z* values correspond to a monomer present at the first position in the sequence and are clearly identified as monomer **1**.

## Discussion

We introduce a versatile photochemical platform for generating a library of functional sequence-defined macromolecules with varying length, composition and topology. Based on a combination of sequential and modular light-induced approaches, precision homo-, co- and block copolymers are reported, entailing the synthesis of several functional building blocks ready to be combined on demand. Owing to the absolute control of the monomer order within the polymer chain—monomer unit after monomer unit—the macromolecules are as monodisperse and feature absolute chain-end fidelity. Although the synthetic strategy offers a unique photochemical access route to sequence-defined macromolecules, the in-depth characterization of the sequence order, for example, the composition and the topology, requires multiple analytical tools when considering molecules with unknown chemical history. The present study provides a methodology to code and, to a certain extent, decode chemical information contained in photochemically prepared sequence-defined macromolecules, in some cases without recourse to the synthetic history. From a more general point of view, photochemistry provides a wide variety of efficient and orthogonal reactions ideal for sequencing light-triggered monomers into polymer chains, thus coding information. We hope that the current photochemical technology platform will pave the way for activities into mimicking selected features of nature's macromolecules such as writing and transcripting information or possibly even self-replication based on photochemical processes.

## Methods

### General

Supplementary Methods and characterization of the intermediate and final compounds are described in detail in the Supplementary Information section. For all molecular analytical data (NMR, mass spectrometry (MS) and ultaviolet–visible analysis) of the compounds refer to Supplementary Figs 1–147 and Supplementary Tables 1–28. The principle of the photoreaction and the schematic drawings of the photoreactor are depicted in Supplementary Figs 37–39. The tandem MALDI-ToF-ToF data are represented in the Supplementary Figs 148–159 and the Supplementary Tables 29–32.

### Size-exclusion chromatography

SEC measurements were performed on a TOSOH Eco-SEC HLC-8320 GPC System, comprising an autosampler, a SDV 5 μm bead size guard column (50 × 8 mm, PSS) followed by three SDV 5 μm columns (300 × 7.5 mm, subsequently 100, 1,000 and 10^5^ Å pore size, PSS) and a differential refractive index detector using tetrahydrofuran (THF) as the eluent at 30 °C with a flow rate of 1 ml min^−1^. The SEC system was calibrated using linear PS standards ranging from 266 to 2.52 10^6^ g mol^−1^. Calculation of the molecular weight proceeded via the Mark–Houwink–Sakurada parameters for PS in THF at 30 °C, that is, *K*=13.63 10^−3^ ml g^−1^, *α*=0.714.

### NMR spectroscopy

^1^H NMR spectroscopy, H-H proton correlation spectroscopy and C-H correlation spectroscopy (heteronuclear multiple-quantum correlation) were performed on a Bruker AM 500 spectrometer (500 MHz for ^1^H/125 MHz for ^13^C/470 MHz for ^19^F). The analytes were dissolved in CDCl_3_ and the residual solvent peaks were employed for shift correction. The following abbreviations were used to describe peak patterns when appropriate: s (singlet), d (doublet), dd (doublet of doublets), ddd (doublet of doublets of doublets), t (triplet), q (quadruplet), dt (doublet of triplet), td (triplet of doublet), tt (triplet of triplet), qd (quadruplet of doublet), dtq (doublet of triplet of quadruplet), tdd (triplet of doublet of doublet) and m (multiplet). For compounds **2**, **4–6** and **4c–6c**, a signal splitting of analogous carbon atoms (noted with a star * in ^13^C NMR spectroscopy) occurred due to a high rotation barrier of the maleimide-N-C5. Consequently, two mirror symmetric conformational isomers are present and the atoms do not have the same electronic environment. This phenomenon is observed for all the synthesized monomers in the ^13^C NMR spectra. The effect is too weak to be detected in ^1^H NMR spectroscopy.

### Electrospray ionization MS analysis

A Q Exactive (Orbitrap) mass spectrometer (Thermo Fisher Scientific, San Jose, CA, USA) equipped with a HESI II probe was employed to record high resolution electrospray ionization–MS. Calibration was carried out in the *m/z* range 74–1,822 using premixed calibration solutions (Thermo Fisher Scientific). A constant spray voltage of 4.7 kV and a dimensionless sheath gas of 5 were employed. The S-lens RF level was set to 62.0, while the capillary temperature was set to 250 °C. All samples were dissolved at a concentration of 0.05 mg ml^−1^ in a mixture of THF and MeOH (3:2) doped with 100 μmol sodium trifluoroacetate and injected with a flow of 5 μl min^−1^.

### Ultraviolet–visible spectroscopy

Ultraviolet–visible spectroscopy was conducted on a Varian Cary 300 Bio spectrophotometer. The samples were dissolved with a concentration of 0.04 mg ml^−1^ in dry dichloromethane (DCM).

### Spectrophotometry to determine the source emission spectrum

The source emission spectrum was measured in the 200–800 nm range with an ultraviolet–visible spectrometer SR600 (Model 840 320, Opystec Dr Gröbel) calibrated to the National Metrology Institute of Germany (Physikalisch-Technische Bundesanstalt) and equipped with a linear silicon photodiode array. Measurements were performed with a resolution of 0.6 nm. The emission spectrum was registered after a dark measurement and results from the averaging of 20 spectra employing an integration time of 400 ms. The probe was placed at the identical distance employed for irradiating the reaction samples.

### MALDI–ToF–MS analysis

The mass spectra were obtained using MALDI–ToF. An Autoflex III instrument (Bruker Daltonics, Bremen, Germany) equipped with a smartbeam Nd:YAG laser (355 nm, 200 Hz) was employed. A 1:1 mixture of 2,5-dihydroxybenzoic acid and 2-cyano-3-(4-hydroxyphenyl)acrylic acid, freshly dissolved in THF, was used as the matrix solution (40 mg ml^−1^). Analyte samples were obtained as THF solutions without the concentration control. Analyte/matrix mixtures (1/3, v/v) were obtained immediately before the on-target deposition. The mixtures were applied on a stainless steel target, pre-washed with pure THF, in three successive deposition air-drying steps (0.3 μl each time). After the deposition, the entire target was additionally dried under the elevated air flow in the flow bench (15 min). All operations were performed at ambient temperature and extensive exposure to light was avoided at all stages. Depending on the required *m/z* range, both linear and reflection positive-ion measurement modes were used. Calibration of the measurement methods before measurements was carried out following the manufacturer's procedures. The spectra were acquired and processed with the ‘Compass 1.3 for flex' software package from Bruker.

### Tandem MALDI–ToF–MS analysis

An Autoflex III MALDI–ToF mass spectrometer (Bruker Daltonics, Germany) was employed for the tandem MS/MS measurements. The system is equipped with a laser operating at 355 nm with a frequency of 200 Hz. Fragmentation was performed using the so-called LIFT mode. Precursor ions were selected and, after collision-induced dissociation using argon as collision gas, formed fragment ions were accelerated in a second ToF unit. For sample preparation solutions of polymer (2 mg ml^−1^) and DCTB (*trans*-2-[3-(4-*tert*-butylphenyl)-2-methyl-2-propenylidene]malononitrile) matrix (20 mg ml^−1^) were mixed (1/10, v/v). 1 μl of the resulting solution was deposited on the stainless steel target plate and, after air drying, inserted into the mass spectrometer.

### Flash chromatography

Flash chromatography was performed on an Isolera Biotage One (OS 578). The fractions were collected via a ultraviolet detector (254 nm). A SNAP Ultra (10 g) cartridge was employed for the purification in direct mode and a SNAP C18 (12 g) cartridge for the reverse mode (both column volume of 15 ml). The analyte was dried on an adapted short pre-column before purification.

### Preparative HPLC

Preparative HPLC was conducted with a Jasco LC-2000 Plus Series system equipped with two PU 2,087 Plus pumps (flow up to 50 ml min^−1^) and a diode array detector (MD-2010 Plus 195–650 nm). The purification of the compounds was performed in reverse phase mode with a 218TP Vydac C18 column (22 × 250 mm, Grace Davison Discovery Science).

### Data availability

All data are available from the authors upon reasonable request addressed to C.B.-K.

## Additional information

**How to cite this article:** Zydziak, N. *et al*. Coding and decoding libraries of sequence-defined functional copolymers synthesized via photoligation. *Nat. Commun.* **7,** 13672 doi: 10.1038/ncomms13672 (2016).

**Publisher's note**: Springer Nature remains neutral with regard to jurisdictional claims in published maps and institutional affiliations.

## Supplementary Material

Supplementary Information Supplementary Figures 1-159, Supplementary Tables 1-32, Supplemenatry Methods and Supplemenatry References
