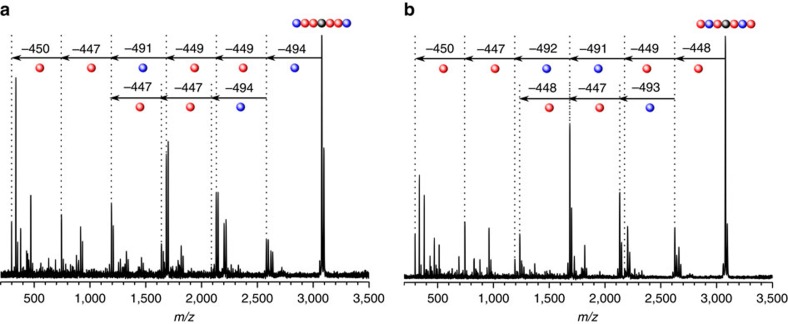# Erratum: Coding and decoding libraries of sequence-defined functional copolymers synthesized via photoligation

**DOI:** 10.1038/ncomms14166

**Published:** 2016-12-23

**Authors:** Nicolas Zydziak, Waldemar Konrad, Florian Feist, Sergii Afonin, Steffen Weidner, Christopher Barner-Kowollik

Nature Communications 7: Article number: 13672; DOI: 10.1038/ncomms13672 (2016); Published 11 30 2016; Updated 12 23 2016

Figures 1 and 6 of this Article contain errors that were introduced during the production process. In Fig. 1, the reaction conditions required to convert **1** to **7d**, **2e** to **9c** and **1d** to **10d** should be described by (i) and not (iv). In Fig. 6, the arrow representing the monomer fragment of 448 Da is pointing to the wrong dashed spectral line. The corrected versions of Figs 1 and 6 are shown below as Figs 1 and 2, respectively.

## Figures and Tables

**Figure 1:**
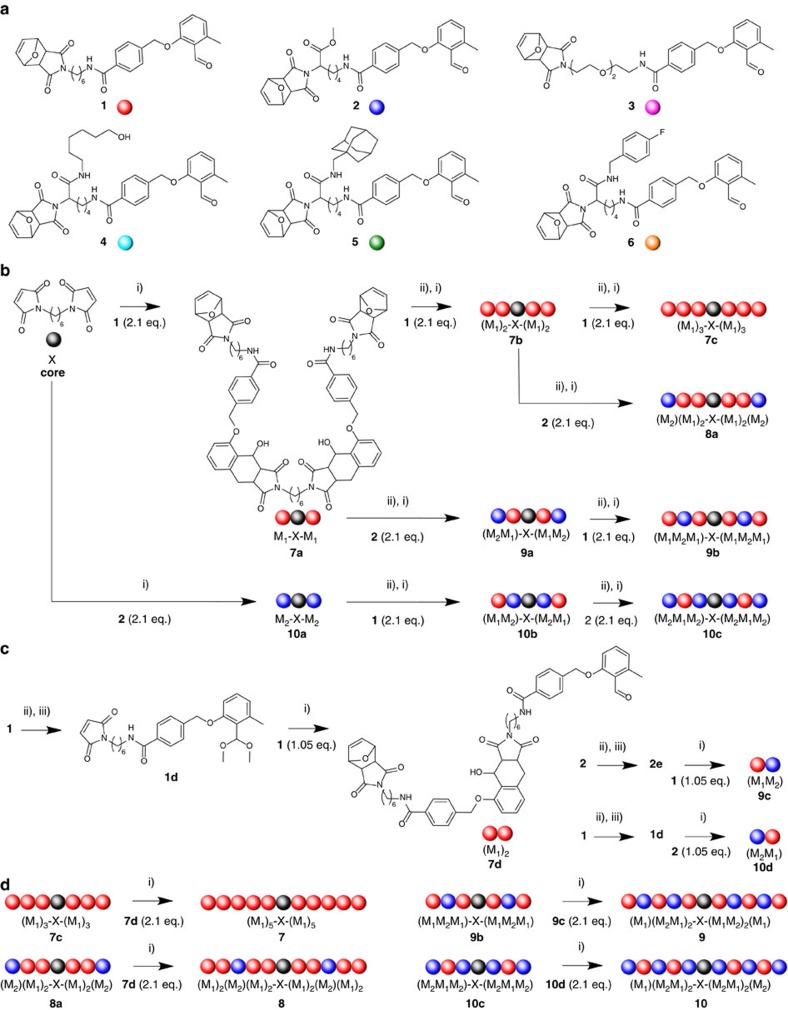


**Figure 2:**