# Supplementary material for: Coding and decoding libraries of sequence-defined functional copolymers synthesized via photoligation
Source: Nat Commun. 2016 Nov 30;7:13672. doi: 10.1038/ncomms13672 (PMC5141382; doi:10.1038/ncomms13672)
Supplement: Supplementary Information — Supplementary Figures 1-159, Supplementary Tables 1-32, Supplemenatry Methods and Supplemenatry References [file ncomms13672-s1.pdf]

Characterization of Synthon 1 (Monomer M_1)

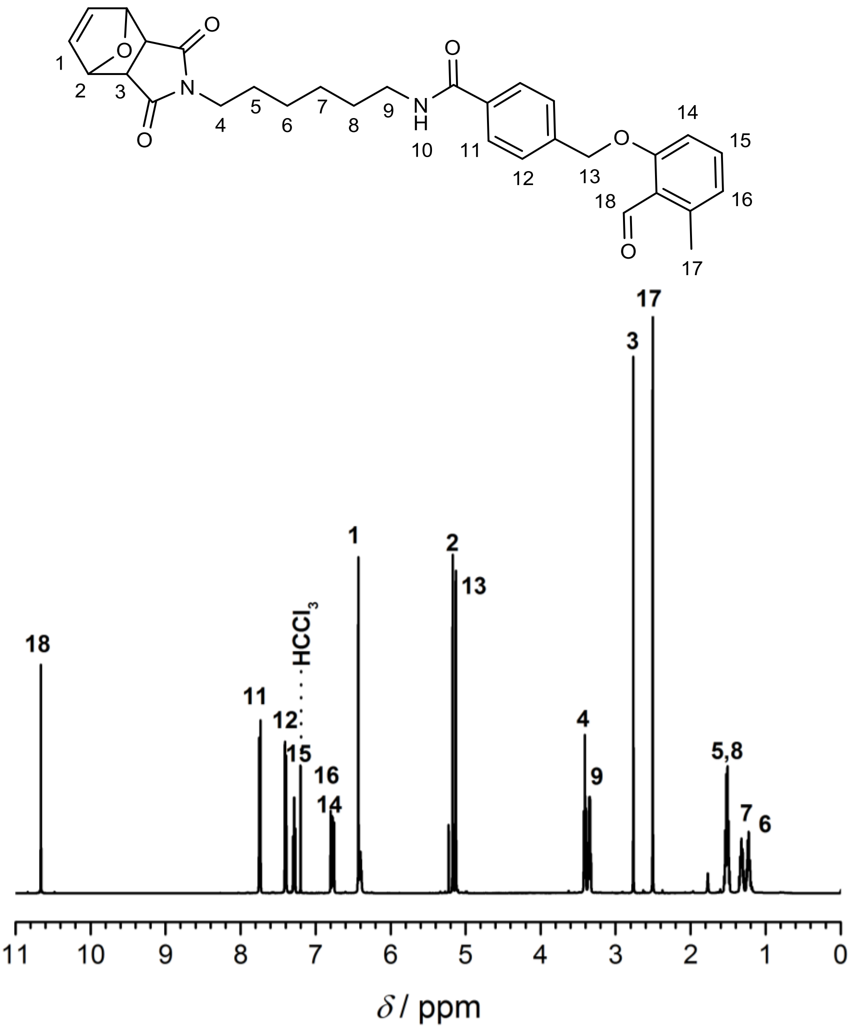

Supplementary Figure 1. ^{1}H NMR spectrum of **1** ($CDCl_3$).

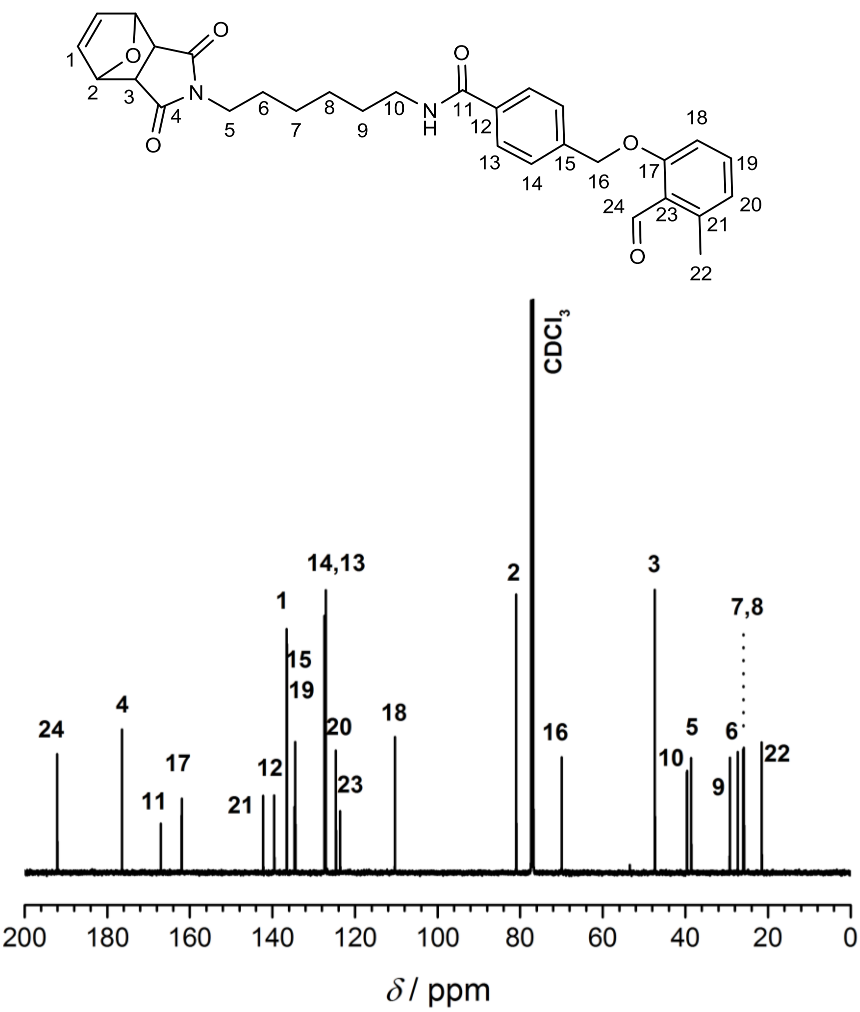

Supplementary Figure 2. ^{13}C NMR spectrum of **1** ($CDCl_3$).

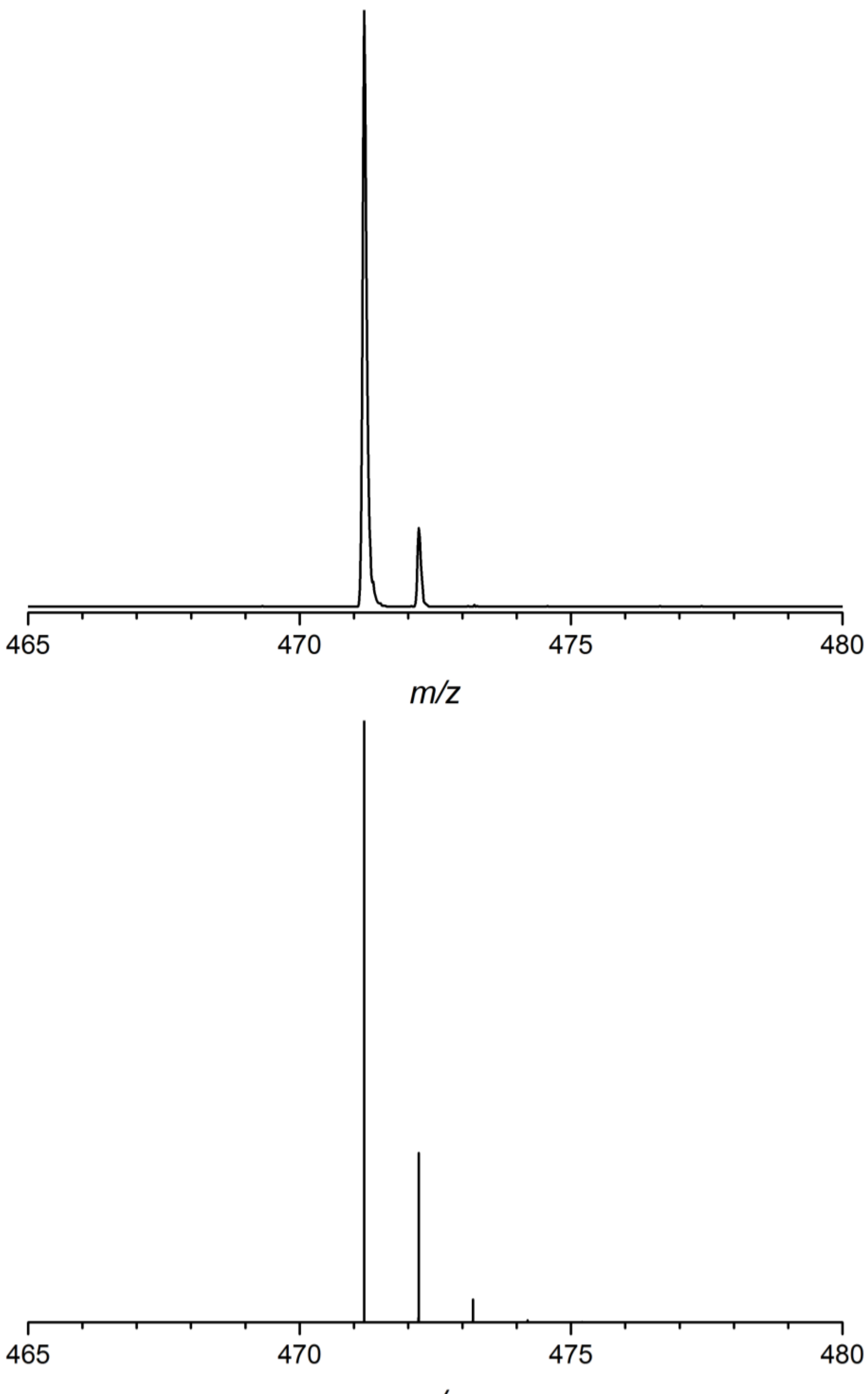

Supplementary Figure 3. MALDI–ToF experimental (top) and calculated (bottom) zoom spectra of **1**.

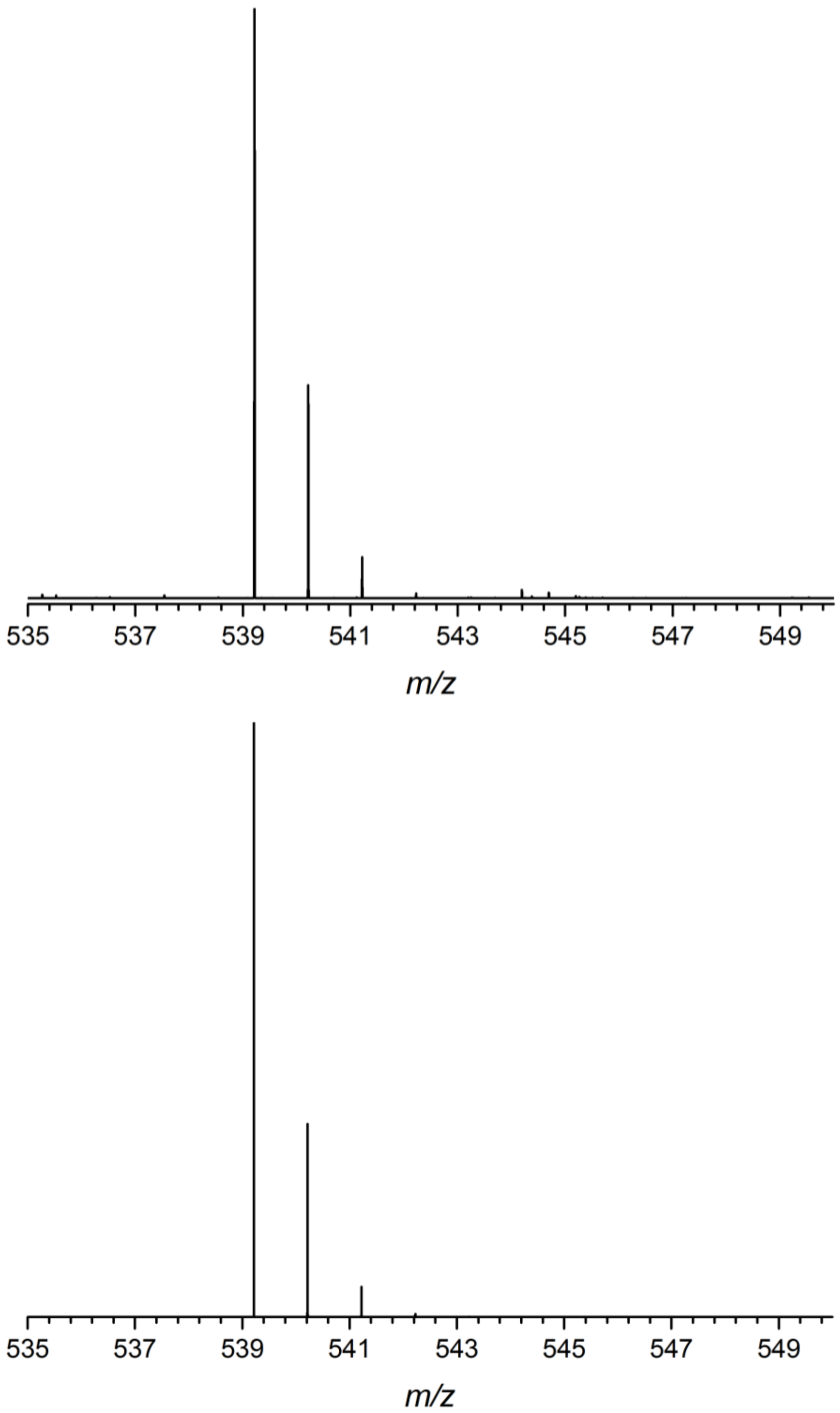

Supplementary Figure 4. ESI-MS experimental (top) and calculated (bottom) zoom spectra of **1**.

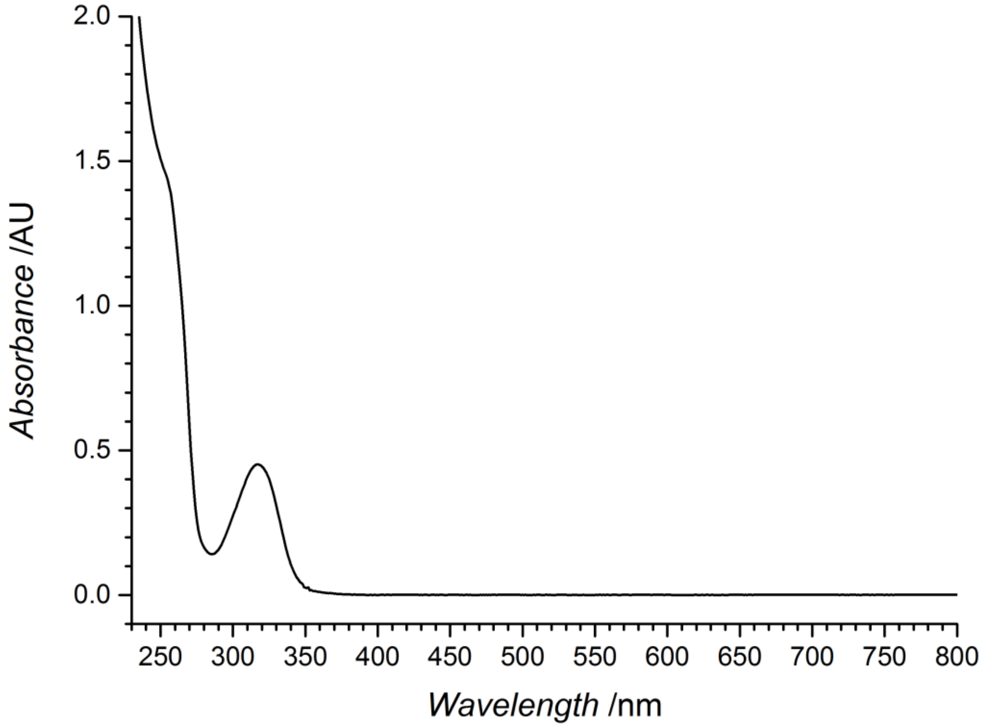

Supplementary Figure 5. UV-VIS spectrum of **1** (0.04 mg mL^{-1} in dry DCM).

Characterization of Synthon 2 (Monomer M_2)

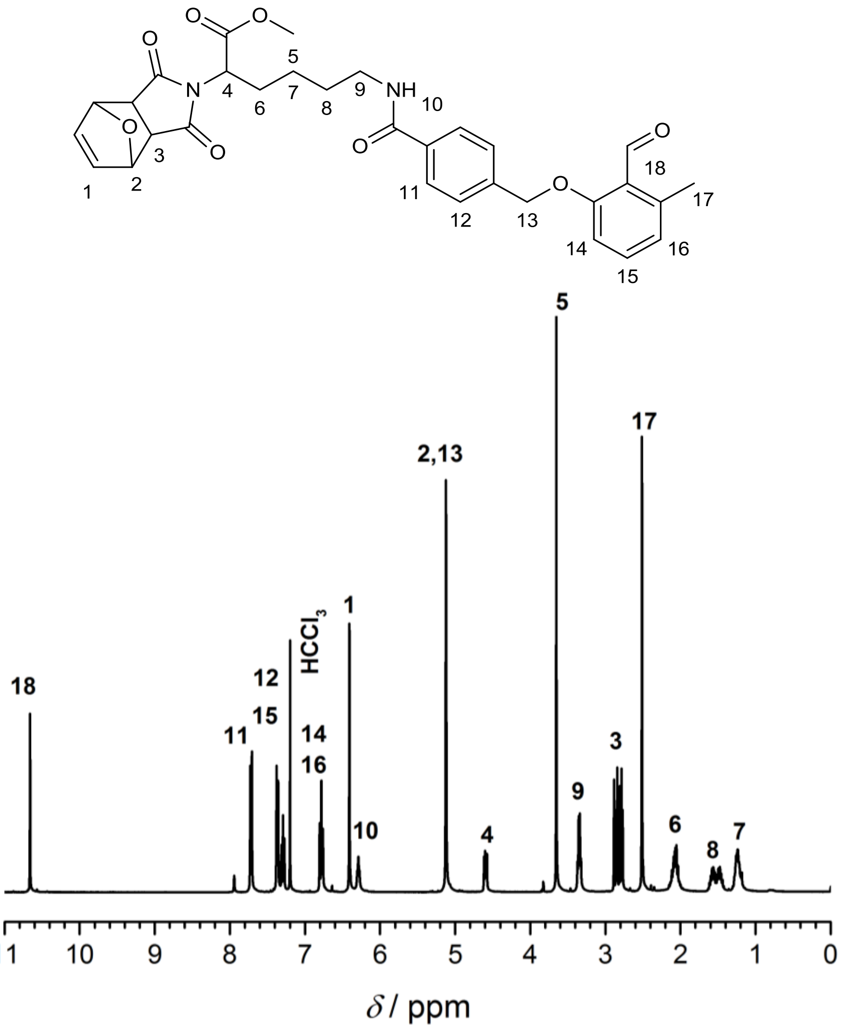

Supplementary Figure 6. ^{1}H NMR spectrum of **2** ($CDCl_3$).

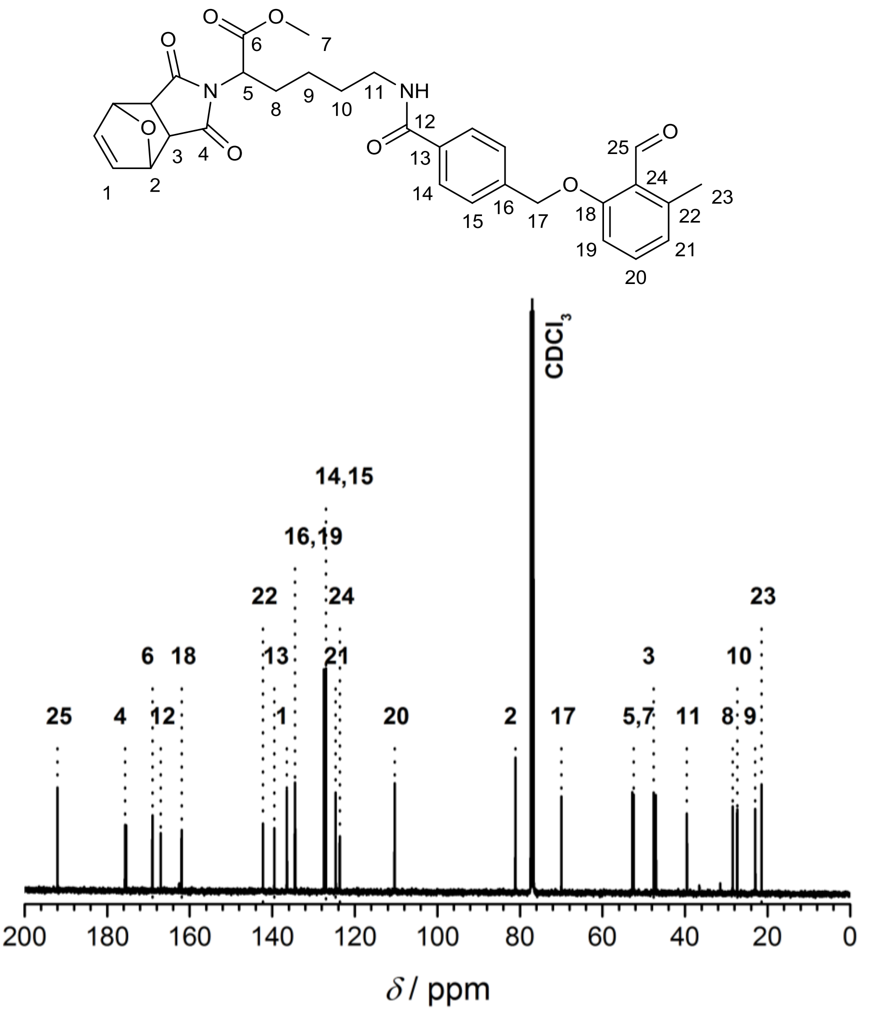

Supplementary Figure 7. ^{13}C NMR spectrum of **2** ($CDCl_3$).

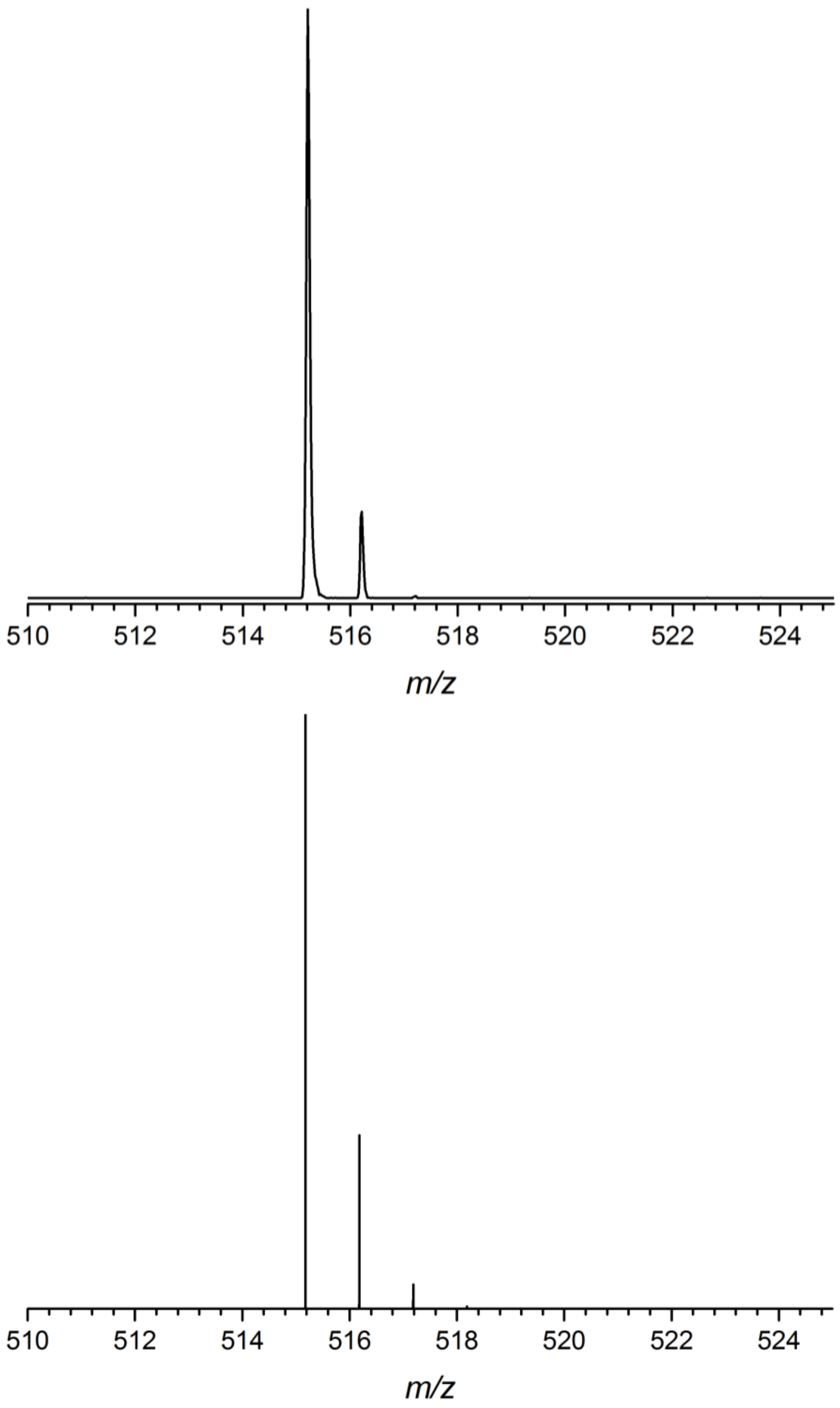

Supplementary Figure 8. MALDI–ToF experimental (top) and calculated (bottom) zoom spectra of **2**.

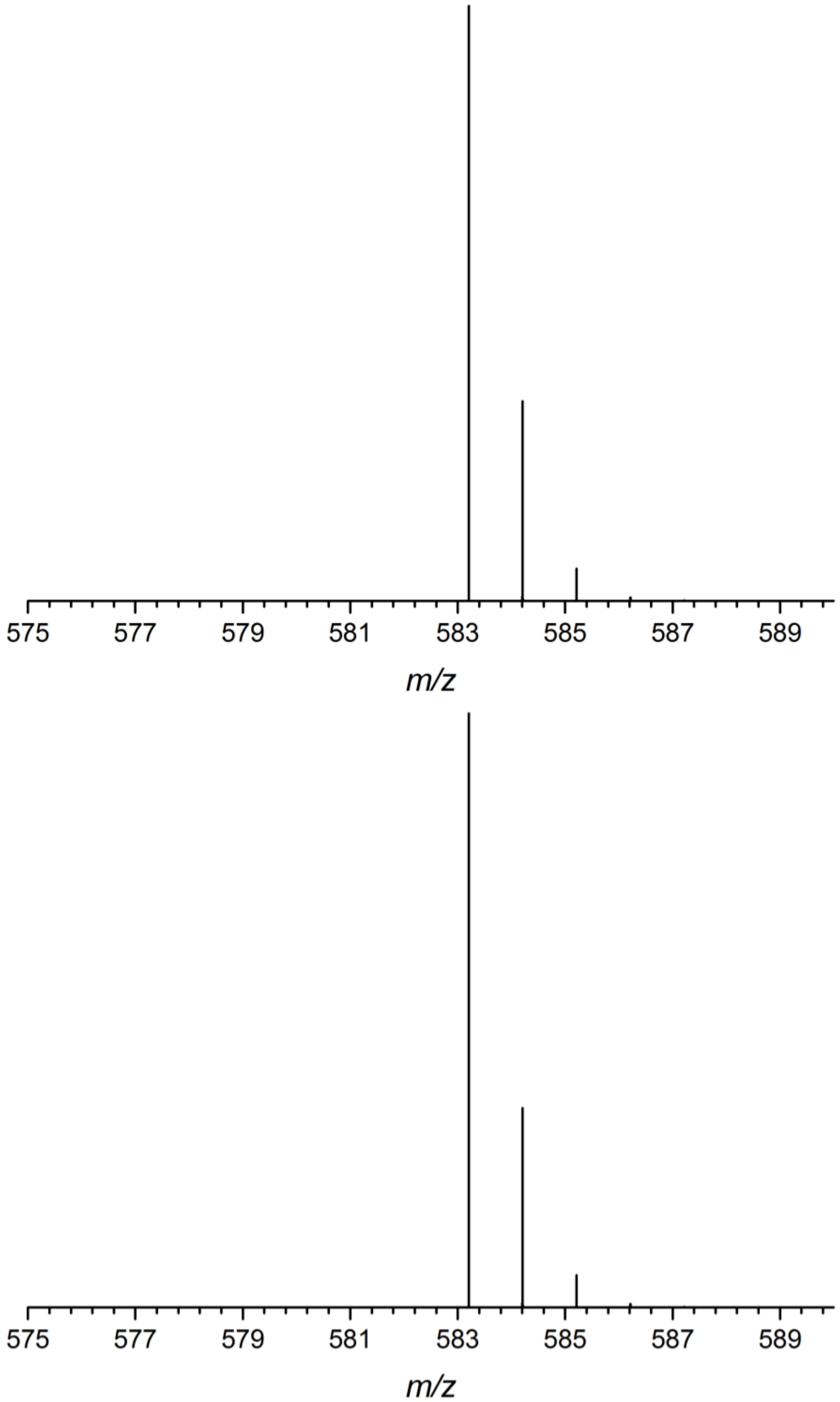

Supplementary Figure 9. ESI-MS experimental (top) and calculated (bottom) zoom spectra of **2**.

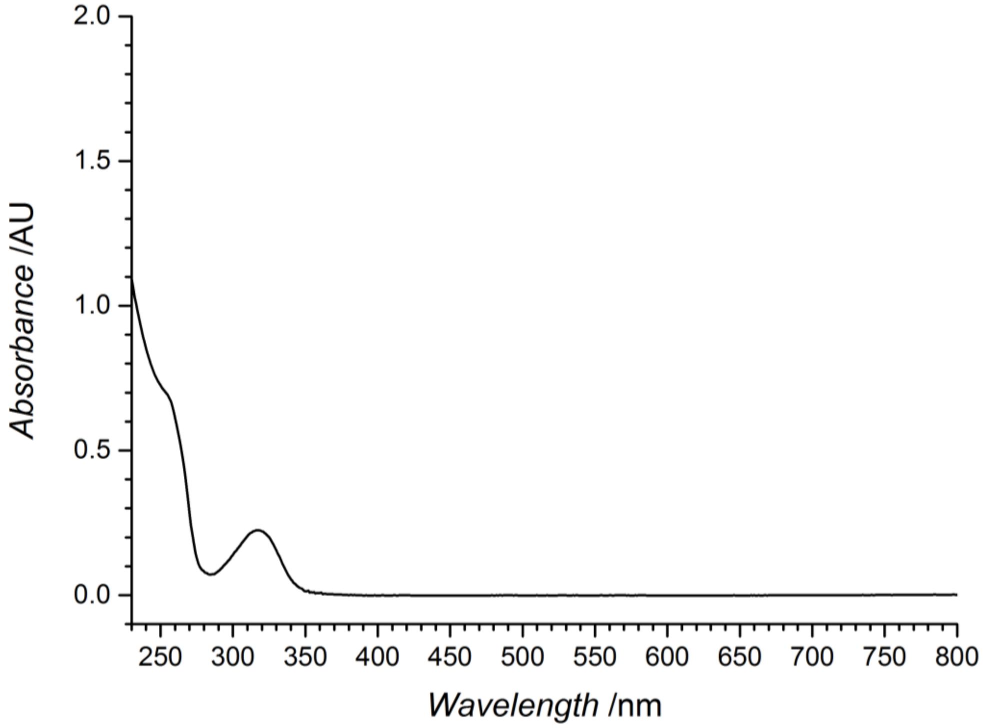

Supplementary Figure 10. UV-VIS spectrum of **2** (0.04 mg mL^{-1} in dry DCM).

Characterization of Synthon 3 (Monomer M_3)

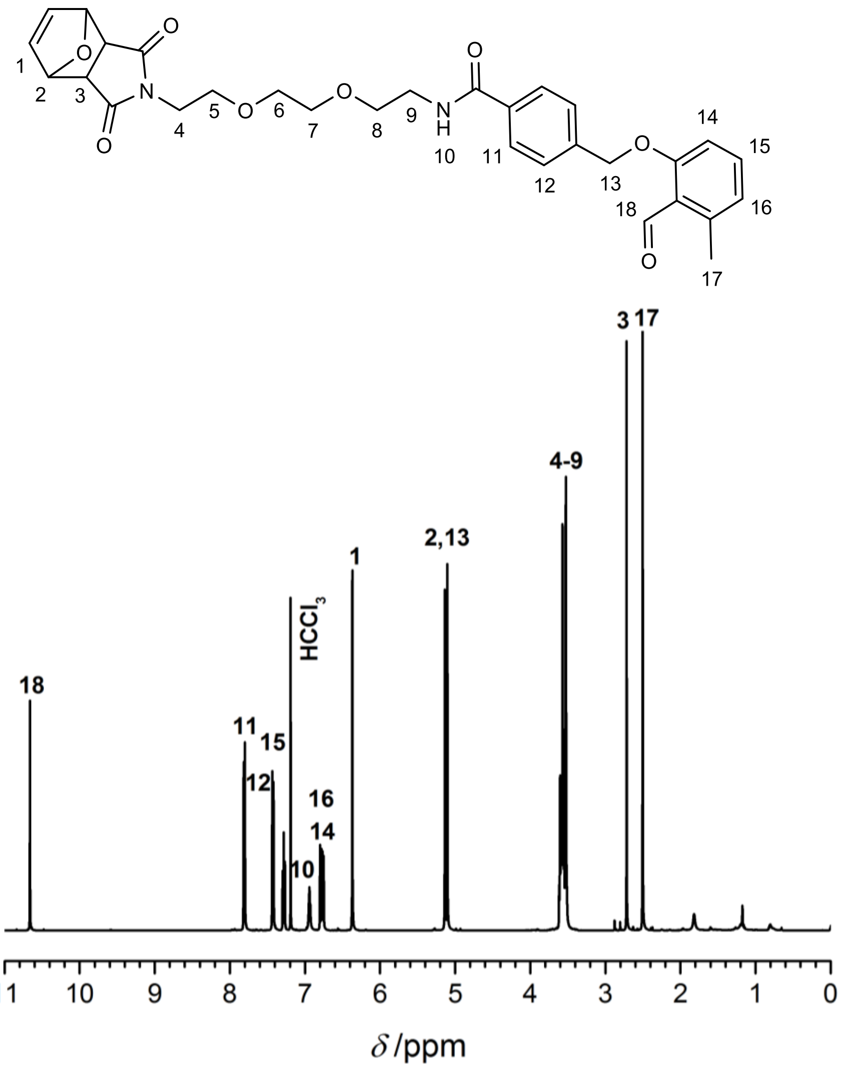

Supplementary Figure 11. ^{1}H NMR spectrum of **3** ($CDCl_3$).

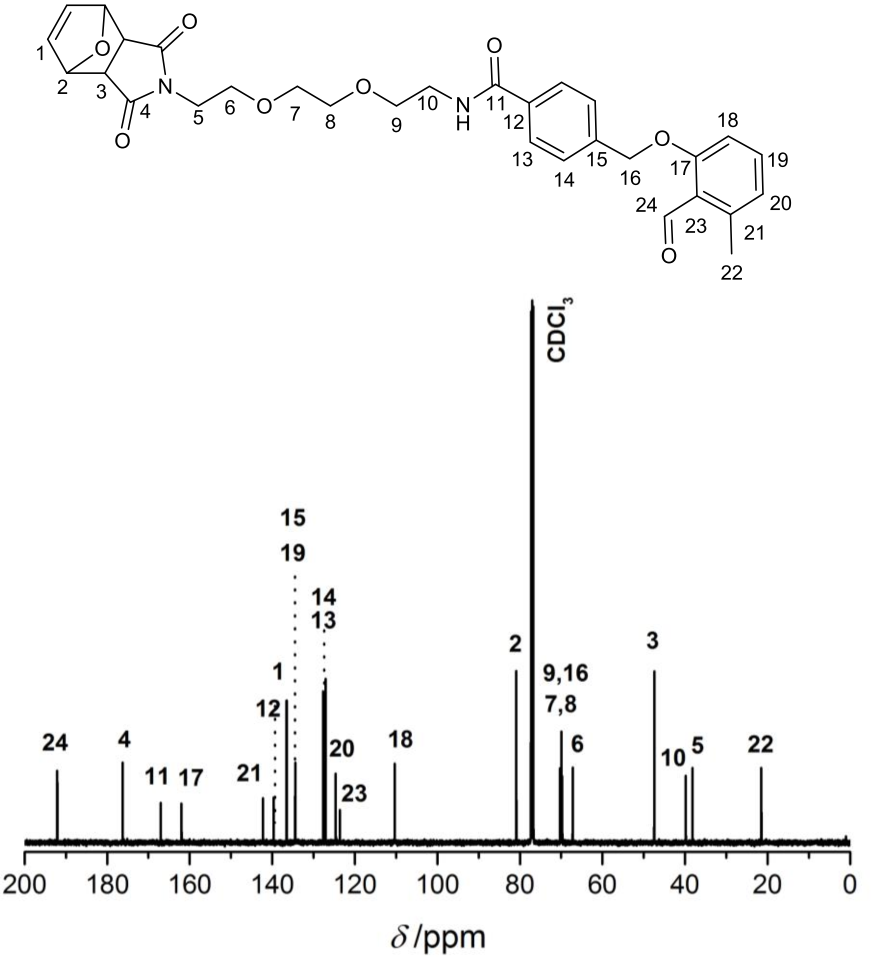

Supplementary Figure 12. ^{13}C NMR spectrum of **3** ($CDCl_3$).

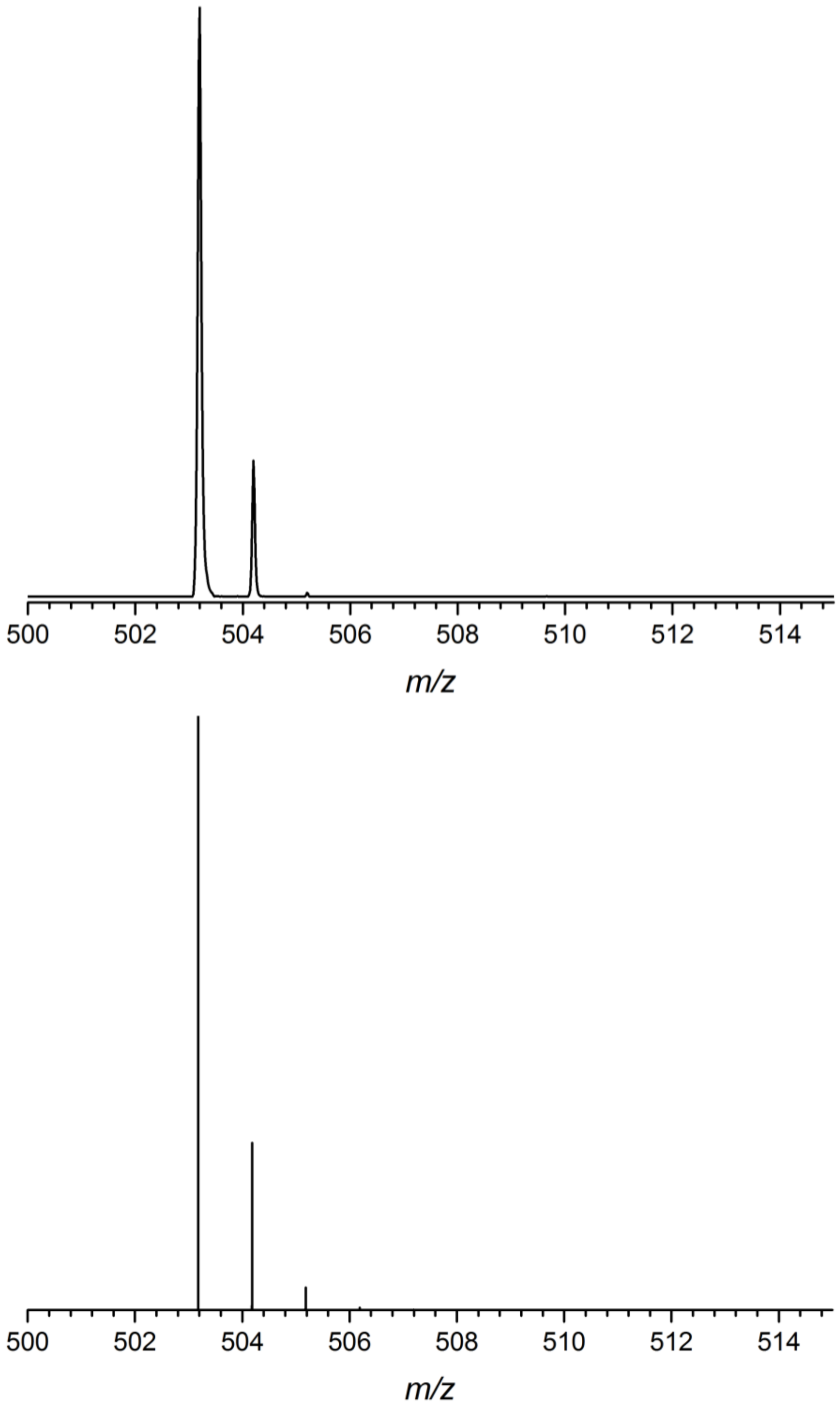

Supplementary Figure 13. MALDI–ToF experimental (top) and calculated (bottom) zoom spectra of **3**.

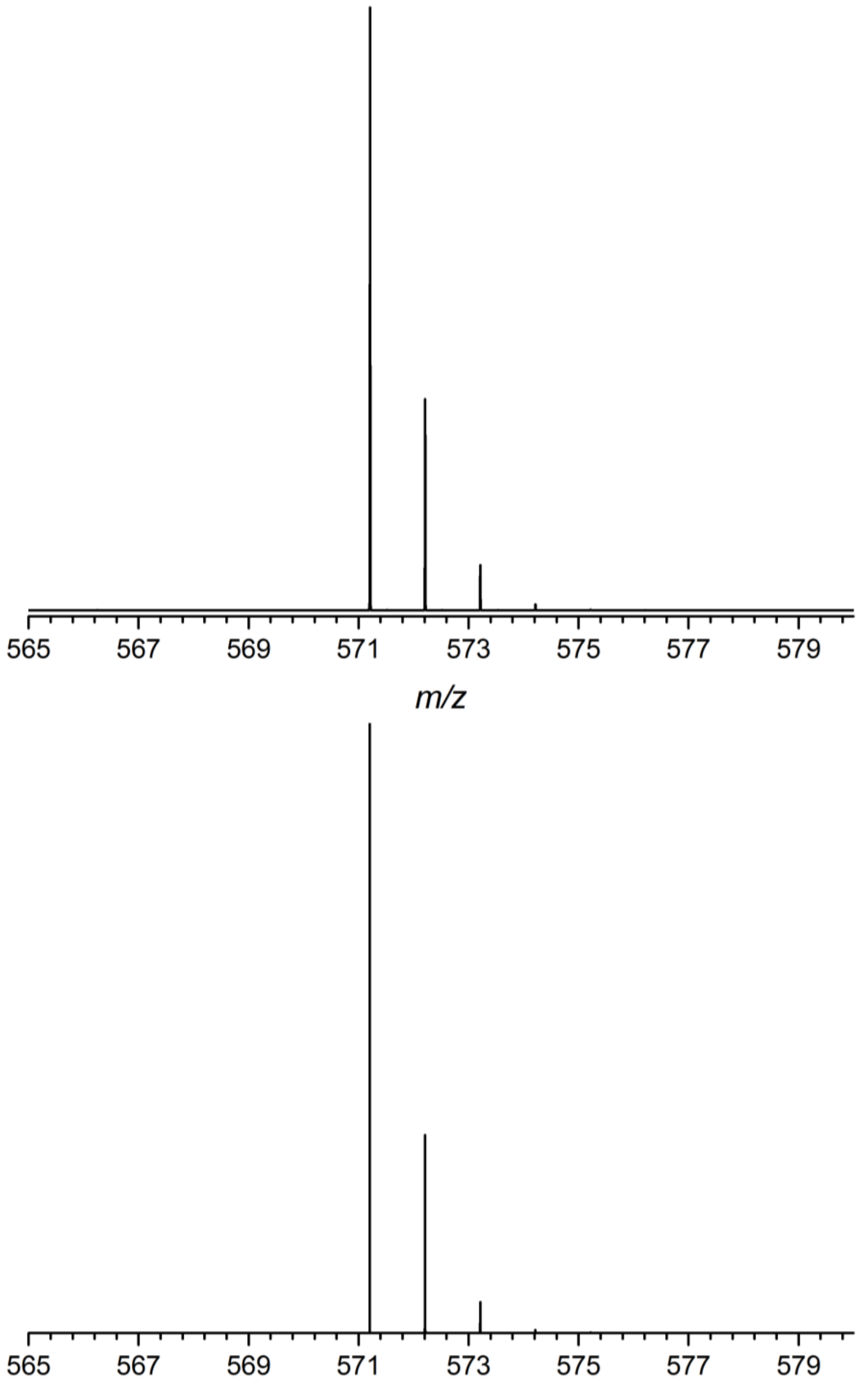

Supplementary Figure 14. ESI-MS experimental (top) and calculated (bottom) zoom spectra of **3**.

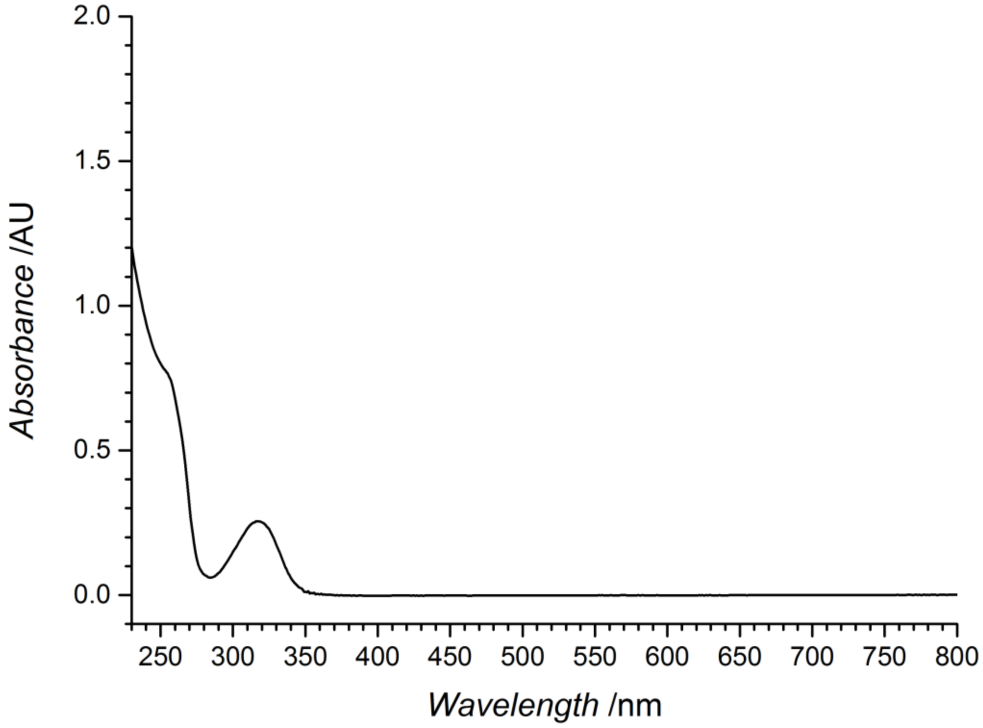

Supplementary Figure 15. UV-VIS spectrum of **3** (0.04 mg mL^{-1} in dry DCM).

Characterization of Synthon 4 (Monomer M$_4$)

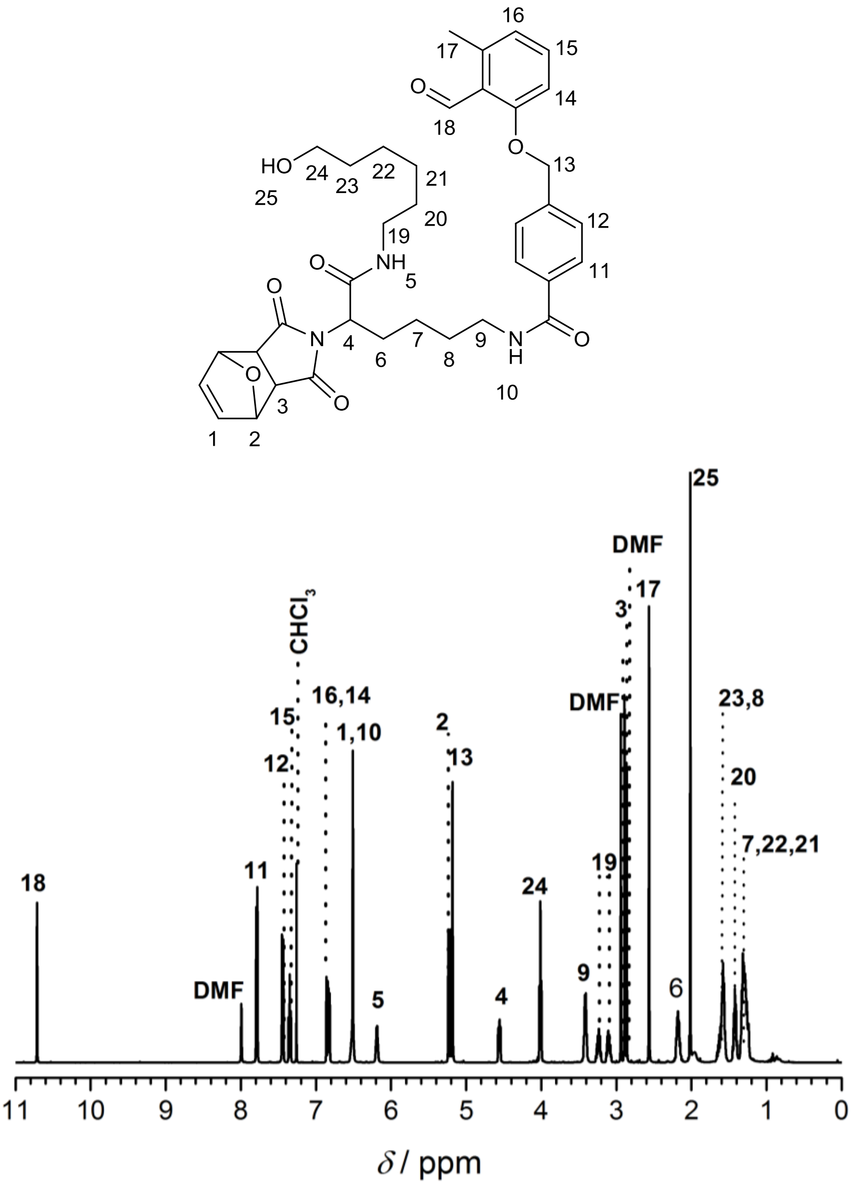

Supplementary Figure 16. ^{1}H NMR spectrum of **4** ($CDCl_3$).

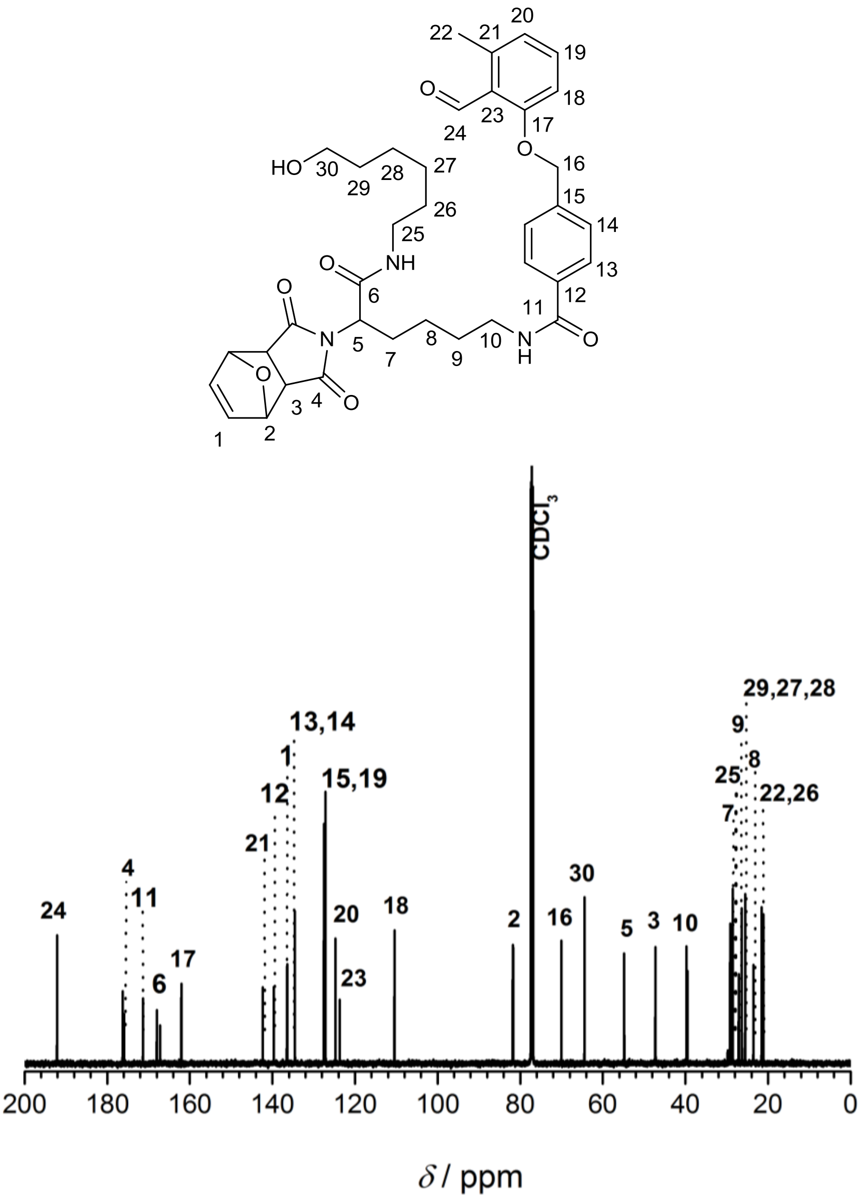

Supplementary Figure 17. ^{13}C NMR spectrum of **4** ($CDCl_3$).

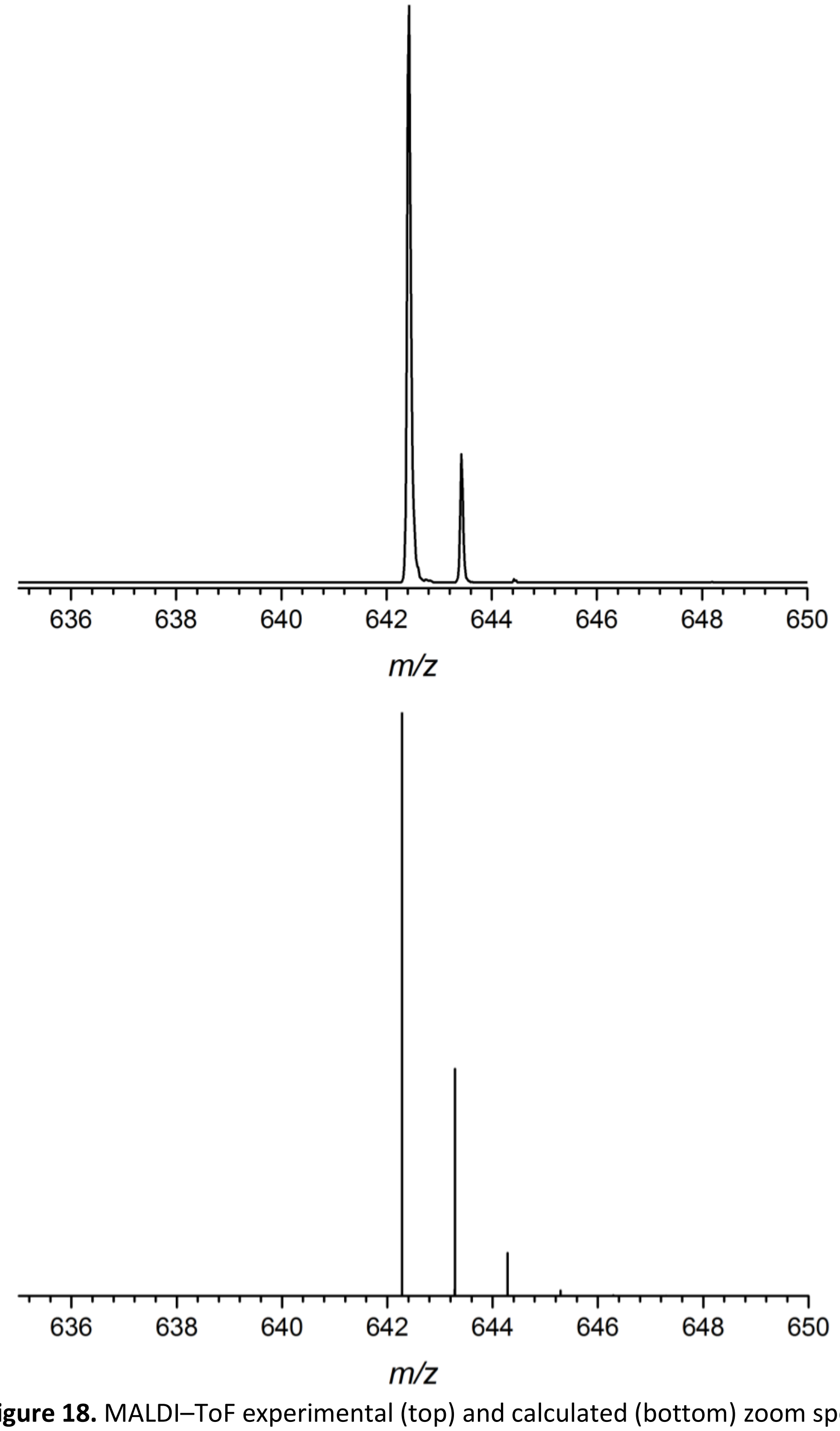

Supplementary Figure 18. MALDI–ToF experimental (top) and calculated (bottom) zoom spectra of **4**.

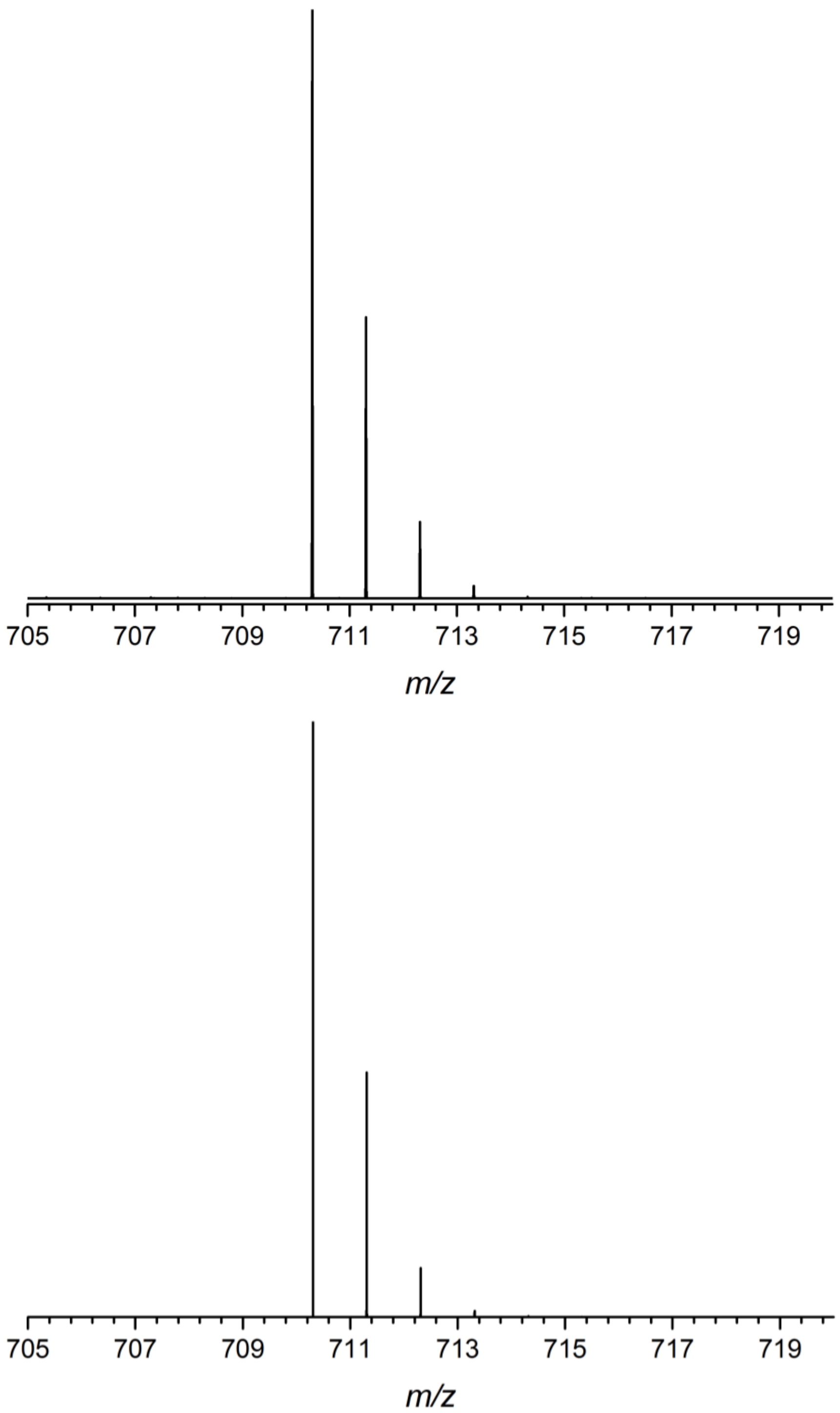

Supplementary Figure 19. ESI-MS experimental (top) and calculated (bottom) zoom spectra of **4**.

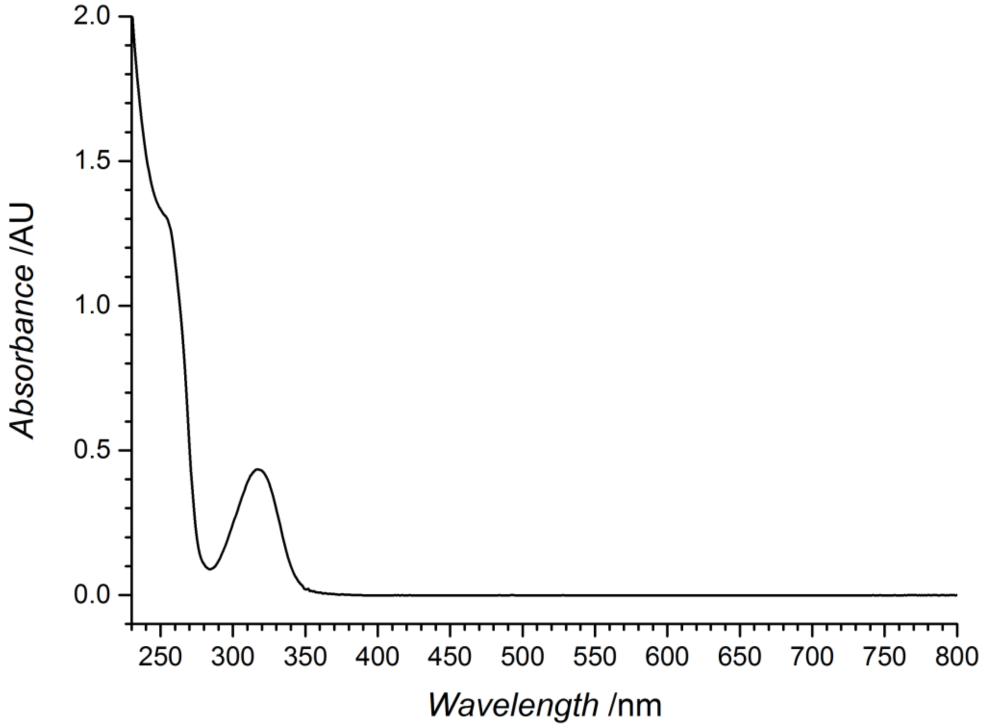

Supplementary Figure 20. UV-VIS spectrum of **4** (0.04 mg mL^{-1} in dry DCM).

Characterization of Synthon 5 (Monomer M_5)

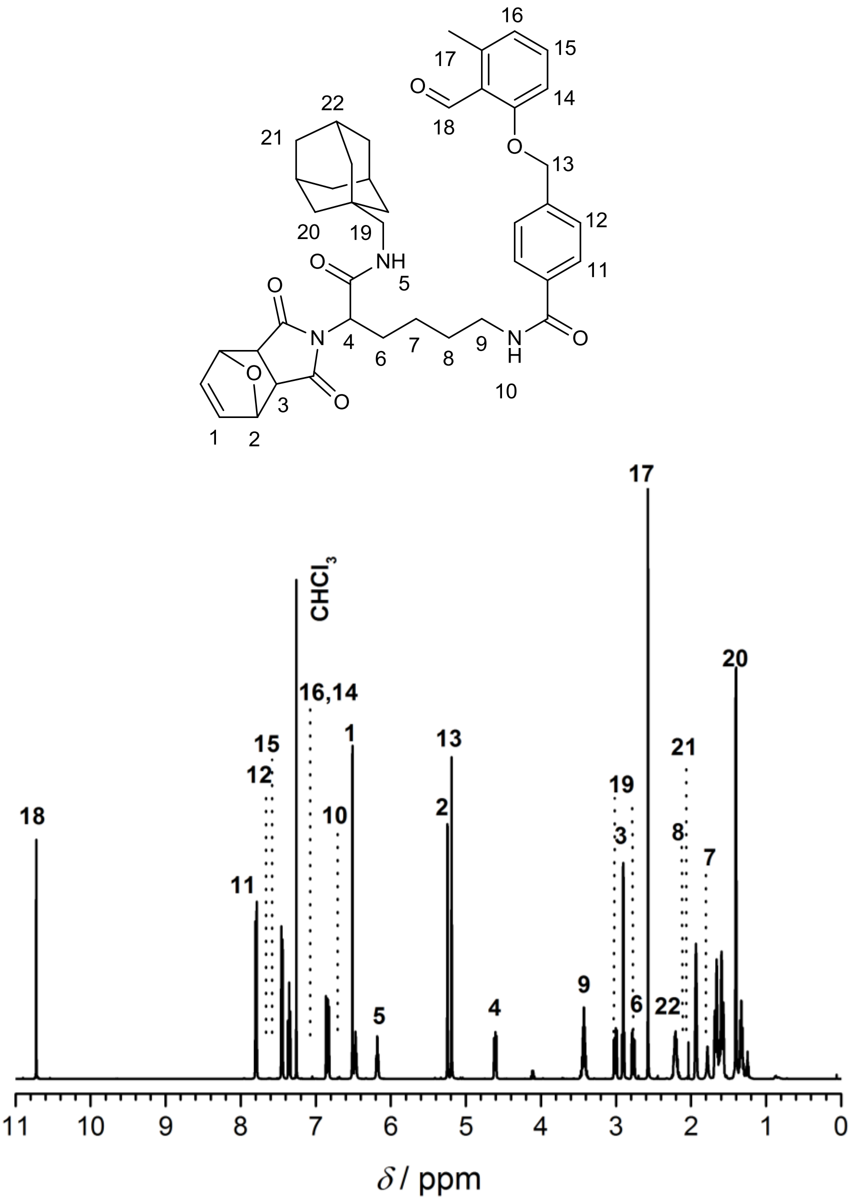

Supplementary Figure 21. ^{1}H NMR spectrum of **5** ($CDCl_3$).

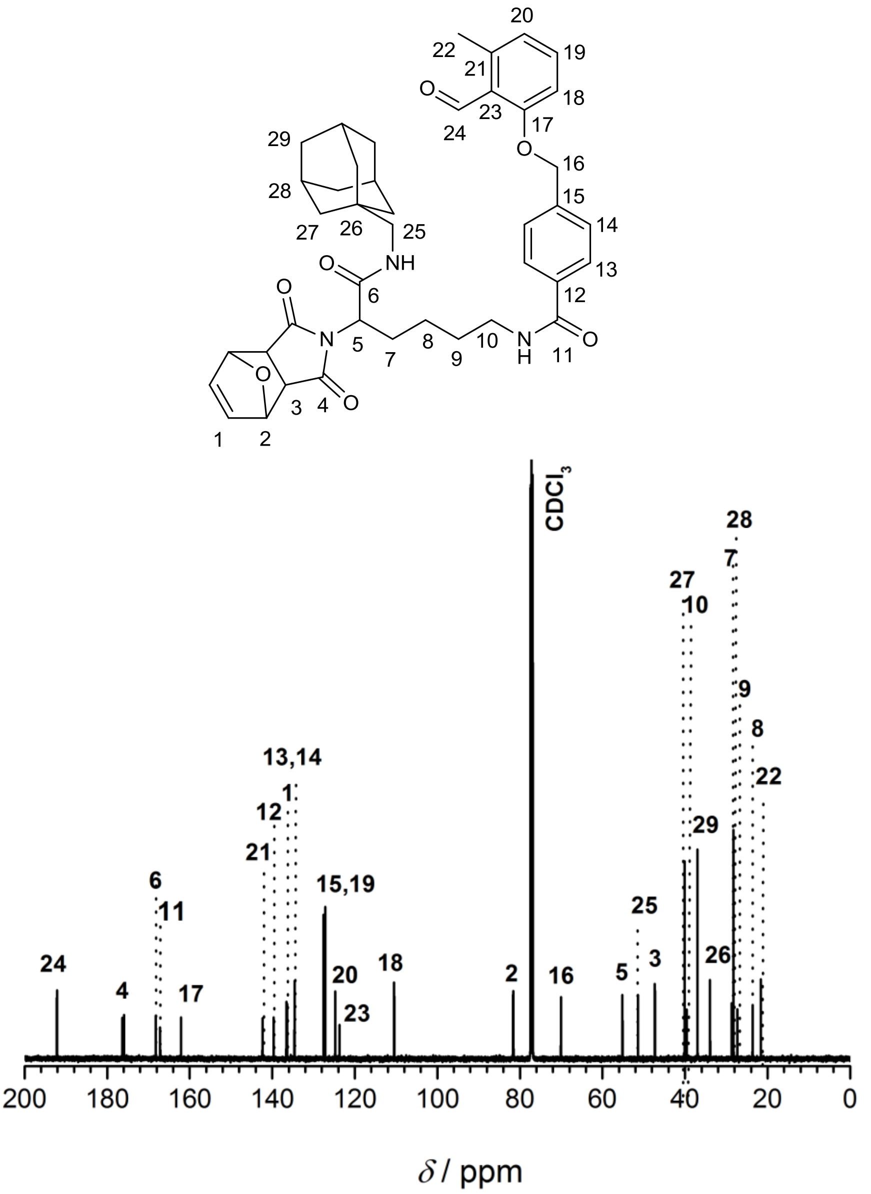

Supplementary Figure 22. ^{13}C NMR spectrum of **5** ($CDCl_3$).

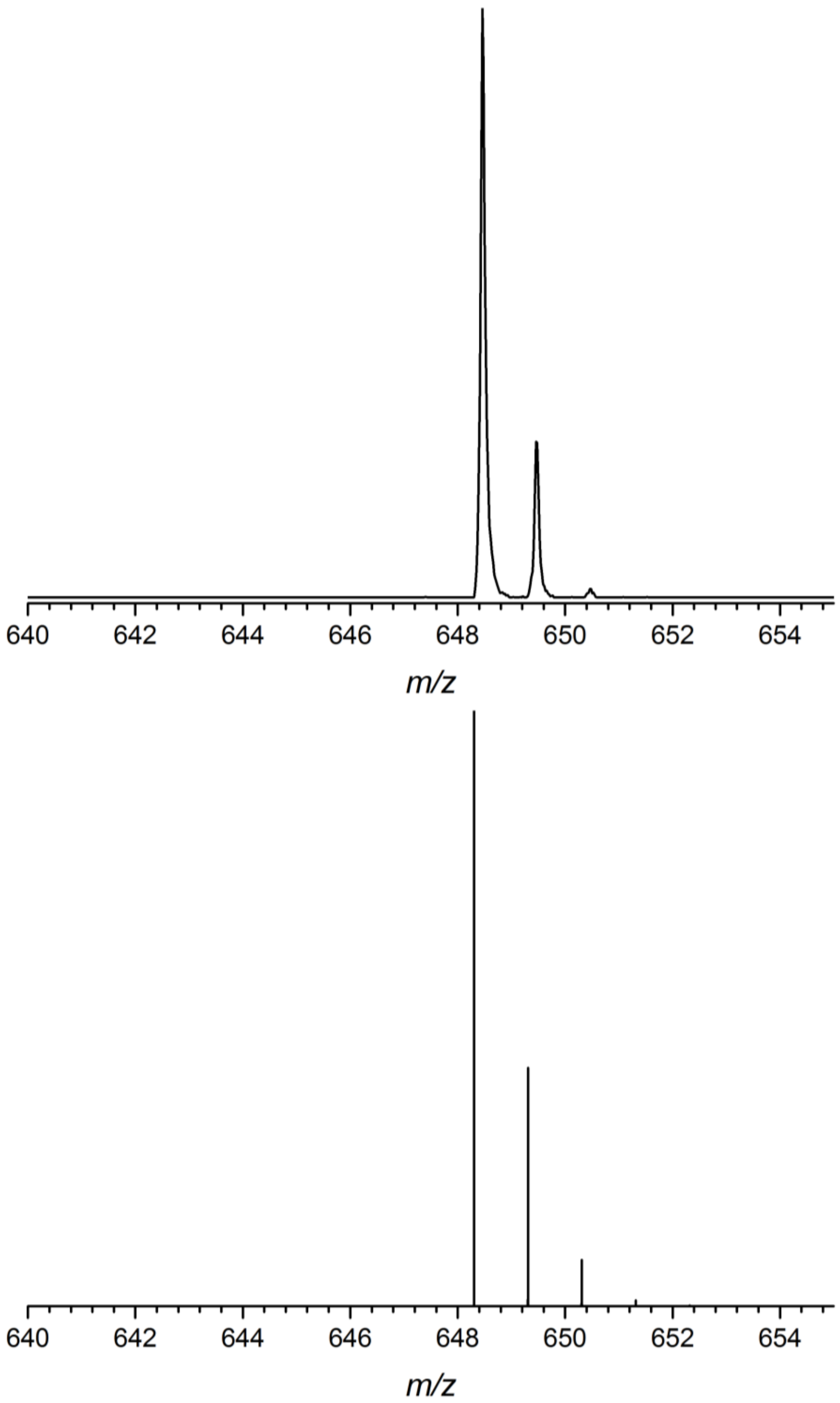

Supplementary Figure 23. MALDI–ToF experimental (top) and calculated (bottom) zoom spectra of **5**.

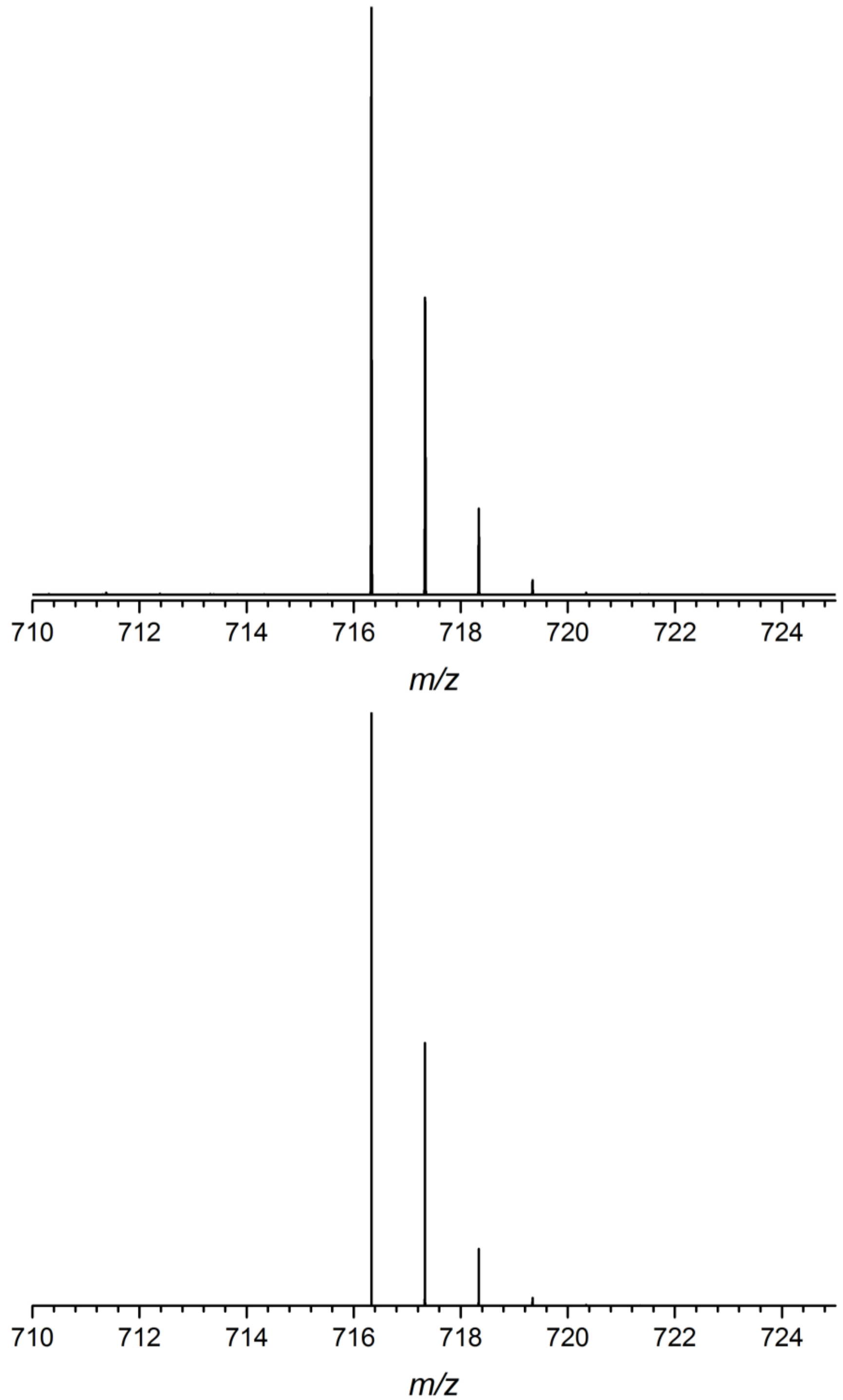

Supplementary Figure 24. ESI-MS experimental (top) and calculated (bottom) zoom spectra of **5**.

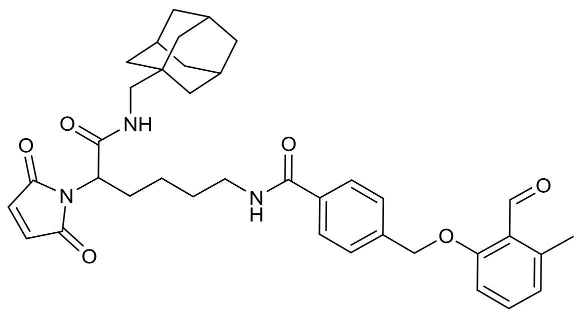

A$_1$

Supplementary Figure 25. Molecule identified in Supplementary Supplementary Table **5** (**A$_1$**).

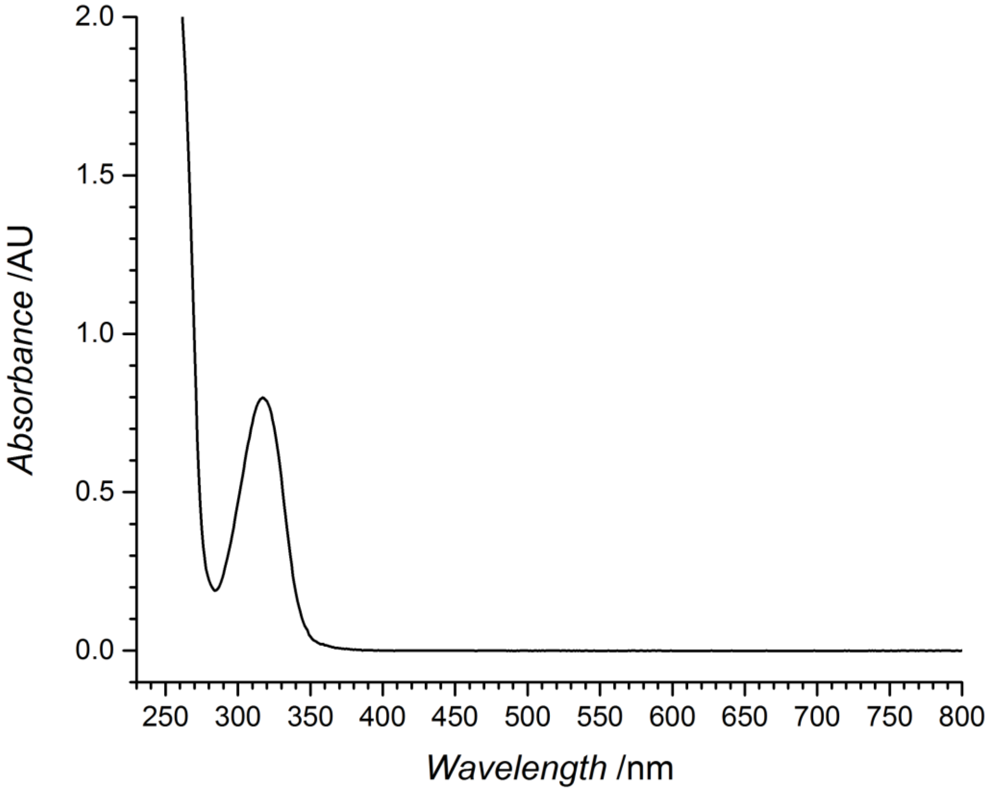

Supplementary Figure 26. UV-VIS spectrum of **5** (0.04 mg mL^{-1} in dry DCM).

Characterization of Synthon 6 (Monomer M$_6$)

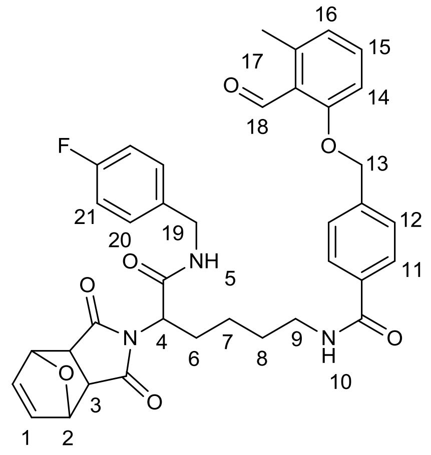

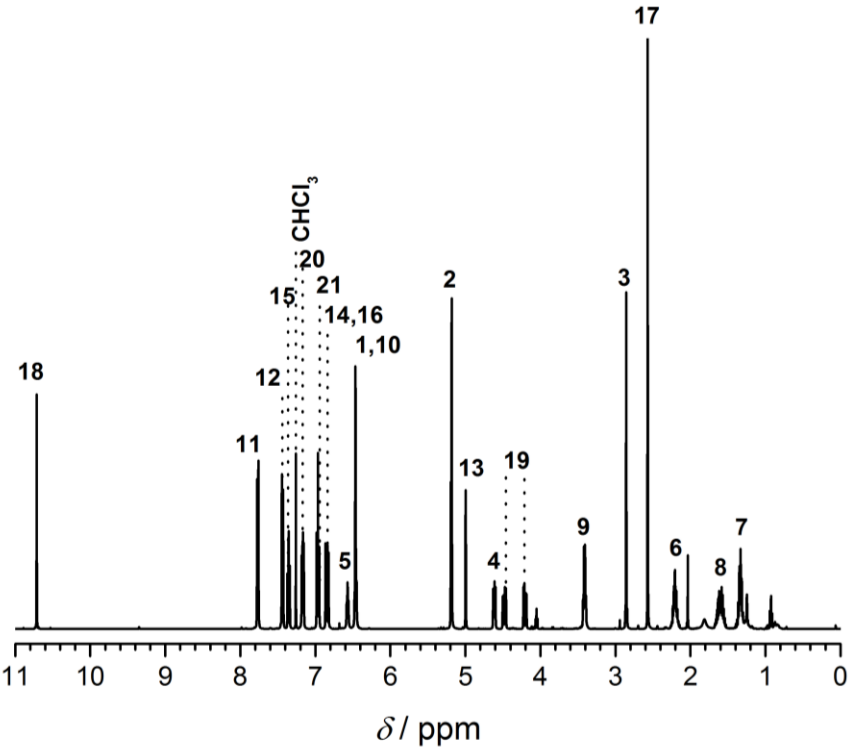

Supplementary Figure 27. ^{1}H NMR spectrum of **6** ($CDCl_3$).

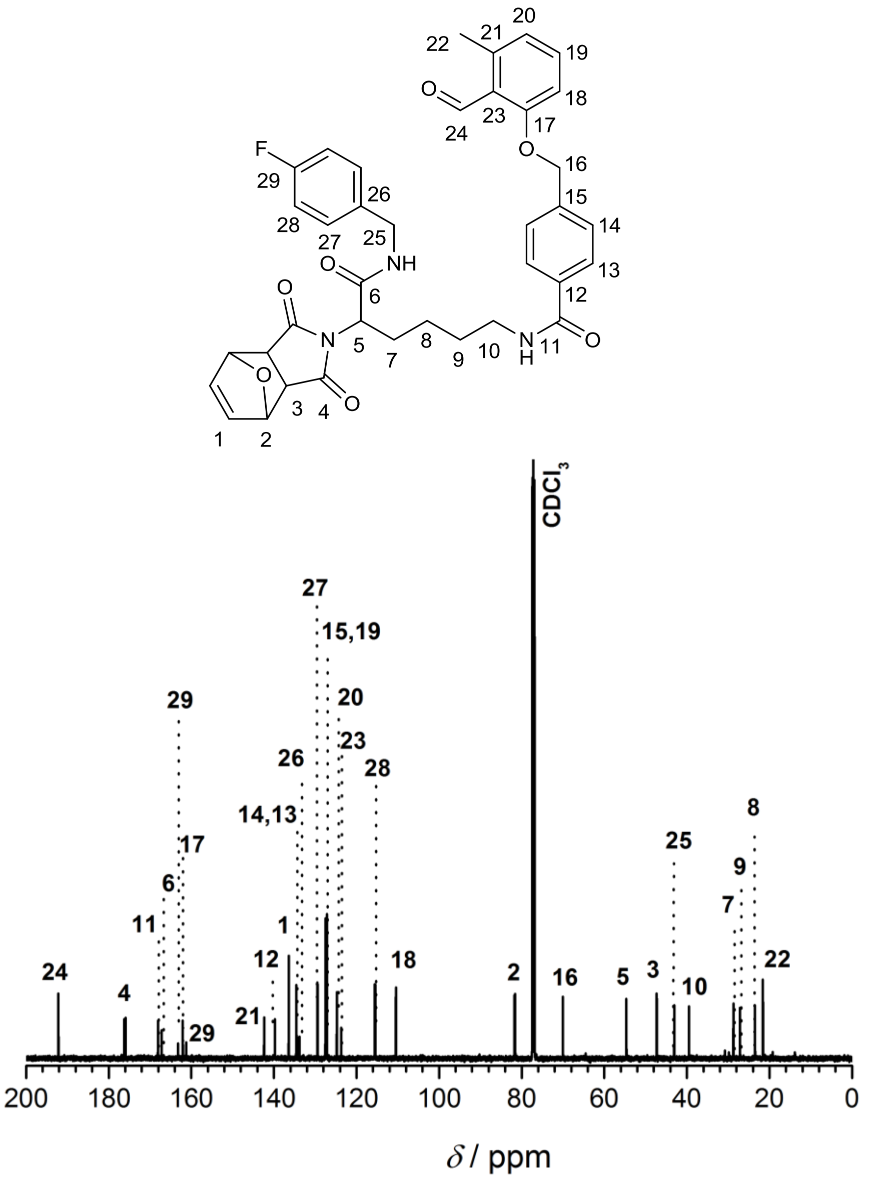

Supplementary Figure 28. ^{13}C NMR spectrum of **6** ($CDCl_3$).

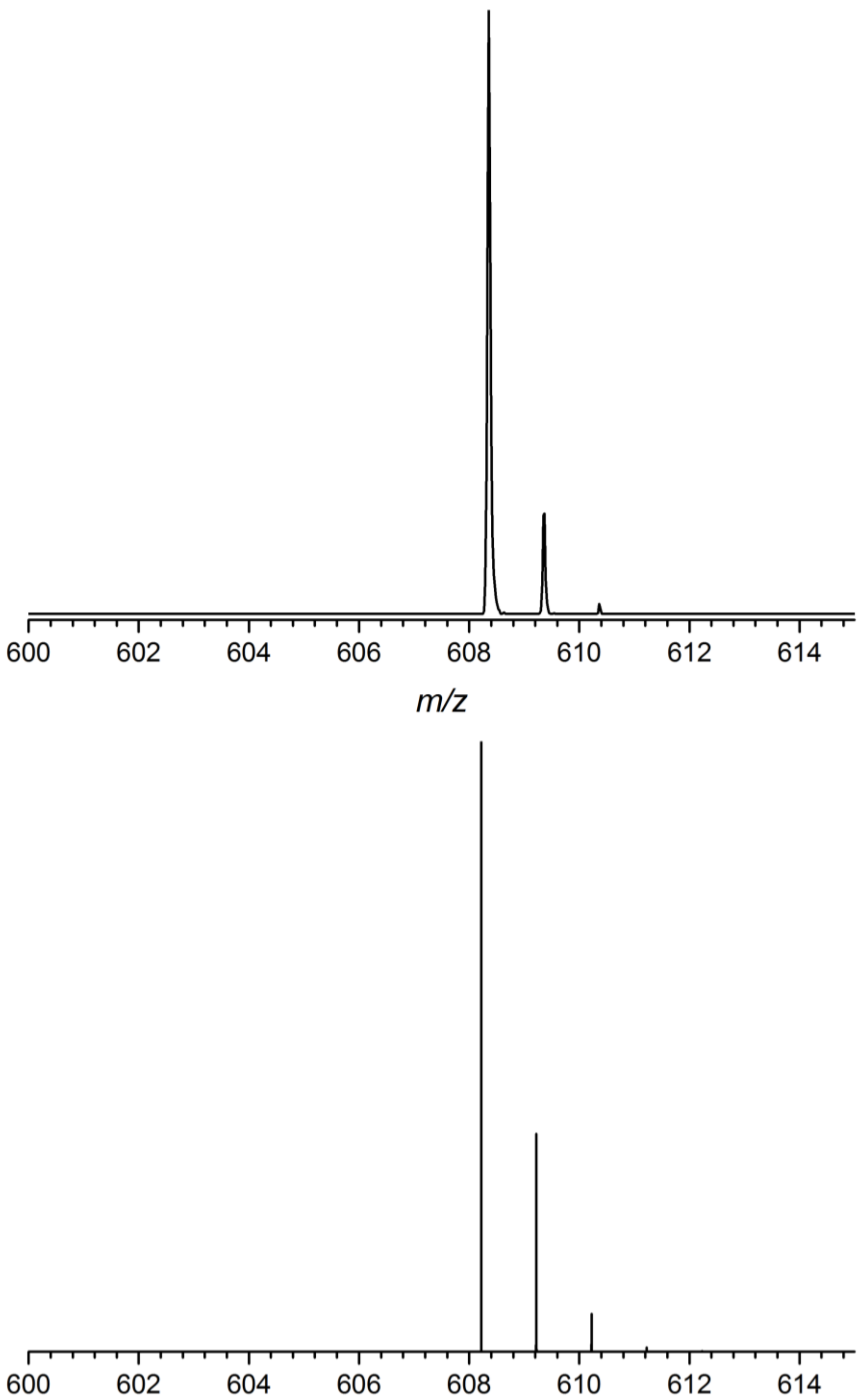

Supplementary Figure 29. MALDI–ToF experimental (top) and calculated (bottom) zoom spectra of **6**.

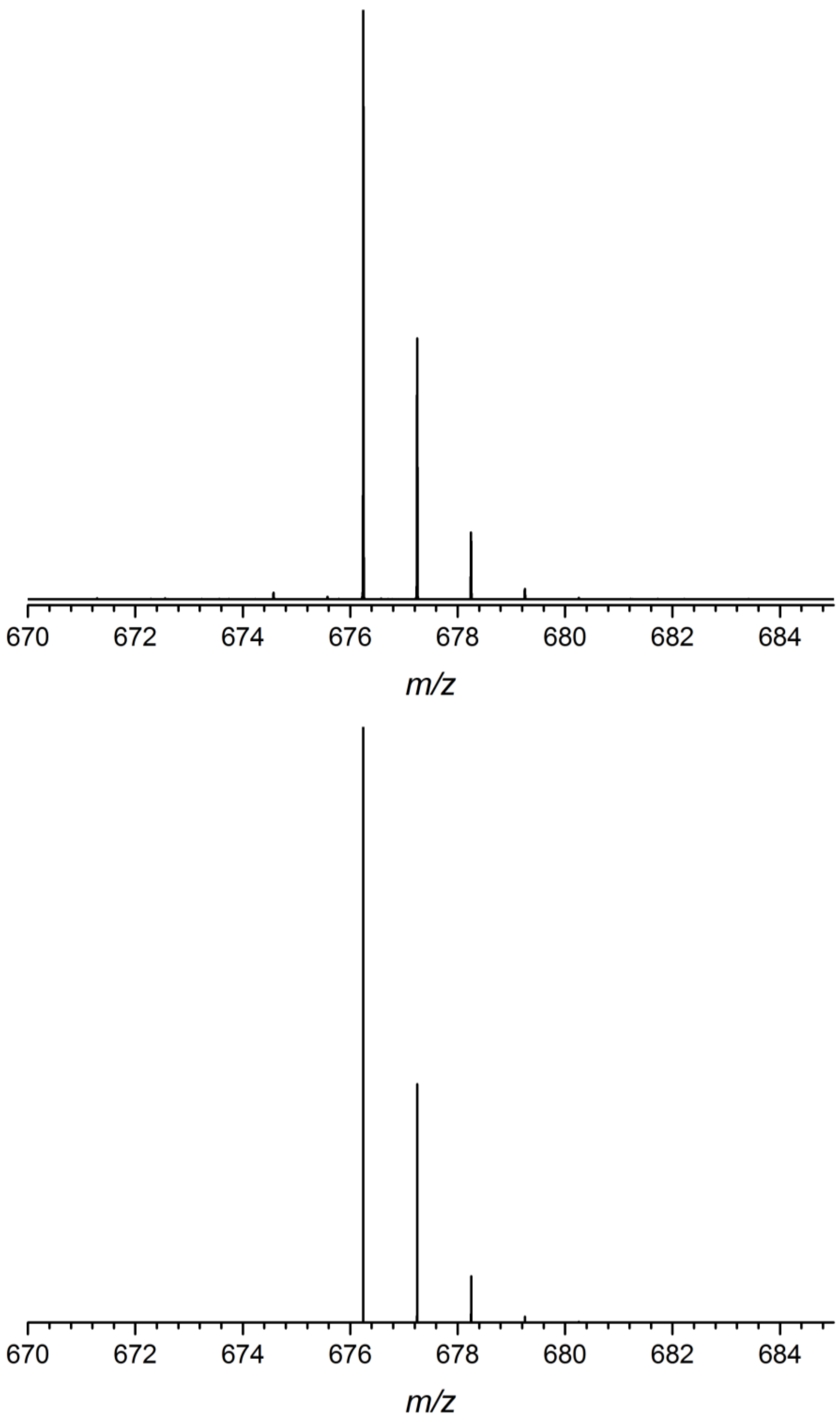

Supplementary Figure 30. ESI-MS experimental (top) and calculated (bottom) zoom spectra of **6**.

.

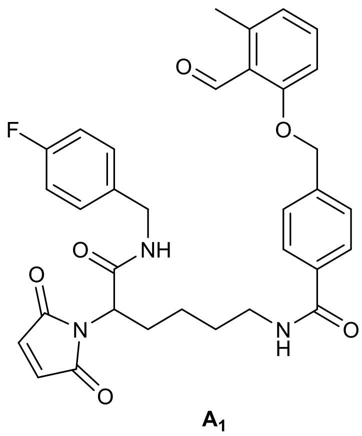

$\mathbf{A_1}$

Supplementary Figure 31. Molecule identified in Supplementary Supplementary Table **6** ($\mathbf{A_1}$).

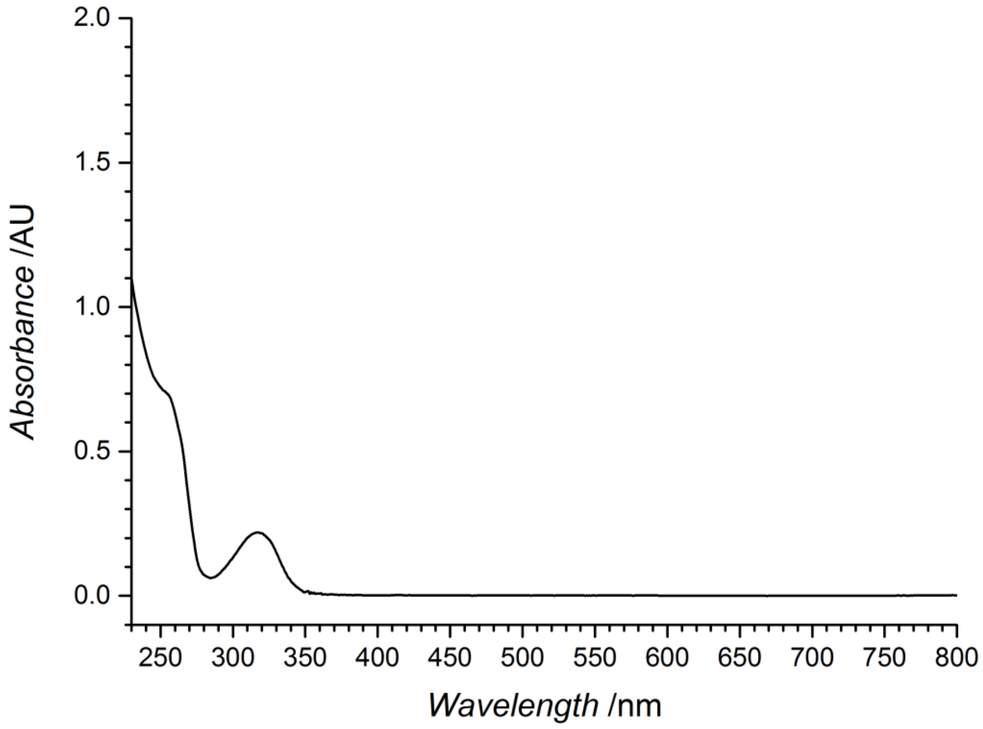

Supplementary Figure 32. UV-VIS spectrum of **6** (0.04 mg mL^{-1} in dry DCM).

Characterization of Acetal Monomers 1d, 2e, 3d, 5e

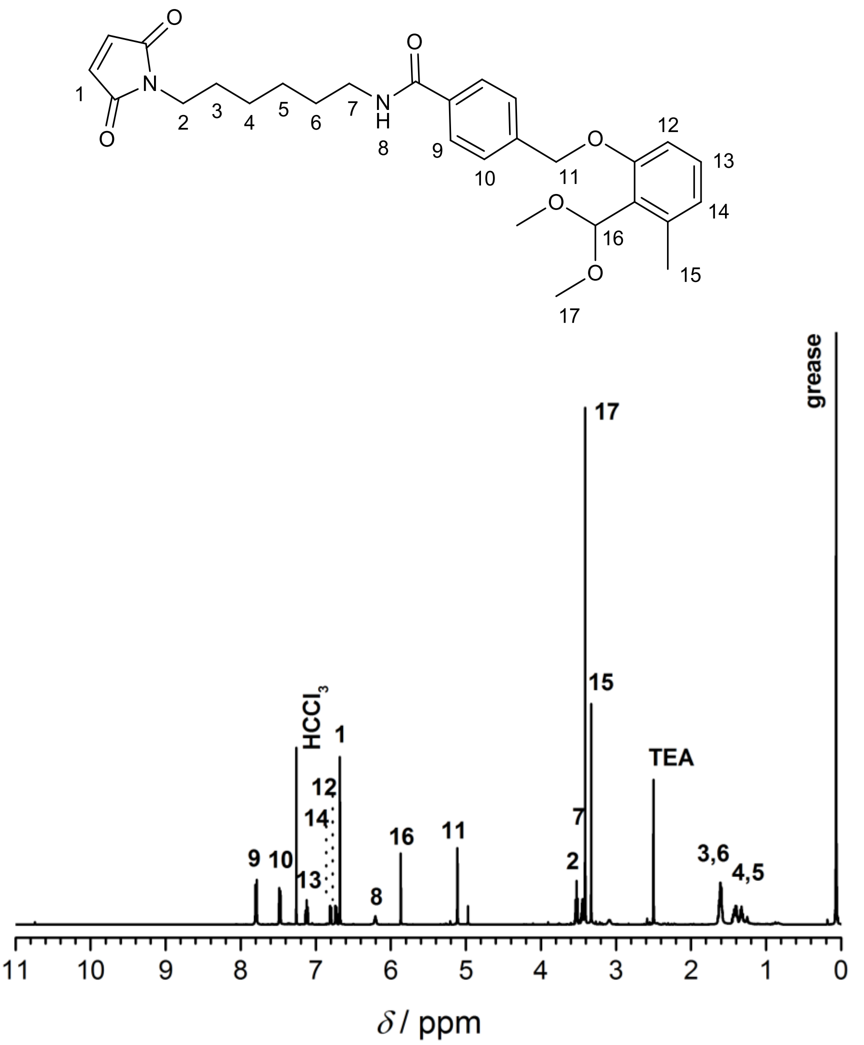

Supplementary Figure 33. ^{1}H NMR spectrum of **1d** ($CDCl_3$).

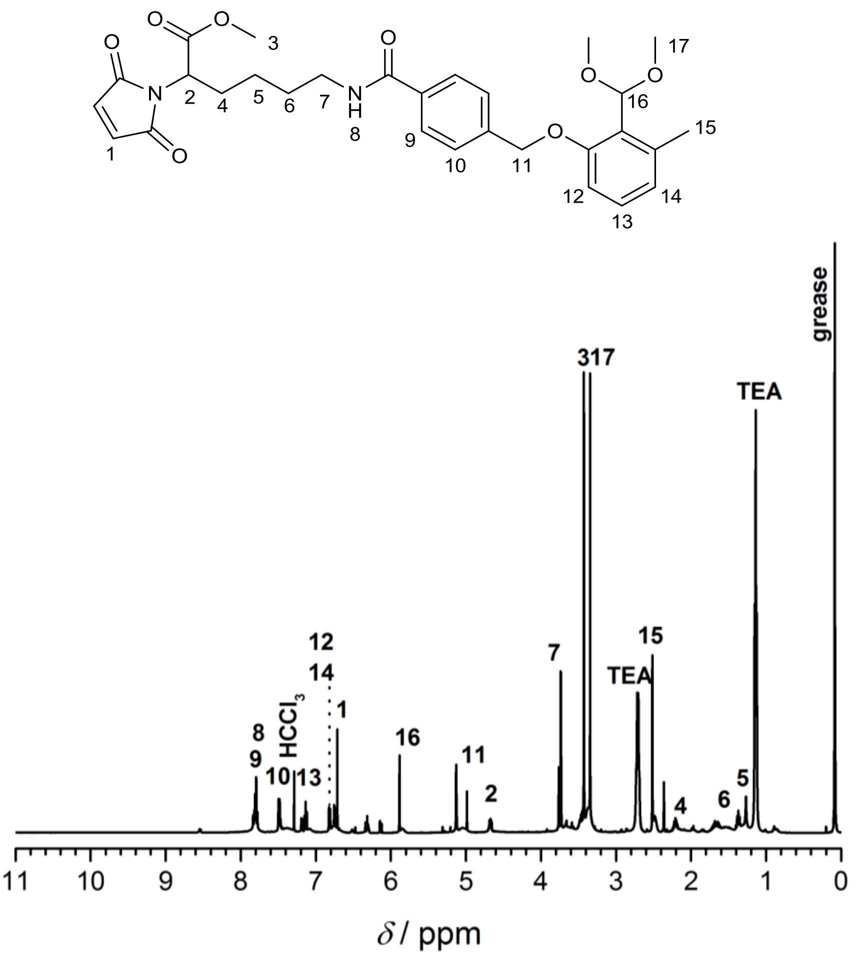

Supplementary Figure 34. ^{1}H NMR spectrum of **2e** ($CDCl_3$).

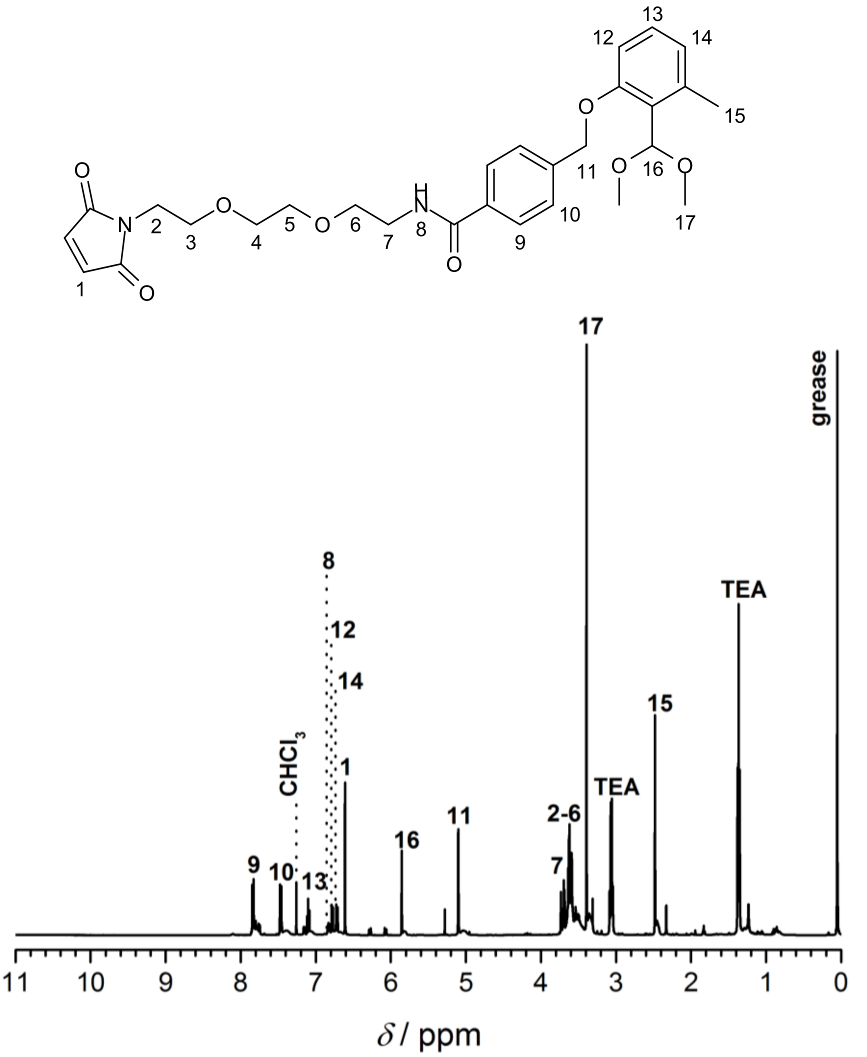

Supplementary Figure 35. ^{1}H NMR spectrum of **3d** ($CDCl_3$).

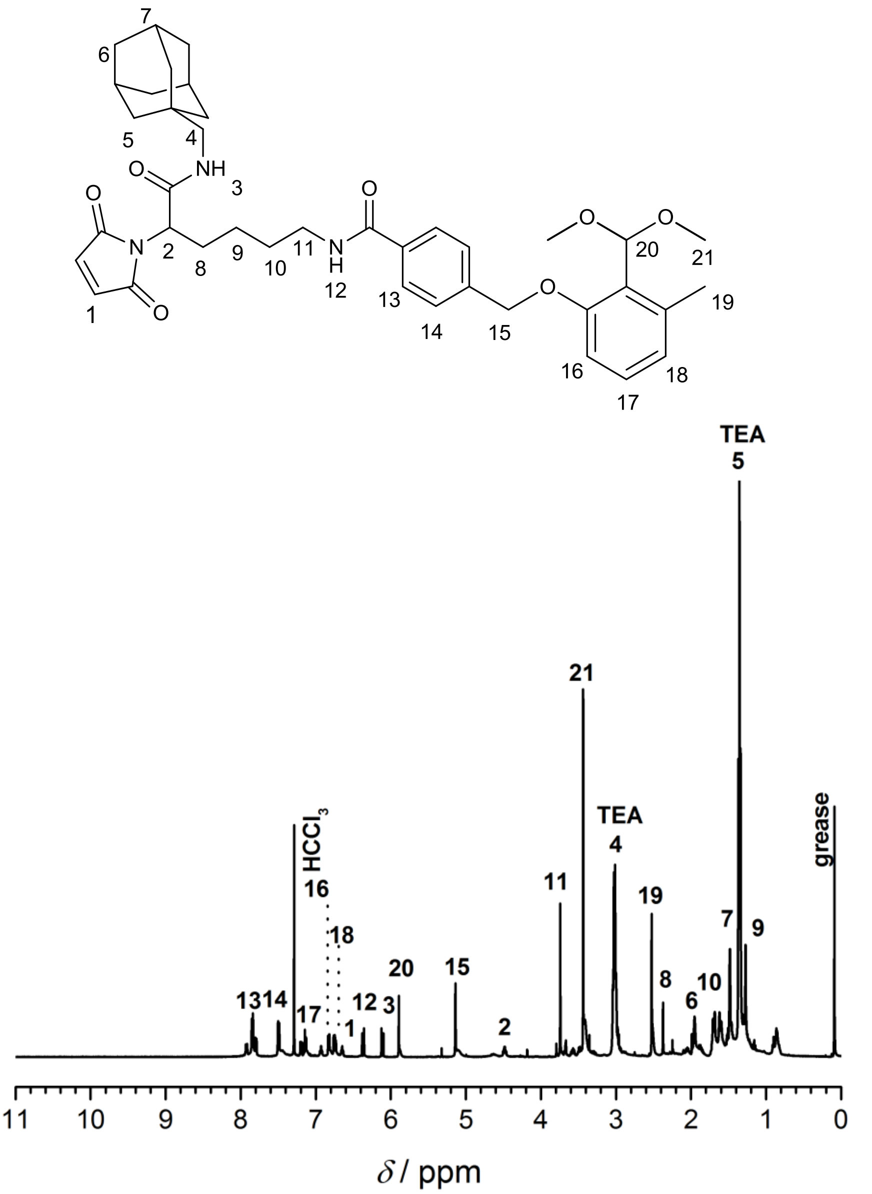

Supplementary Figure 36. ^{1}H NMR spectrum of **5e** ($CDCl_3$).

Photoreaction and Photo-Reactor

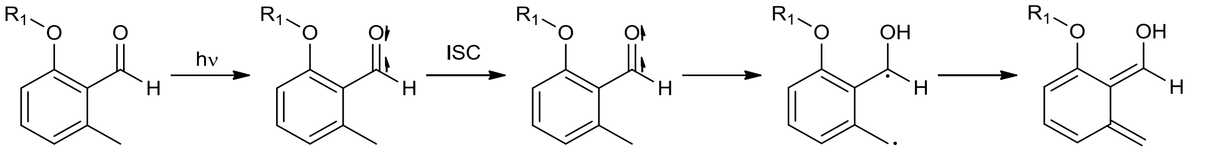

Supplementary Figure 37. Mechanism of the photoenol-driven photoreaction for the generation of a highly reactive diene (ortho-quinodimethane).

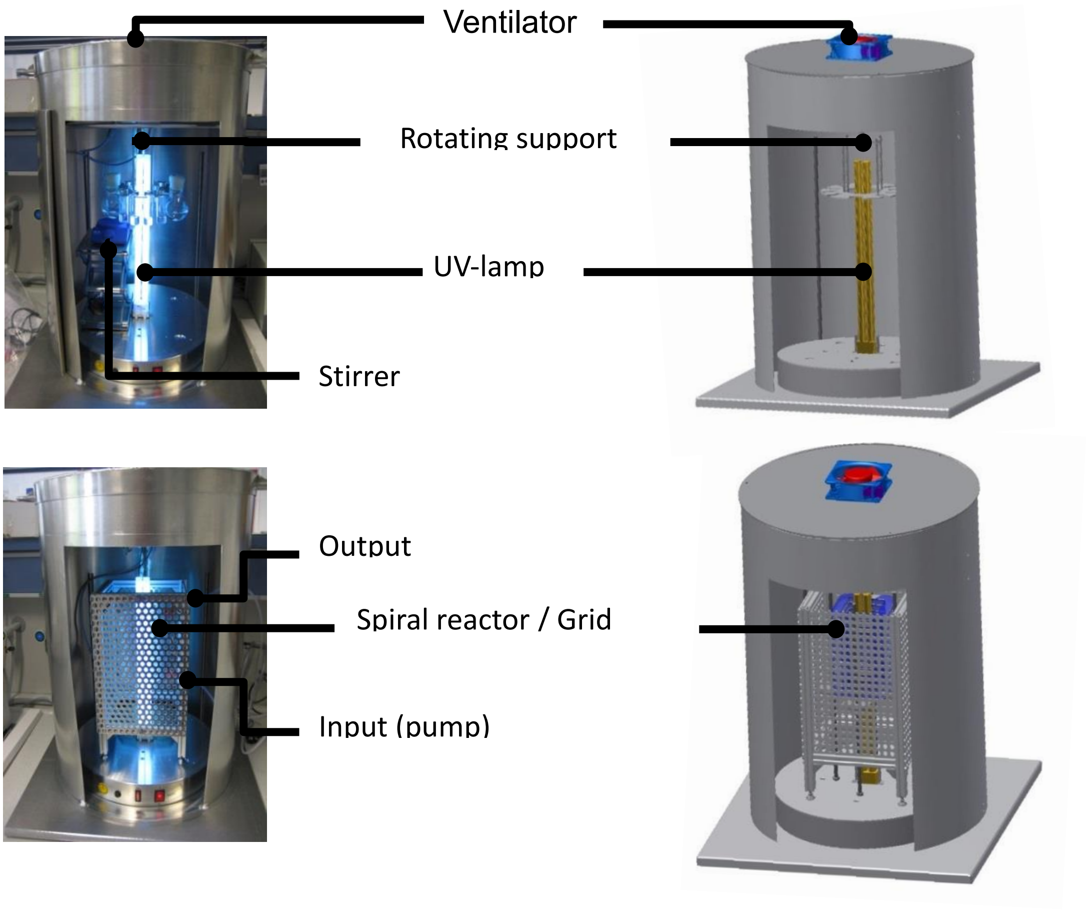

Supplementary Figure 38. Schematic drawings and photographs of the batch reaction set-up (top) and flow reactor (bottom).

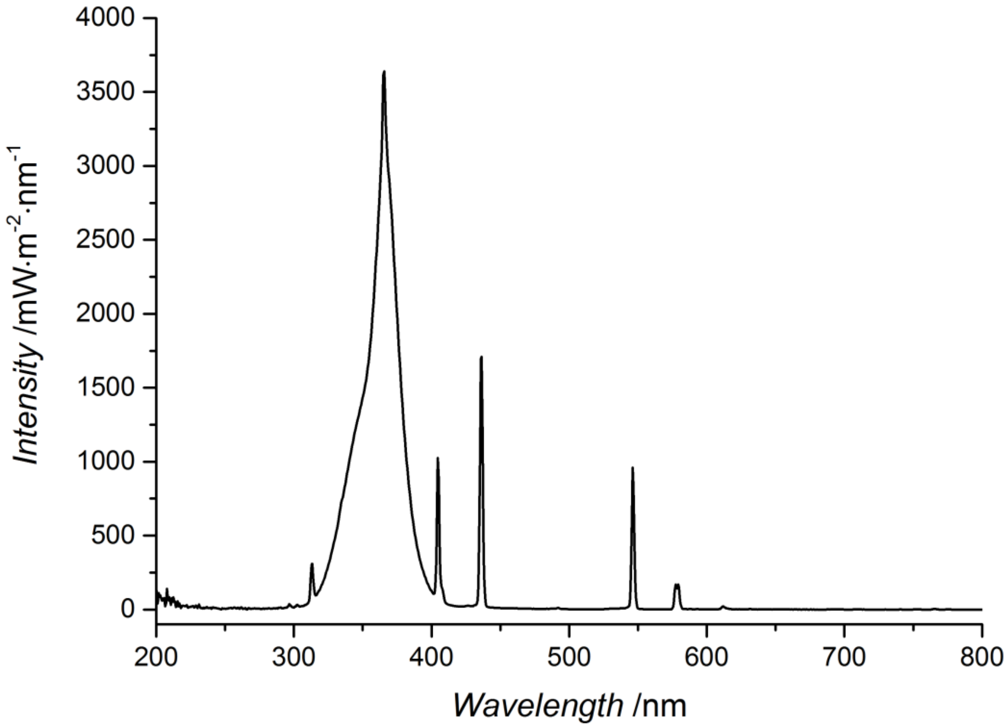

Supplementary Figure 39. Emission spectrum of the employed PL-L lamp.

Characterization of symmetric Homopolymer 7 (($(M_1)_5$-X-$(M_1)_5$) and precursors 7a-d (7a: (M_1)-X-(M_1), 7b: $(M_1)_2$-X-$(M_1)_2$, 7c: $(M_1)_3$-X-$(M_1)_3$, 7d: $(M_1)_2$)

Characterization of 7a (M_1)-X-(M_1)

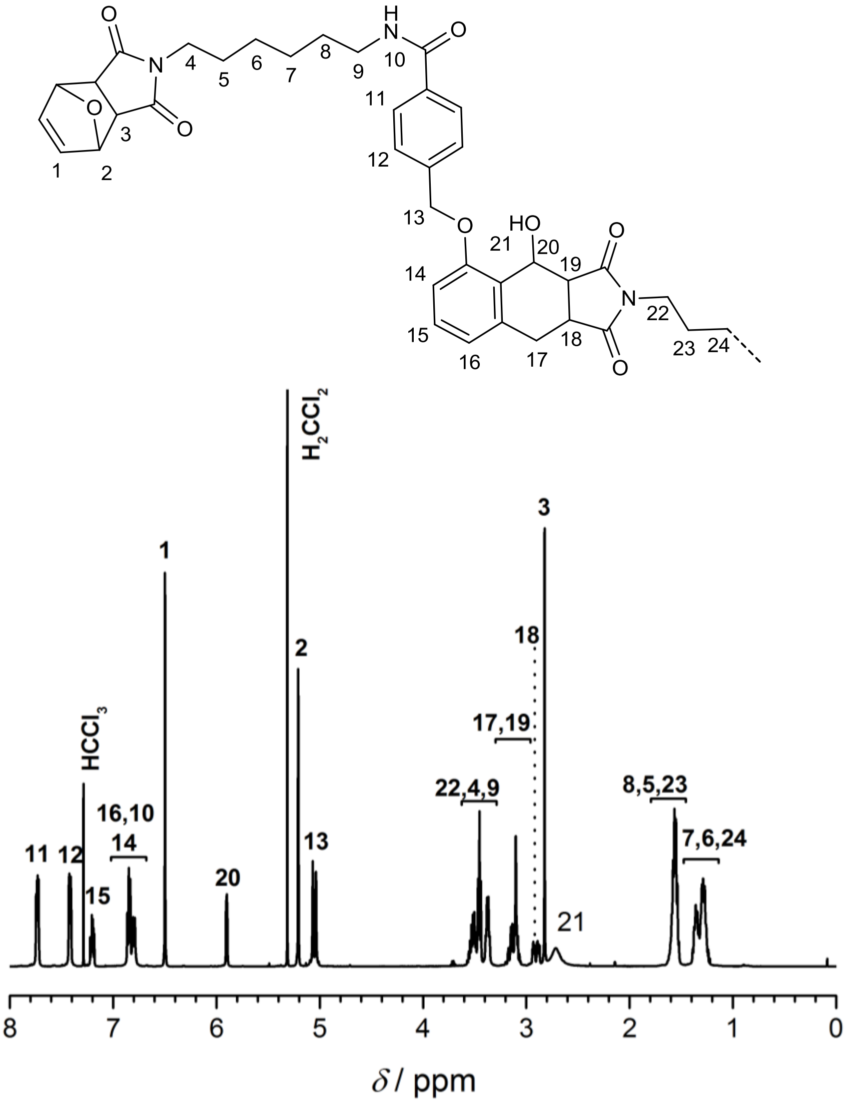

Supplementary Figure 40. ^{1}H NMR spectrum of **7a** ($CDCl_3$).

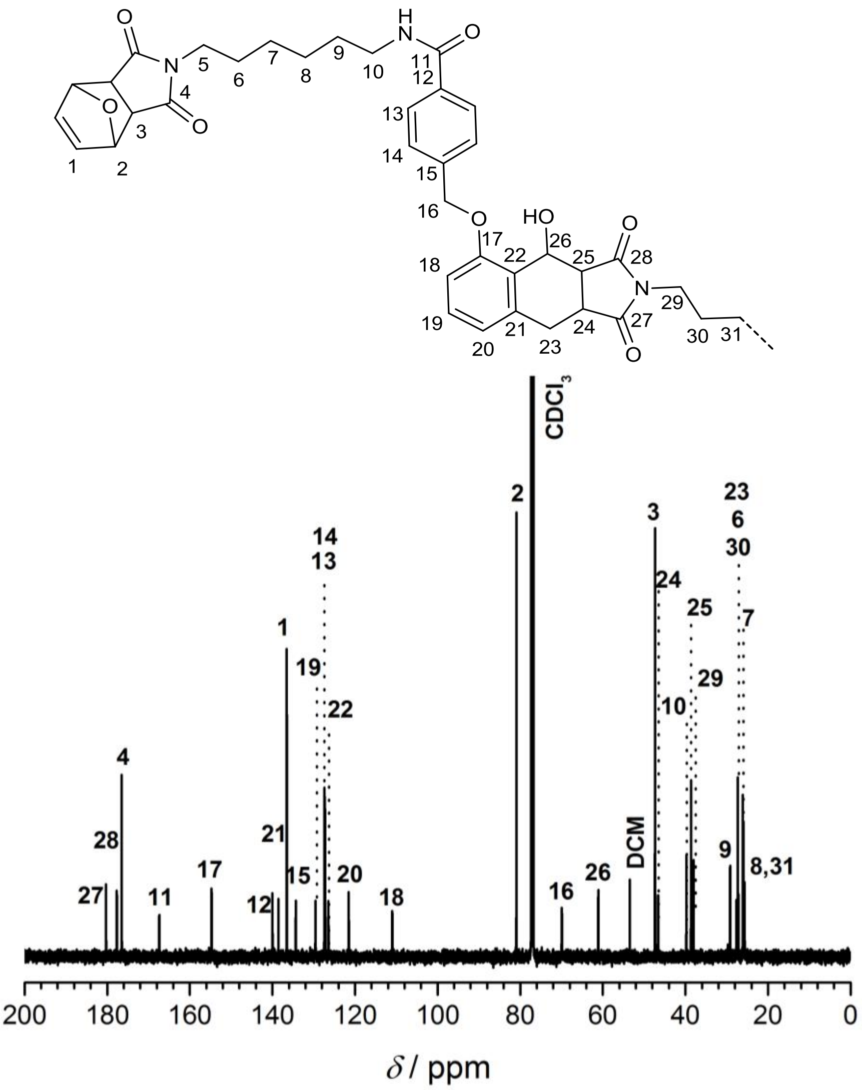

Supplementary Figure 41. ^{13}C NMR spectrum of **7a** ($CDCl_3$).

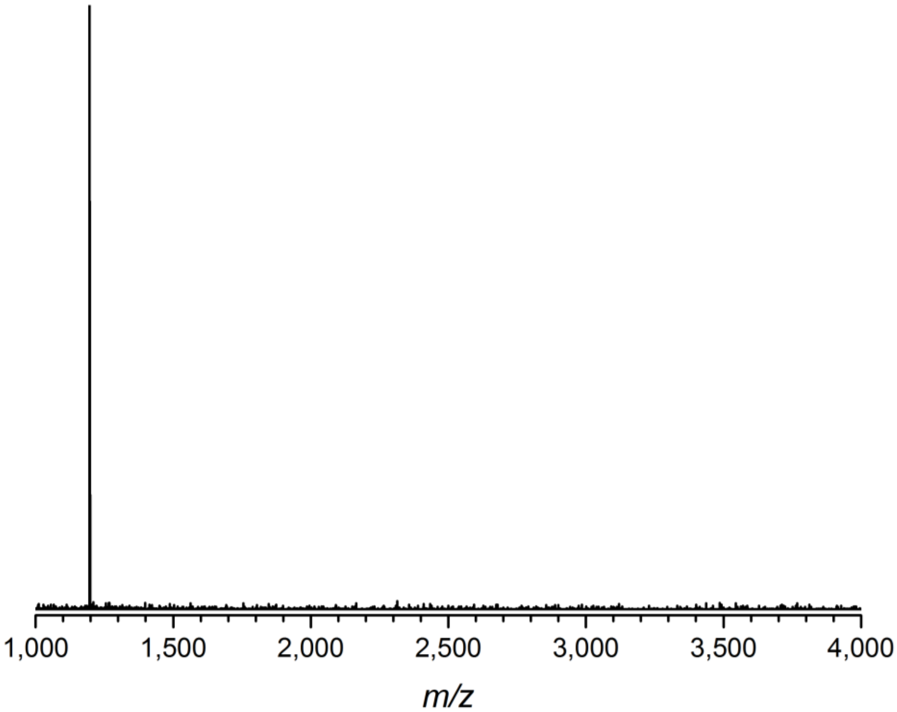

Supplementary Figure 42. MALDI–ToF overview spectrum of **7a**. The peak assignment can be found in Supplementary Table 7.

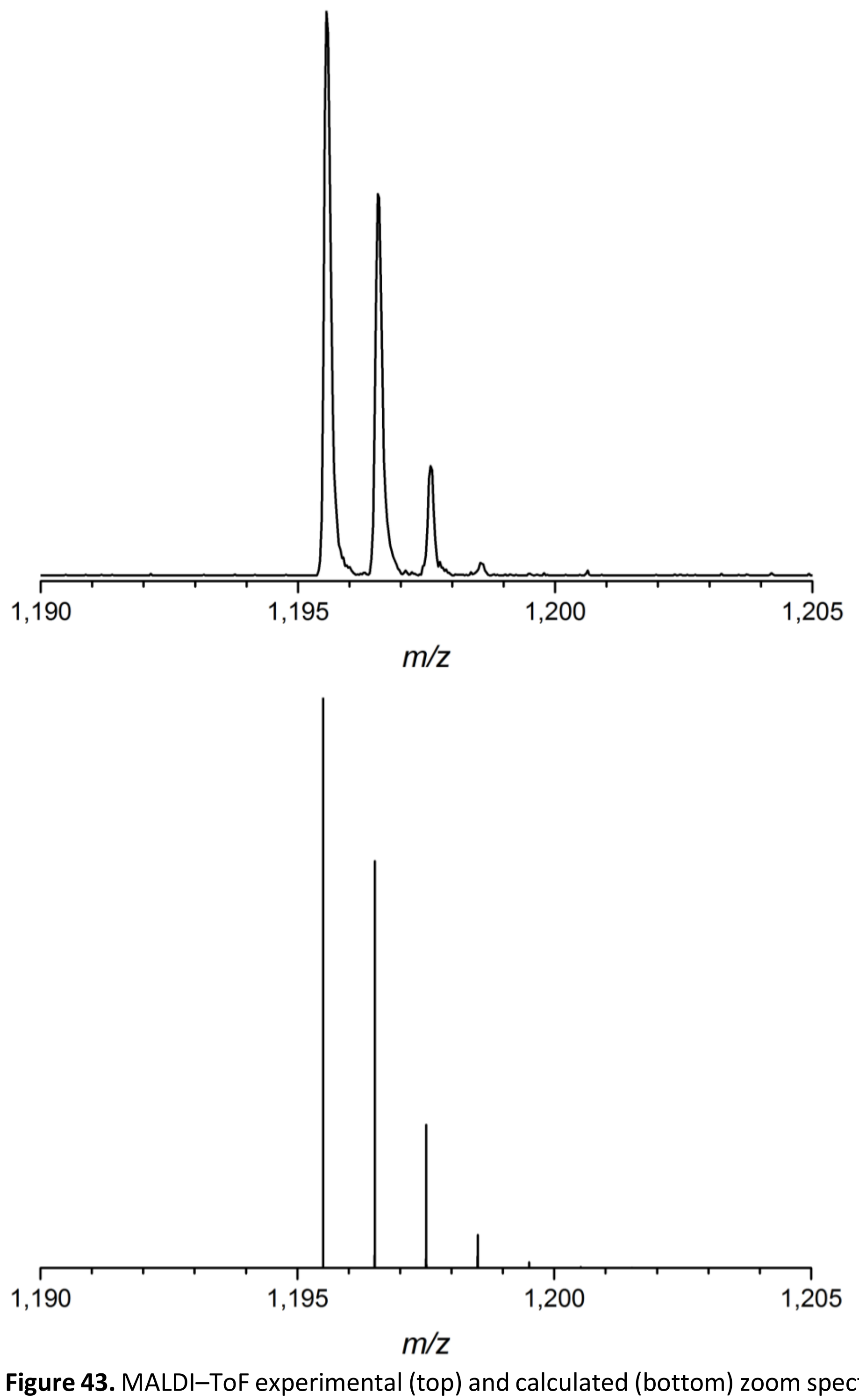

Supplementary Figure 43. MALDI–ToF experimental (top) and calculated (bottom) zoom spectra of **7a**.

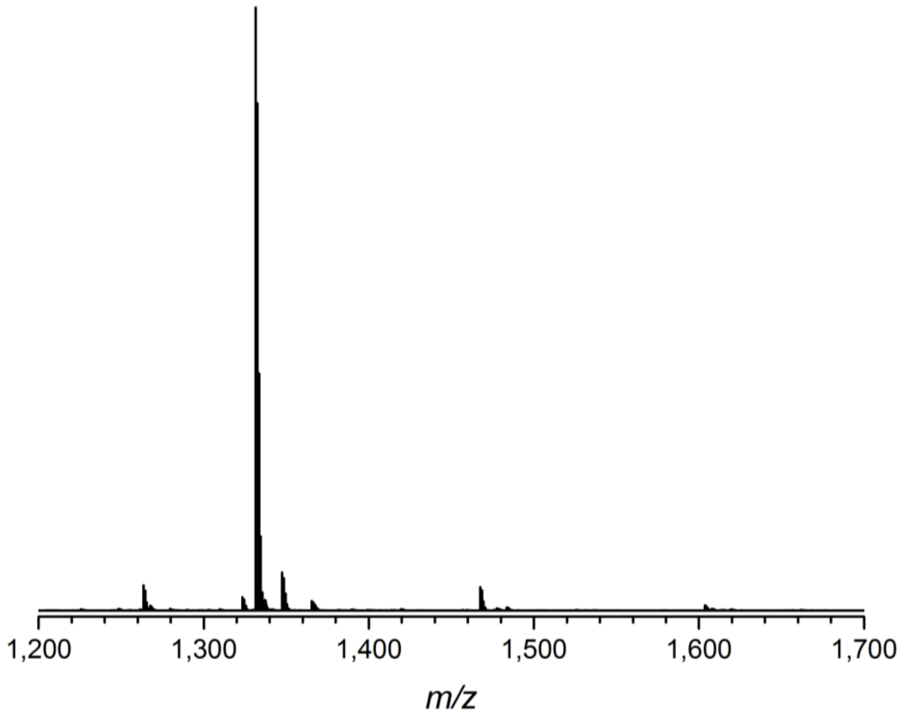

Supplementary Figure 44. ESI-MS overview spectrum of **7a**. All major peaks belong to the target molecule with different counter ions. All peak assignments can be found in Supplementary Table 7.

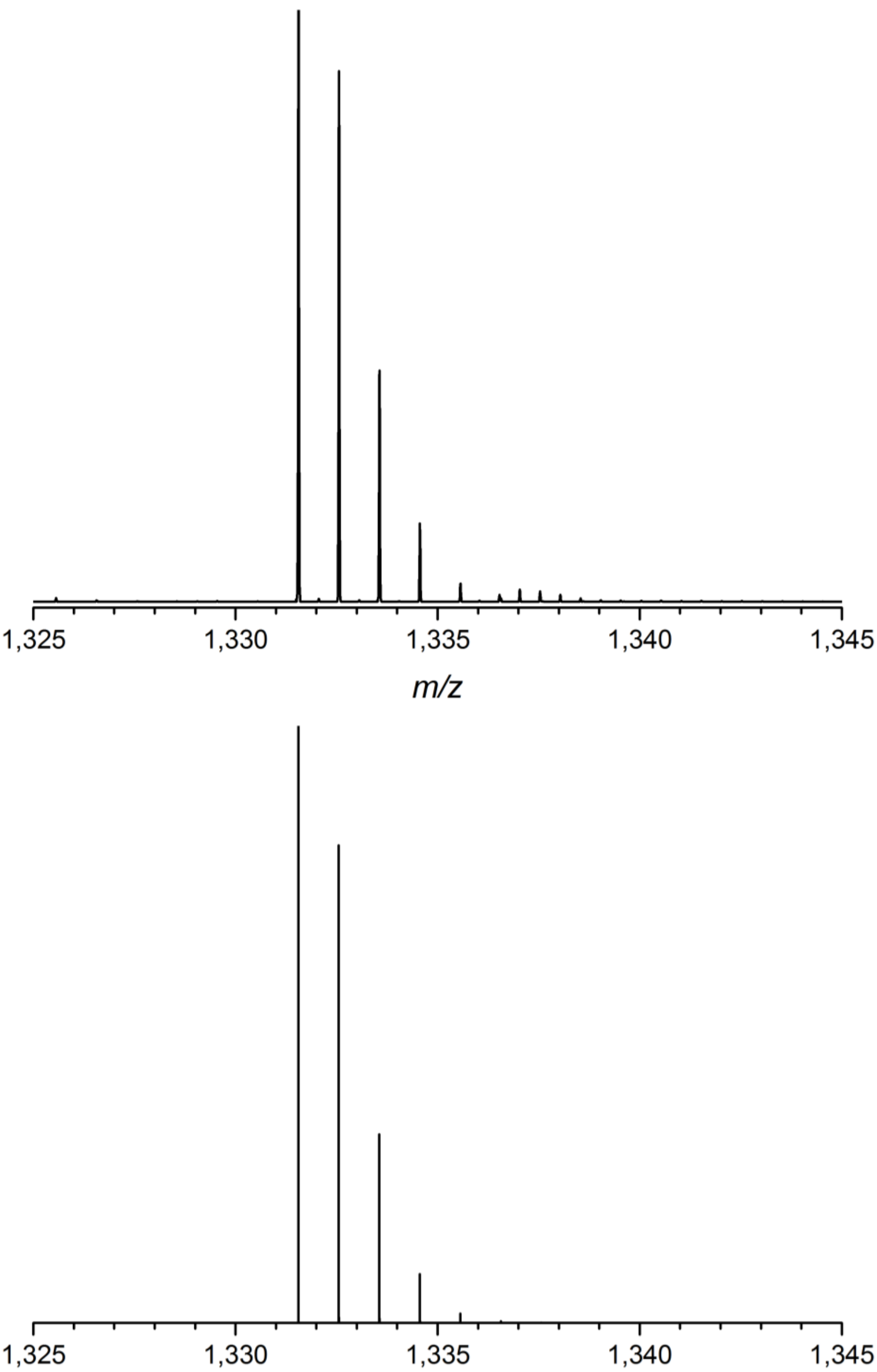

Supplementary Figure 45. ESI-MS experimental (top) and calculated (bottom) zoom spectra of **7a**.

Characterization of 7b $(M_1)_2$-X-$(M_1)_2$

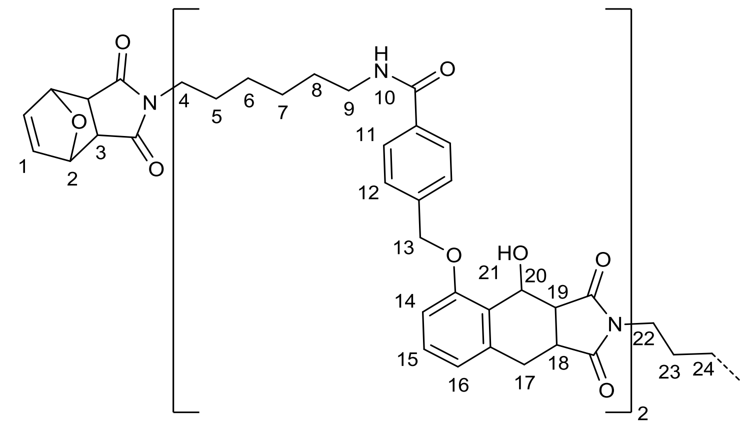

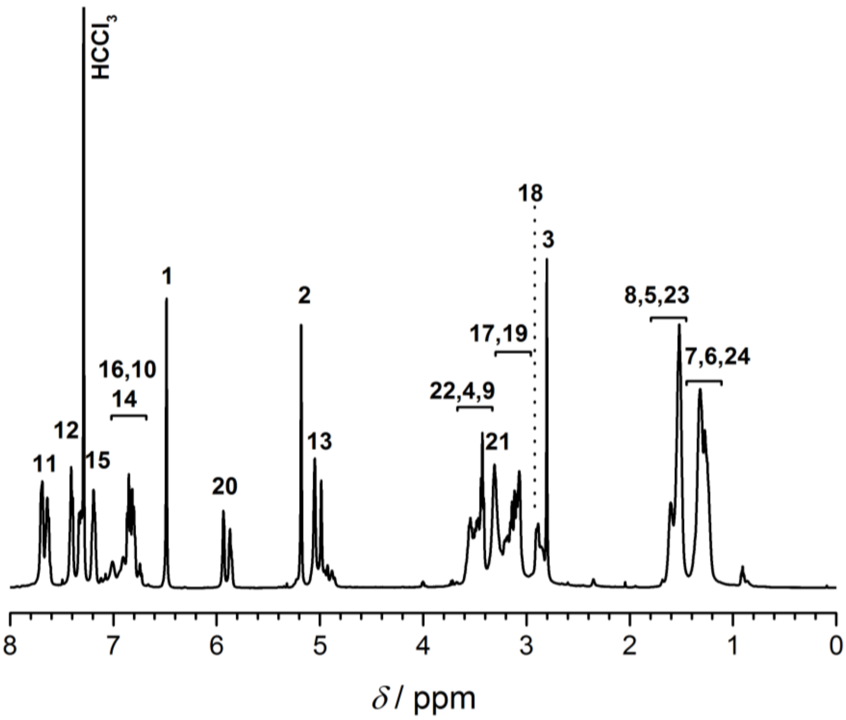

Supplementary Figure 46. ^{1}H NMR spectrum of **7b** ($CDCl_3$).

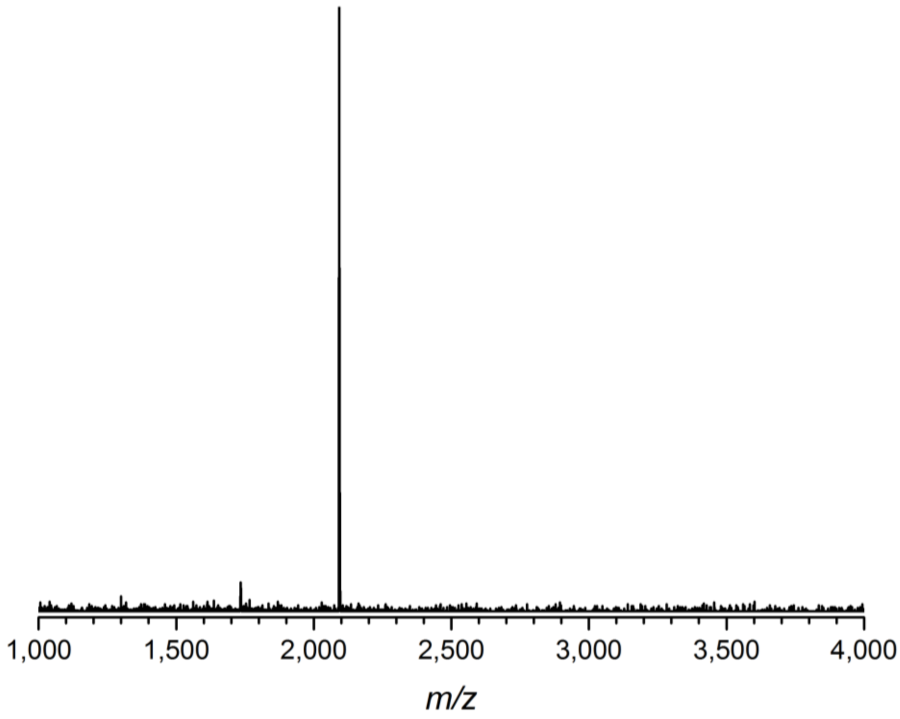

Supplementary Figure 47. MALDI–ToF overview spectrum of **7b**. All major peaks belong to the target molecule with different counter ions. All peak assignments can be found in Supplementary Table 8.

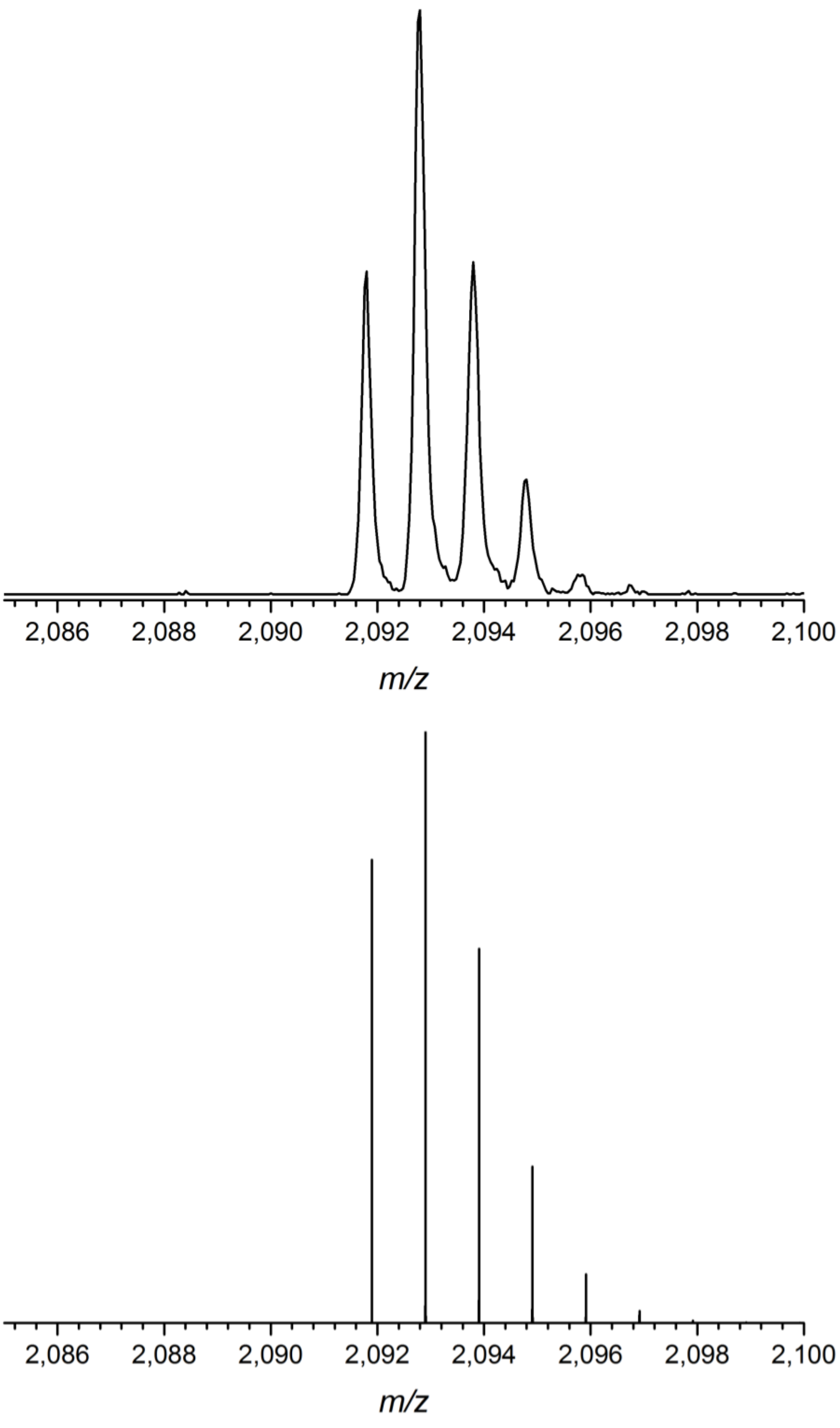

Supplementary Figure 48. MALDI–ToF experimental (top) and calculated (bottom) zoom spectra of **7b**.

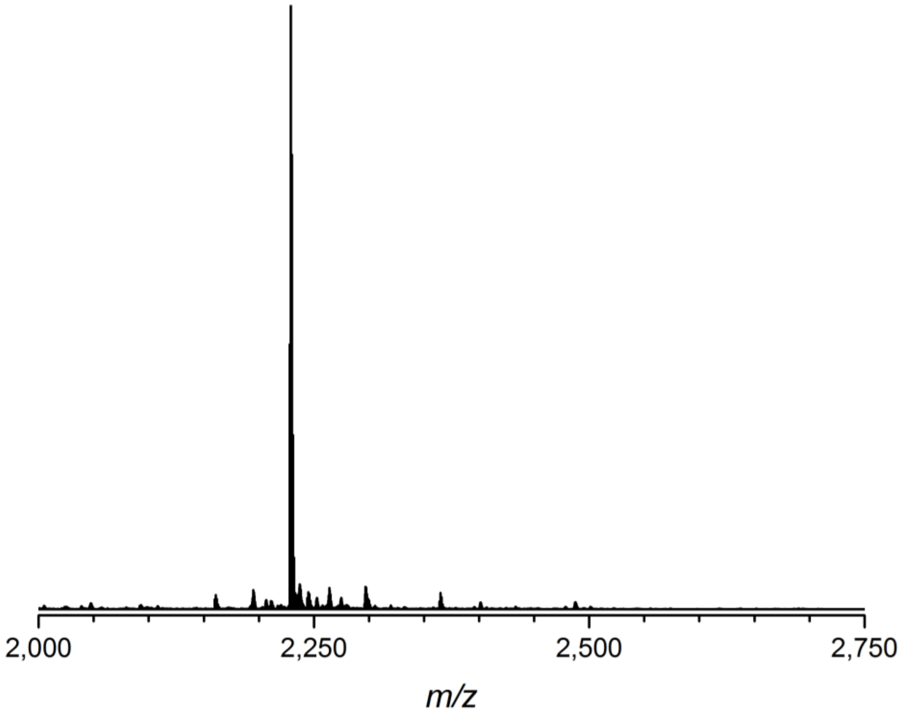

Supplementary Figure 49. ESI-MS overview spectrum of **7b**. All major peaks belong to the target molecule with different counter ions. All peak assignments can be found in Supplementary Table 8.

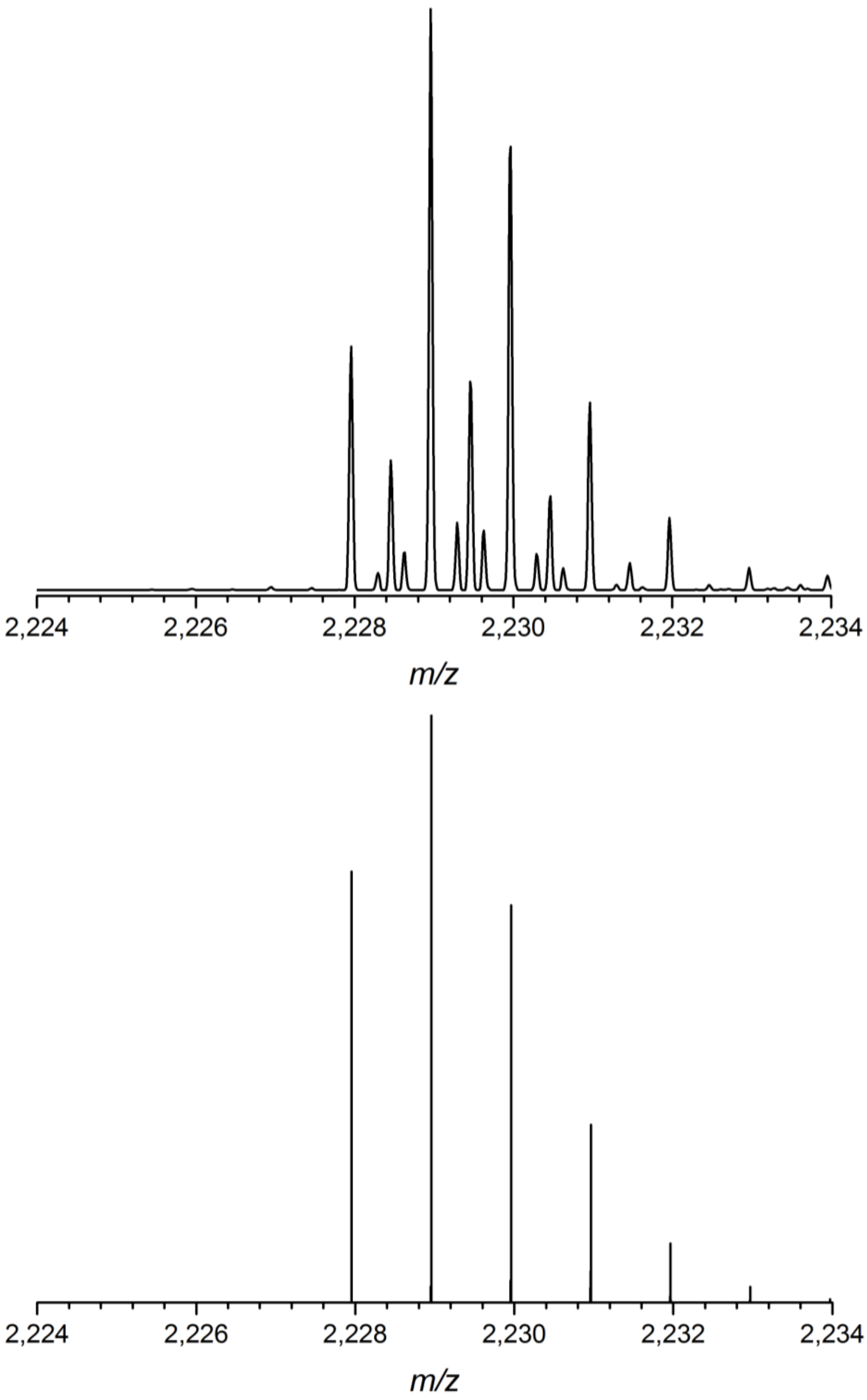

Supplementary Figure 50. ESI-MS experimental (top) and calculated (bottom) zoom spectra of **7b**. The exact mass of the measured sample is matching with the assigned species (see Supplementary Table 8), however there is likely an additional signal from a double charged cluster of two molecules.

Characterization of 7c $(M_1)_3$-X-$(M_1)_3$

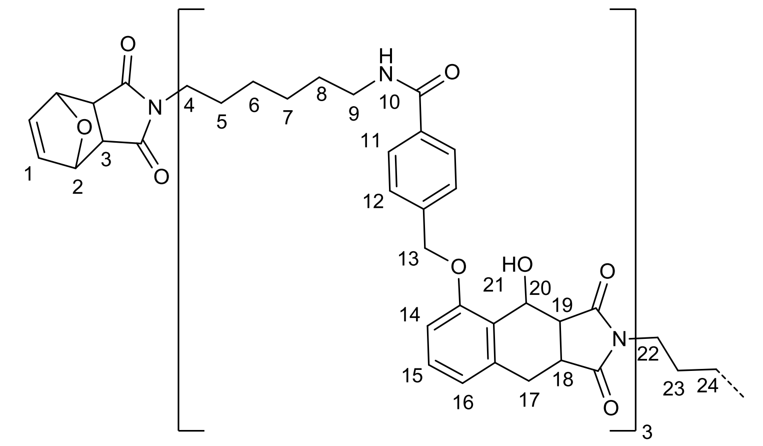

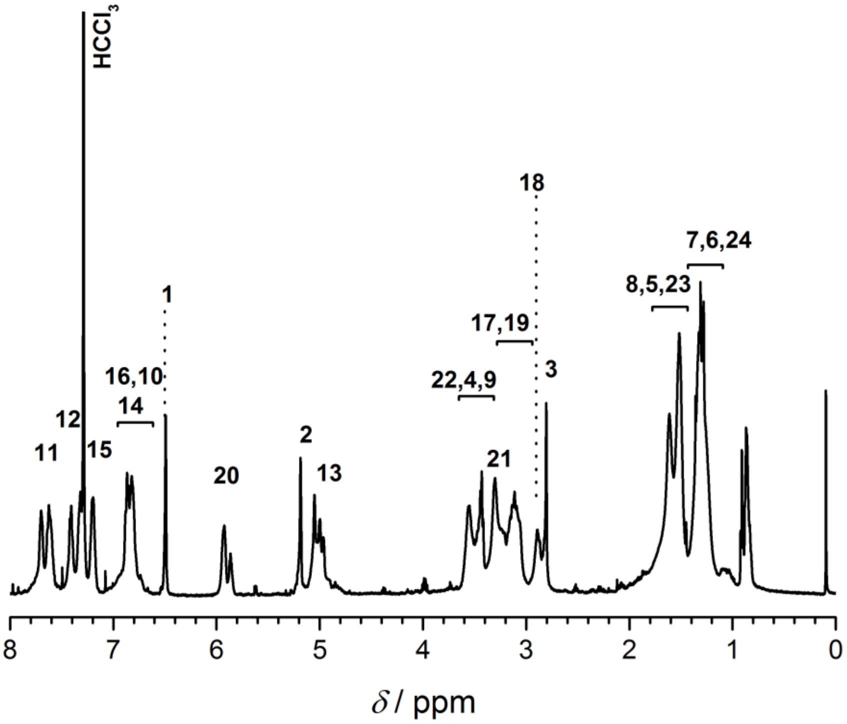

Supplementary Figure 51. ^{1}H NMR spectrum of **7c** ($CDCl_3$).

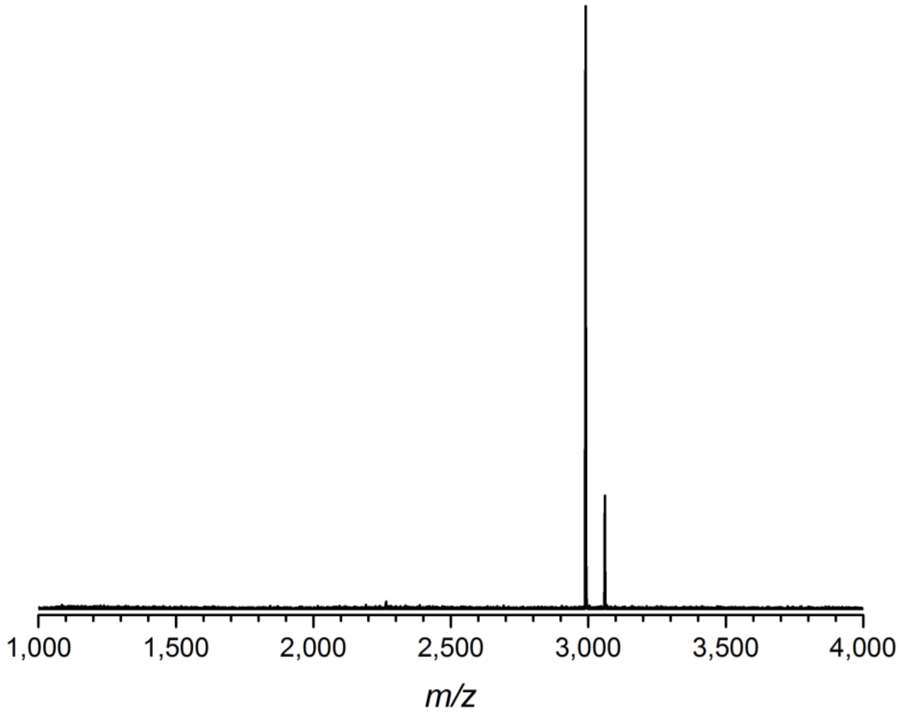

Supplementary Figure 52. MALDI–ToF overview spectrum of **7c**. All major peaks belong to the target molecule with different counter ions. All peak assignments can be found in Supplementary Table 9.

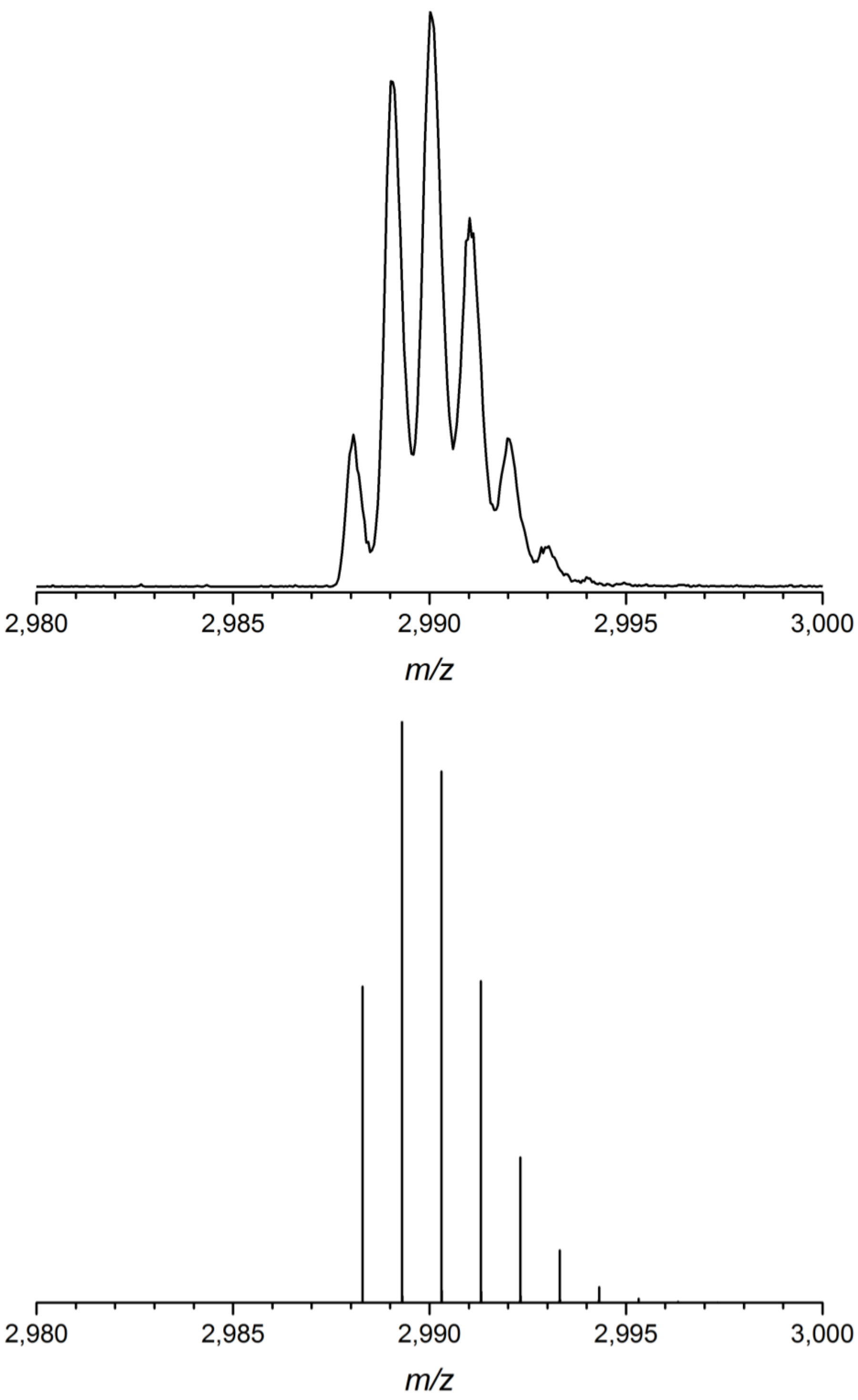

Supplementary Figure 53. MALDI–ToF experimental (top) and calculated (bottom) zoom spectra of **7c**.

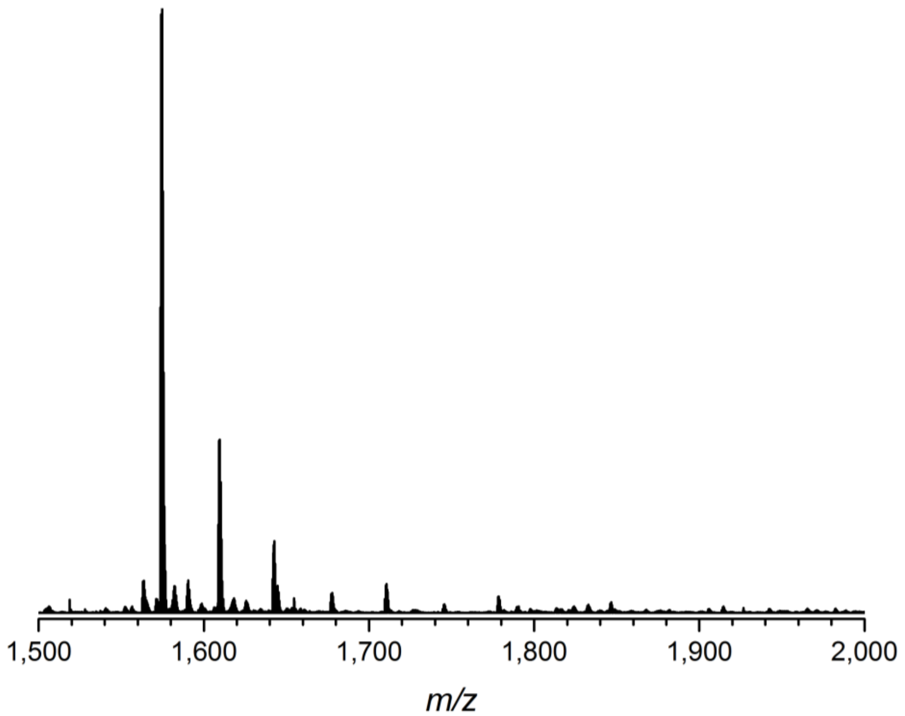

Supplementary Figure 54. ESI-MS overview spectrum of **7c**. All major peaks belong to the target molecule with different counter ions. All peak assignments can be found in Supplementary Table 9.

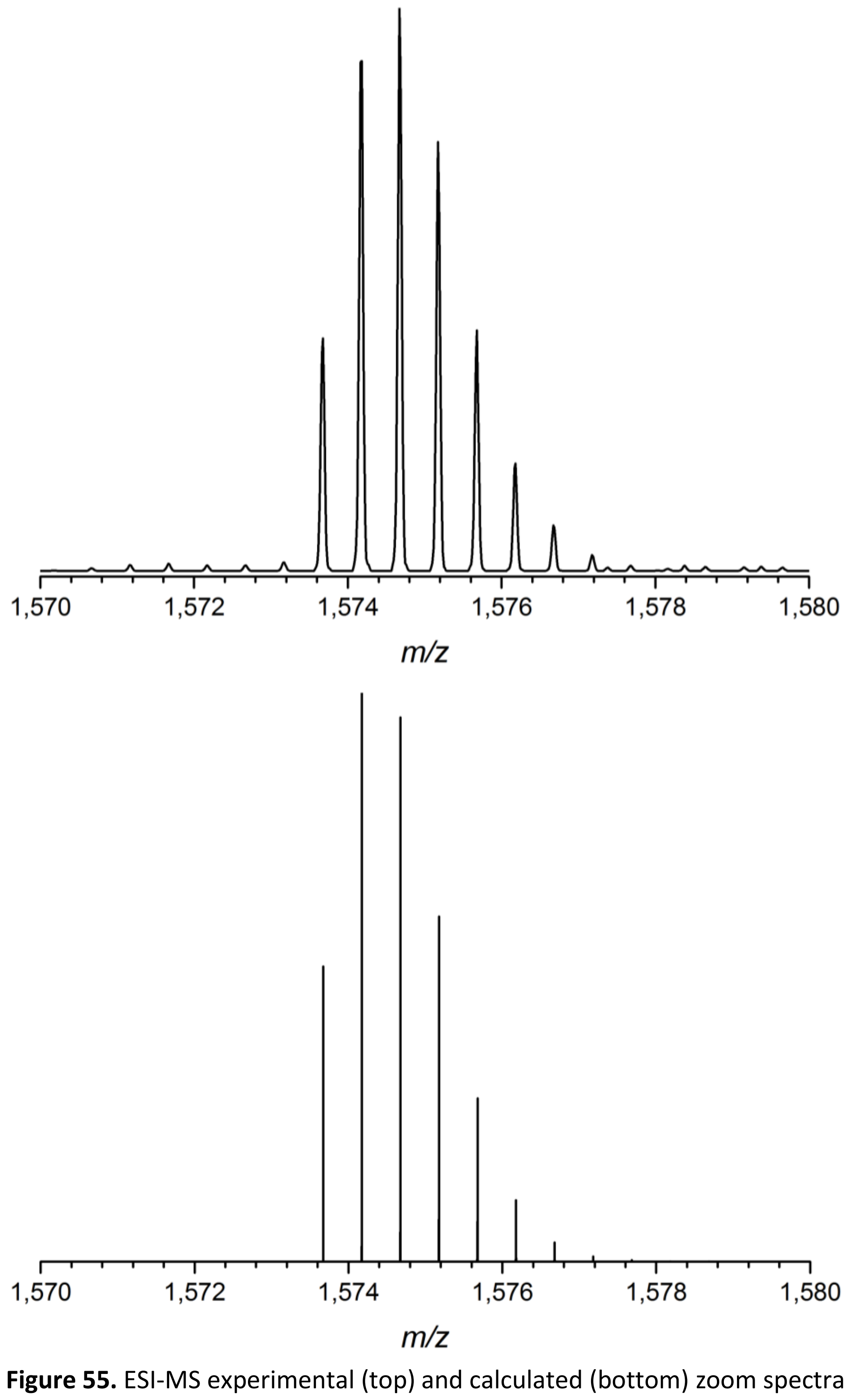

Supplementary Figure 55. ESI-MS experimental (top) and calculated (bottom) zoom spectra of **7c**.

Characterization of 7d $(M_1)_2$

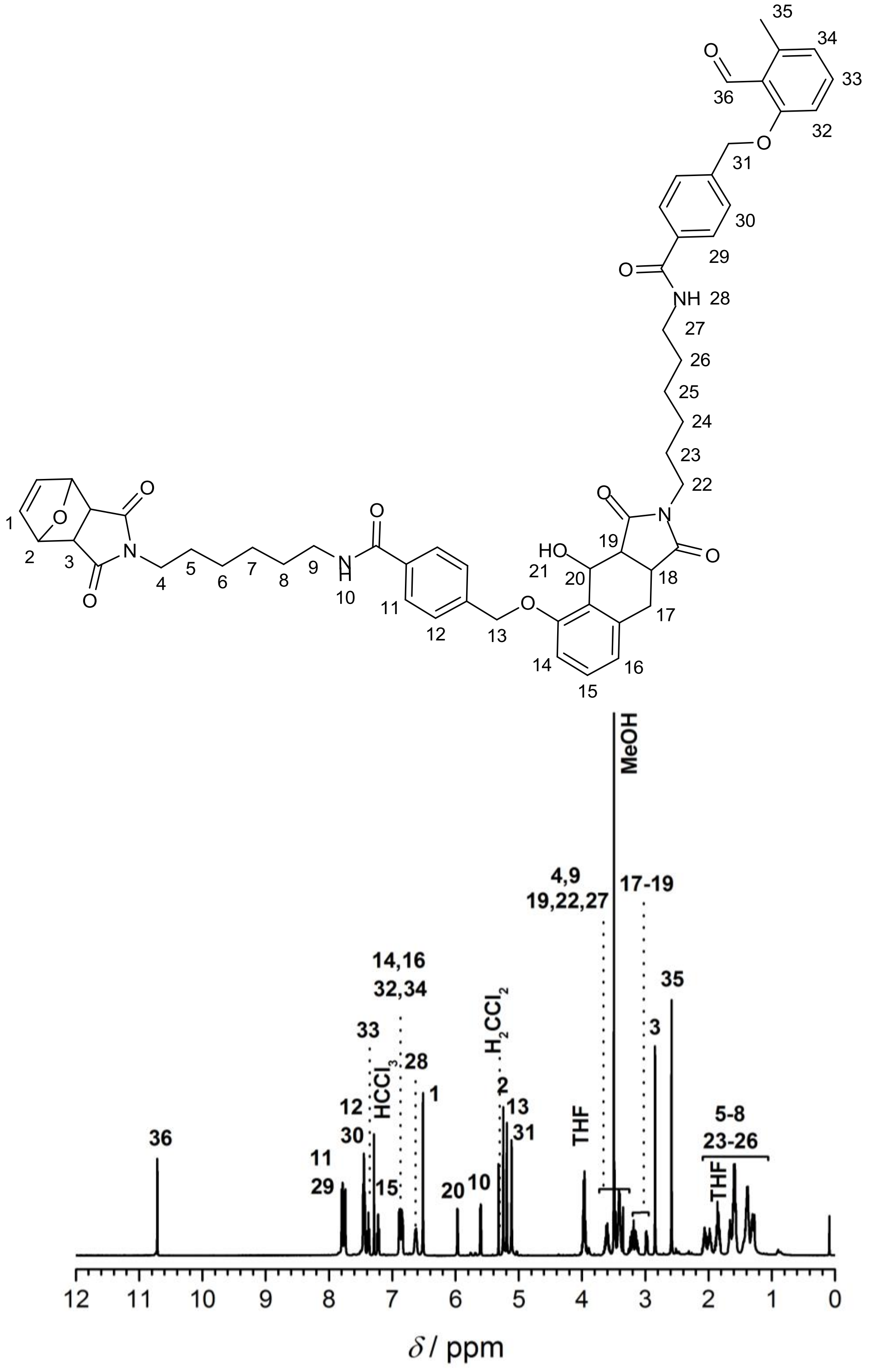

Supplementary Figure 56. ^{1}H NMR spectrum of **7d** ($CDCl_3$).

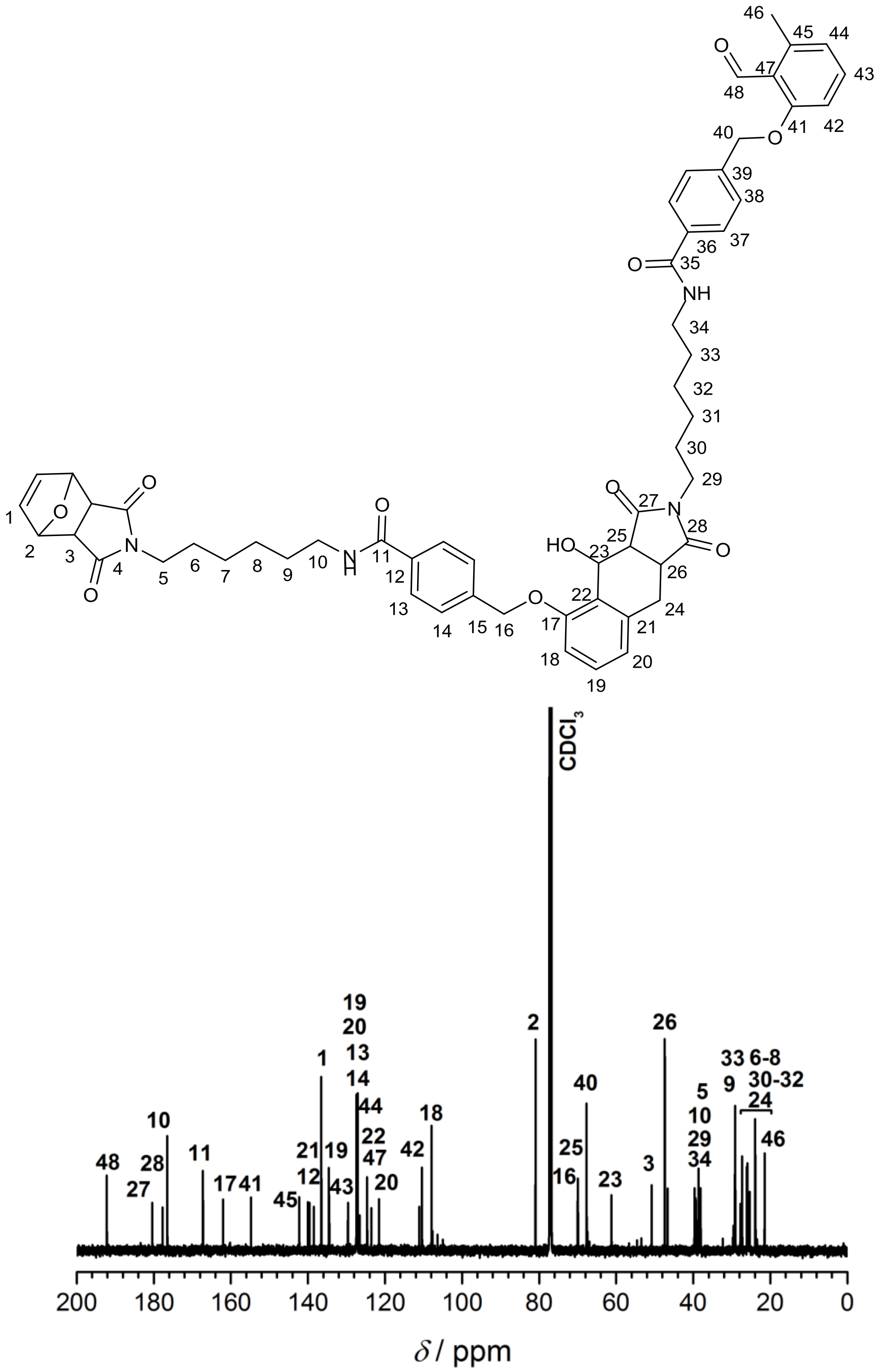

Supplementary Figure 57. ^{13}C NMR spectrum of **7d** ($CDCl_3$).

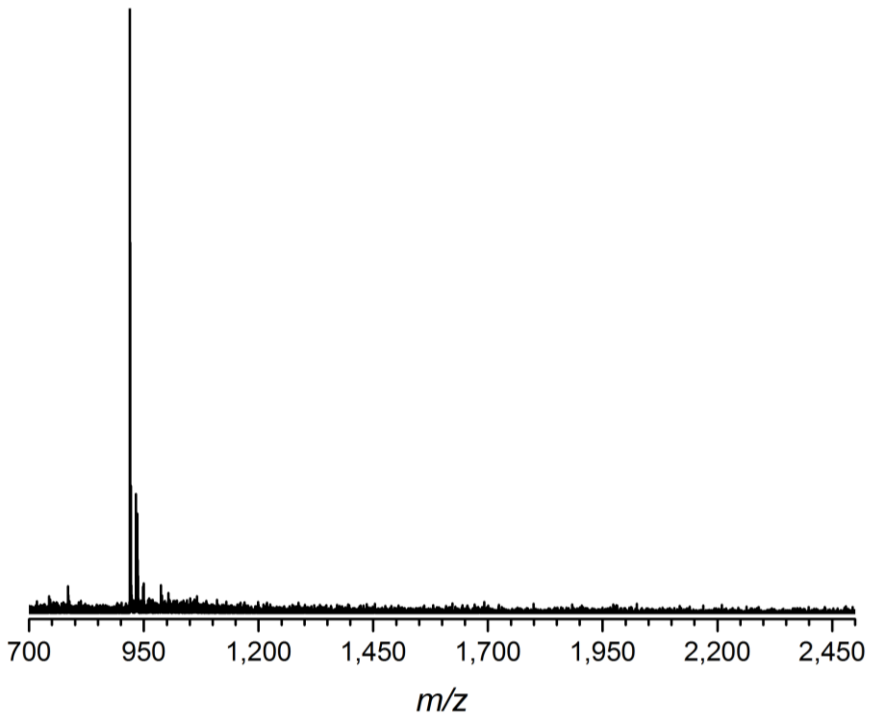

Supplementary Figure 58. MALDI–ToF overview spectrum of **7d**. All major peaks belong to the target molecule with different counter ions. All peak assignments can be found in Supplementary Table 10.

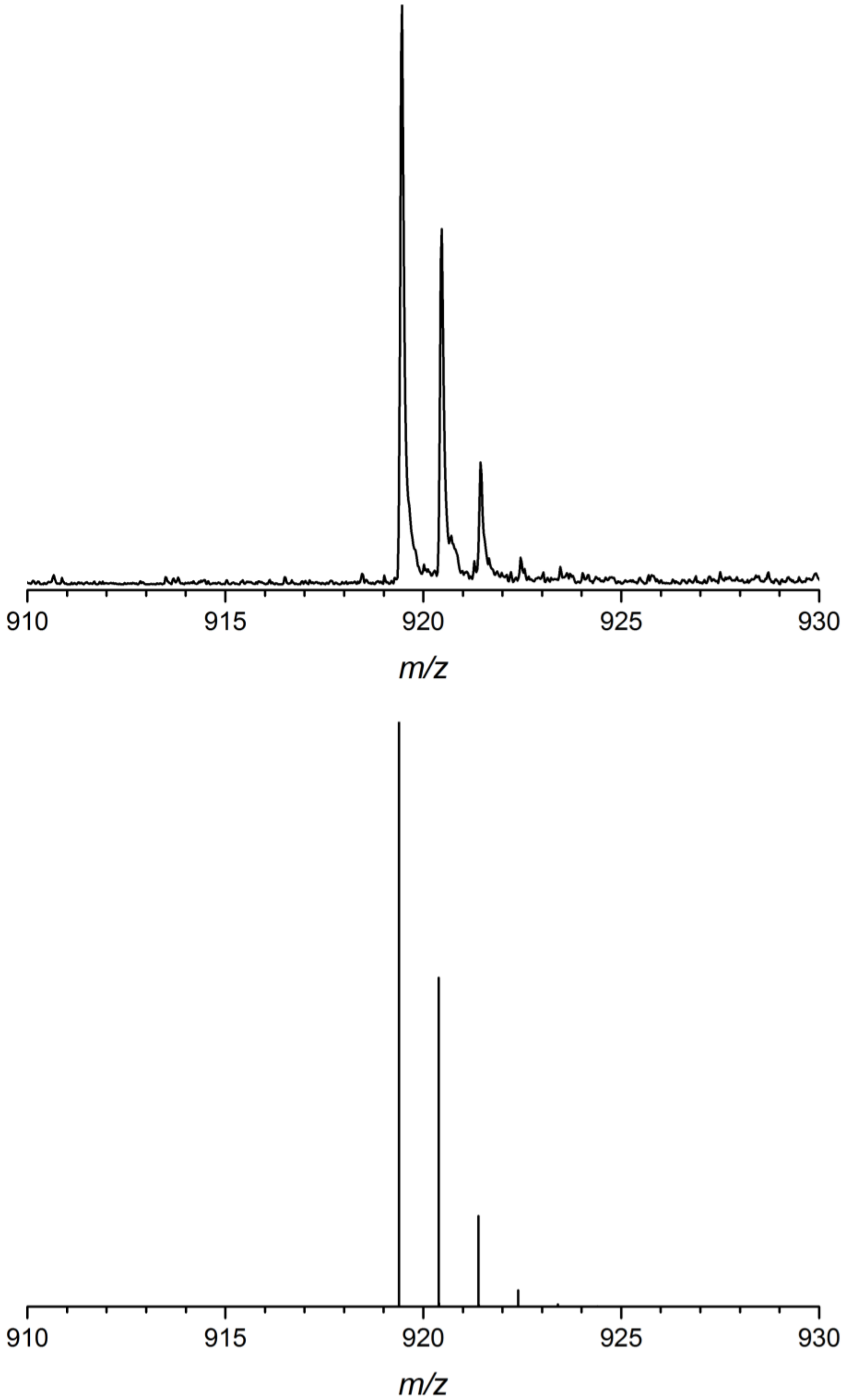

Supplementary Figure 59. MALDI–ToF experimental (top) and calculated (bottom) zoom spectra of **7d**.

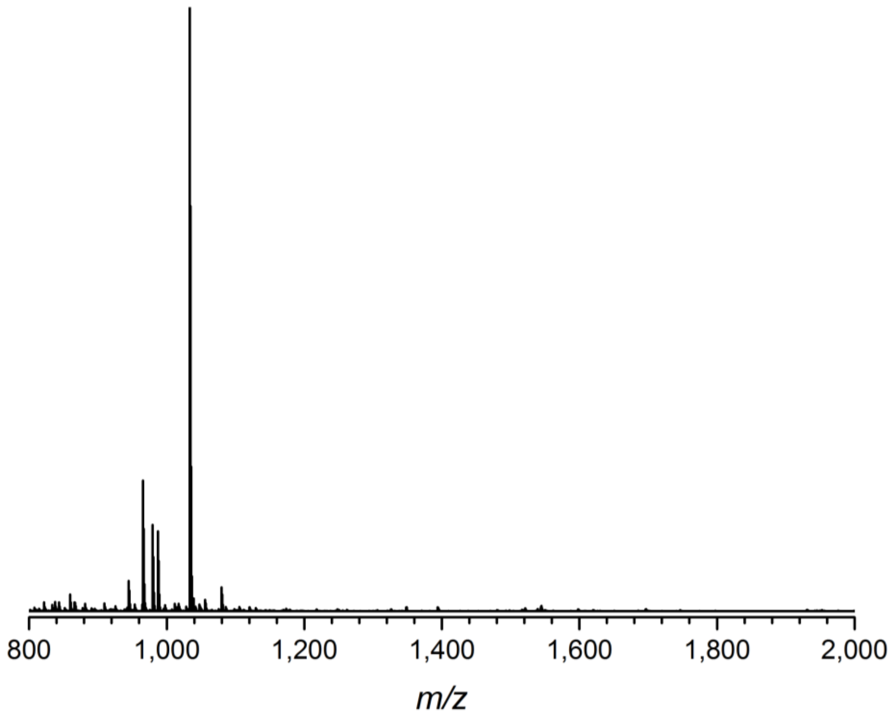

Supplementary Figure 60. ESI-MS overview spectrum of **7d**. All major peaks belong to the target molecule with different counter ions. All peak assignments can be found in Supplementary Table 10.

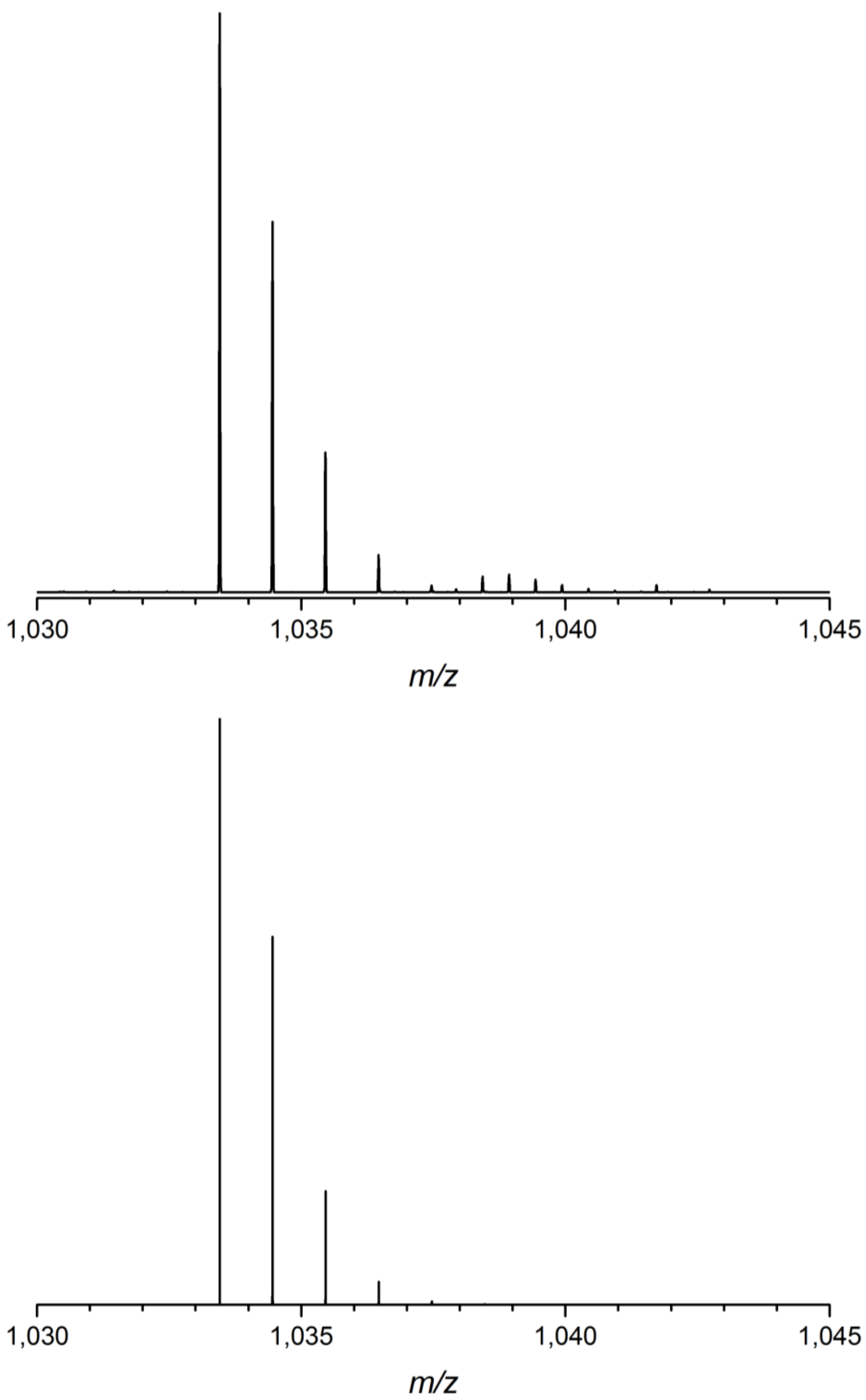

Supplementary Figure 61. ESI-MS experimental (top) and calculated (bottom) zoom spectra of **7d**.

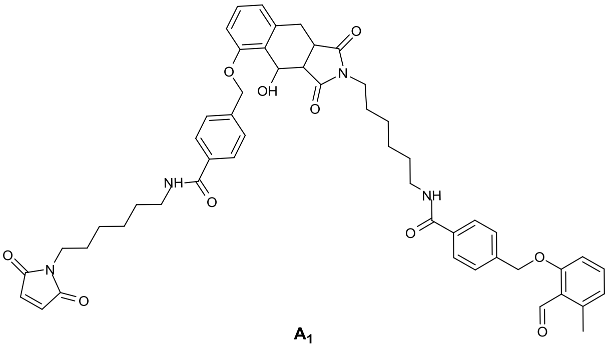

$\mathbf{A_1}$

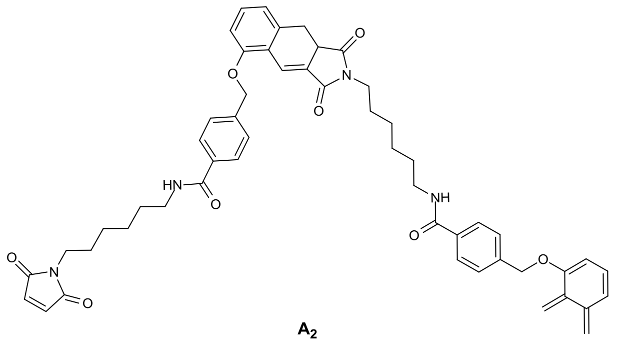

$\mathbf{A_2}$

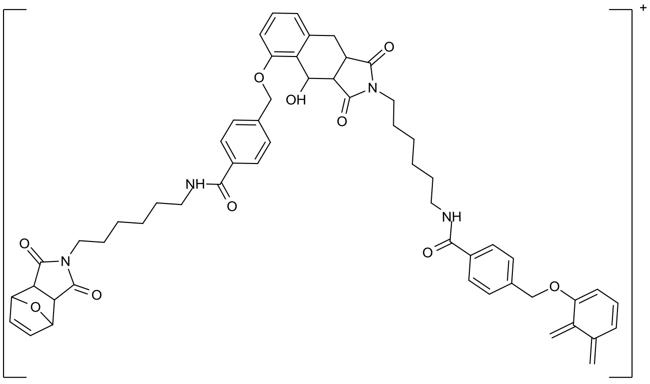

$\mathbf{A_3^+}$

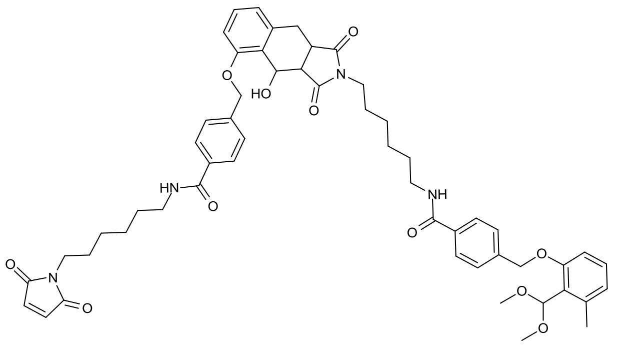

$\mathbf{A_4}$

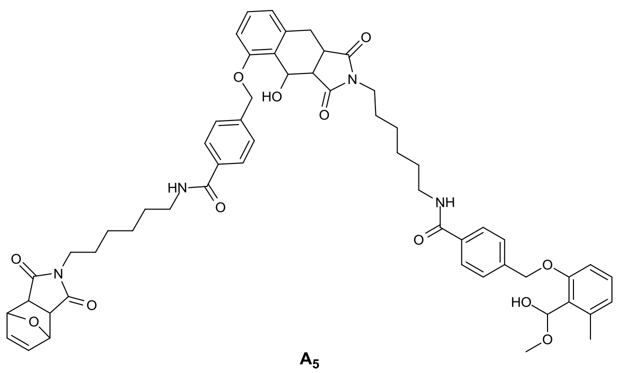

$\mathbf{A_5}$

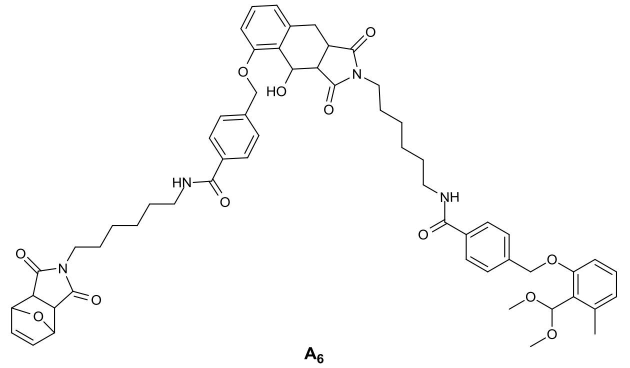

$\mathbf{A_6}$

Supplementary Figure 62. Collation of molecules identified in Supplementary Table 10 ($\mathbf{A_1}$-$\mathbf{A_6}$).

Characterization of 7 $(M_1)_5$-X-$(M_1)_5$

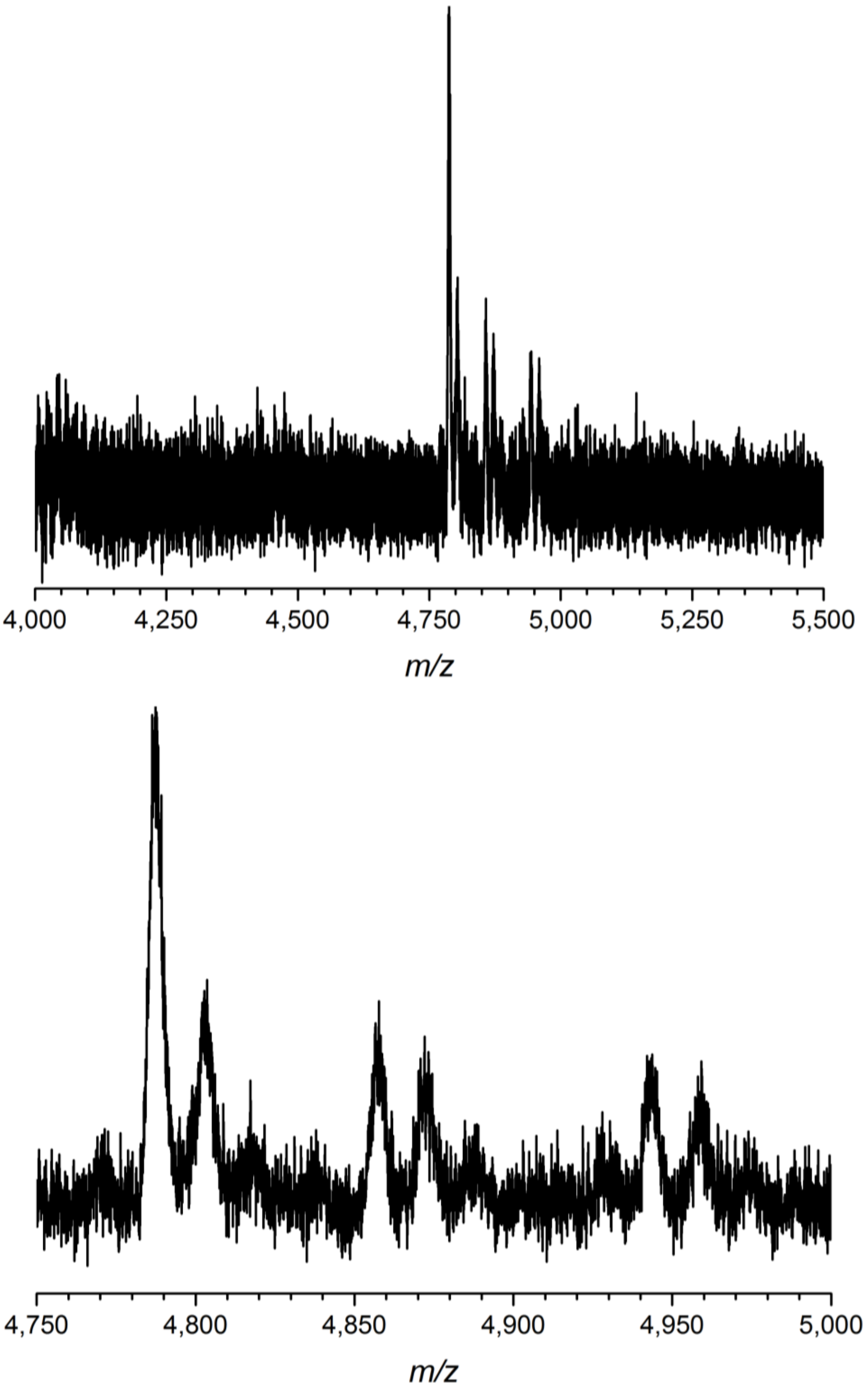

Supplementary Figure 63. MALDI–ToF overview (top) and detailed (bottom) spectra of **7**. All major peaks belong to the target molecule with different counter ions. All peak assignments can be found in Supplementary Table 11.

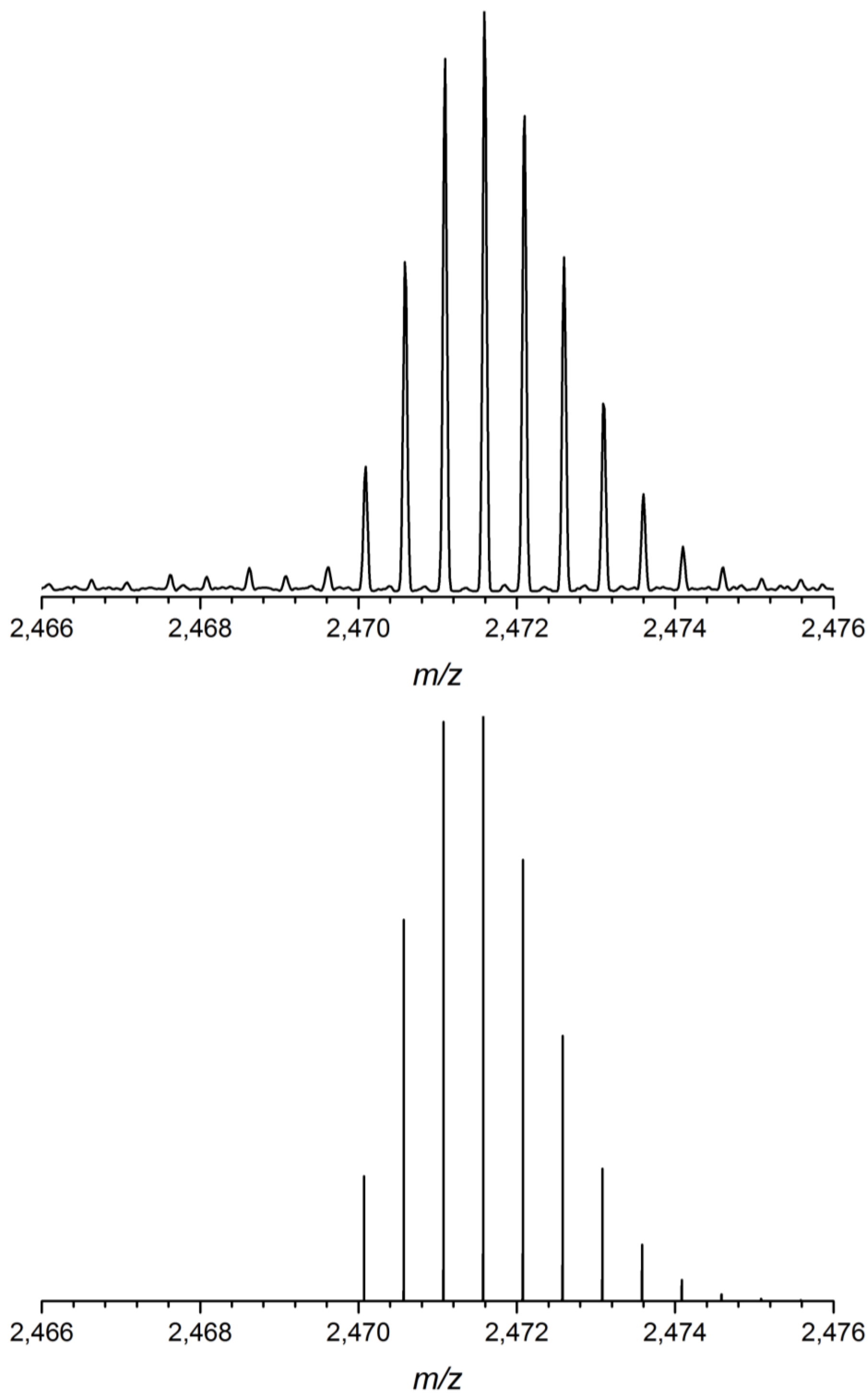

Supplementary Figure 64. ESI-MS experimental (top) and calculated (bottom) zoom spectra of **7**.

Characterization of symmetric Copolymer 8 (($(M_1)_2(M_2)(M_1)_2$-X-$(M_1)_2(M_2)(M_1)_2$) and precursor 8a (($(M_2)(M_1)_2$-X-$(M_1)_2(M_2)$))

Characterization of 8a $(M_2)(M_1)_2$-X-$(M_1)_2(M_2)$

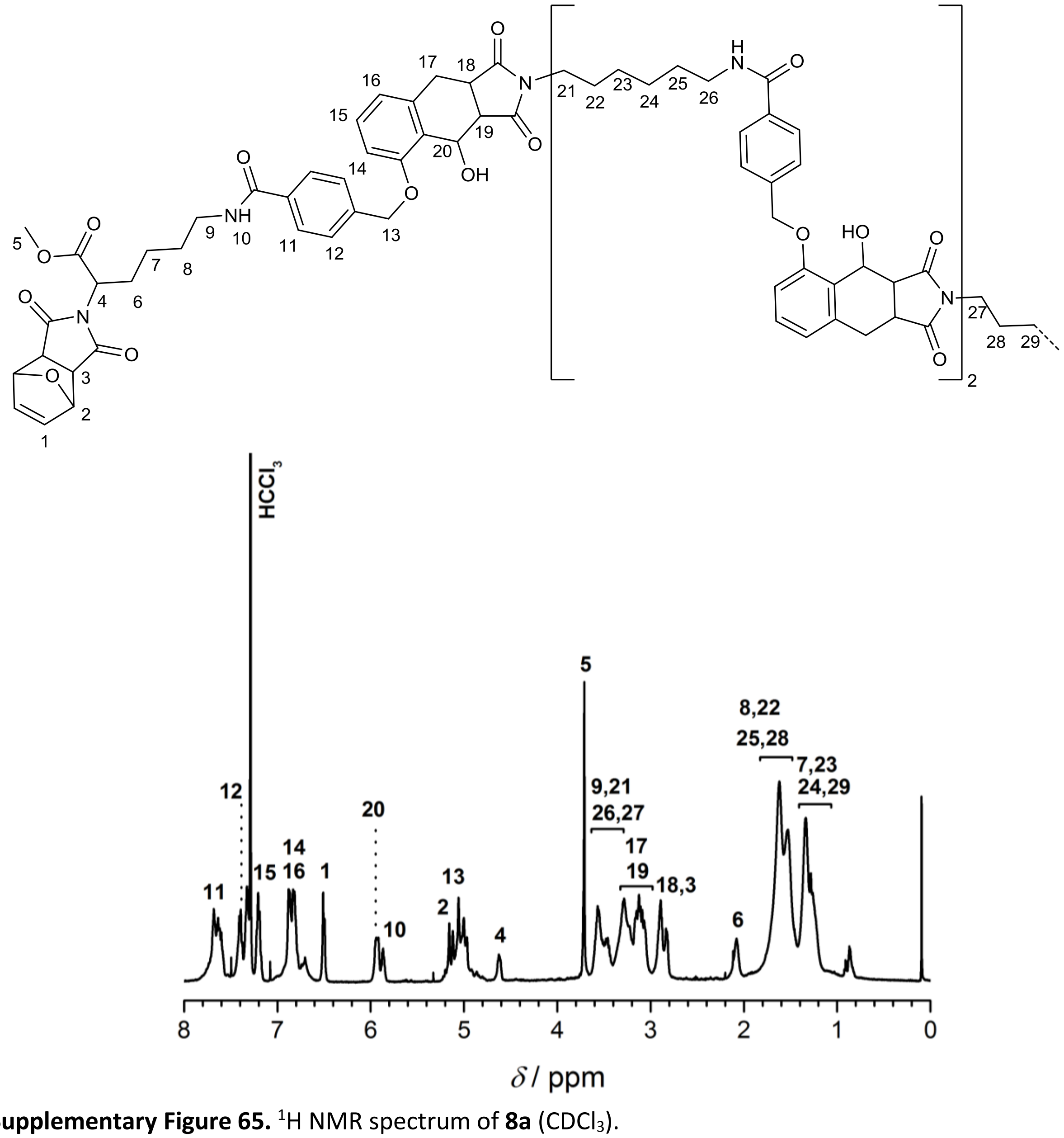

Supplementary Figure 65. ^{1}H NMR spectrum of **8a** ($CDCl_3$).

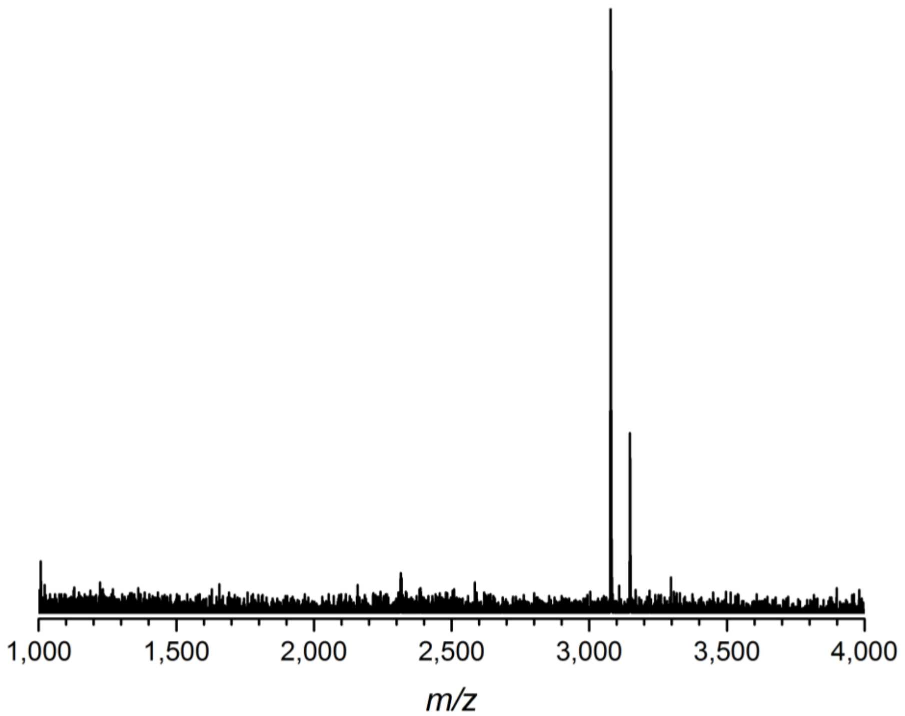

Supplementary Figure 66. MALDI–ToF overview spectrum of **8a**. All major peaks belong to the target molecule with different counter ions. All peak assignments can be found in Supplementary Table 12.

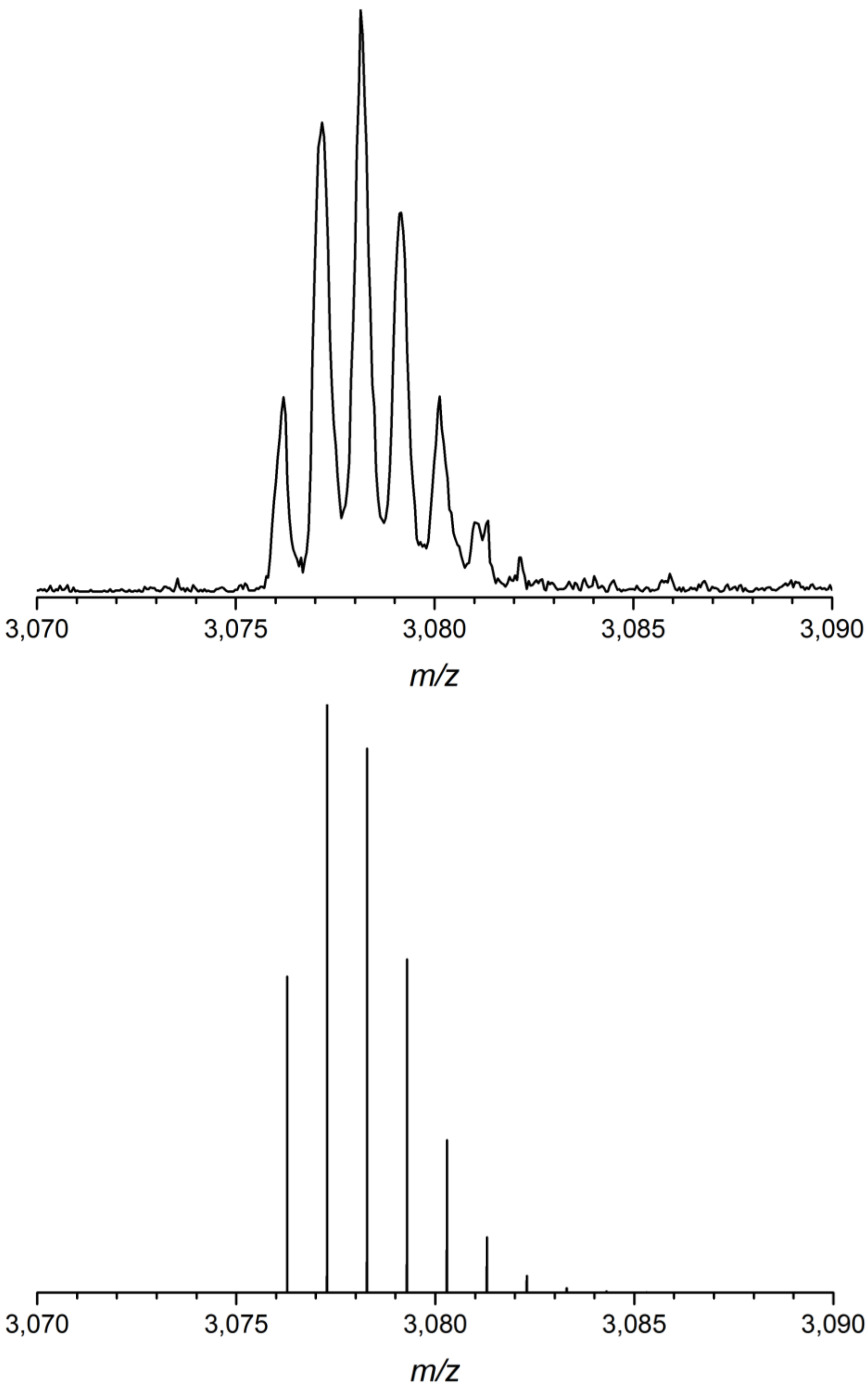

Supplementary Figure 67. MALDI–ToF experimental (top) and calculated (bottom) zoom spectra of **8a**.

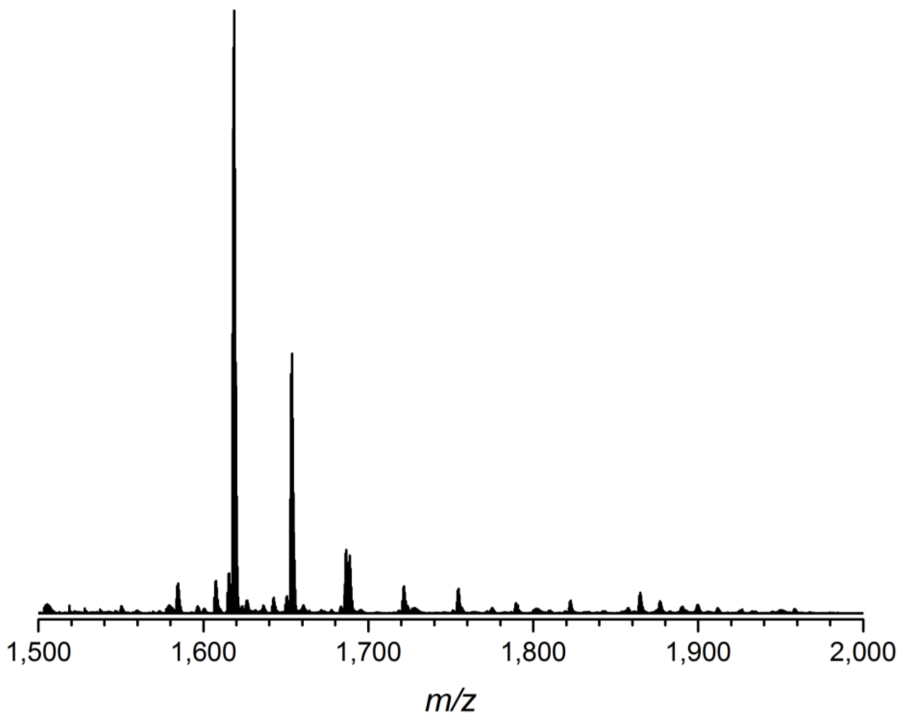

Supplementary Figure 68. ESI-MS overview spectrum of **8a**. All major peaks belong to the target molecule with different counter ions. All peak assignments can be found in Supplementary Table 12.

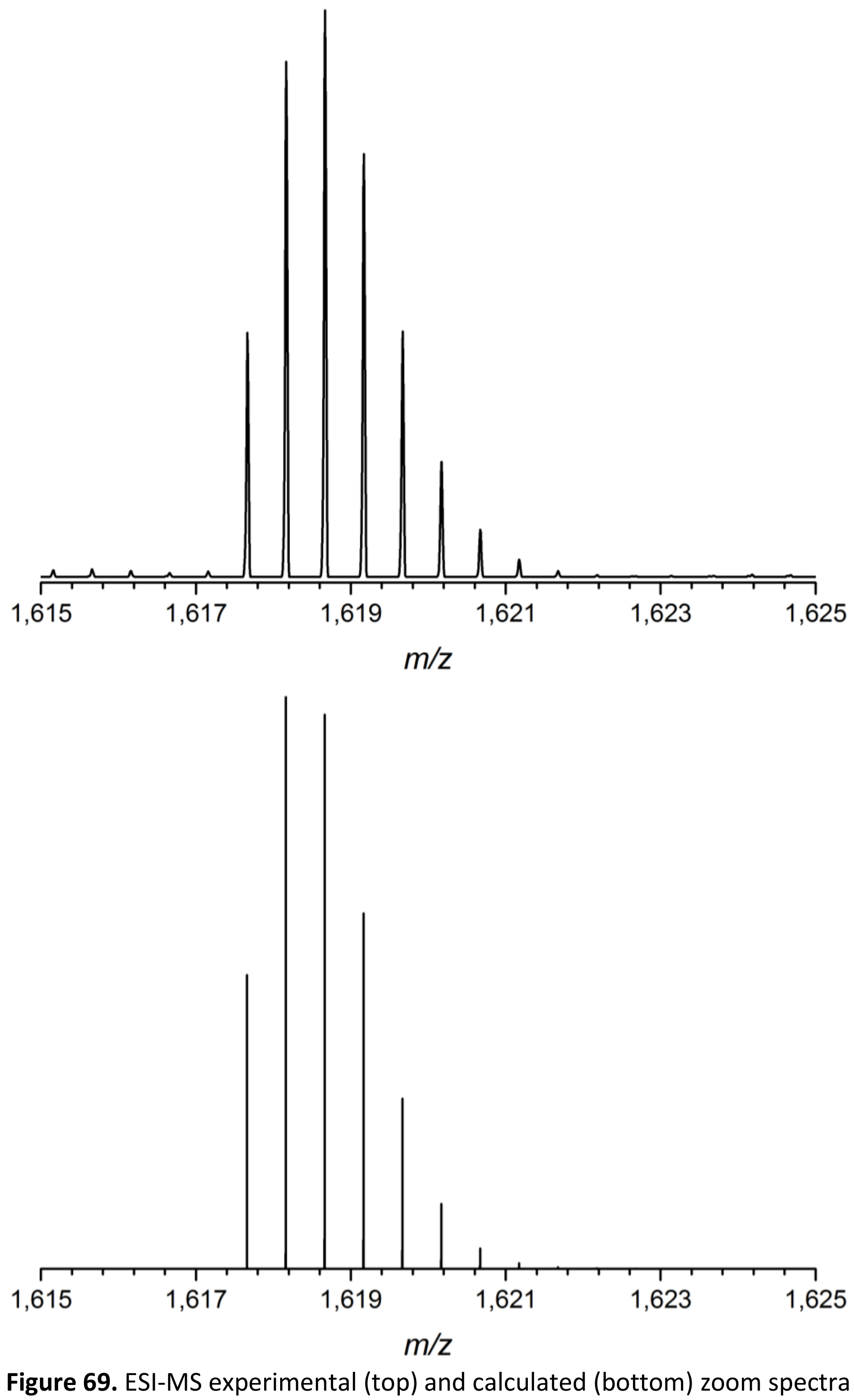

Supplementary Figure 69. ESI-MS experimental (top) and calculated (bottom) zoom spectra of **8a**.

Characterization of 8 $(M_1)_2(M_2)(M_1)_2$-X-$(M_1)_2(M_2)(M_1)_2$

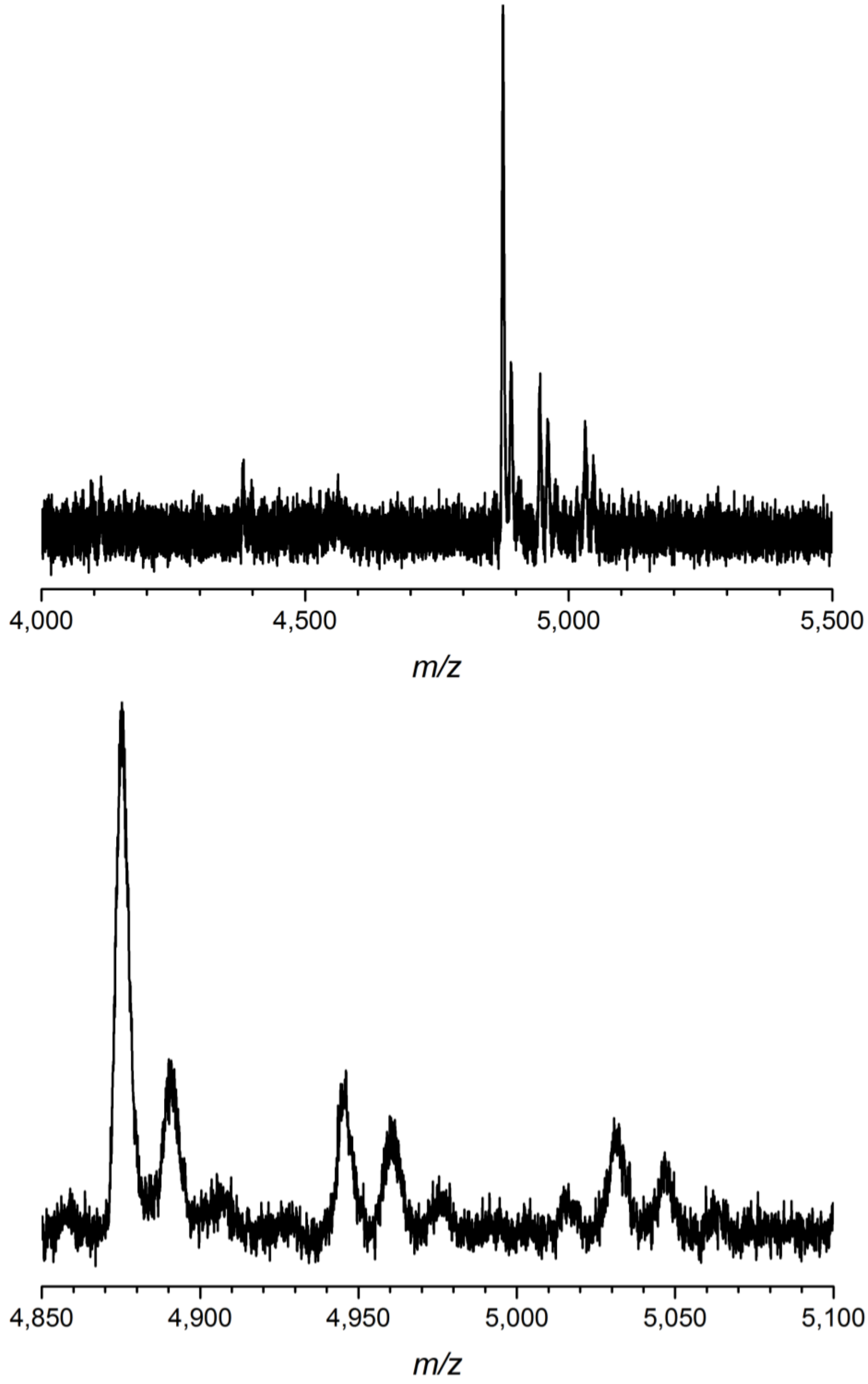

Supplementary Figure 70. MALDI–ToF overview (top) and zoom (bottom) spectra of **8**. All major peaks belong to the target molecule with different counter ions. All peak assignments can be found in Supplementary Table 13.

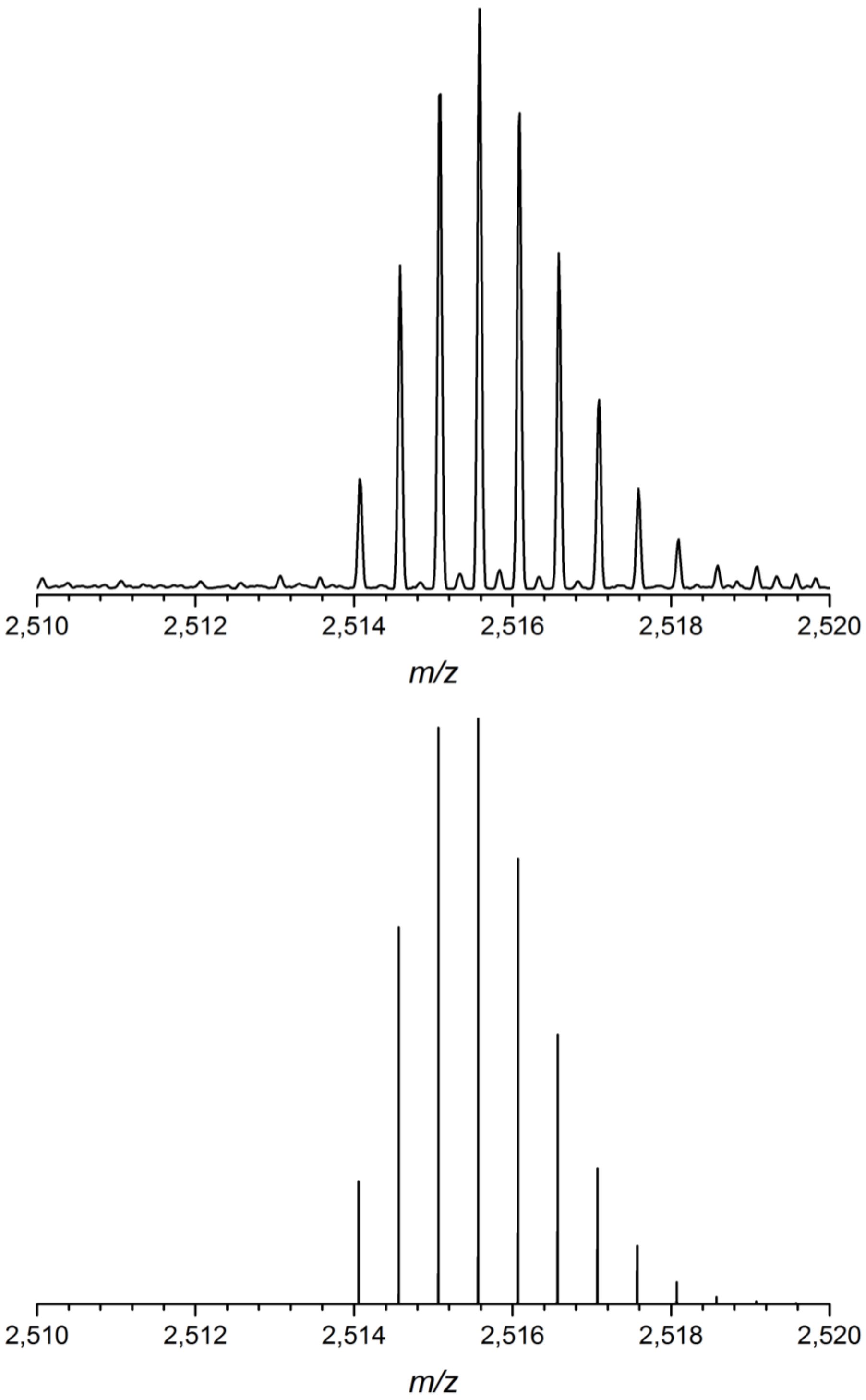

Supplementary Figure 71. ESI-MS experimental (top) and calculated (bottom) zoom spectra of **8**.

Characterization of symmetric Copolymer 9 ((M_1)(M_2M_1)$_2$-X-(M_1M_2)$_2$(M_1)) and precursors 9a-c (9a: (M_2M_1)-X-(M_1M_2), 9b: ($M_1M_2M_1$)-X-($M_1M_2M_1$), 9c: (M_1M_2))

Characterization of 9a (M_2M_1)-X-(M_1M_2)

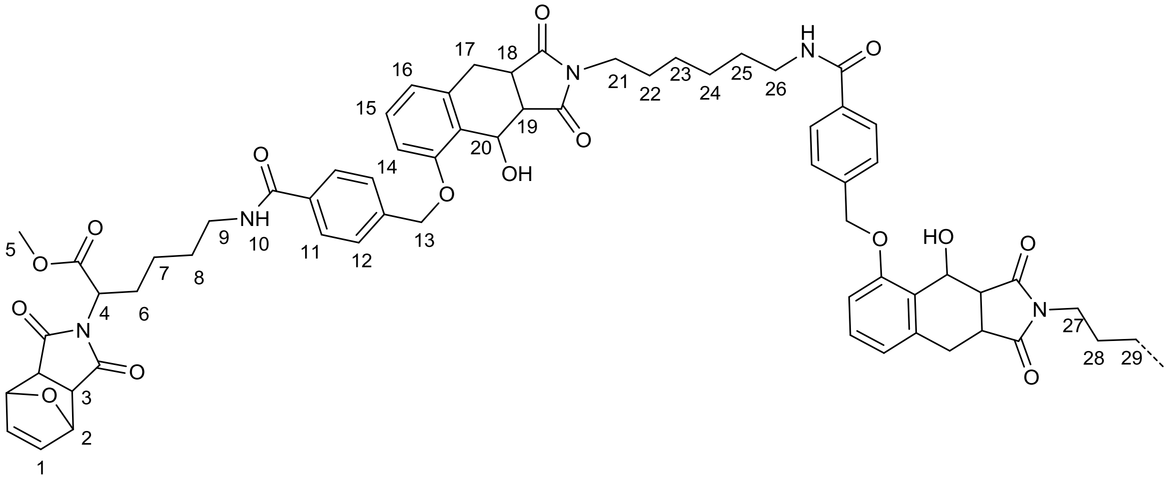

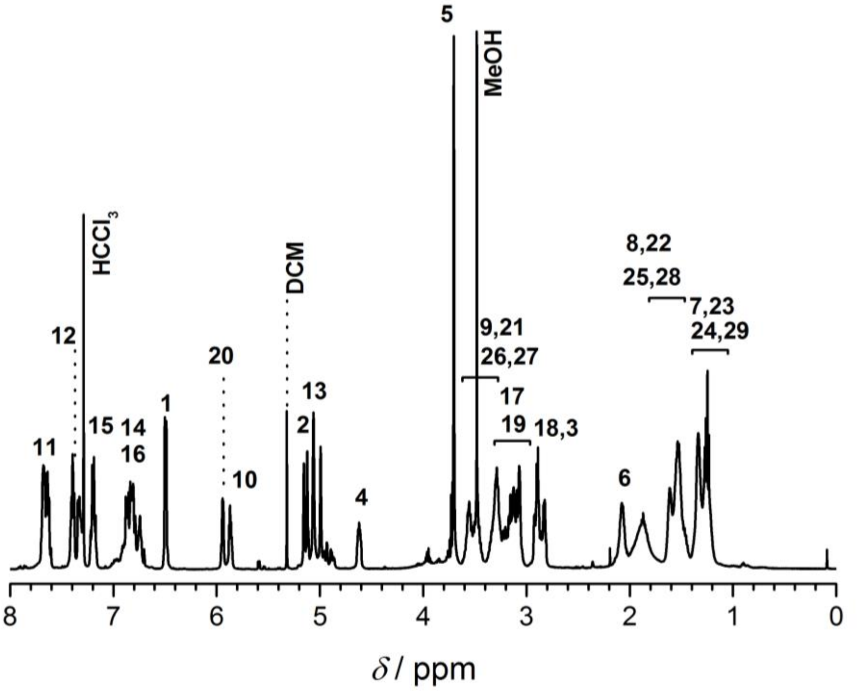

Supplementary Figure 72. ^{1}H NMR spectrum of **9a** ($CDCl_3$).

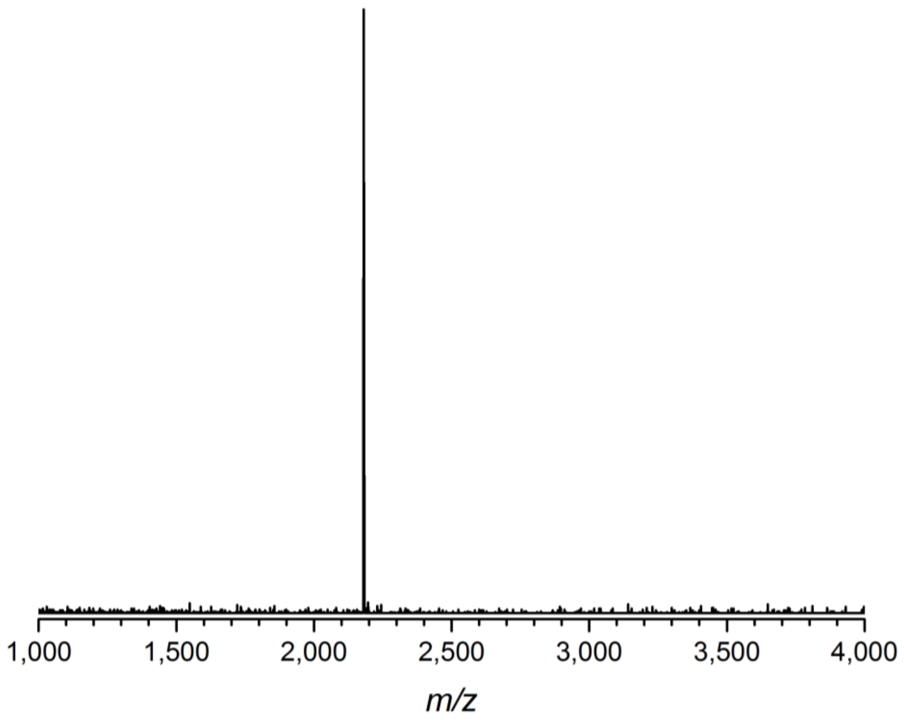

Supplementary Figure 73. MALDI–ToF overview spectrum of **9a**. All peaks belong to the target molecule with a different counter ion. All peak assignments can be found in Supplementary Table 14.

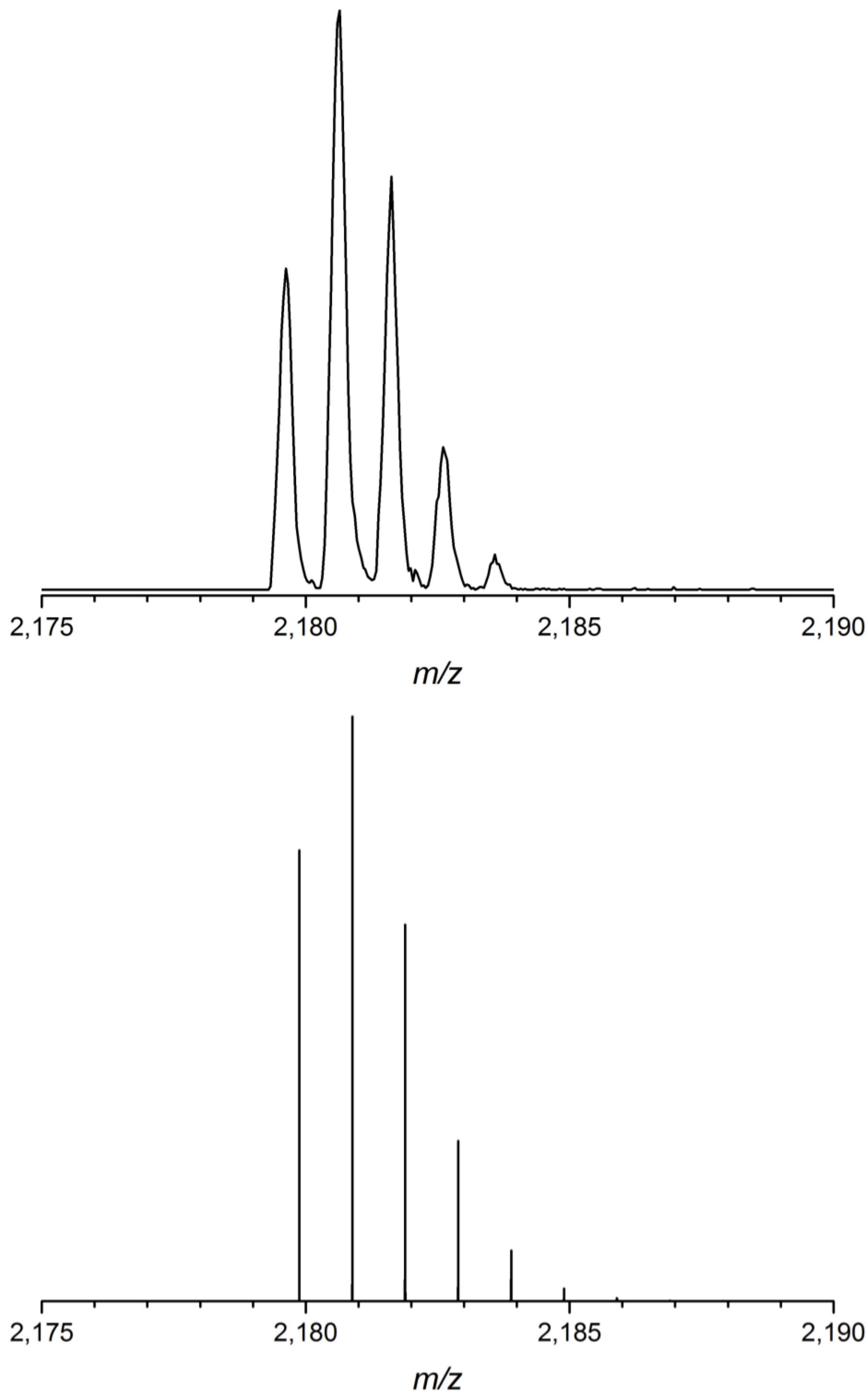

Supplementary Figure 74. MALDI–ToF experimental (top) and calculated (bottom) zoom spectra of **9a**.

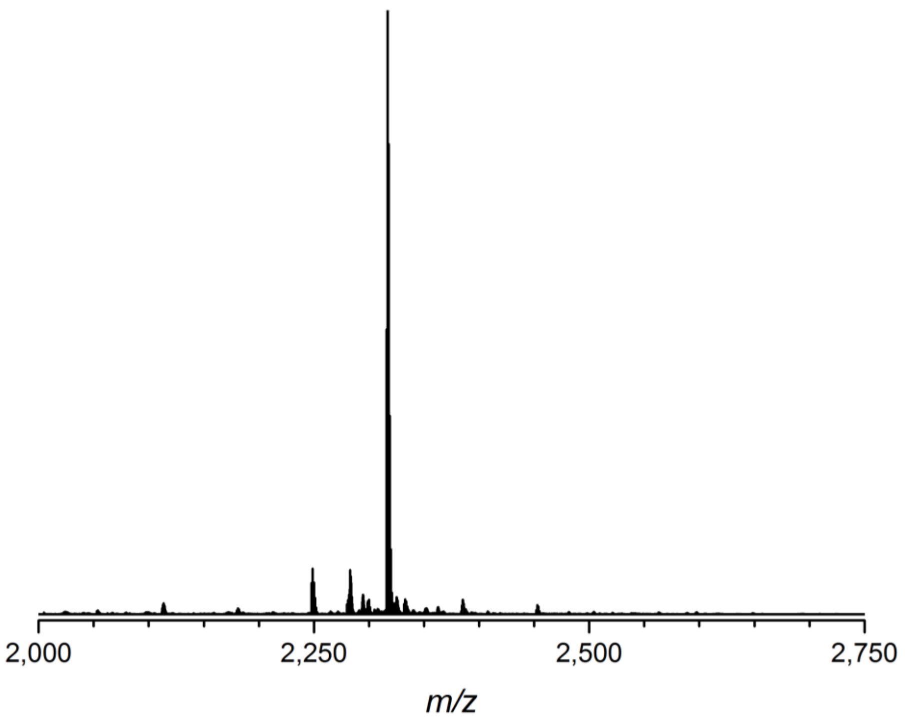

Supplementary Figure 75. ESI-MS overview spectrum of **9a**. All peaks belong to the target molecule with a different counter ion. All peak assignments can be found in Supplementary Table 14.

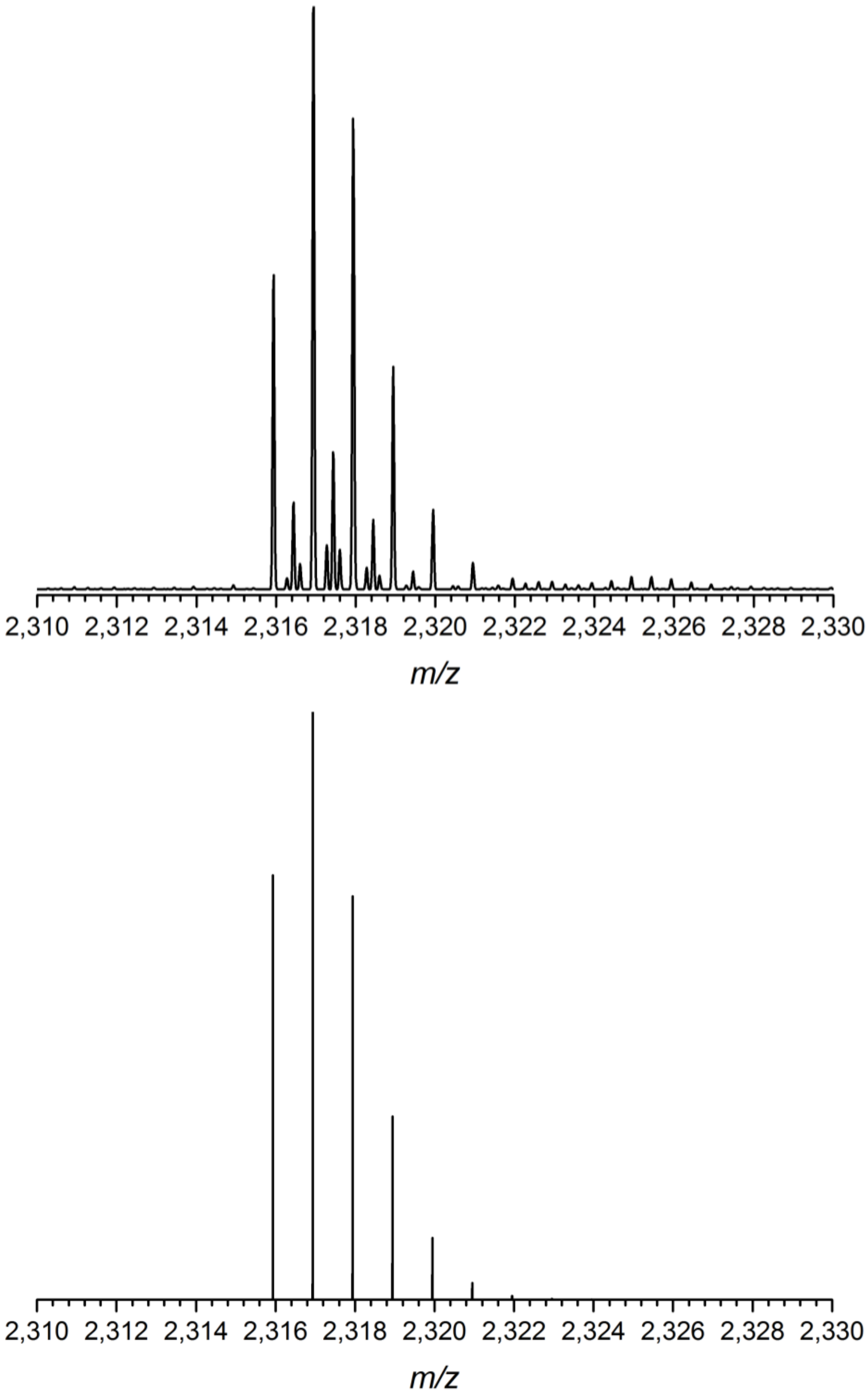

Supplementary Figure 76. ESI-MS experimental (top) and calculated (bottom) zoom spectra of **9a**. The exact mass of the measured sample is matching with the assigned species (see Supplementary Table 14), however there is likely an additional signal from a double charged cluster of two molecules.

Characterization of 9b ($M_1M_2M_1$)-X-($M_1M_2M_1$)

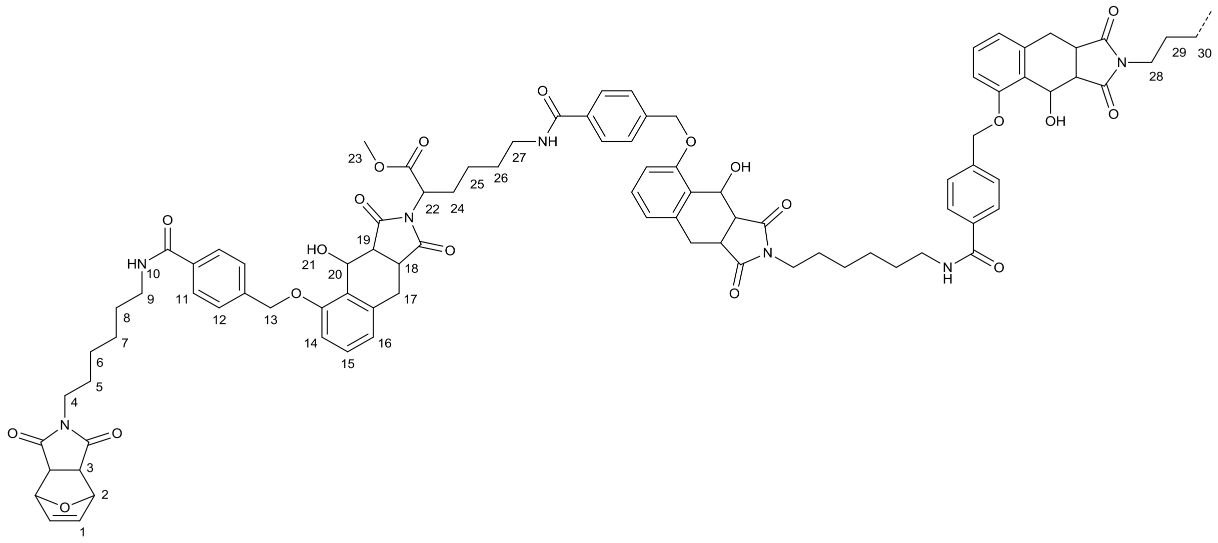

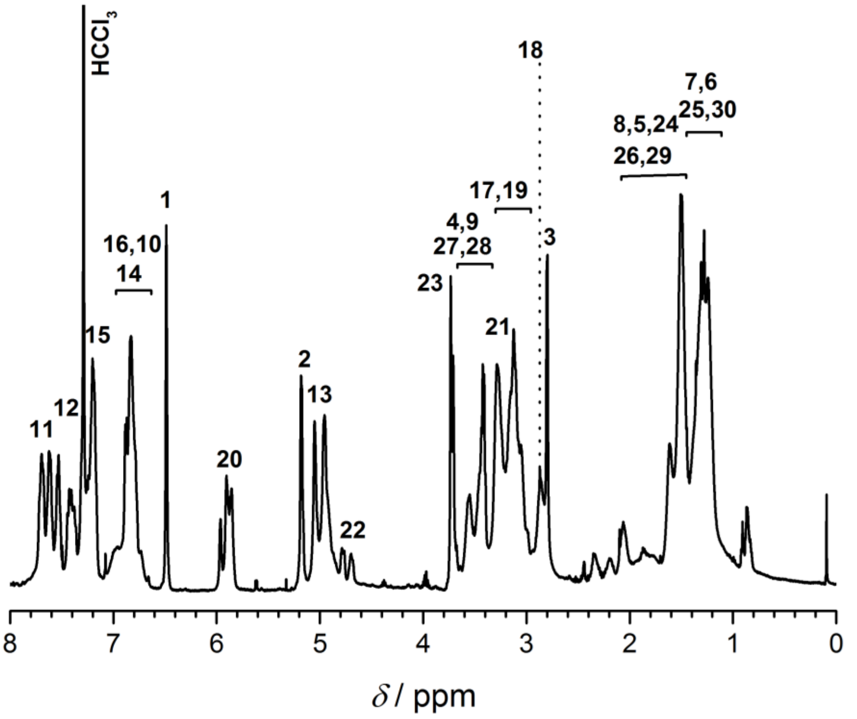

Supplementary Figure 77. ^1H NMR spectrum of **9b** ($CDCl_3$).

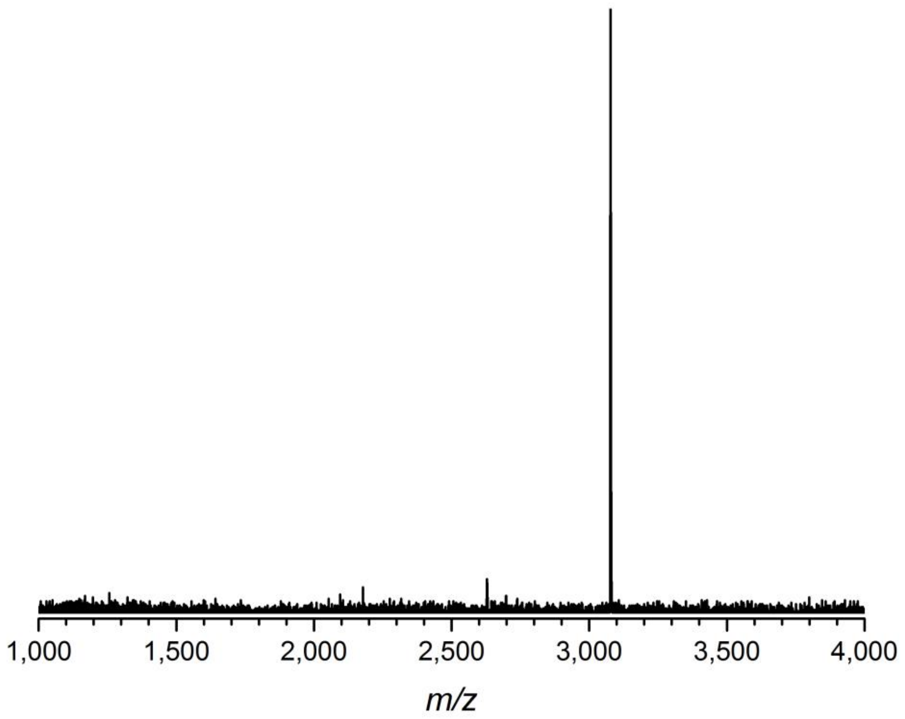

Supplementary Figure 78. MALDI–ToF overview spectrum of **9b**. All peaks belong to the target molecule with a different counter ion. All peak assignments can be found in Supplementary Table 15.

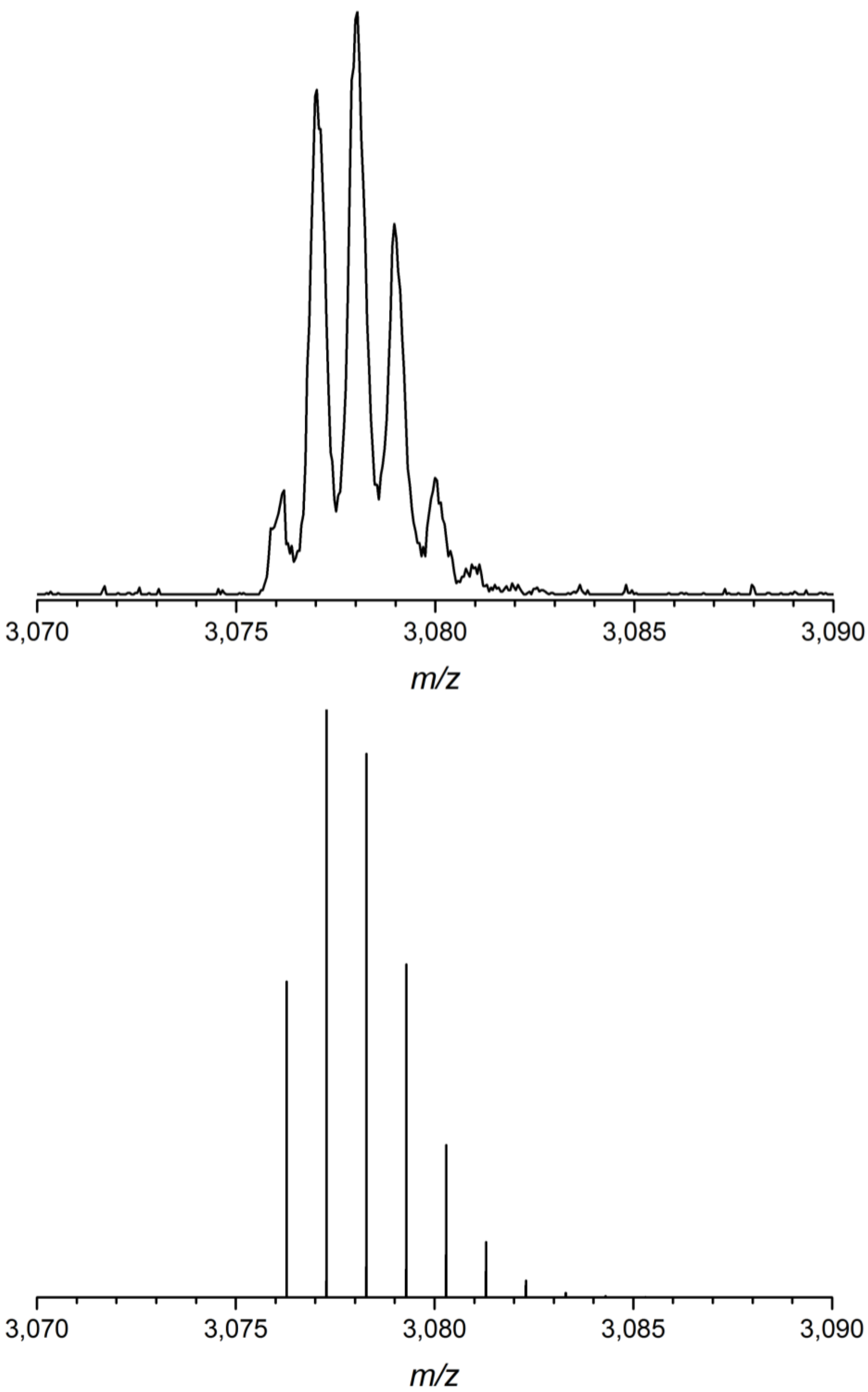

Supplementary Figure 79. MALDI–ToF experimental (top) and calculated (bottom) zoom spectra of **9b**.

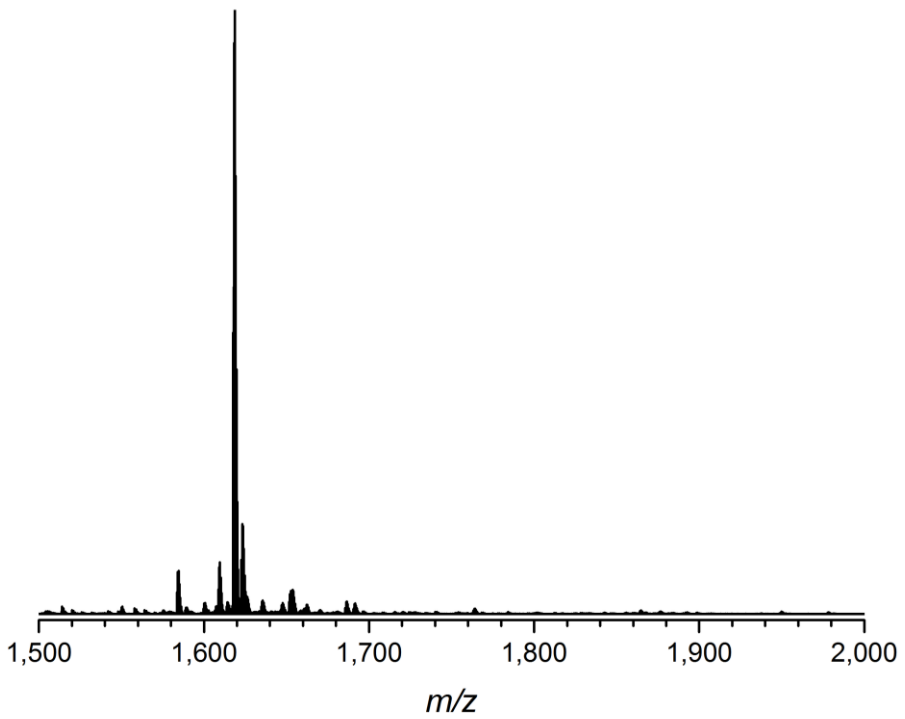

Supplementary Figure 80. ESI-MS overview spectrum of **9b**. All major peaks belong to the target molecule with different counter ions. All peak assignments can be found in Supplementary Table 15.

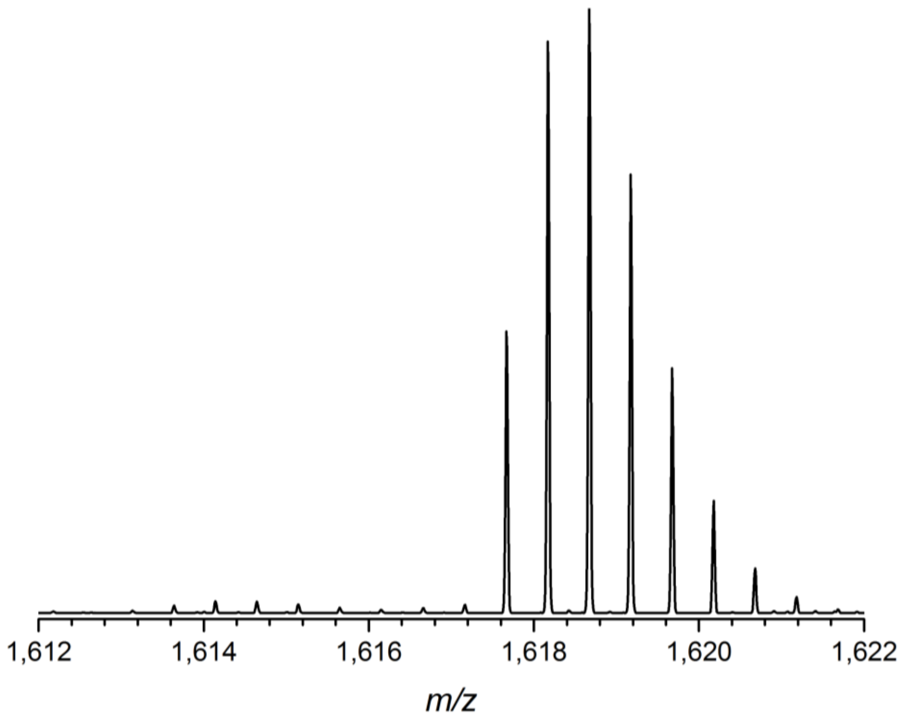

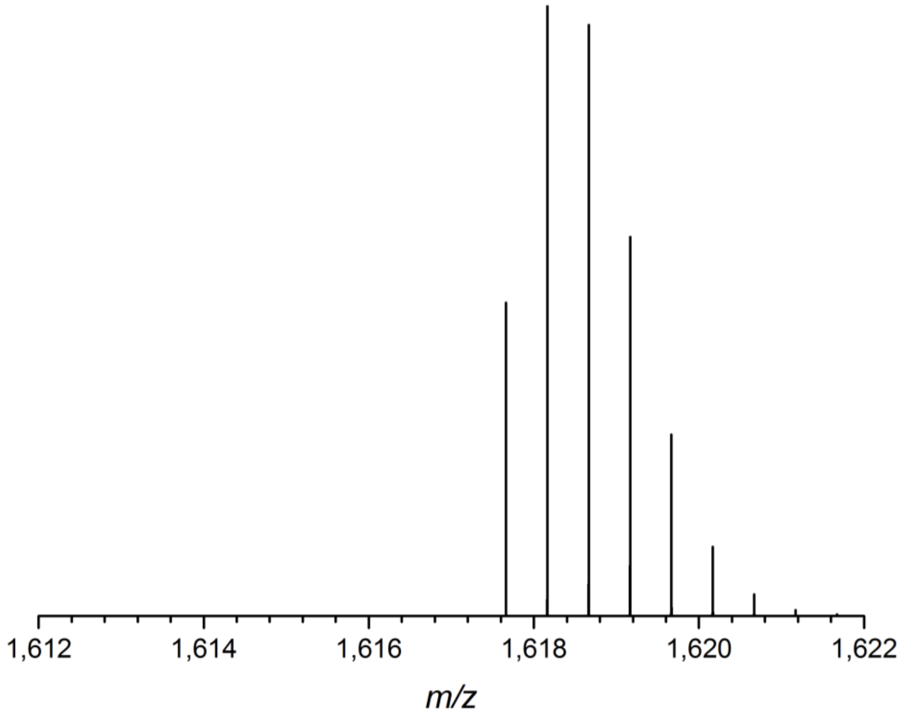

Supplementary Figure 81. ESI-MS experimental (top) and calculated (bottom) zoom spectra of **9b**.

Characterization of 9b ($M_1M_2M_1$)-X-($M_1M_2M_1$)

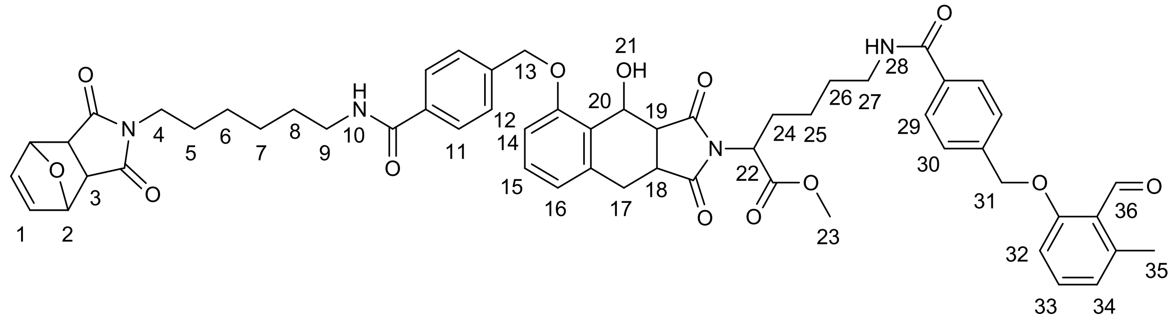

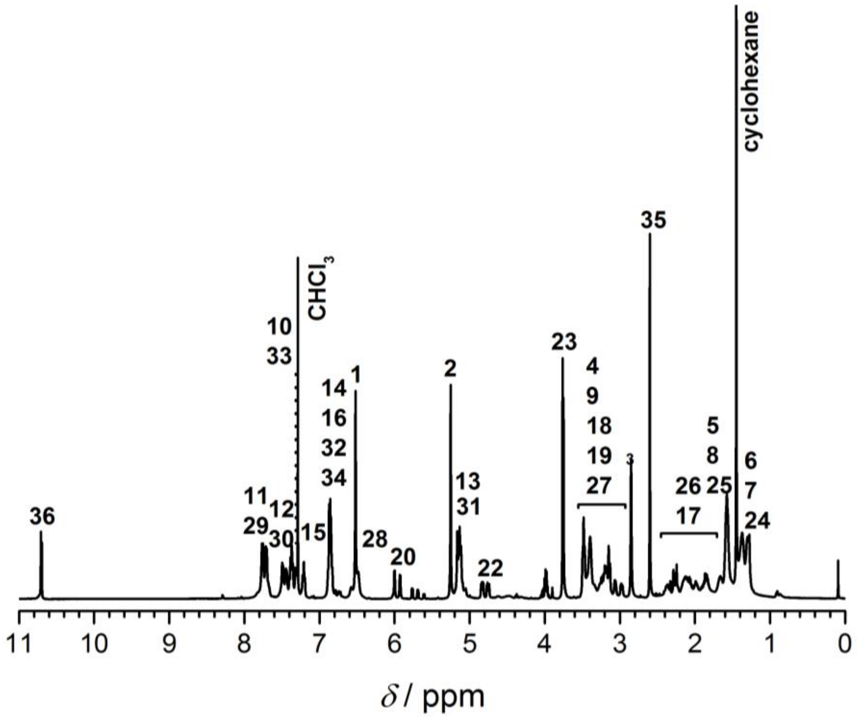

Supplementary Figure 82. ^{1}H NMR spectrum of **9c** ($CDCl_3$).

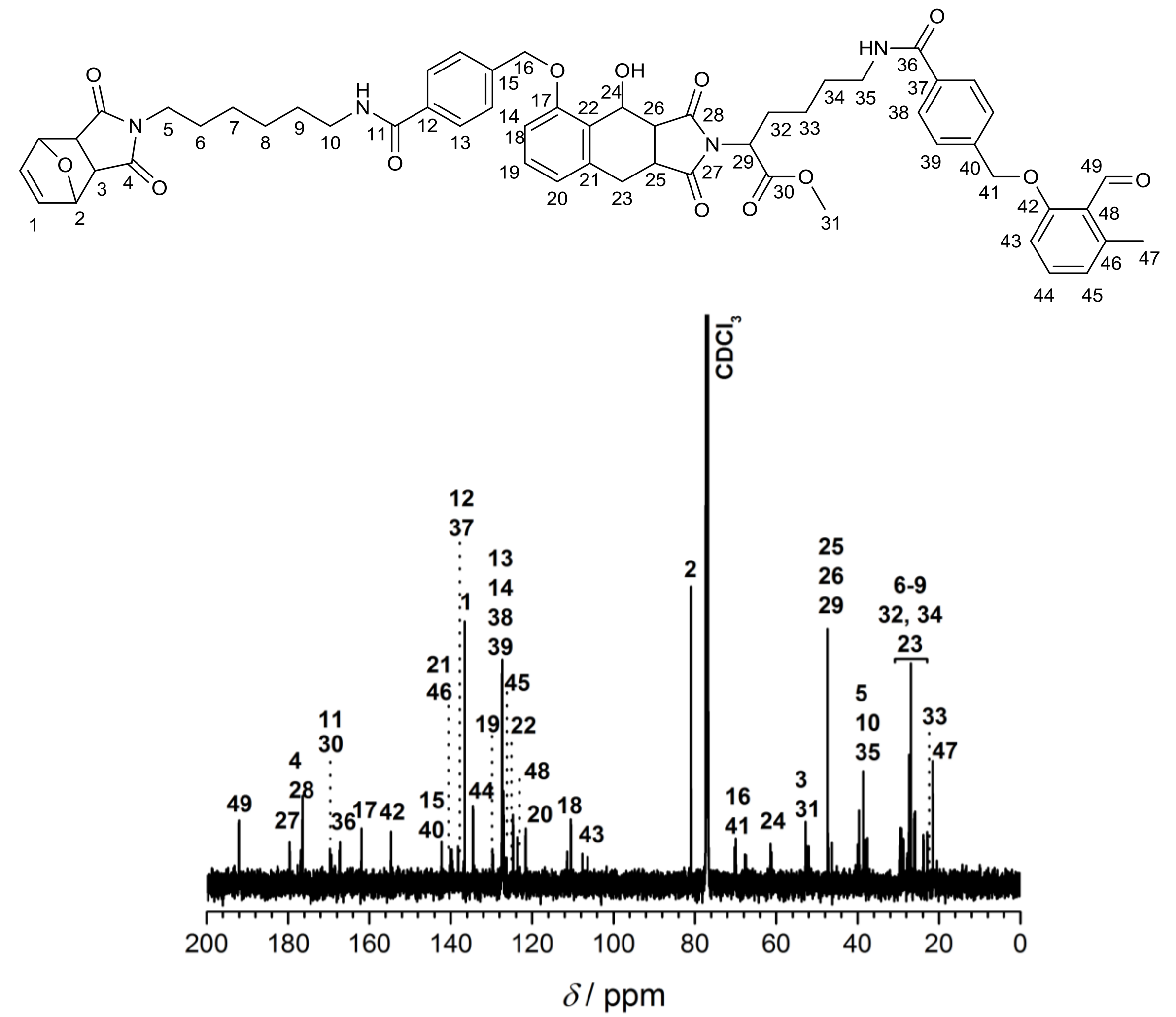

Supplementary Figure 83. ^{13}C NMR spectrum of **9c** ($CDCl_3$).

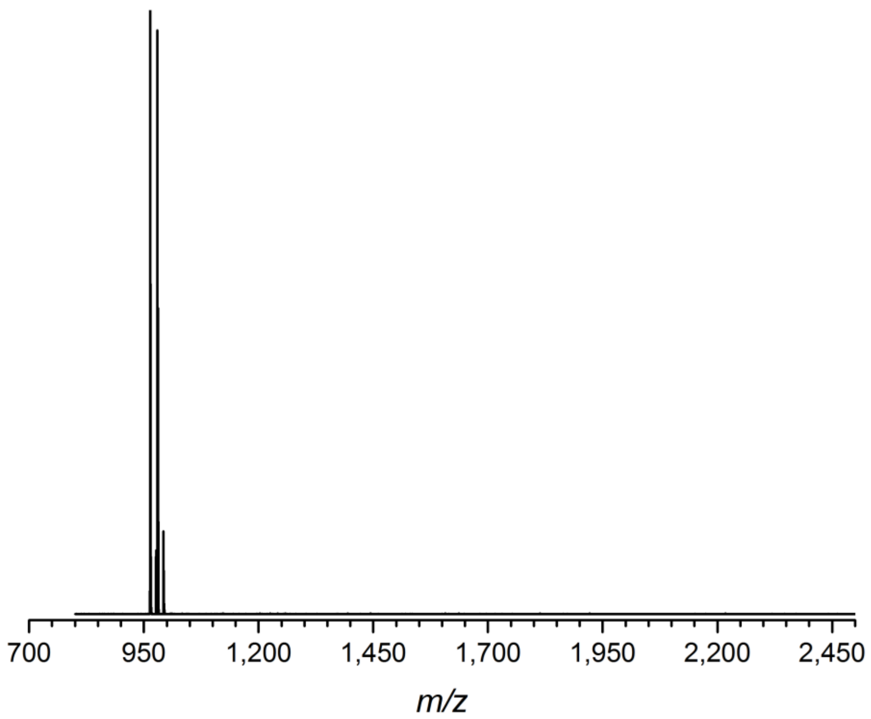

Supplementary Figure 84. MALDI–ToF overview spectrum of **9c**. All major peaks belong to the target molecule with different counter ions. All peak assignments can be found in Supplementary Table 16.

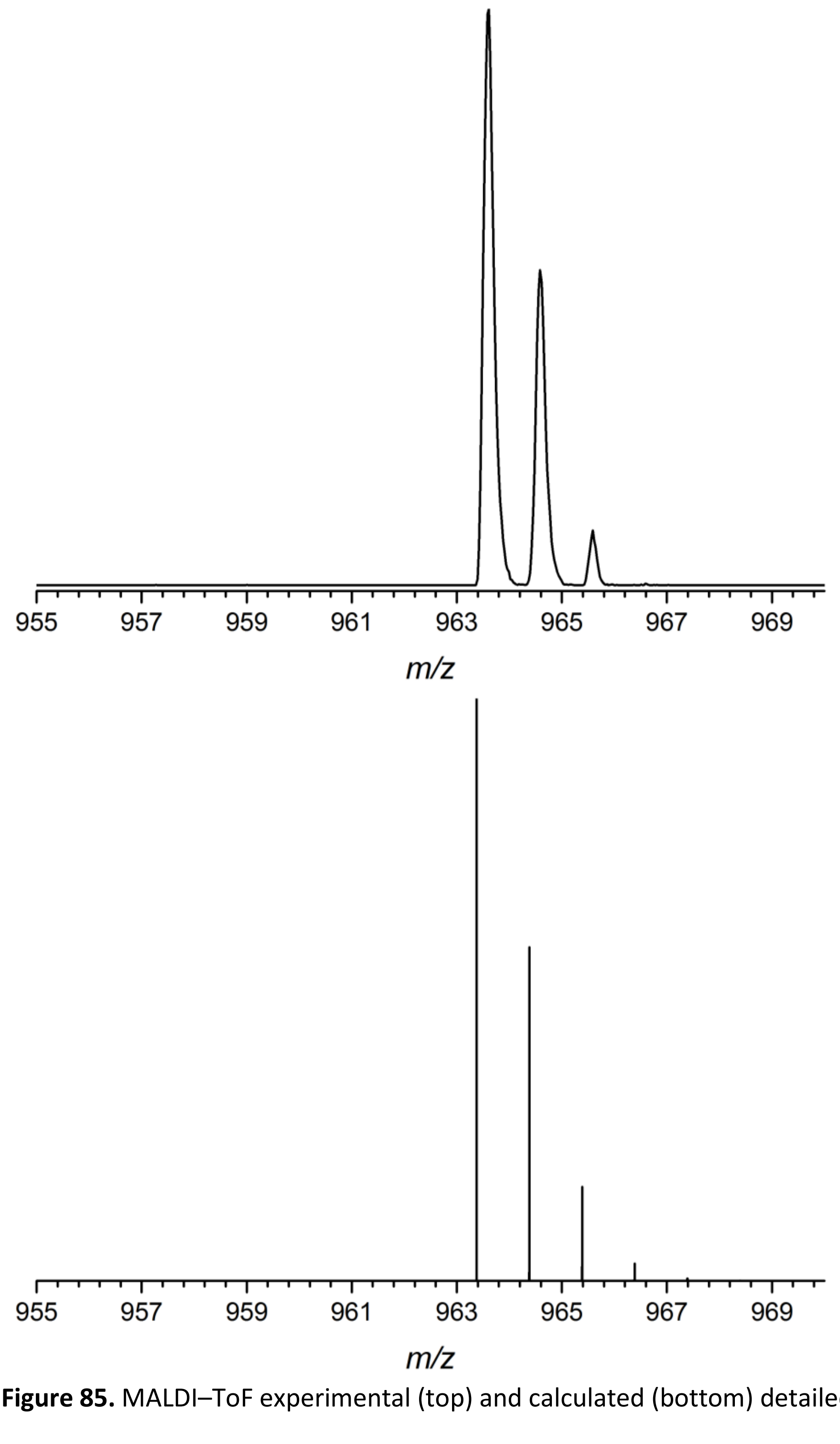

Supplementary Figure 85. MALDI–ToF experimental (top) and calculated (bottom) detailed spectra of **9c**.

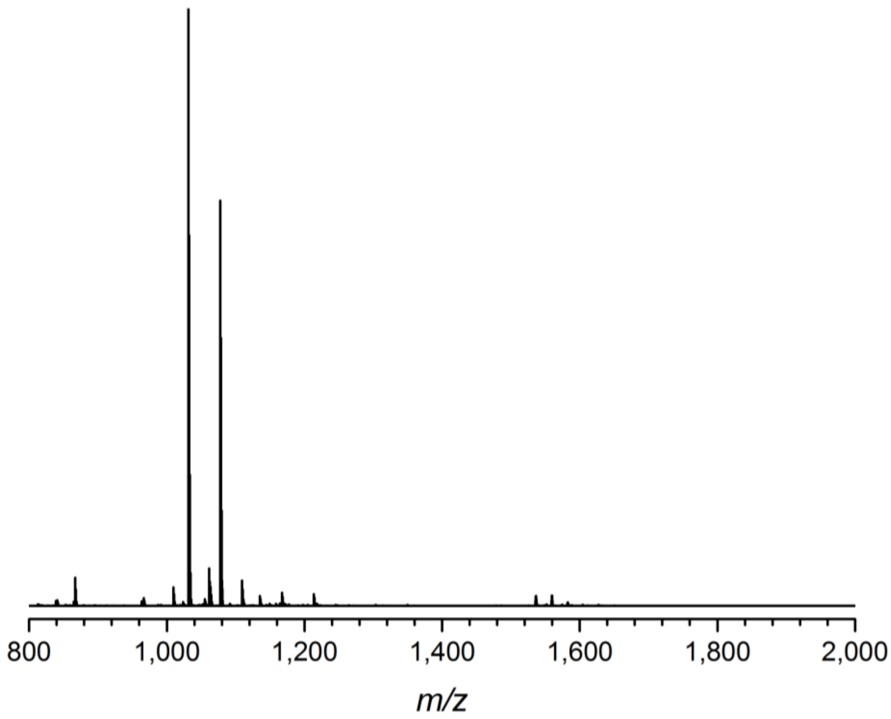

Supplementary Figure 86. ESI-MS overview spectrum of **9c**. All peaks belong to the target molecule with a different counter ion. All peak assignments can be found in Supplementary Table 16.

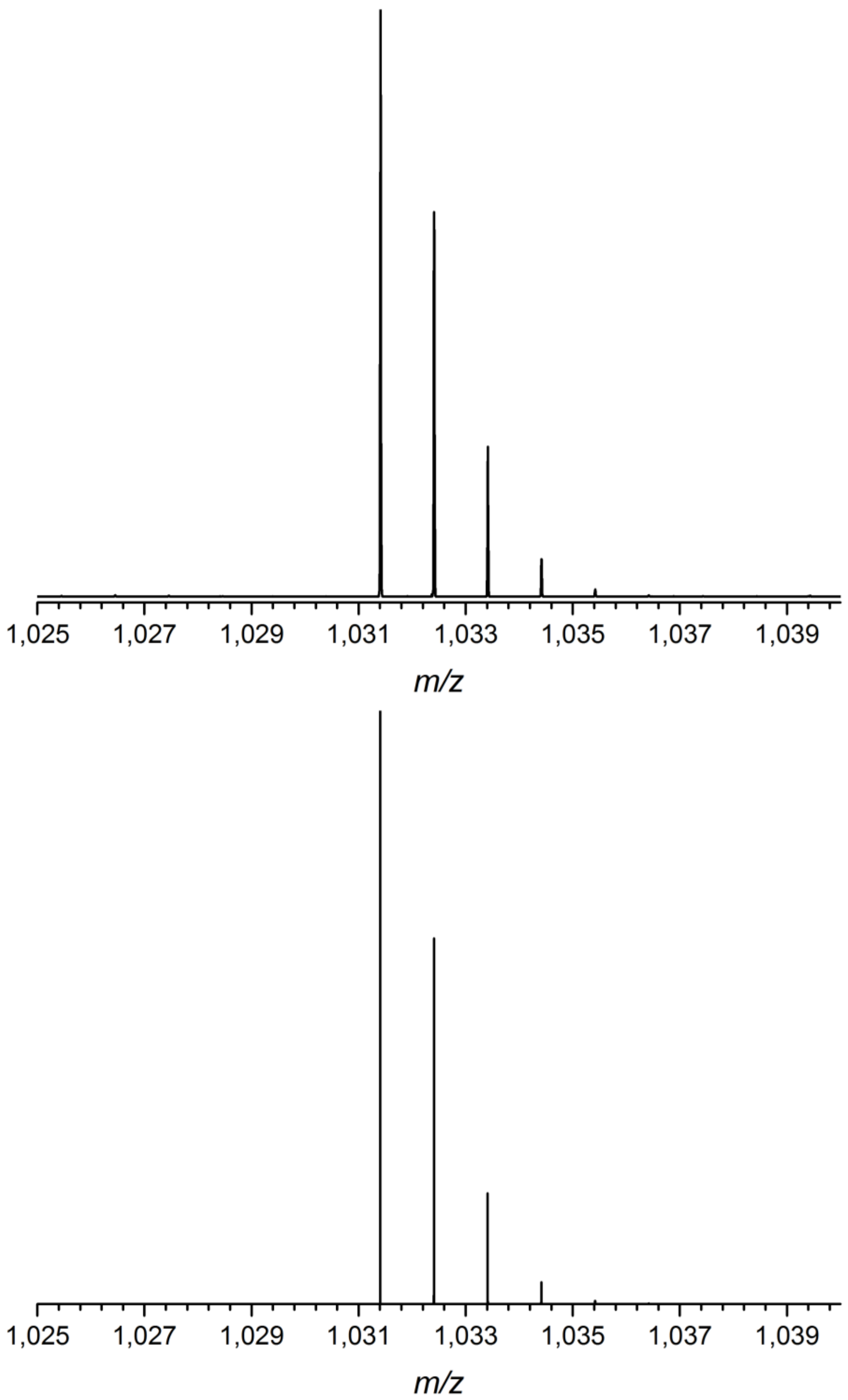

Supplementary Figure 87. ESI-MS experimental (top) and calculated (bottom) zoom spectra of **9c**.

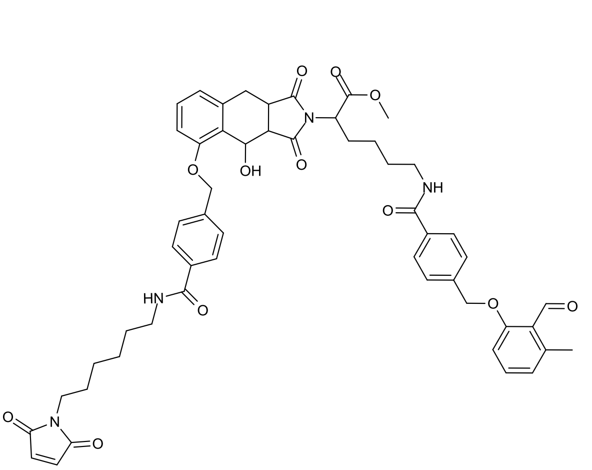

A_1

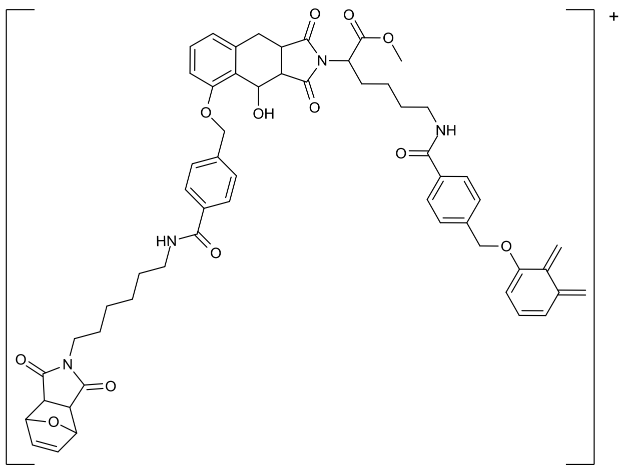

A_2^+

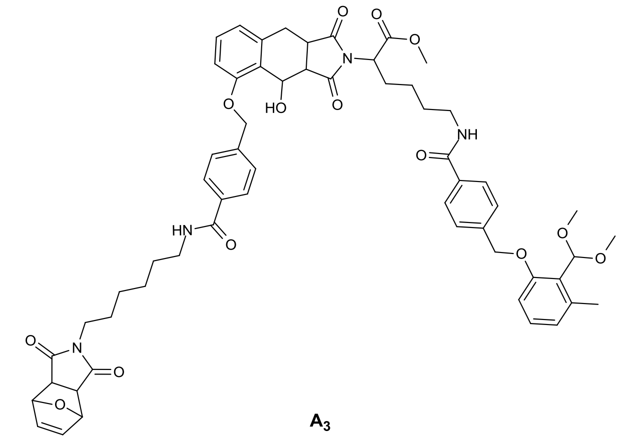

A_3

Supplementary Figure 88. Collation of molecules identified in Supplementary Table 16 (**A_1**-**A_3**).

Characterization of 9 ((M_1)(M_2M_1)$_2$-X-(M_1M_2)$_2$(M_1))

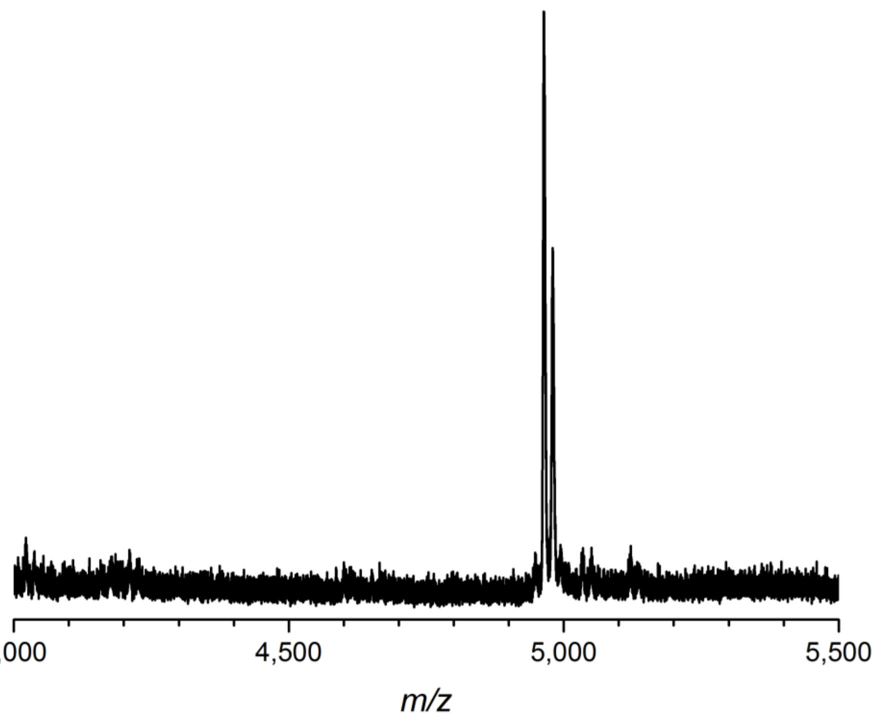

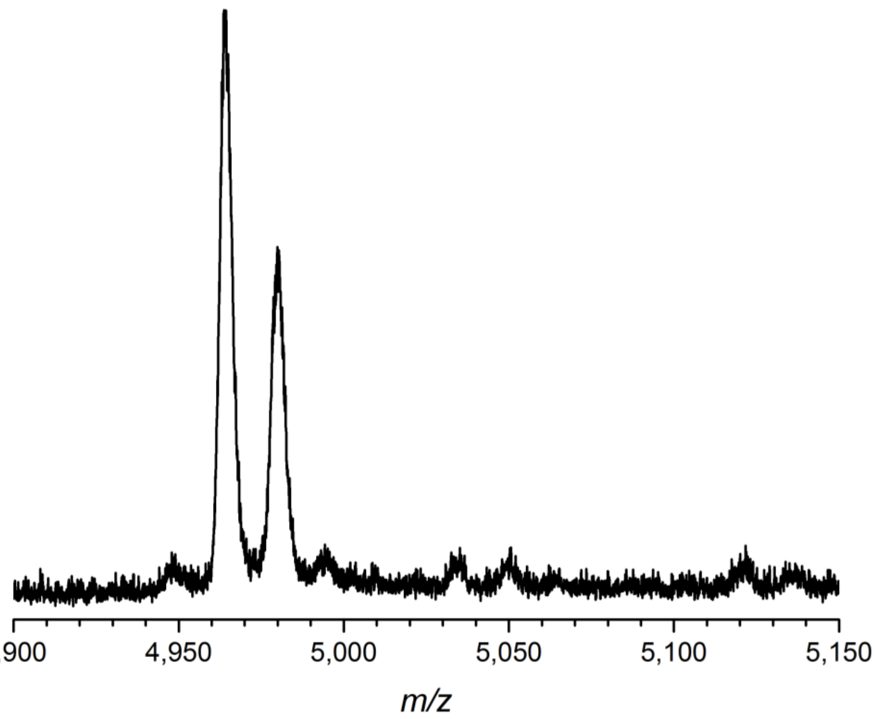

Supplementary Figure 89. MALDI–ToF overview (top) and detailed (bottom) zoom spectra of **9**. All major peaks belong to the target molecule with different counter ions. All peak assignments can be found in Supplementary Table 17.

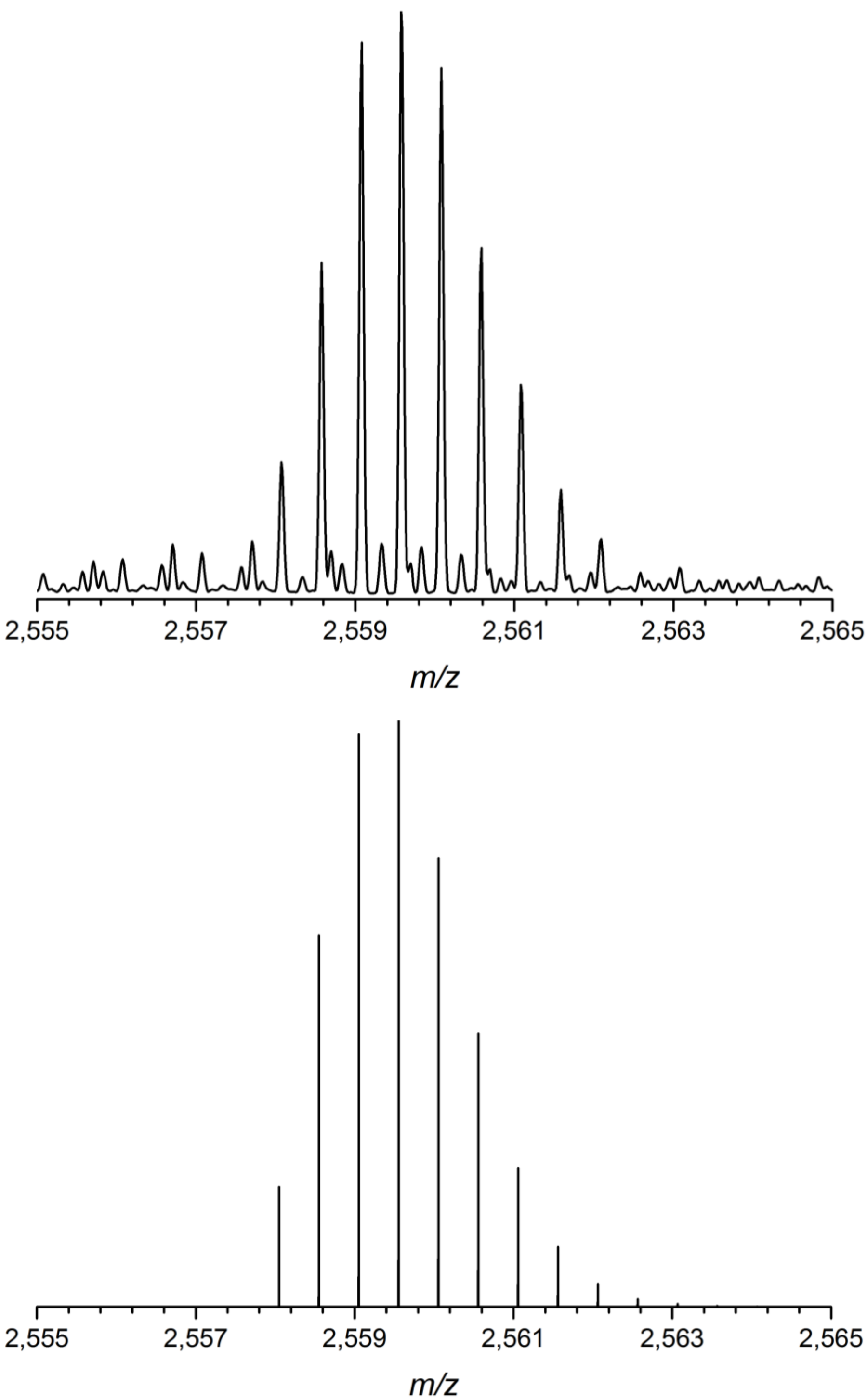

Supplementary Figure 90. ESI-MS experimental (top) and calculated (bottom) zoom spectra of **9**.

Characterization of symmetric Copolymer 10 ((M_2)(M_1M_2)$_2$-X-(M_2M_1)$_2$(M_2)) and precursors 10a-d (10a: (M_2)-X-(M_2), 10b: (M_1M_2)-X-(M_2M_1), 10c: ($M_2M_1M_2$)-X-($M_2M_1M_2$), 10d: (M_2M_1))

Characterization of 10a (M_2)-X-(M_2)

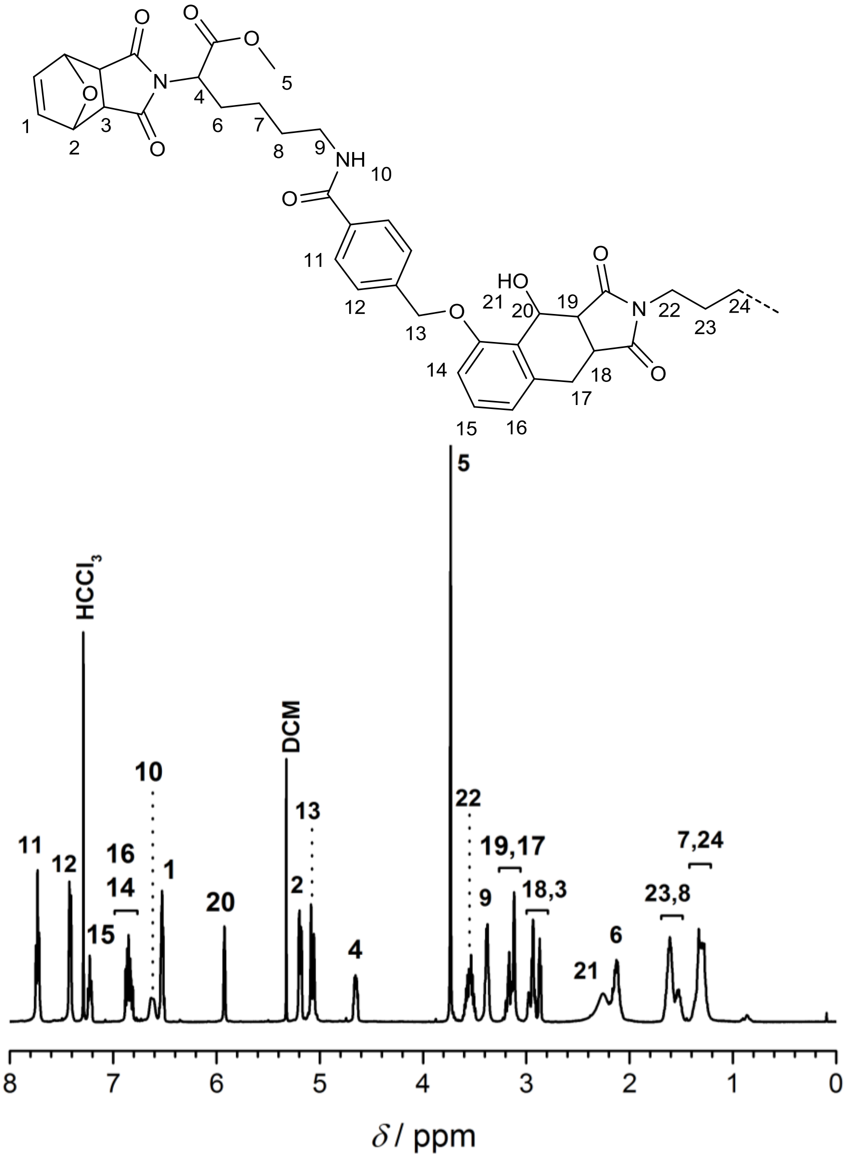

Supplementary Figure 91. ^{1}H NMR spectrum of **10a** ($CDCl_3$).

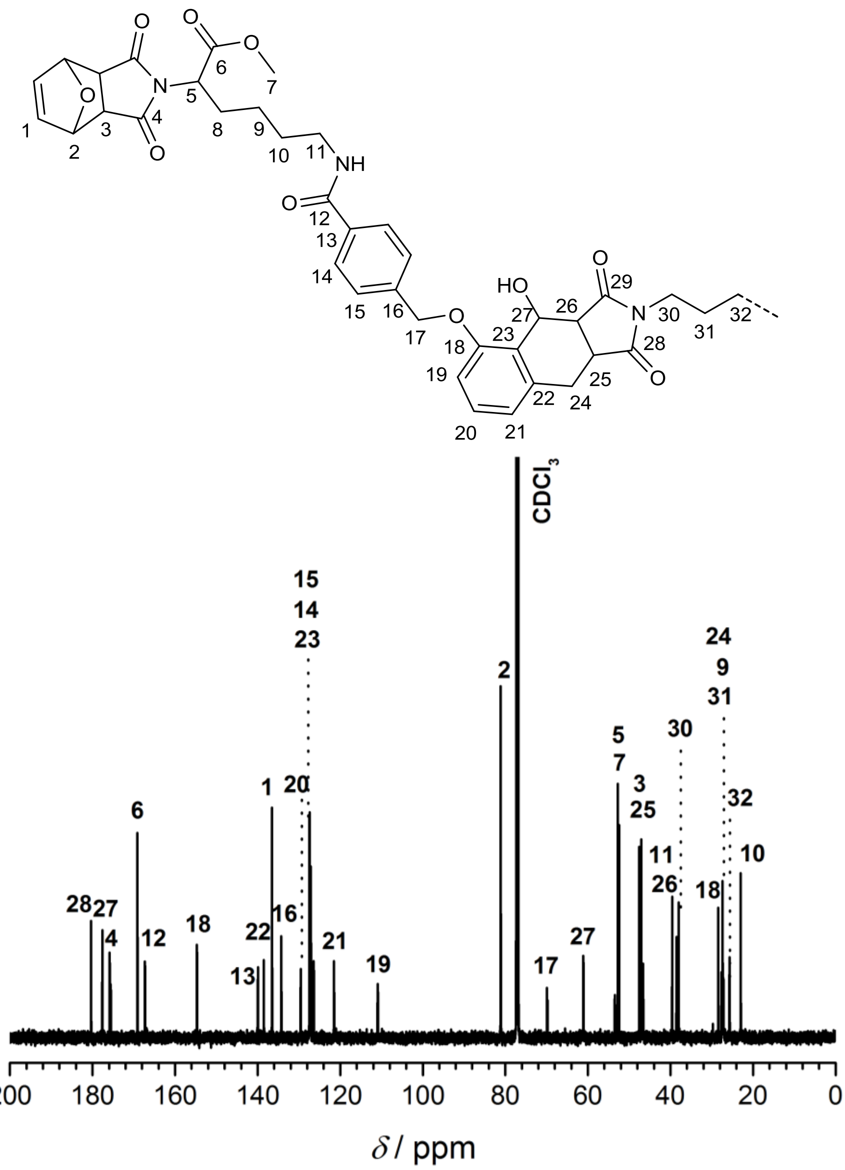

Supplementary Figure 92. ^{13}C NMR spectrum of **10a** ($CDCl_3$).

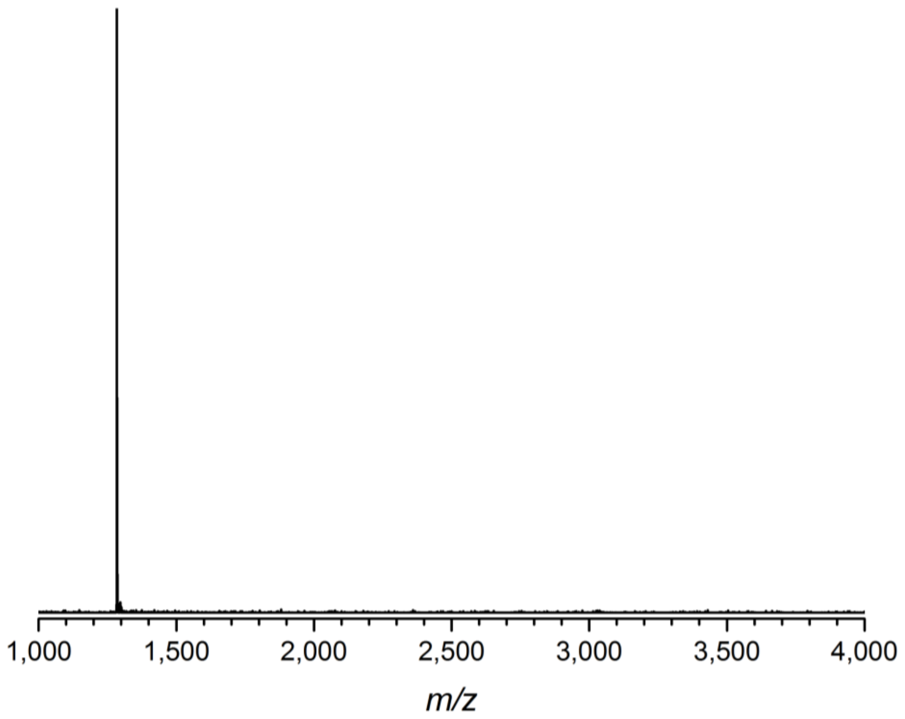

Supplementary Figure 93. MALDI–ToF overview spectrum of **10a**. All peaks belong to the target molecule with a different counter ion. All peak assignments can be found in Supplementary Table 18.

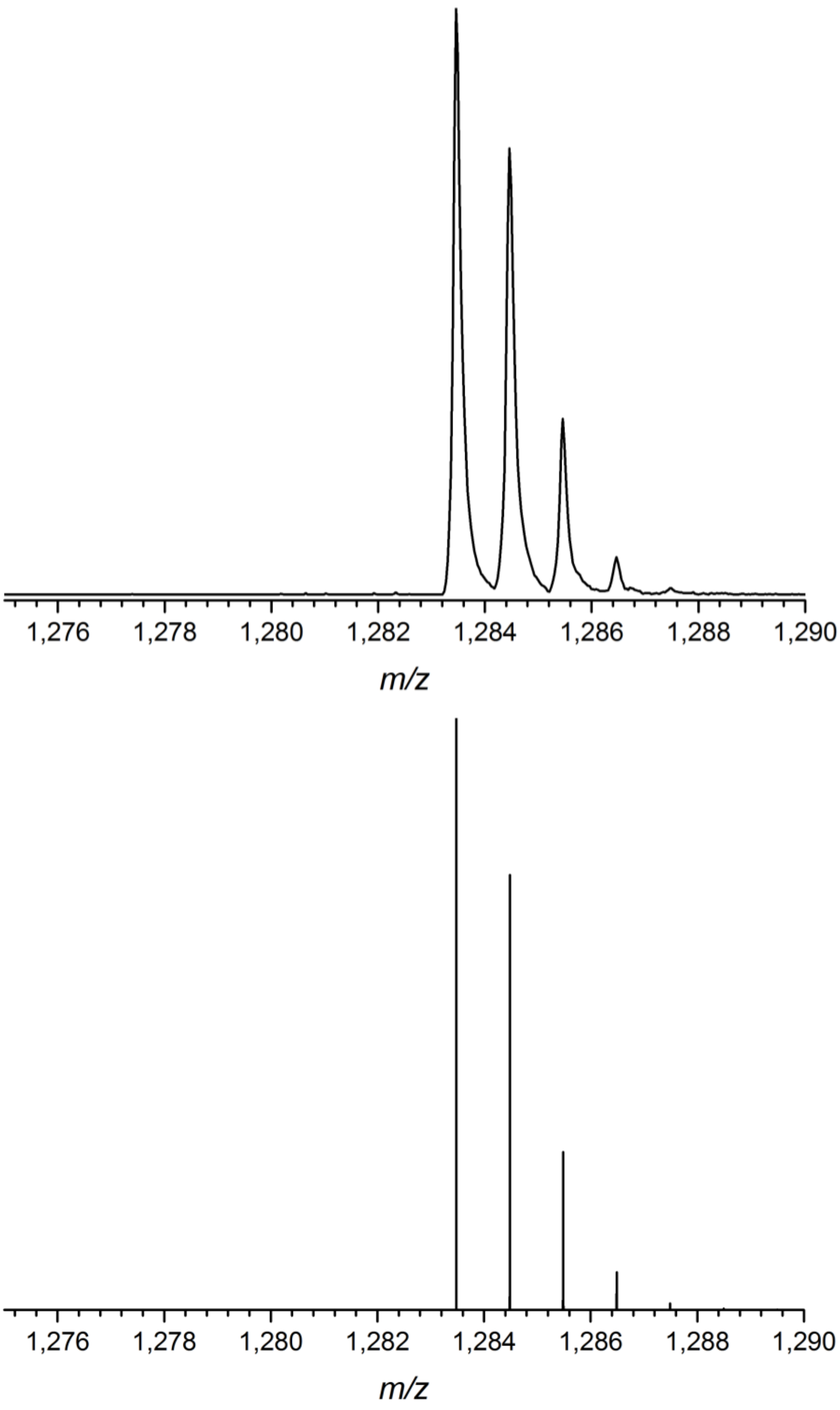

Supplementary Figure 94. MALDI–ToF experimental (top) and calculated (bottom) zoom spectra of **10a**.

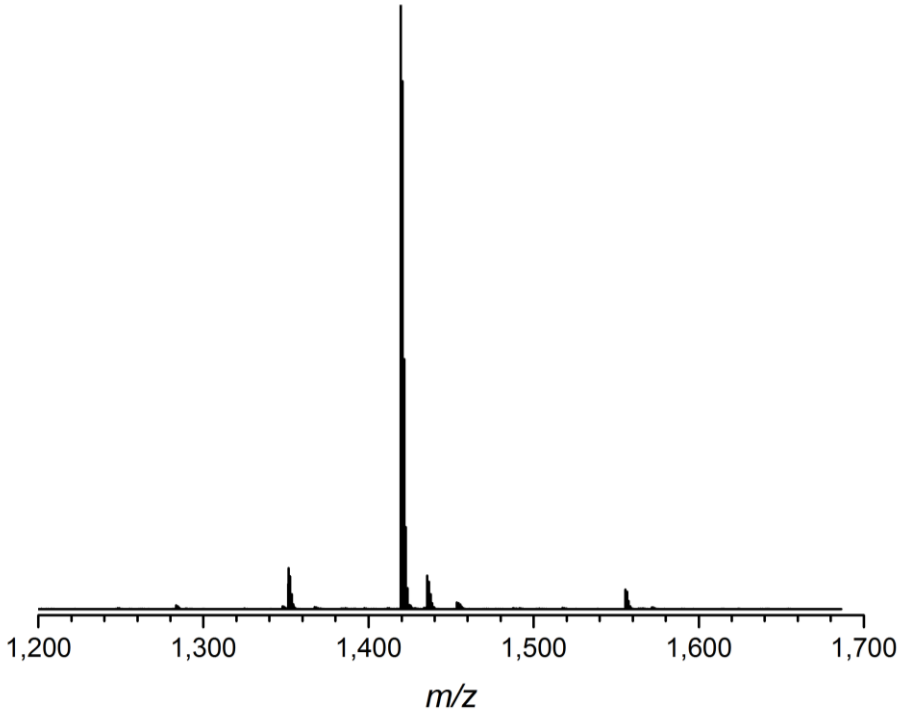

Supplementary Figure 95. ESI-MS overview spectrum of **10a**. All major peaks belong to the target molecule with different counter ions. All peak assignments can be found in Supplementary Table 18.

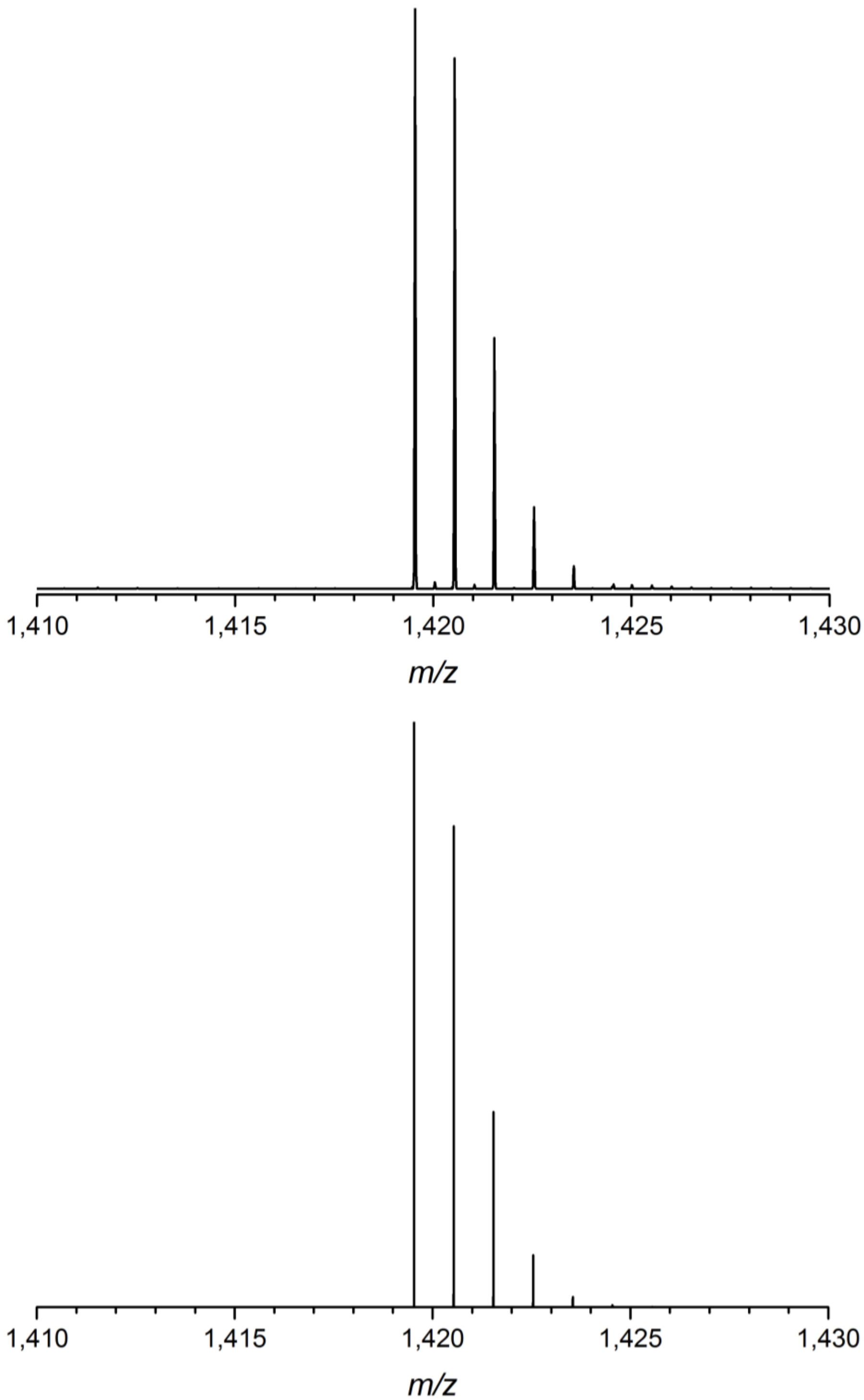

Supplementary Figure 96. ESI-MS experimental (top) and calculated (bottom) zoom spectra of **10a**.

Characterization of 10b (M_1M_2)-X-(M_2M_1)

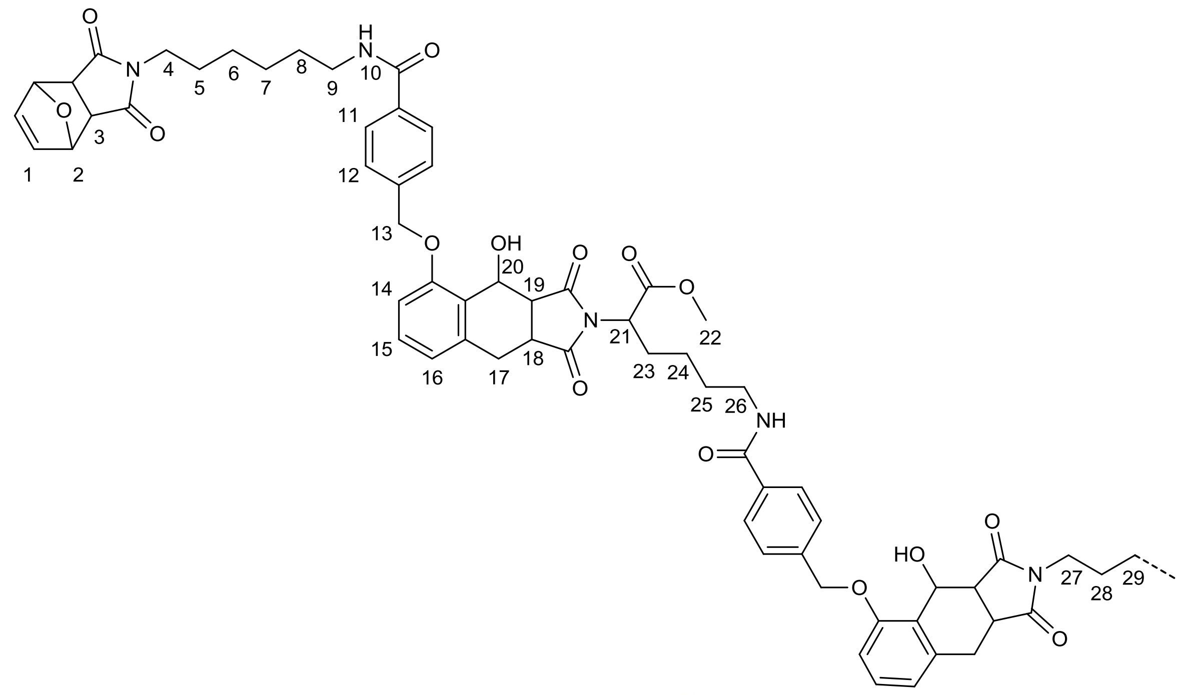

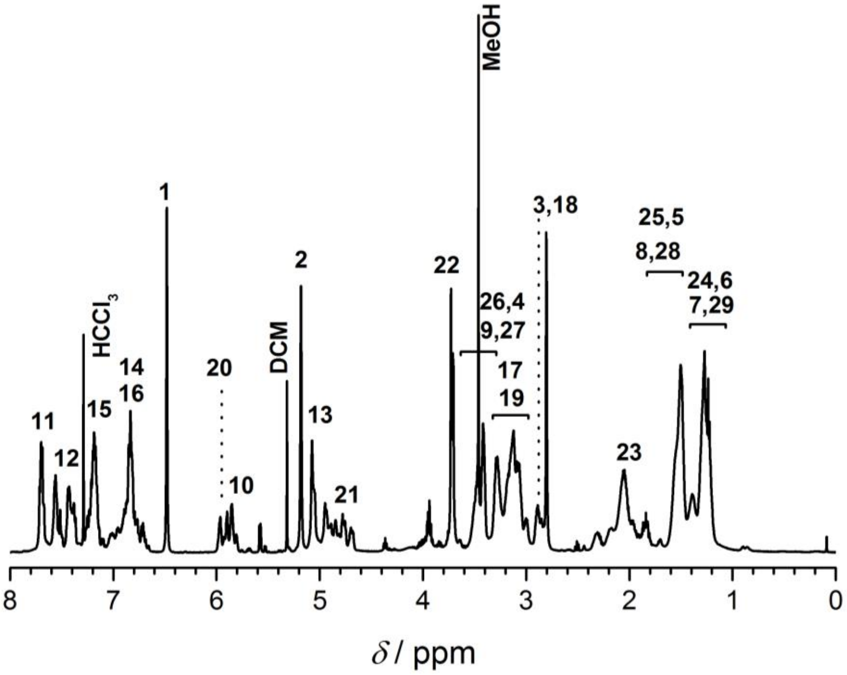

Supplementary Figure 97. ^{1}H NMR spectrum of **10b** ($CDCl_3$).

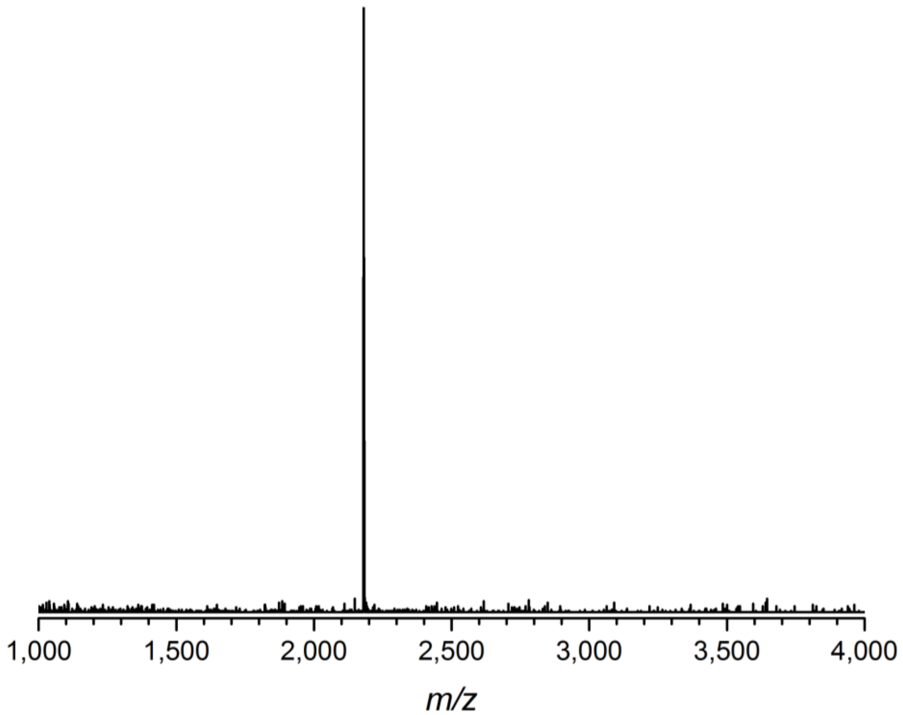

Supplementary Figure 98. MALDI–ToF overview spectrum of **10b**. All peaks belong to the target molecule with a different counter ion. All peak assignments can be found in Supplementary Table 19.

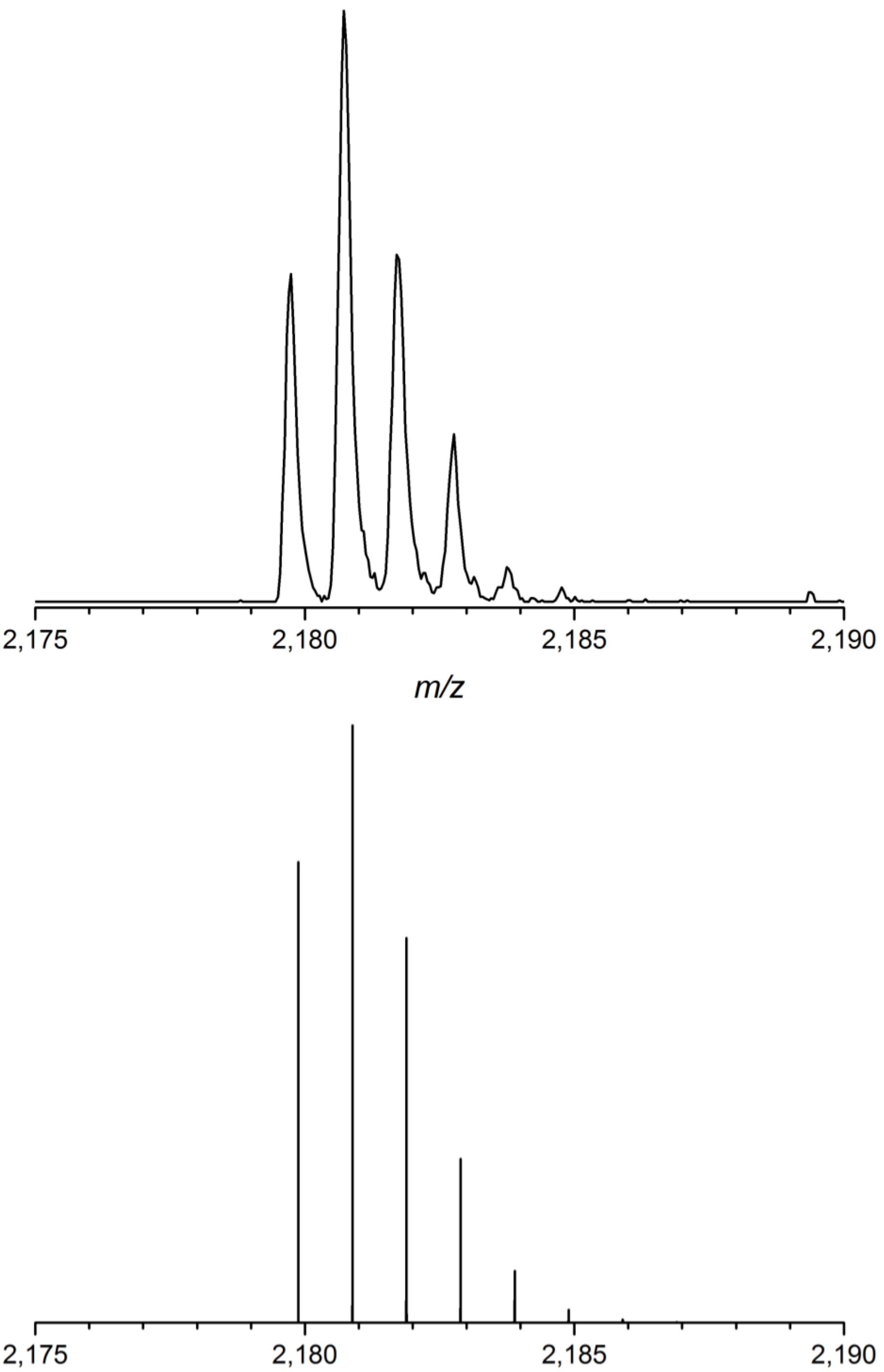

Supplementary Figure 99. MALDI–ToF experimental (top) and calculated (bottom) zoom spectra of **10b**.

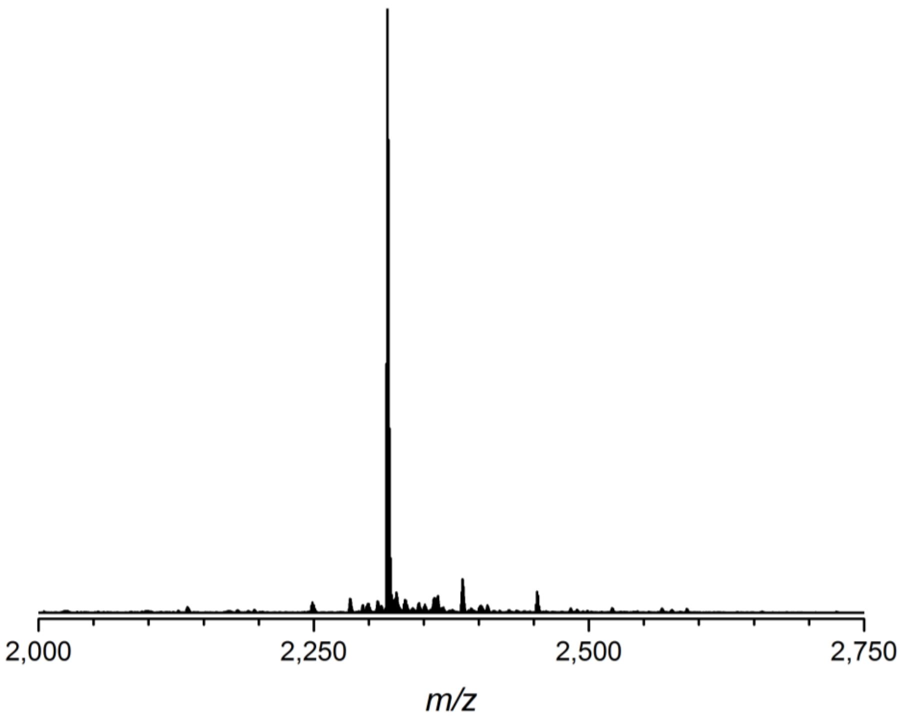

Supplementary Figure 100. ESI-MS overview spectrum of **10b**. All major peaks belong to the target molecule with different counter ions. All peak assignments can be found in Supplementary Table 19.

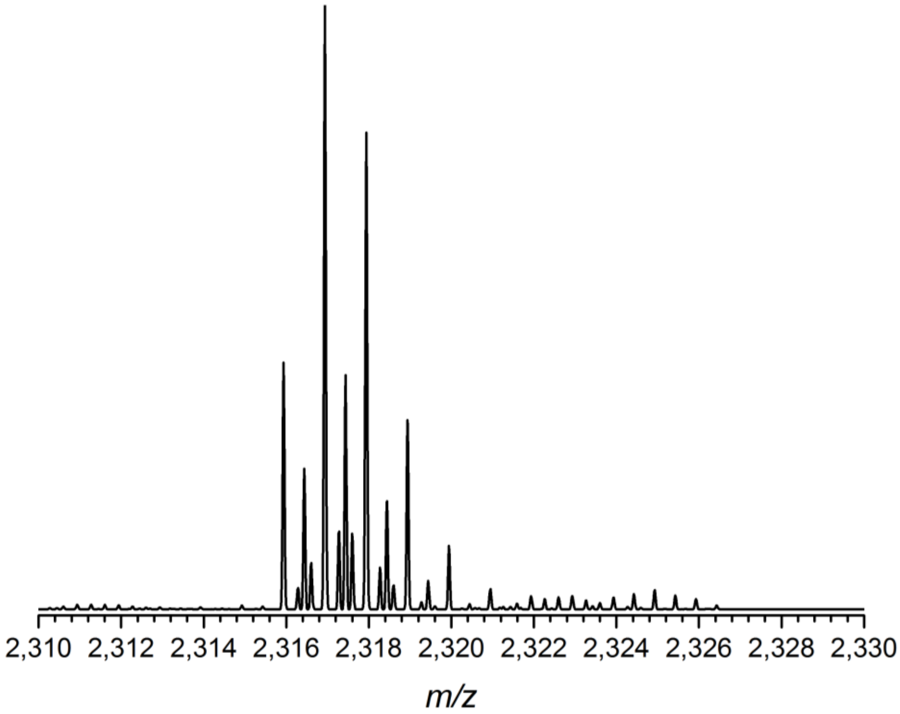

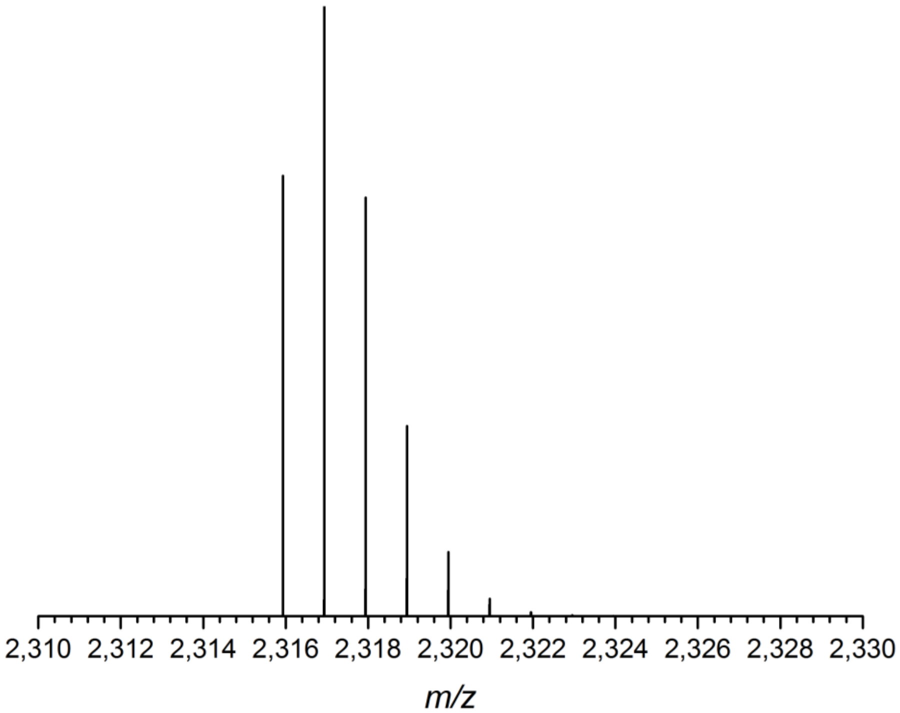

Supplementary Figure 101. ESI-MS experimental (top) and calculated (bottom) zoom spectra of **10b**. The exact mass of the measured sample is matching with the assigned species (see Supplementary Table 19), however there is likely an additional signal from a double charged cluster of two molecules.

Characterization of 10c ($M_2M_1M_2$)-X-($M_2M_1M_2$)

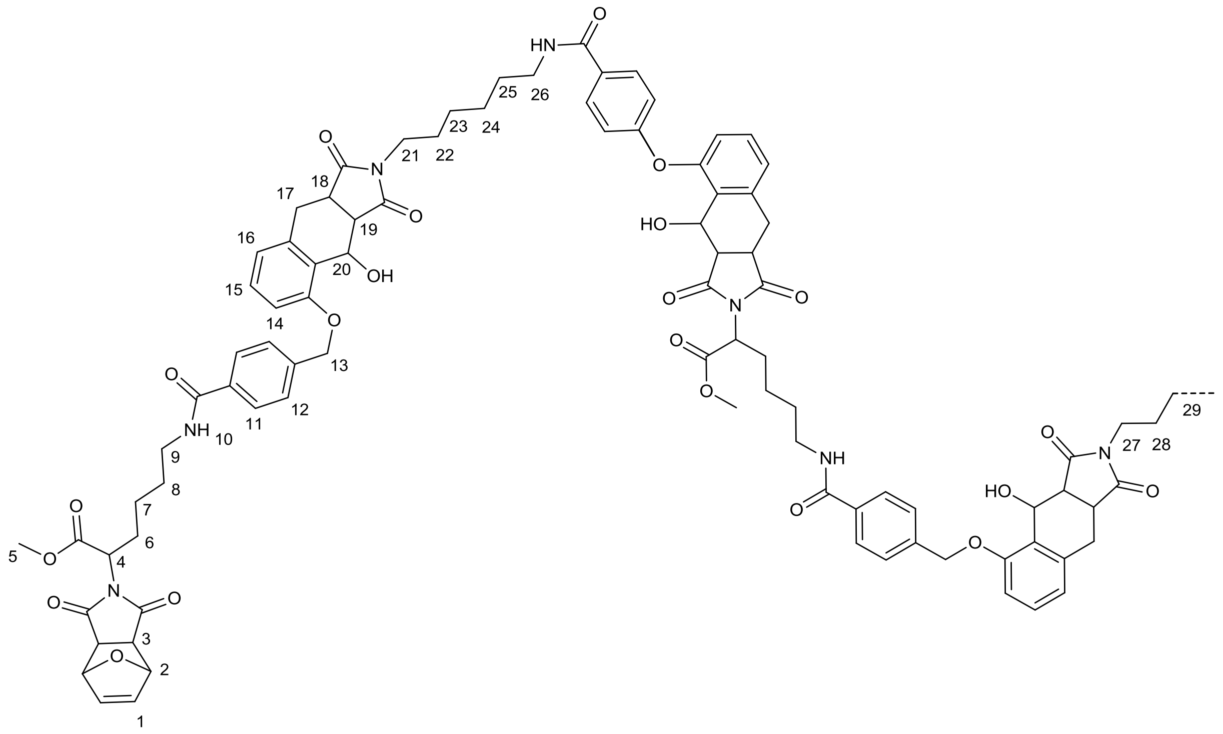

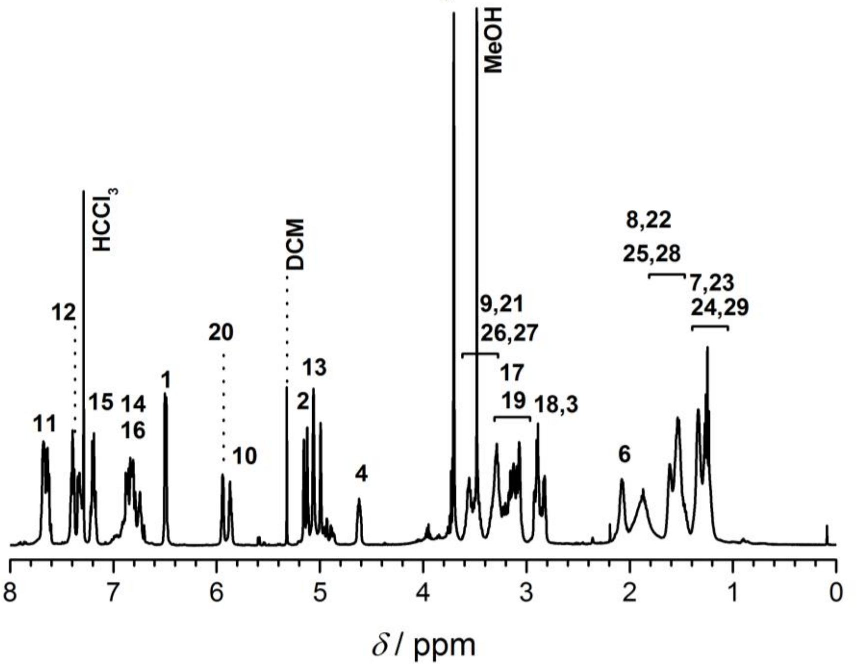

Supplementary Figure 102. ^{1}H NMR spectrum of **10c** ($CDCl_3$).

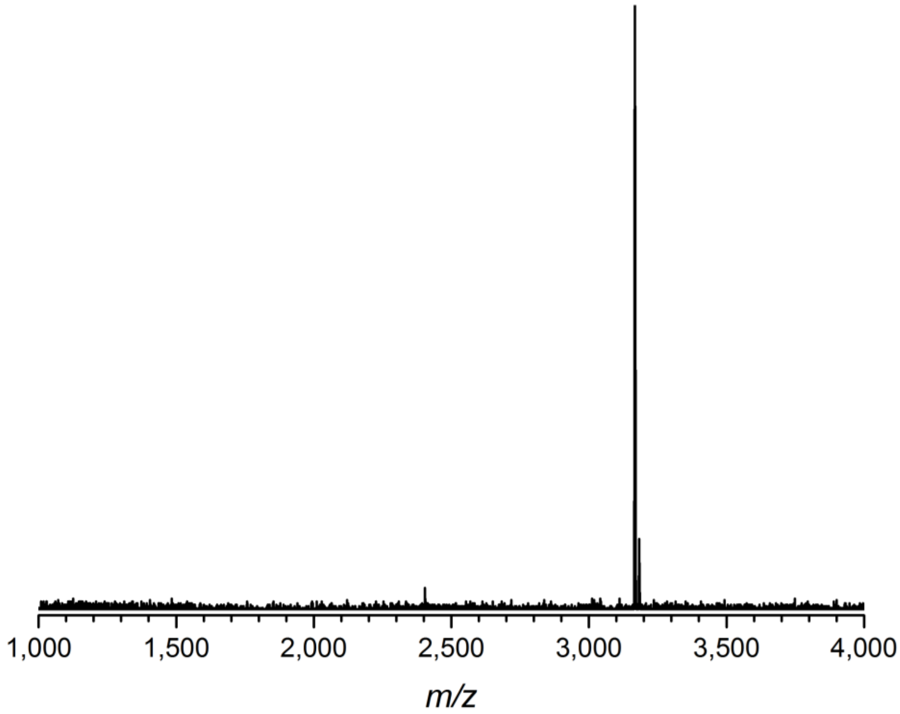

Supplementary Figure 103. MALDI–ToF overview spectrum of **10c**. All major peaks belong to the target molecule with different counter ions. All peak assignments can be found in Supplementary Table 20.

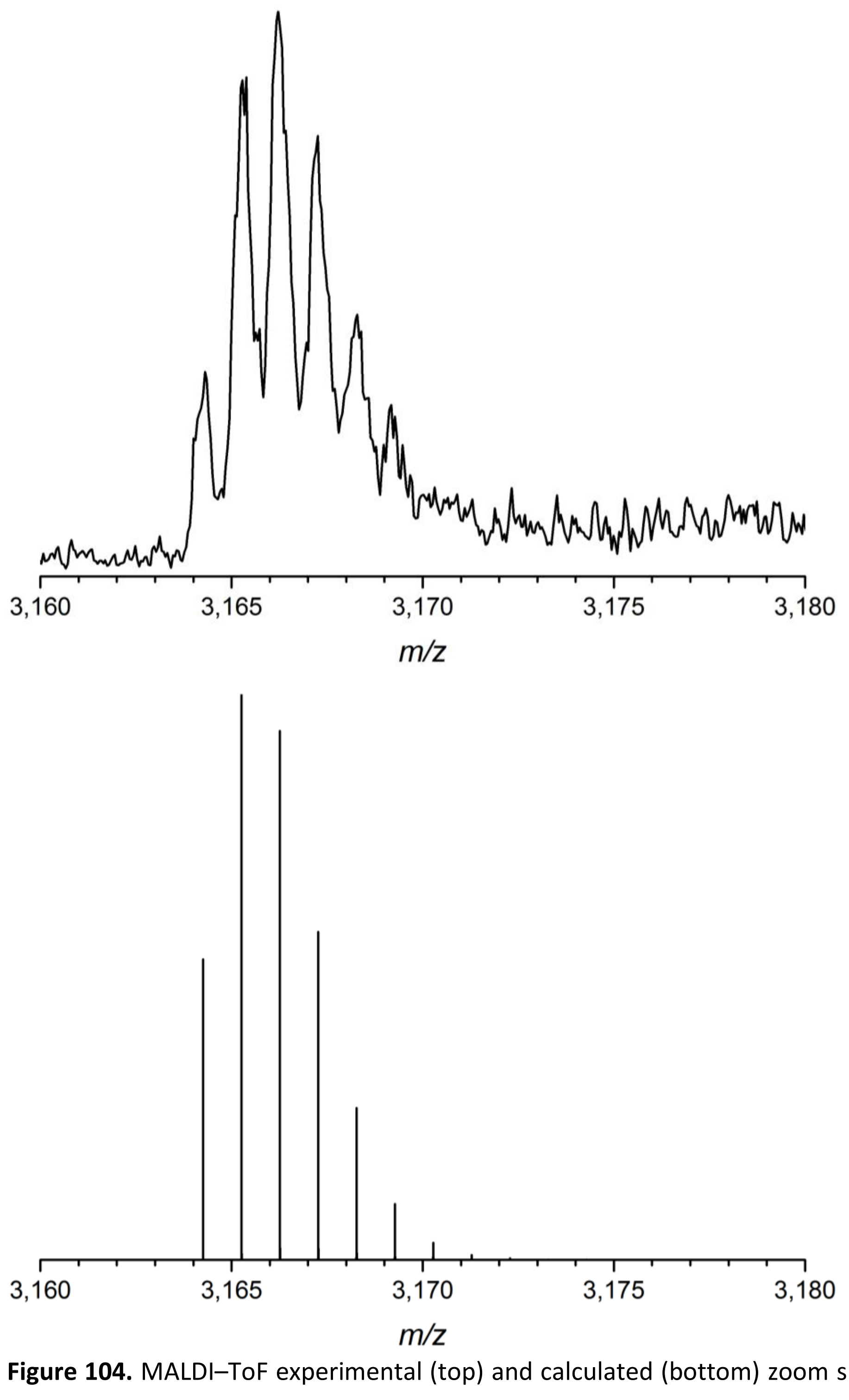

Supplementary Figure 104. MALDI–ToF experimental (top) and calculated (bottom) zoom spectra of **10c**.

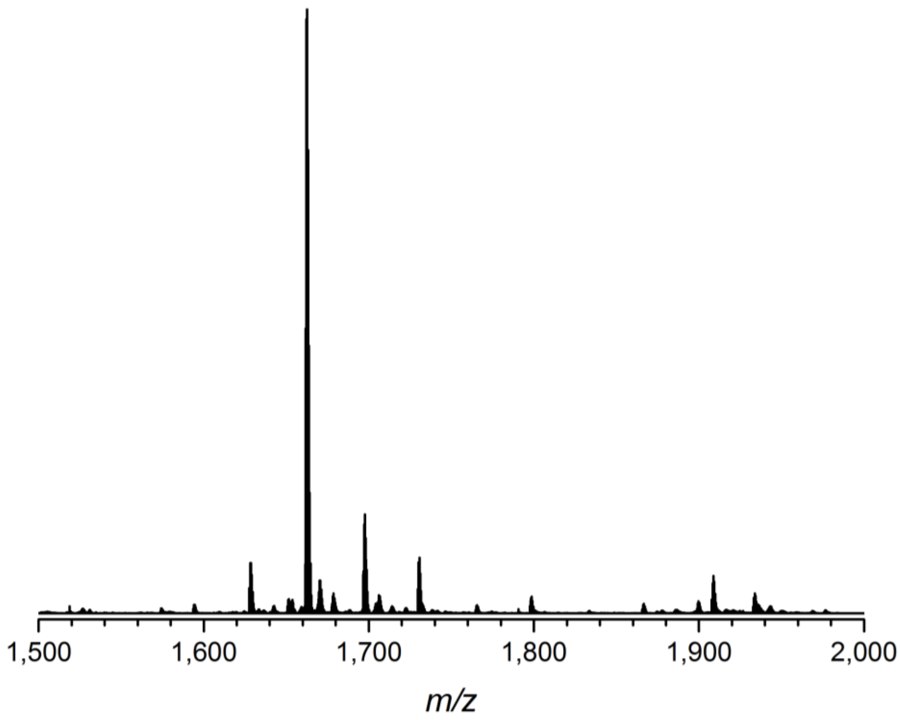

Supplementary Figure 105. ESI-MS overview spectrum of **10c**. All major peaks belong to the target molecule with different counter ions. All peak assignments can be found in Supplementary Table 20.

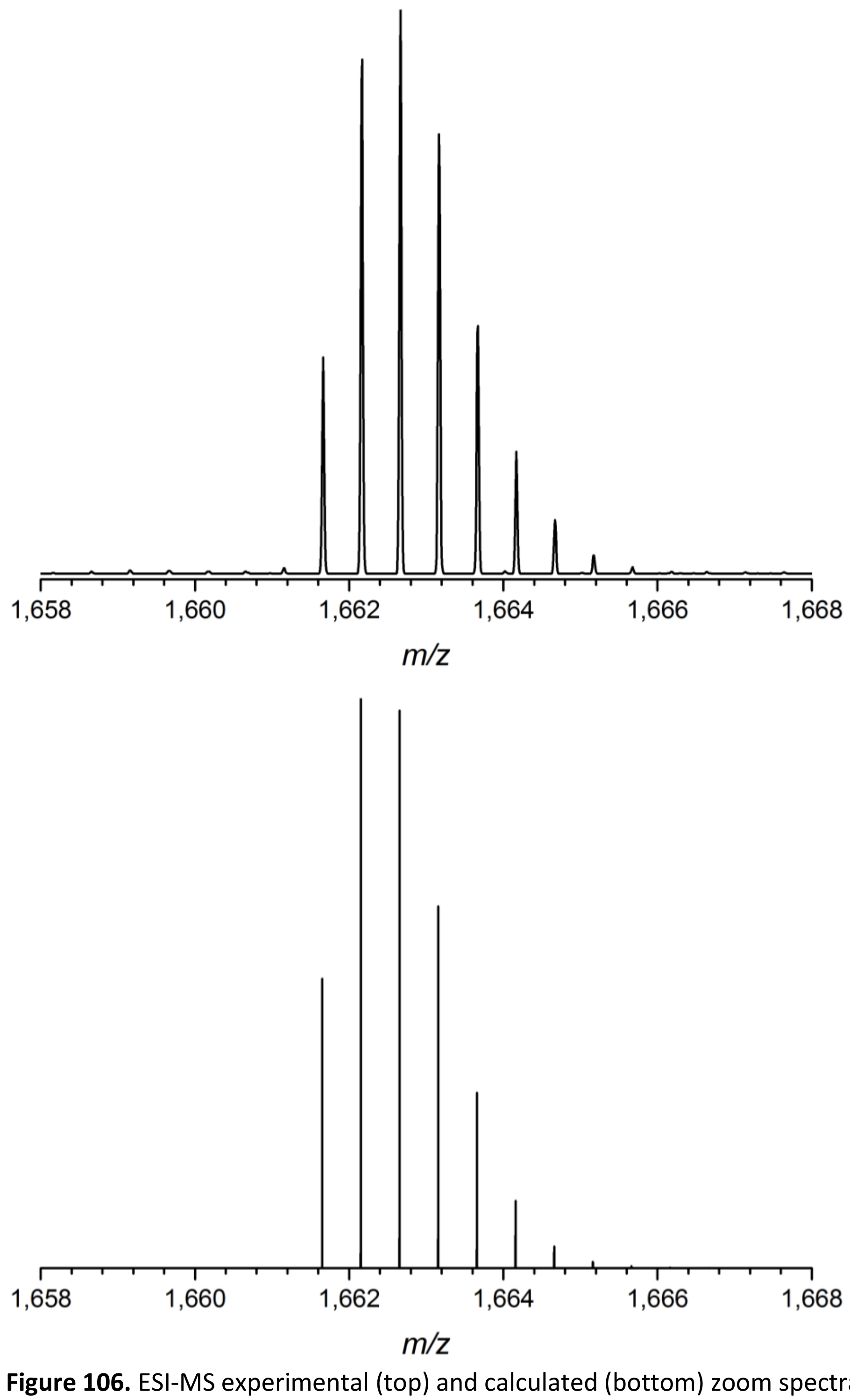

Supplementary Figure 106. ESI-MS experimental (top) and calculated (bottom) zoom spectra of **10c**.

Characterization of 10d (M_2M_1)

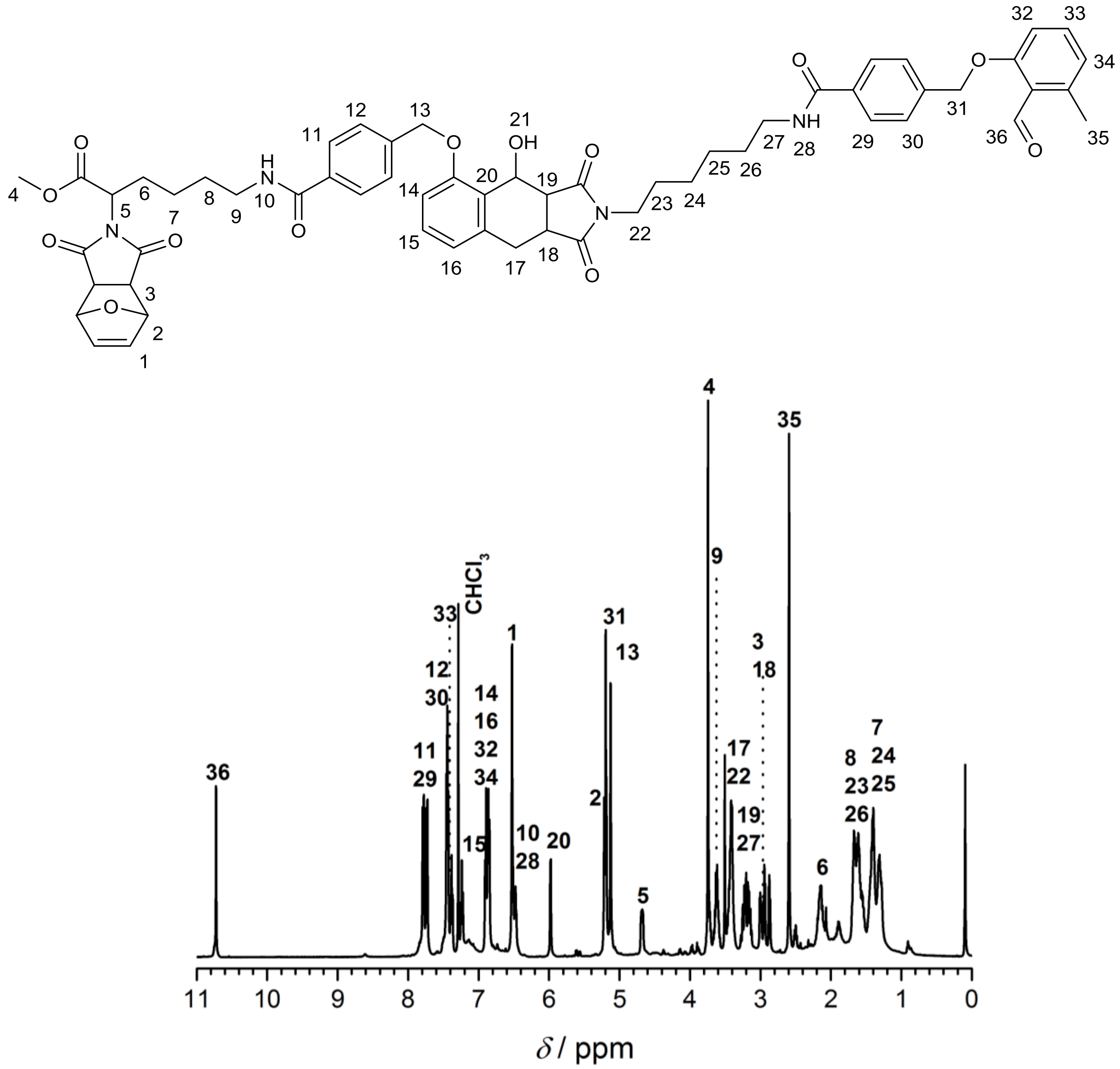

Supplementary Figure 107. ^{1}H NMR spectrum of **10d** ($CDCl_3$).

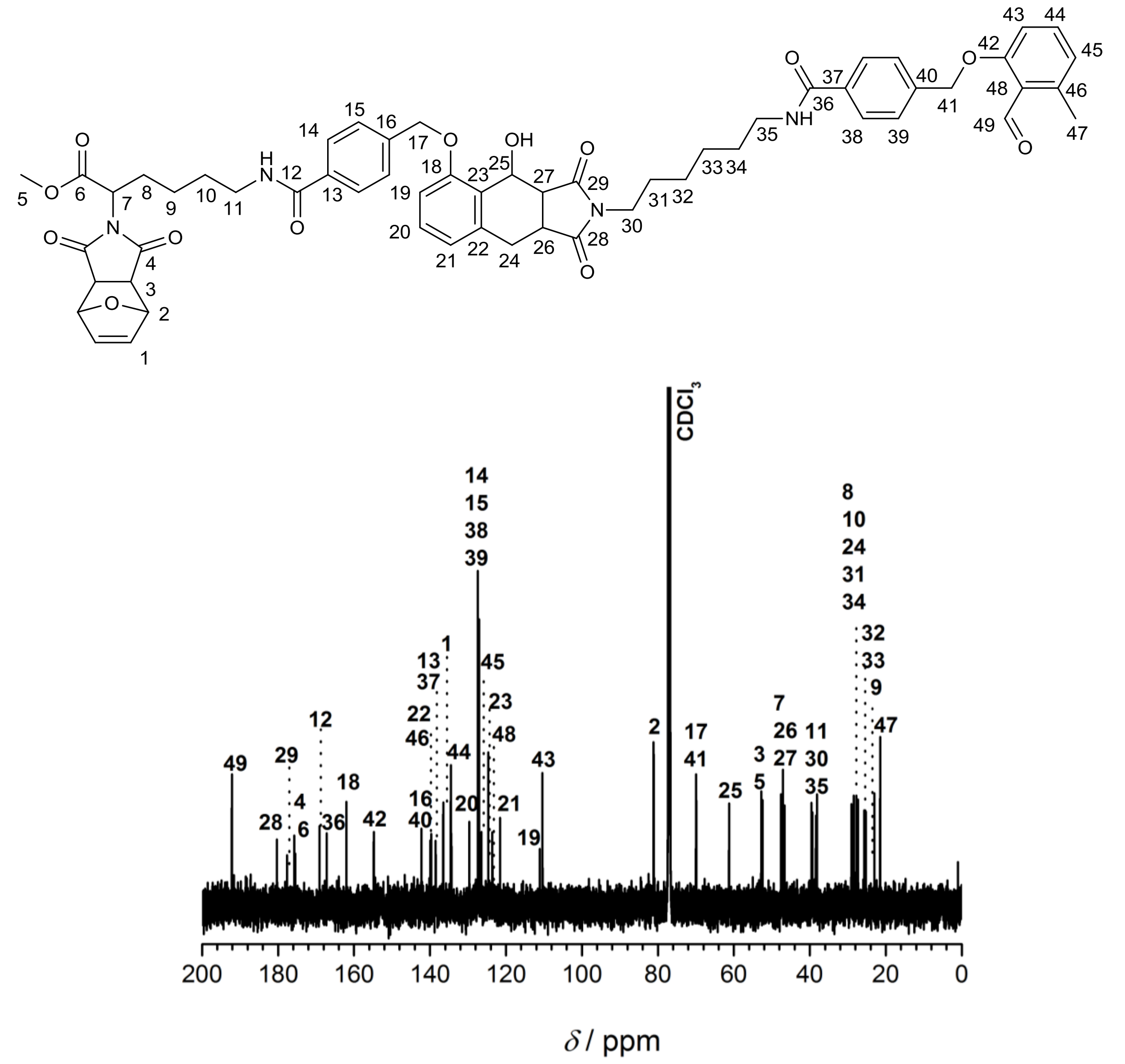

Supplementary Figure 108. ^{13}C NMR spectrum of **10d** ($CDCl_3$).

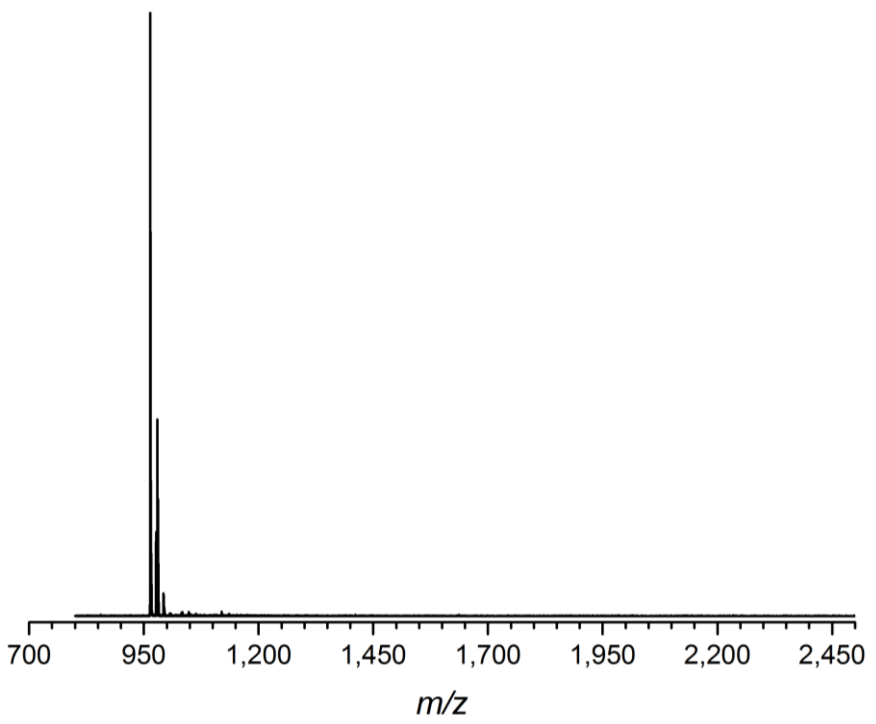

Supplementary Figure 109. MALDI–ToF overview spectrum of **10d**. All major peaks belong to the target molecule with different counter ions. All peak assignments can be found in Supplementary Table 21.

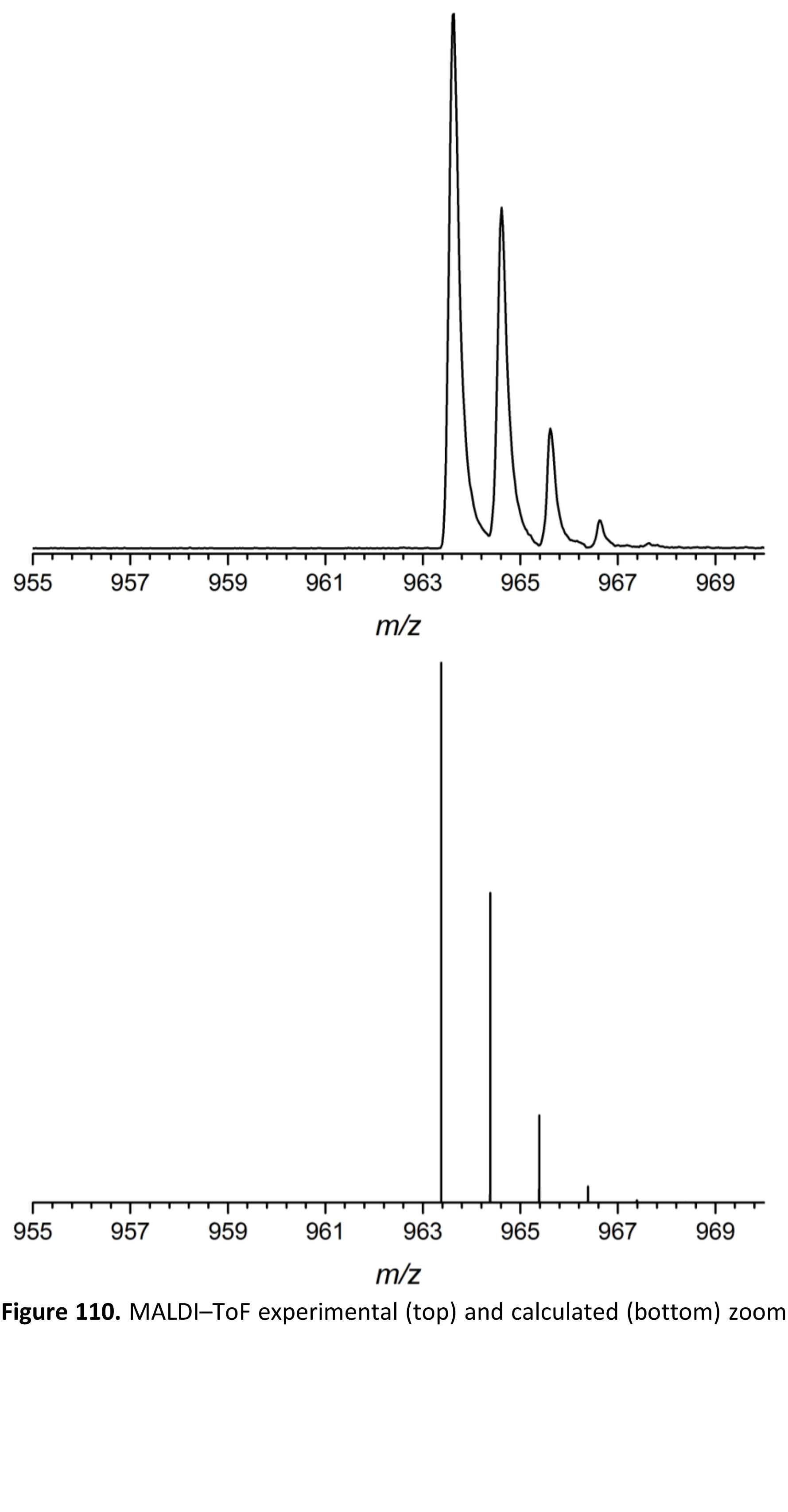

Supplementary Figure 110. MALDI–ToF experimental (top) and calculated (bottom) zoom spectra of **10d**.

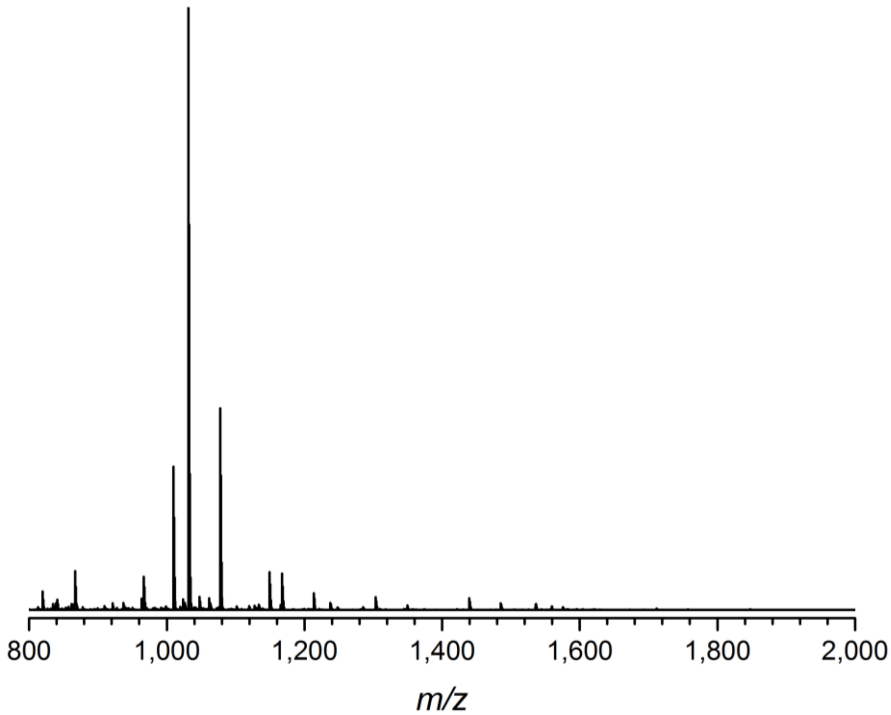

Supplementary Figure 111. ESI-MS overview spectrum of **10d**. All major peaks belong to the target molecule with different counter ions. All peak assignments can be found in Supplementary Table 21.

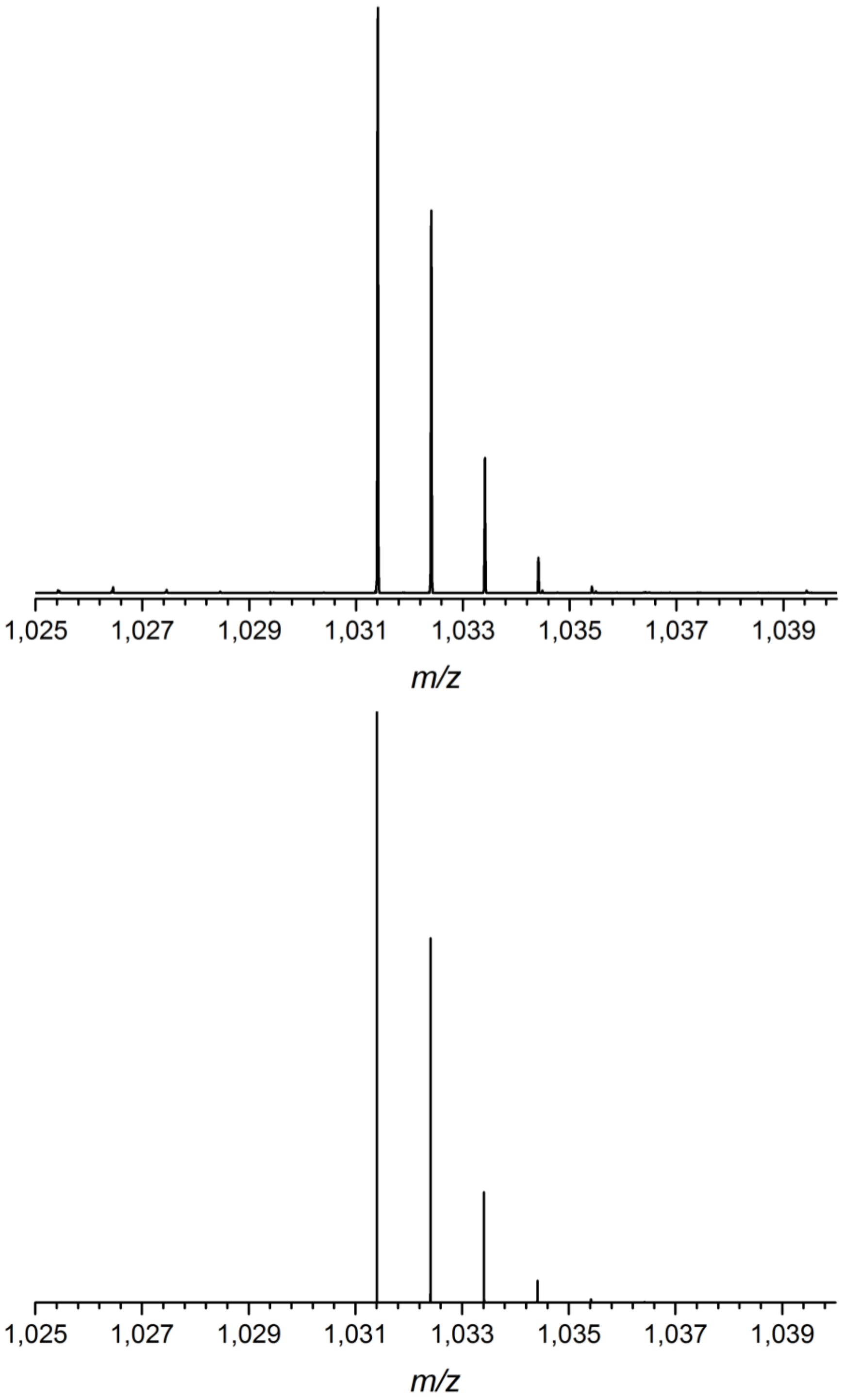

Supplementary Figure 112. ESI-MS experimental (top) and calculated (bottom) zoom spectra of **10d**.

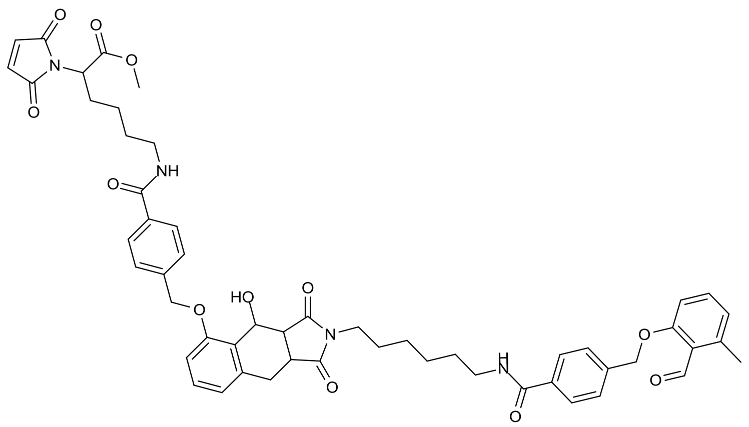

A_1

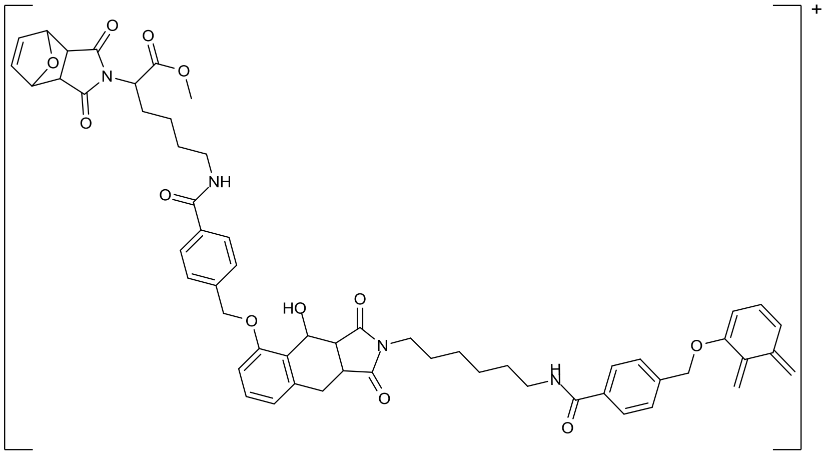

A_2^+

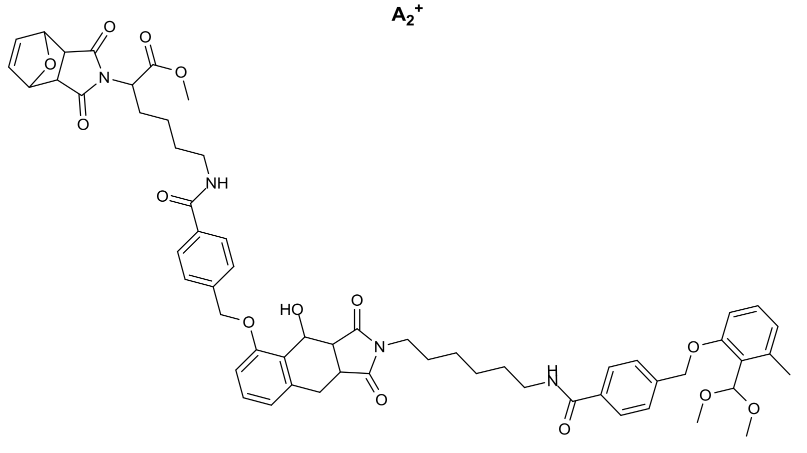

A_3

Supplementary Figure 113. Collation of molecules identified in Supplementary Table 21 (**A_1**-**A_3**).

Characterization of 10 $(M_2)(M_1M_2)_2$-X-$(M_2M_1)_2(M_2)$

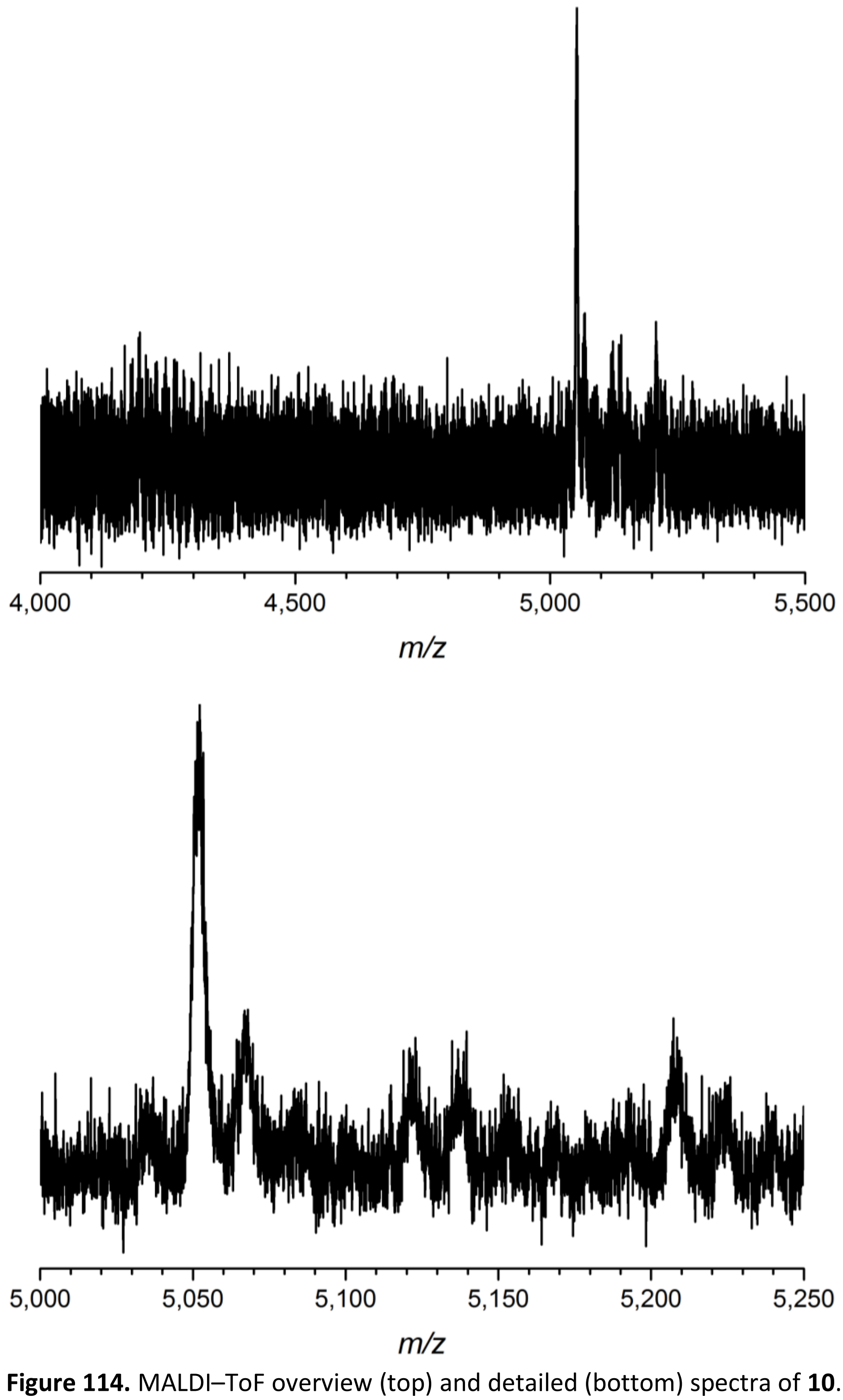

Supplementary Figure 114. MALDI–ToF overview (top) and detailed (bottom) spectra of **10**. All major peaks belong to the target molecule with different counter ions. All peak assignments can be found in Supplementary Table 22.

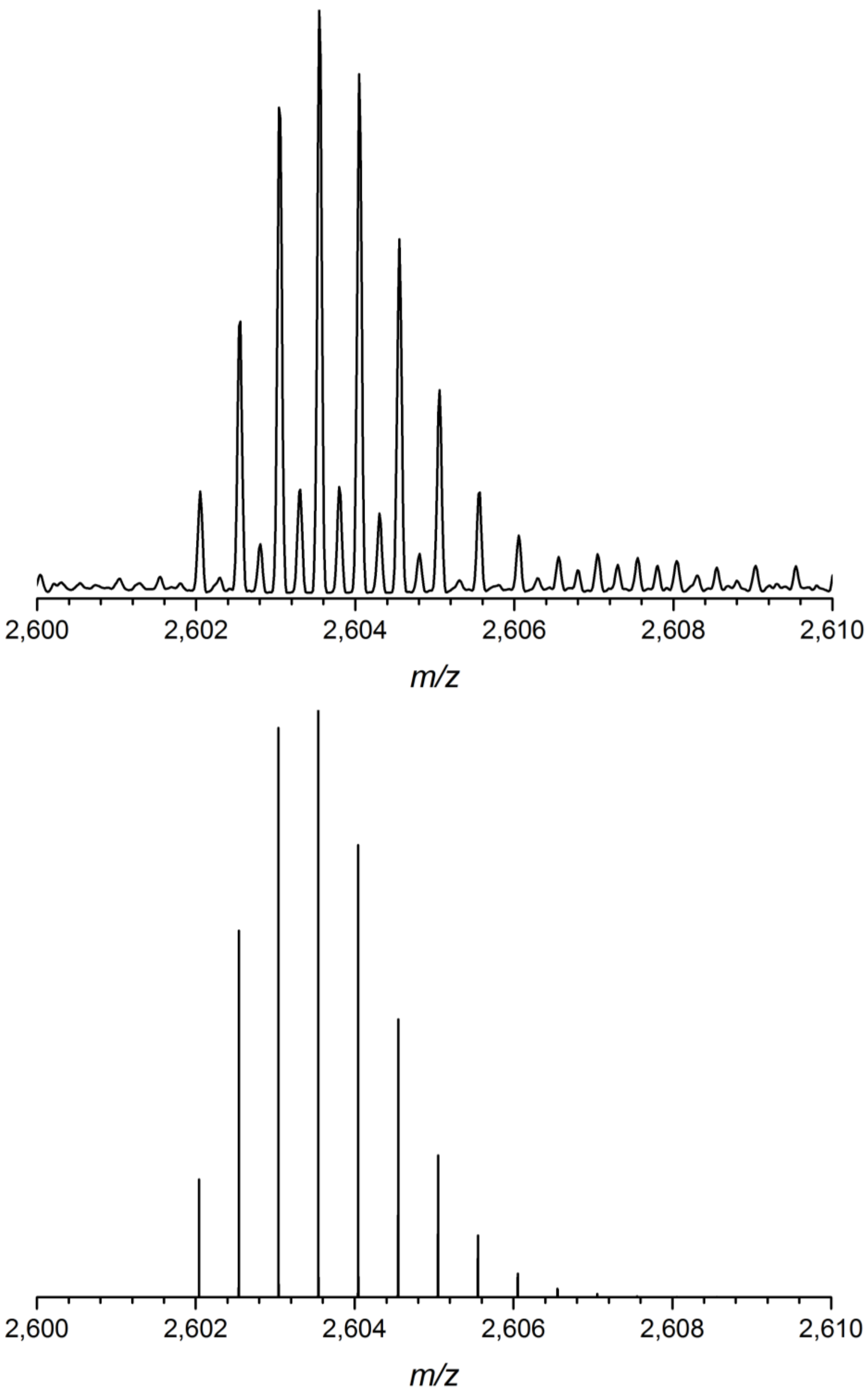

Supplementary Figure 115. ESI-MS experimental (top) and calculated (bottom) zoom spectra of **10**. The exact mass of the measured sample is matching with the assigned species (see Supplementary Table 22), however there is likely an additional signal from a double charged cluster of two molecules.

Characterization of symmetric Copolymer 11 (($M_6M_5M_4M_2$)-X-($M_2M_4M_5M_6$)) and precursors 11a-b (11a: (M_4M_2)-X-(M_2M_4), 11b: (M_5M_6))

Characterization of 11a (M_4M_2)-X-(M_2M_4)

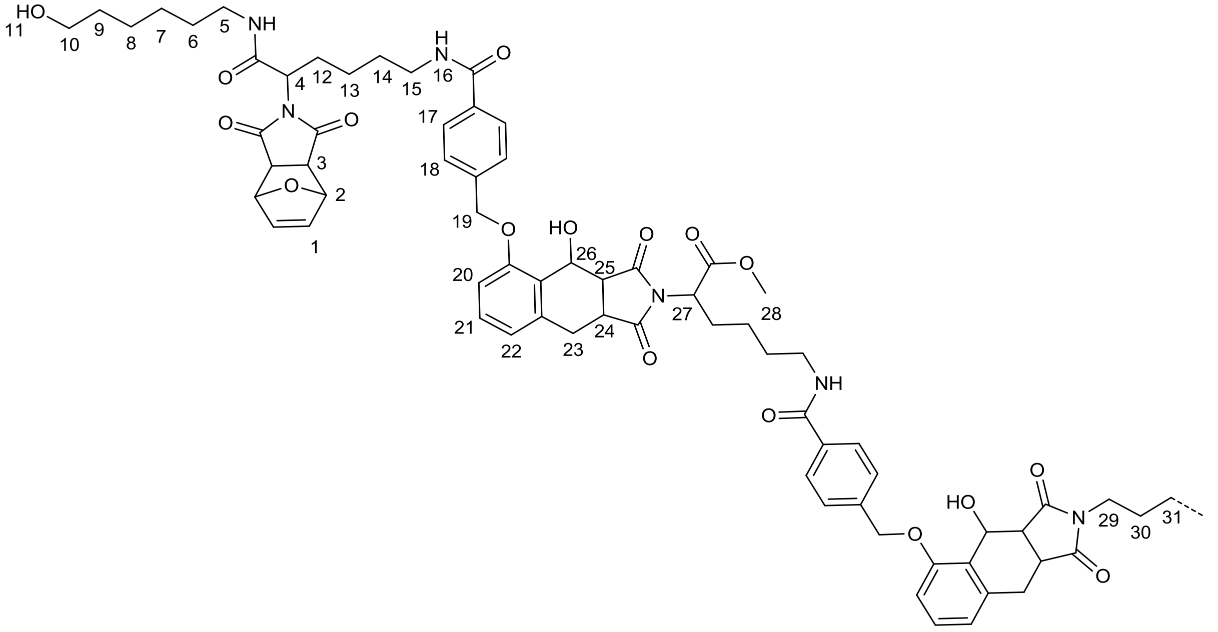

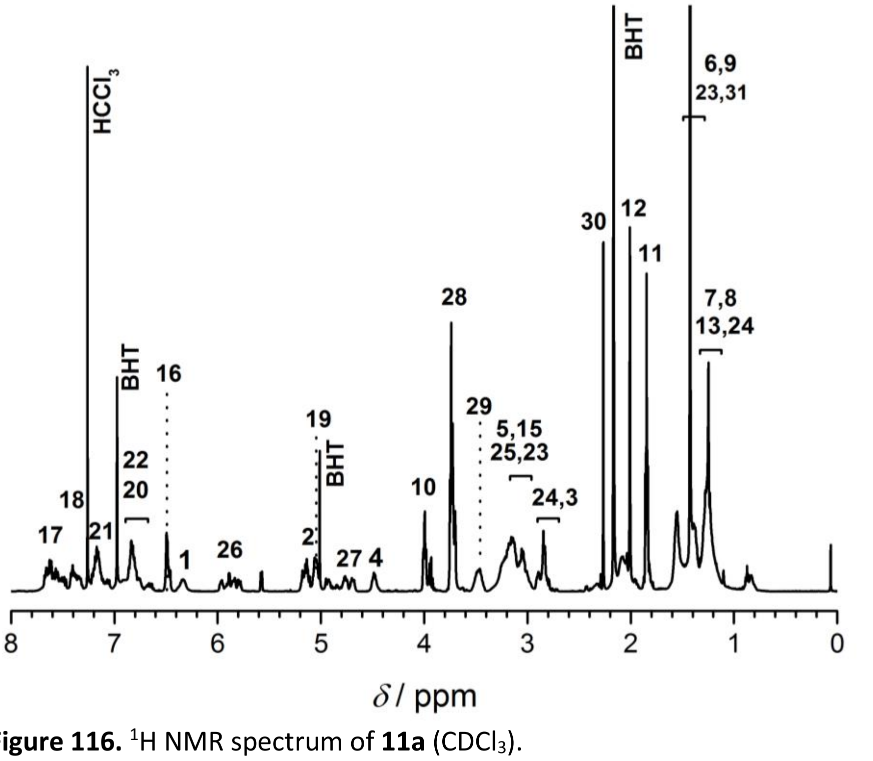

Supplementary Figure 116. ^{1}H NMR spectrum of **11a** ($CDCl_3$).

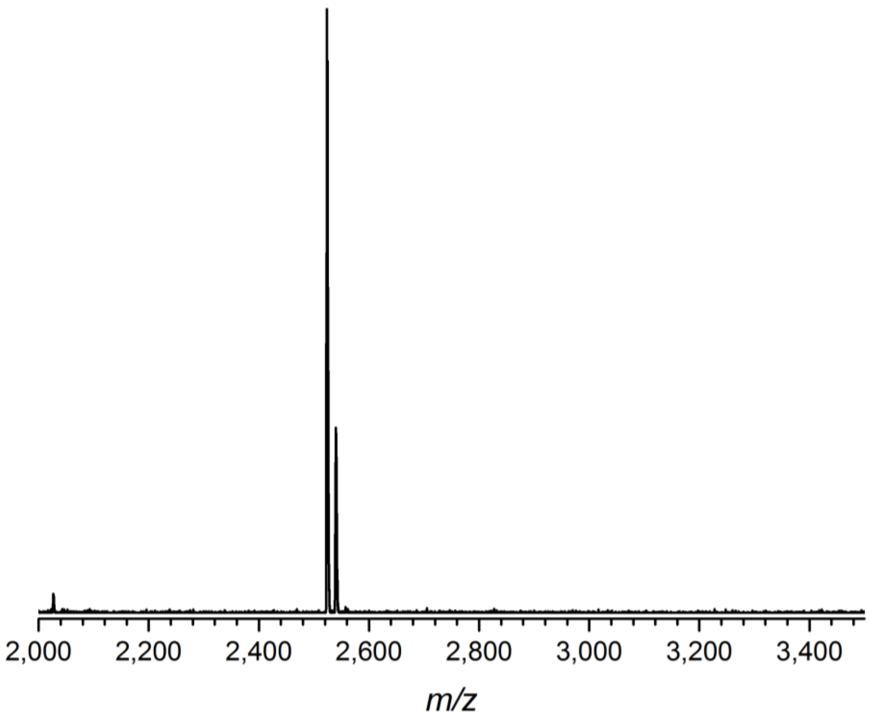

Supplementary Figure 117. MALDI–ToF overview spectrum of **11a**. All major peaks belong to the target molecule with different counter ions. All peak assignments can be found in Supplementary Table 23.

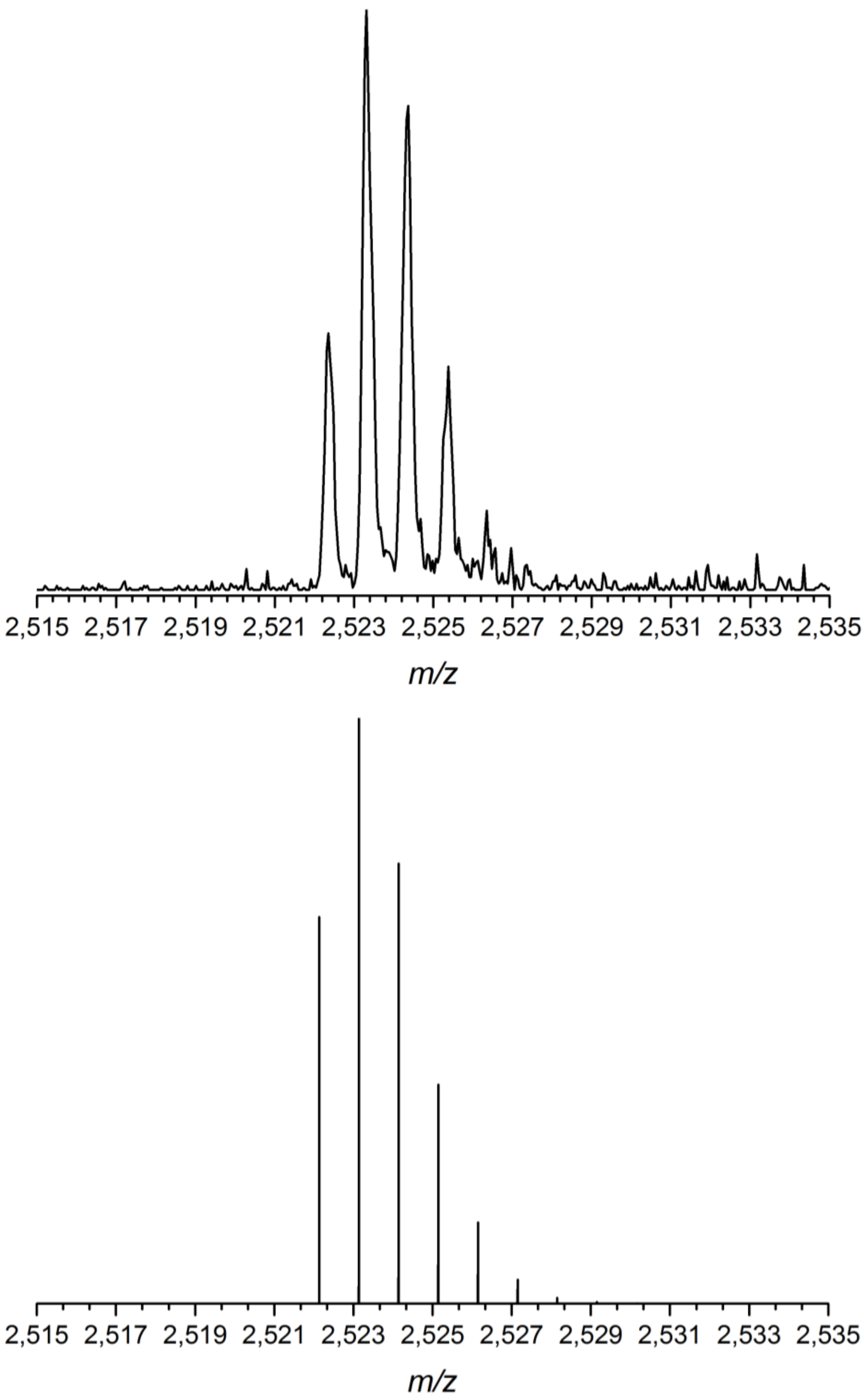

Supplementary Figure 118. MALDI–ToF experimental (top) and calculated (bottom) zoom spectra of **11a**.

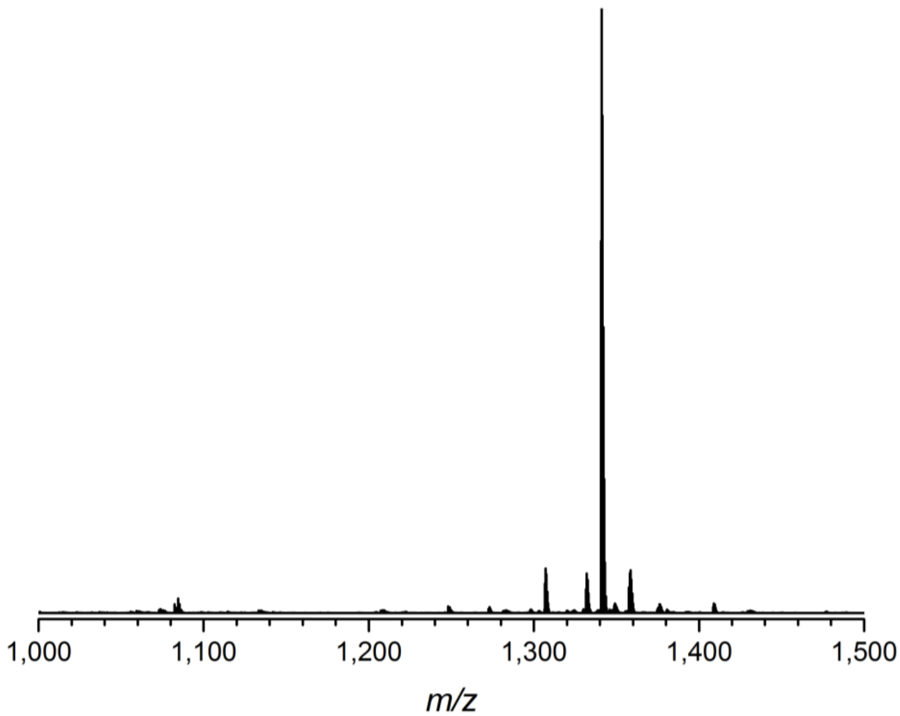

Supplementary Figure 119. ESI-MS overview spectrum of **11a**. All major peaks belong to the target molecule with different counter ions. All peak assignments can be found in Supplementary Table 23.

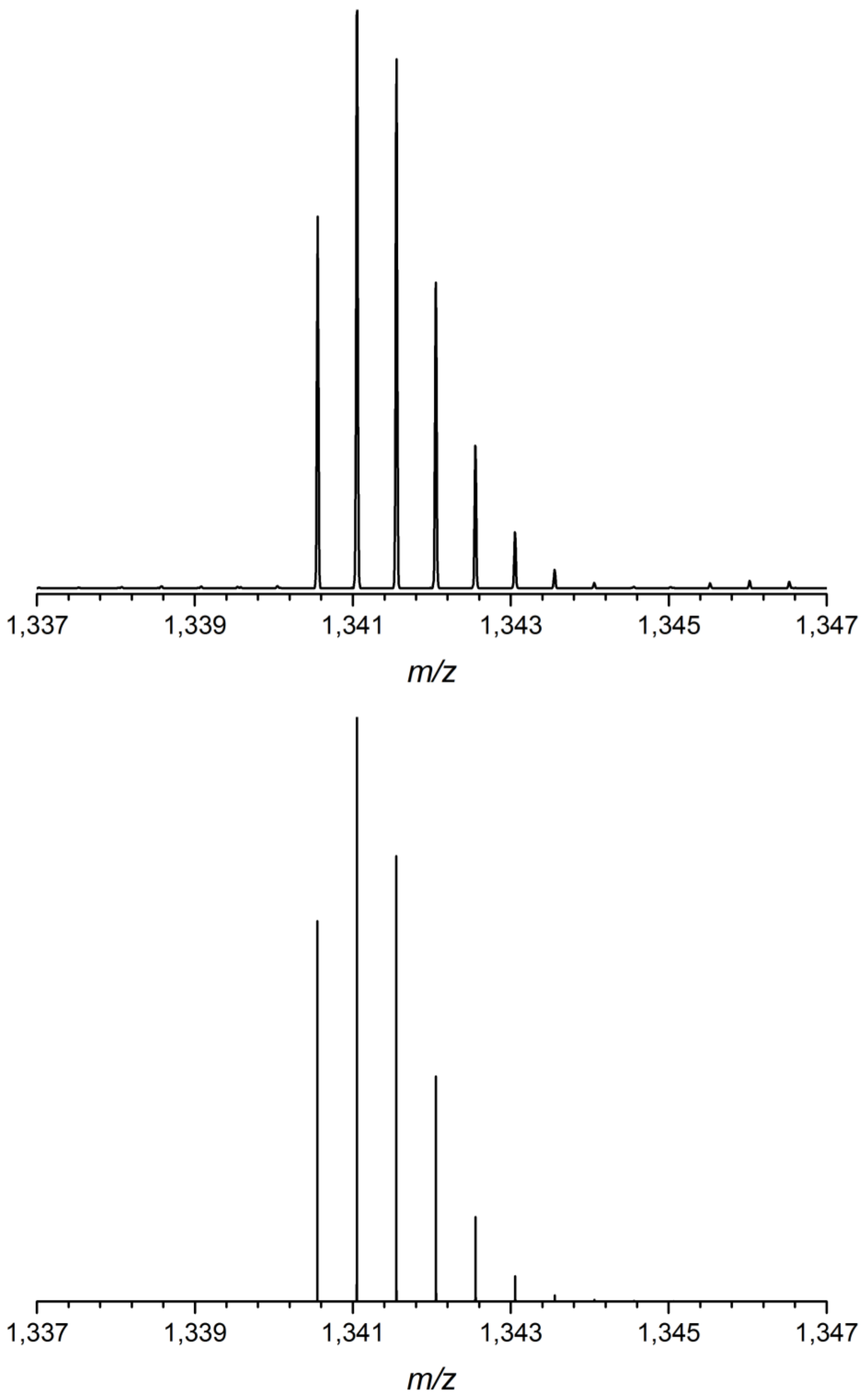

Supplementary Figure 120. ESI-MS experimental (top) and calculated (bottom) zoom spectra of **11a**.

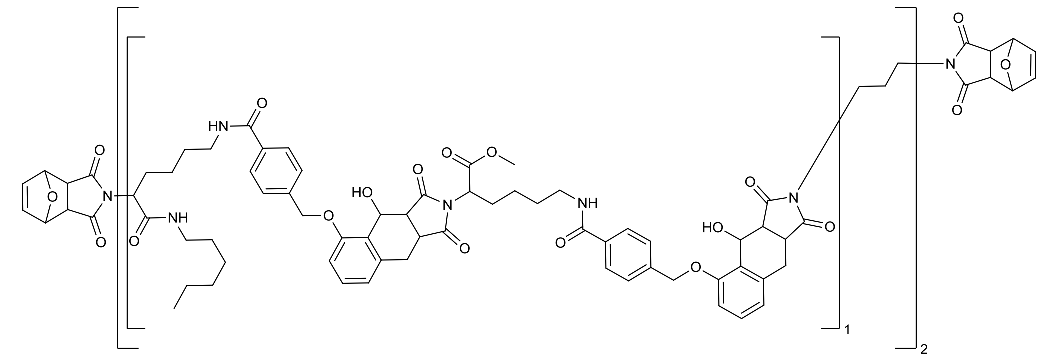

A$_1$

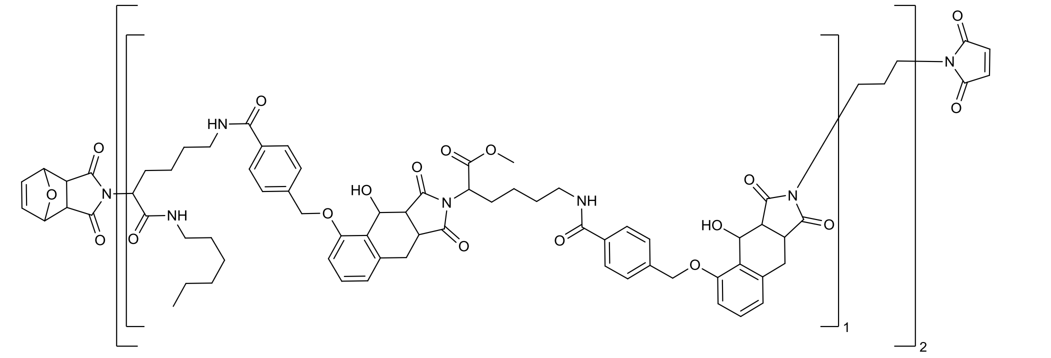

A$_2$

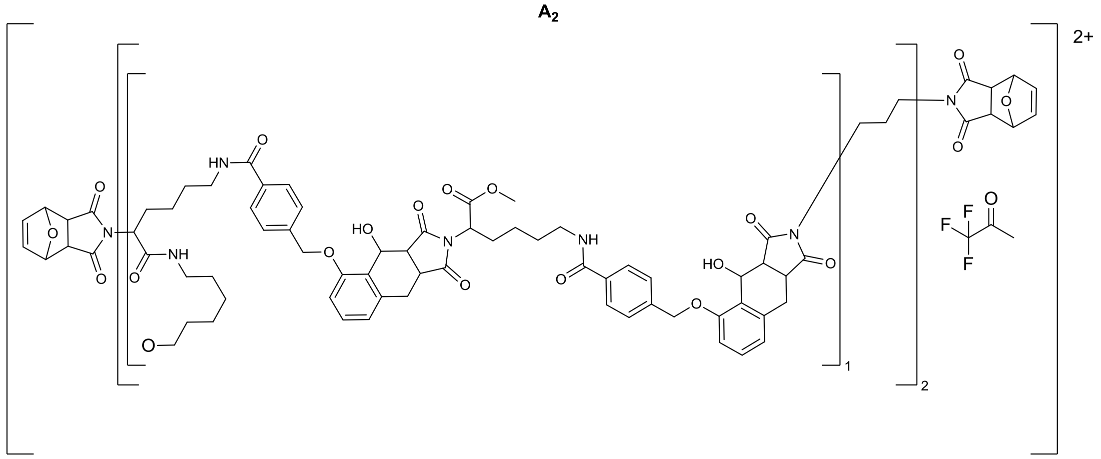

A$_3$

Supplementary Figure 121. Collation of molecules identified in Supplementary Table 23 (**A$_1$**-**A$_3$**).

Characterization of 11b (M_6M_5)

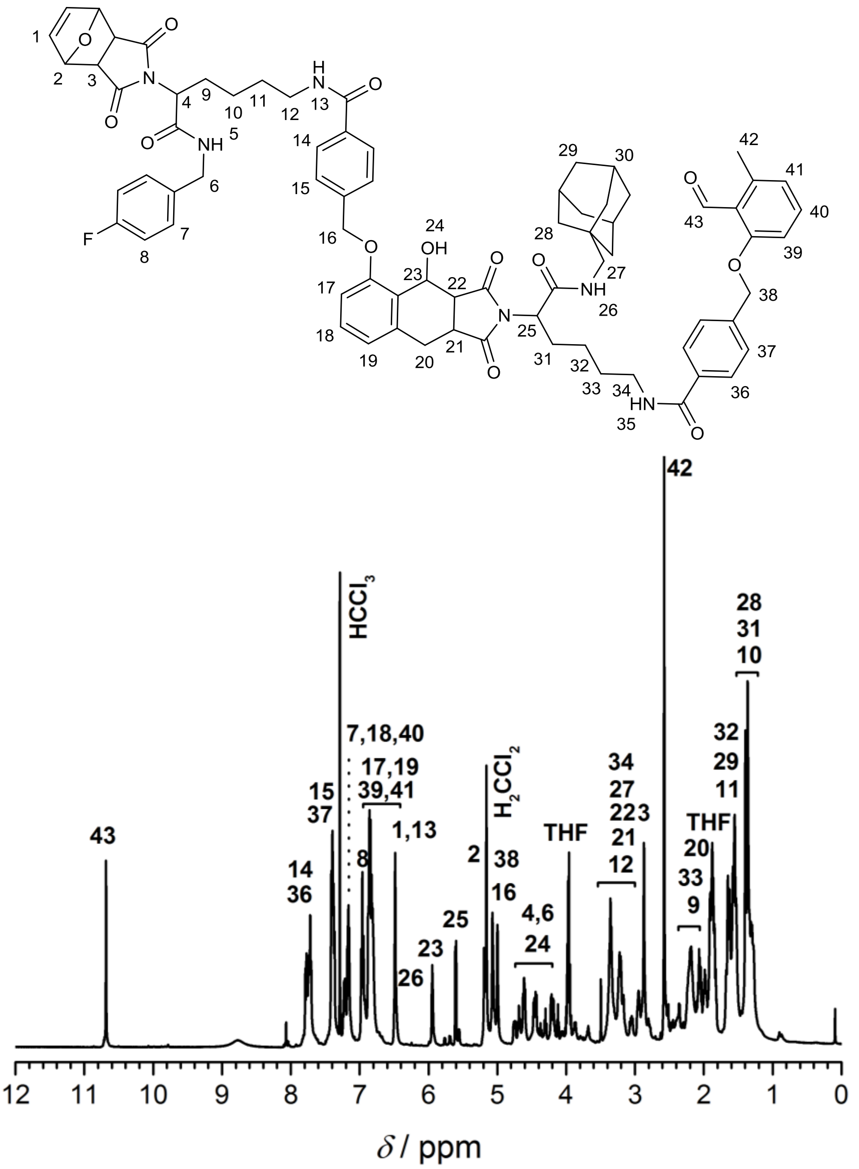

Supplementary Figure 122. ^{1}H NMR spectrum of **11b** ($CDCl_3$).

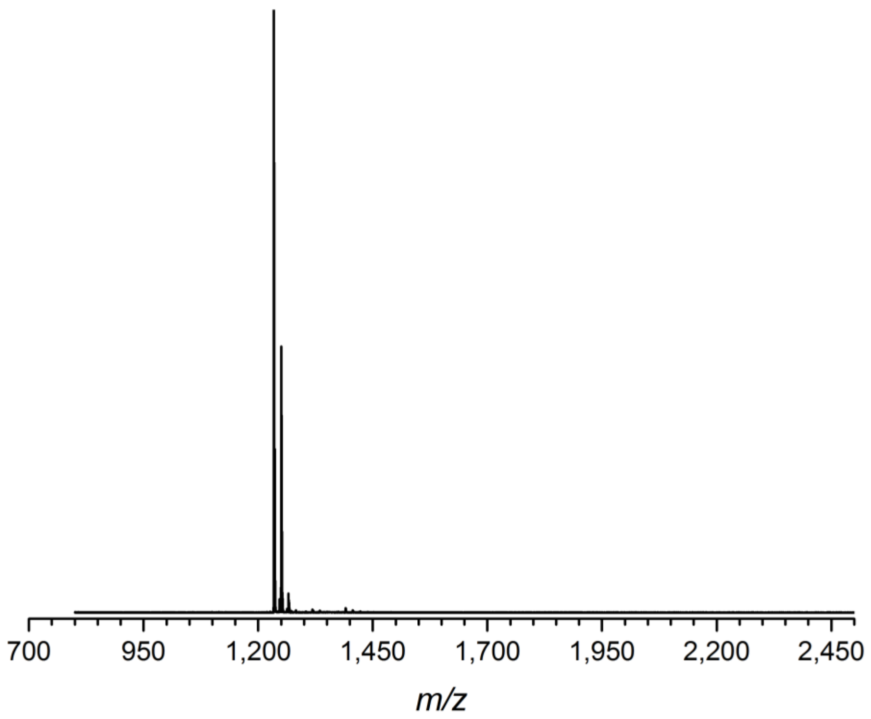

Supplementary Figure 123. MALDI–ToF overview spectrum of **11b**. All major peaks belong to the target molecule with different counter ions. All peak assignments can be found in Supplementary Table 24.

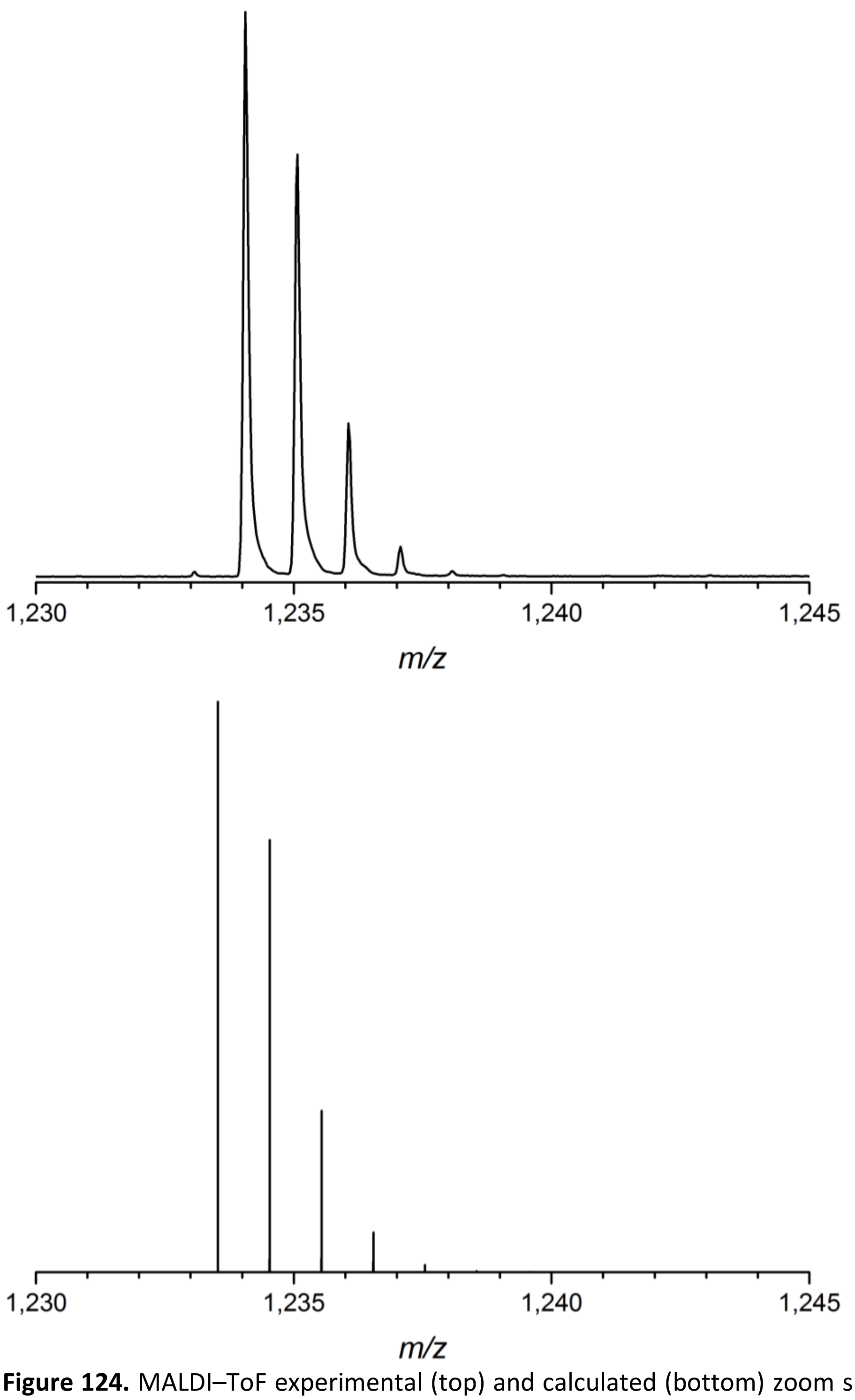

Supplementary Figure 124. MALDI–ToF experimental (top) and calculated (bottom) zoom spectra of **11b**.

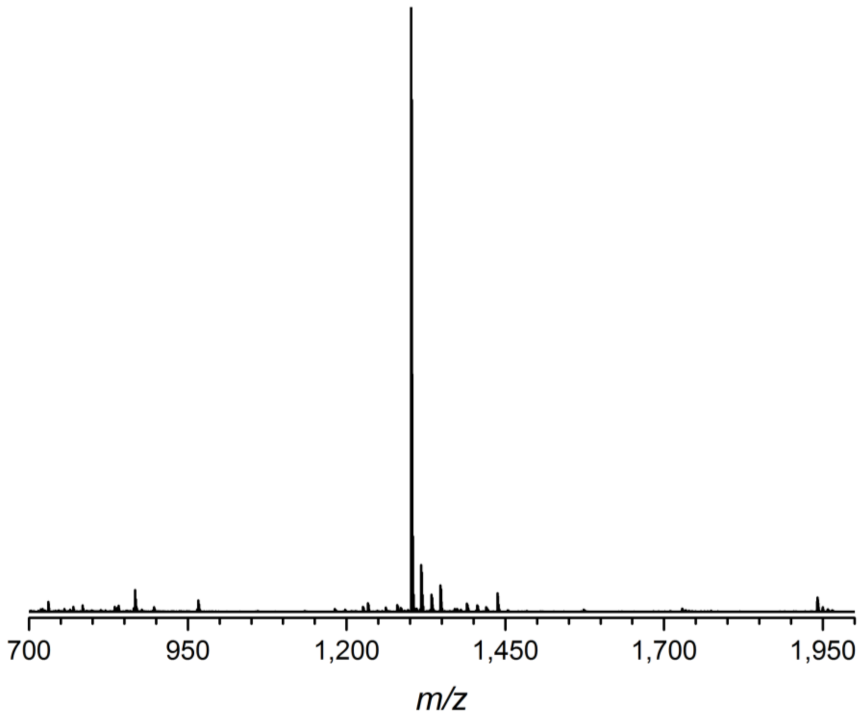

Supplementary Figure 125. ESI-MS overview spectrum of **11b**. All major peaks belong to the target molecule with different counter ions. All peak assignments can be found in Supplementary Table 24.

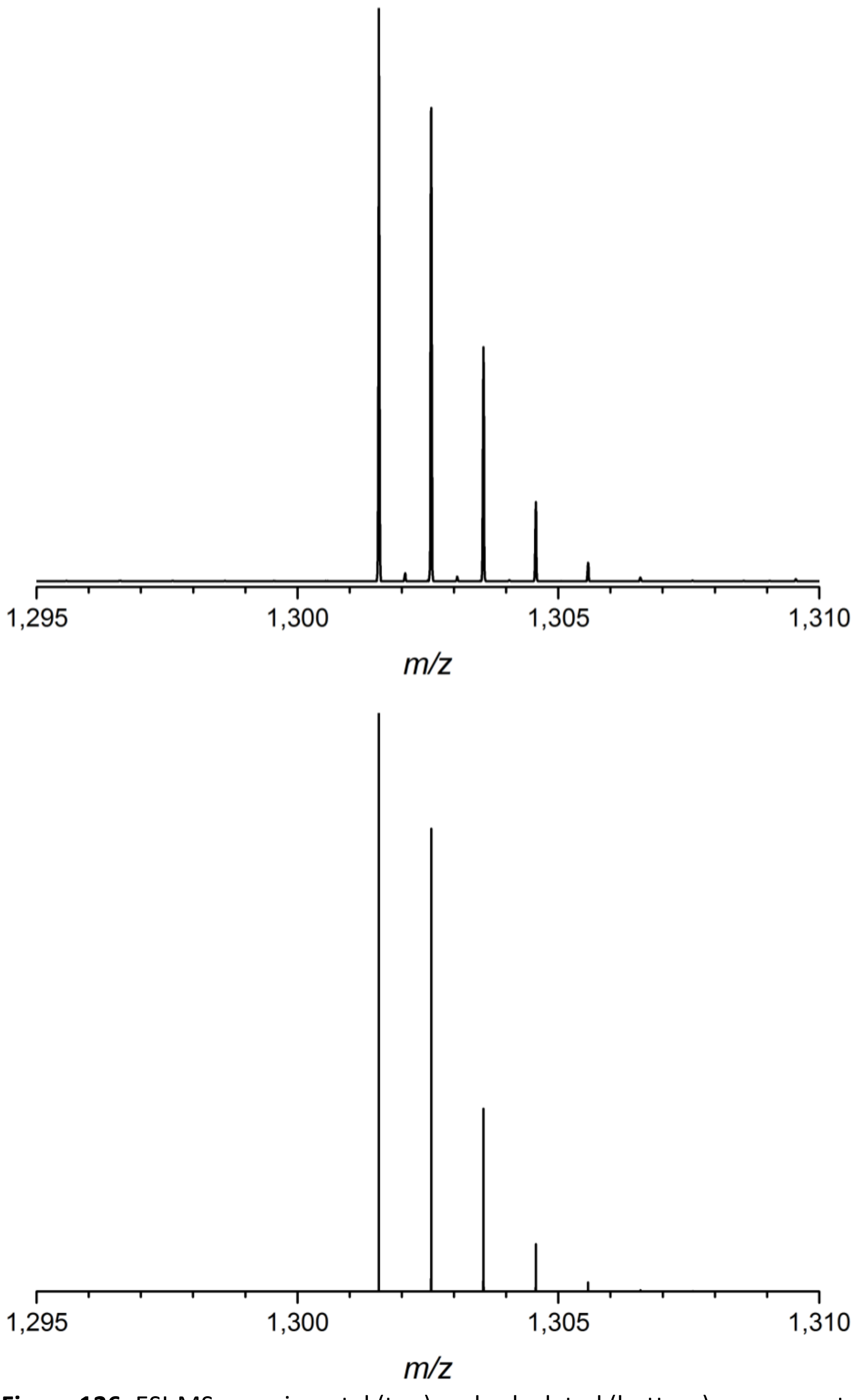

Supplementary Figure 126. ESI-MS experimental (top) and calculated (bottom) zoom spectra of **11b**.

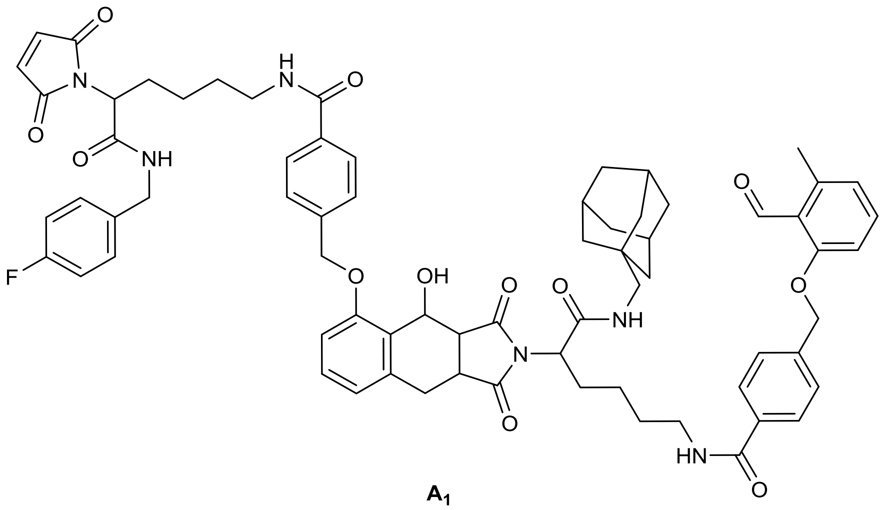

$\mathbf{A_1}$

Supplementary Figure 127. Collation of molecules identified in Supplementary Table 24 ($\mathbf{A_1}$).

Characterization of 11 ($M_6M_5M_4M_2$)-X-($M_2M_4M_5M_6$)

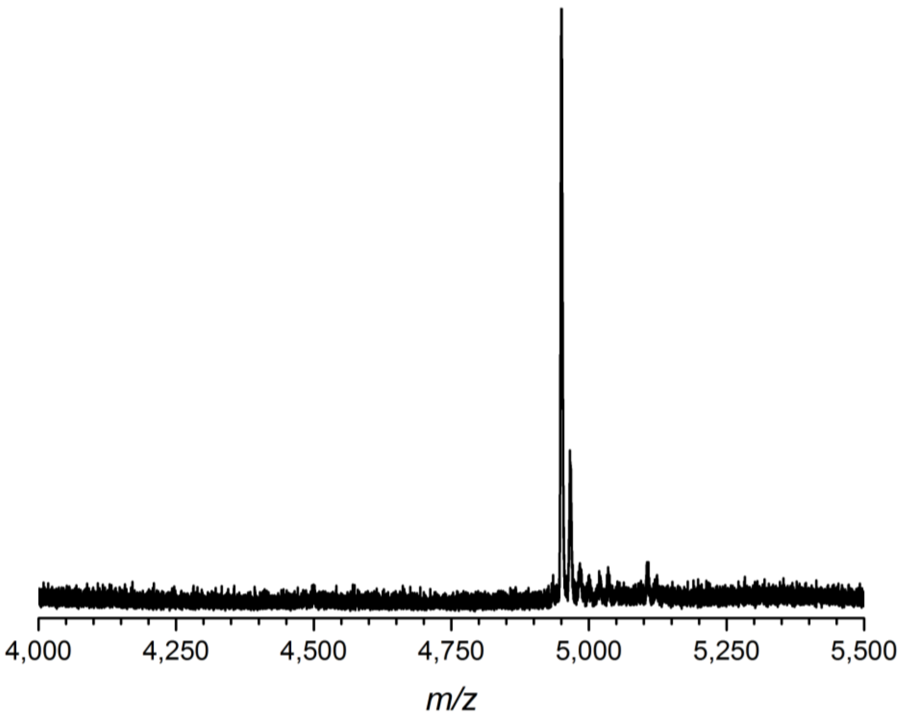

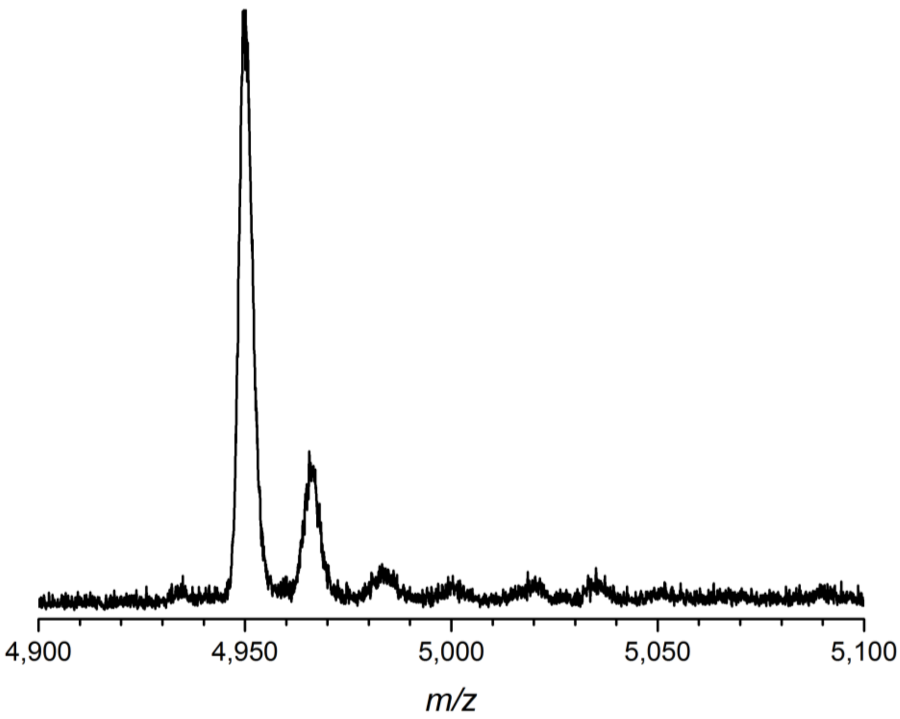

Supplementary Figure 128. MALDI–ToF overview (top) and detailed (bottom) spectrum of **11**. All major peaks belong to the target molecule with different counter ions. All peak assignments can be found in Supplementary Table 25.

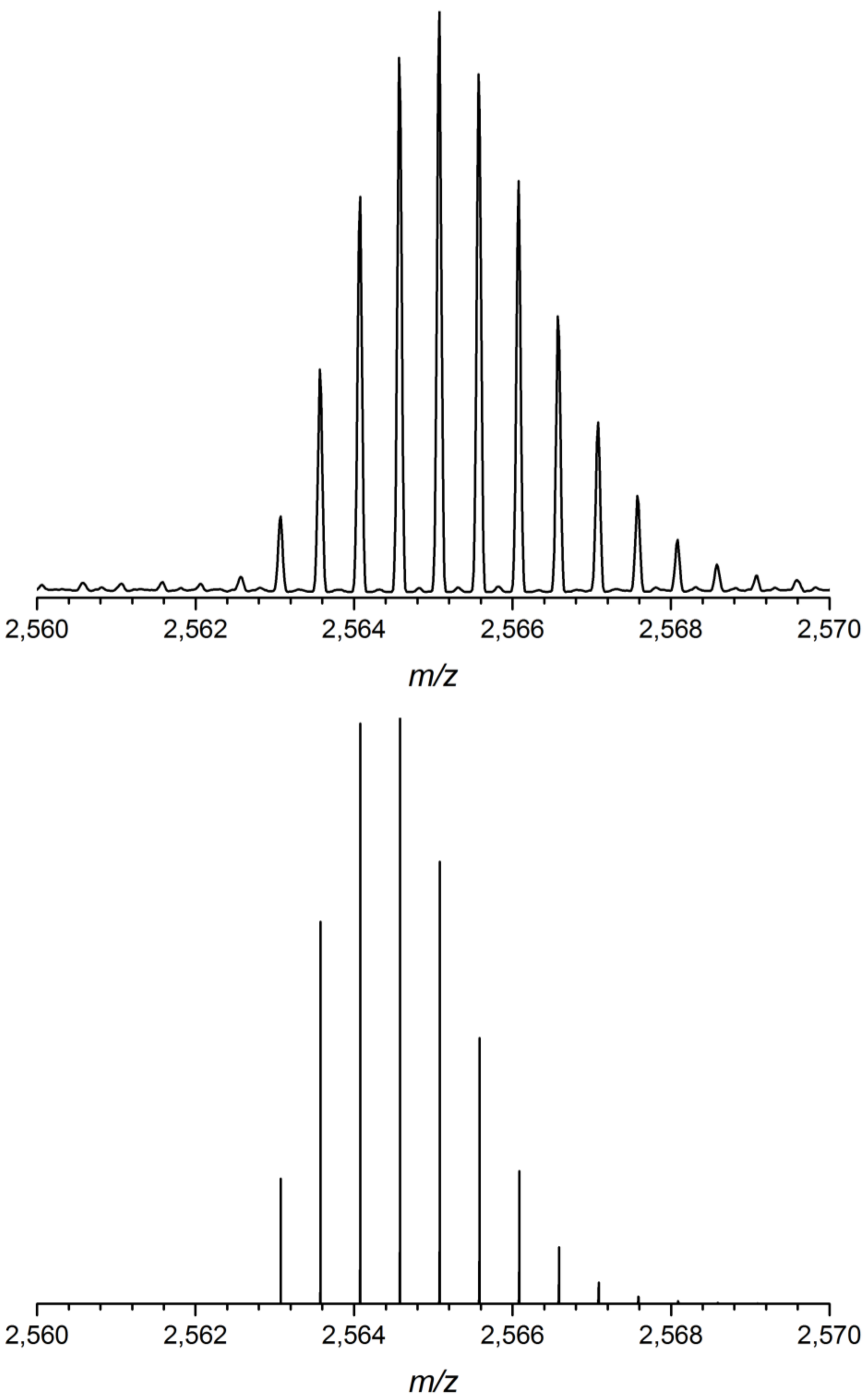

Supplementary Figure 129. ESI-MS experimental (top) and calculated (bottom) zoom spectra of **11**. The exact mass of the measured sample is matching with the assigned species (see Supplementary Table 25), however there is likely an additional species ionized with one additional proton.

Characterization of Homopolymer 12 (($(M_3)_5$) and precursors 12a-b (12a: $(M_3)_2$, 12b: $(M_3)_3$)

Characterization of 12a $(M_3)_2$

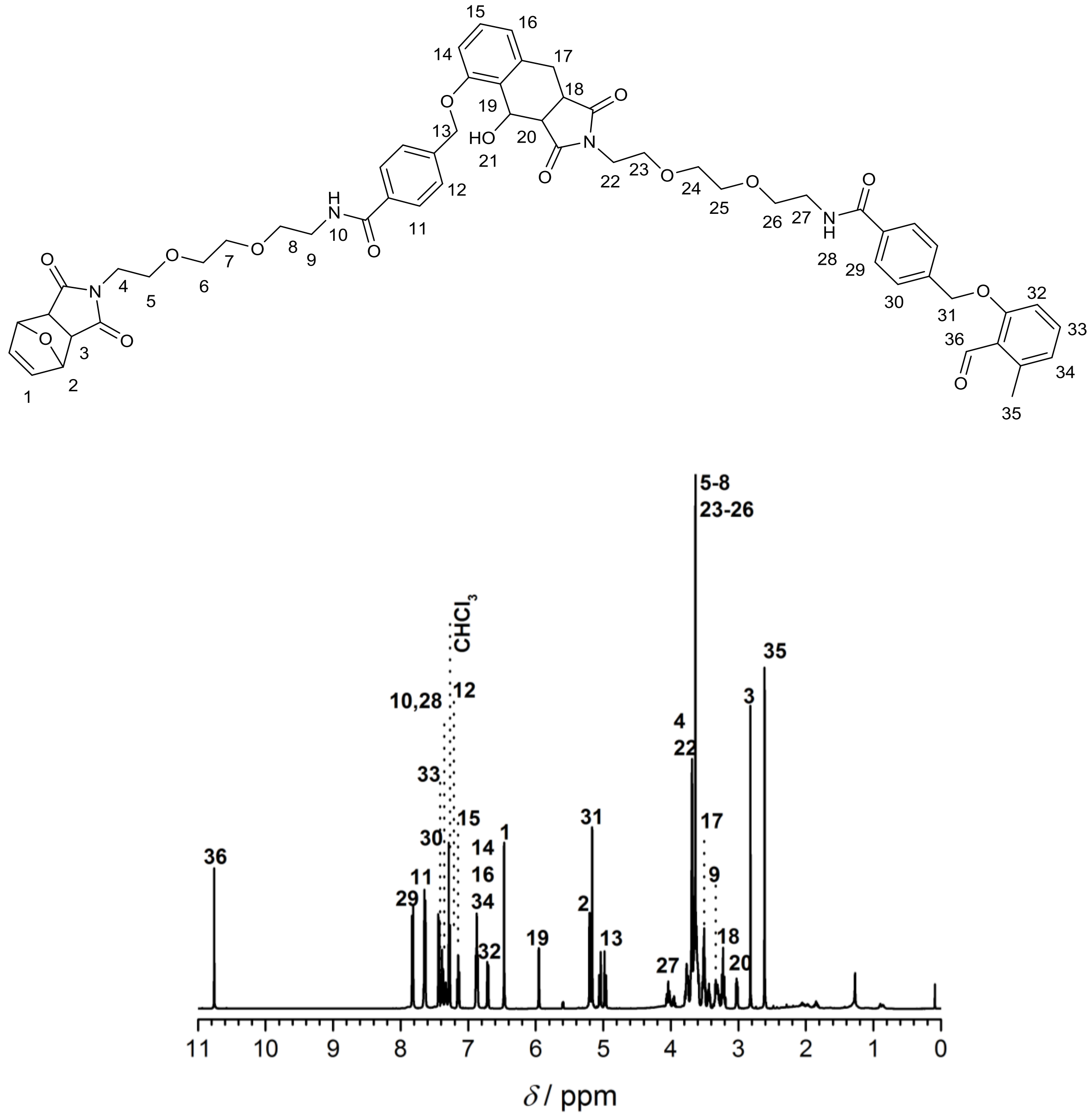

Supplementary Figure 130. ^{1}H NMR spectrum of **12a** ($CDCl_3$).

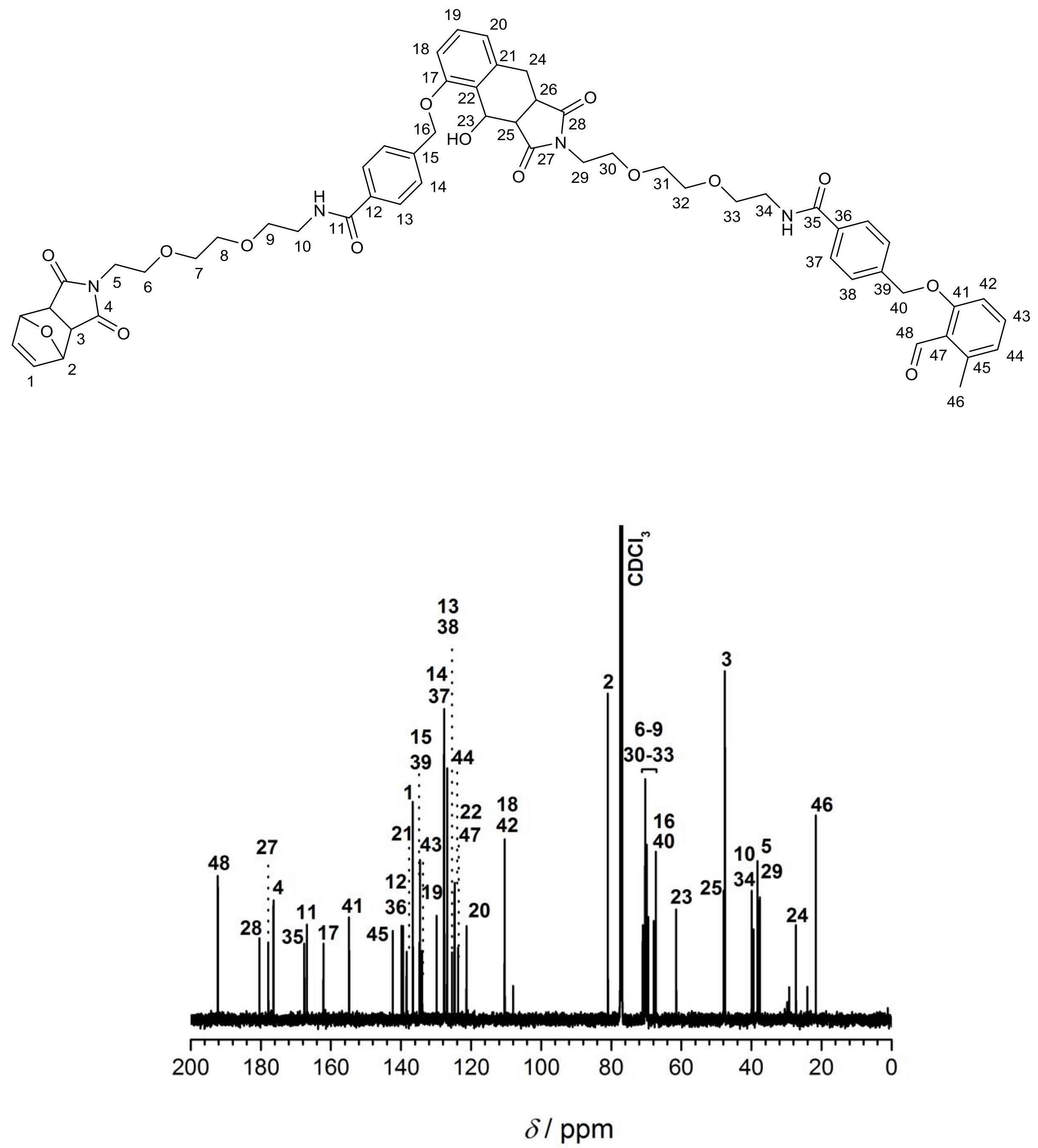

Supplementary Figure 131. ^{13}C NMR spectrum of **12a** ($CDCl_3$).

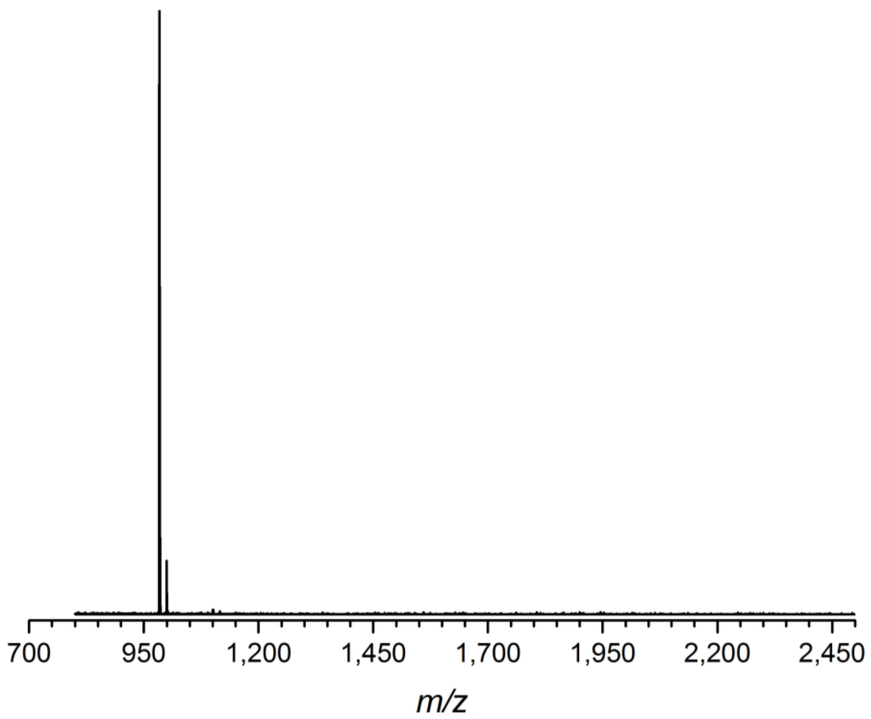

Supplementary Figure 132. MALDI–ToF overview spectrum of **12a**. All major peaks belong to the target molecule with different counter ions. All peak assignments can be found in Supplementary Table 26.

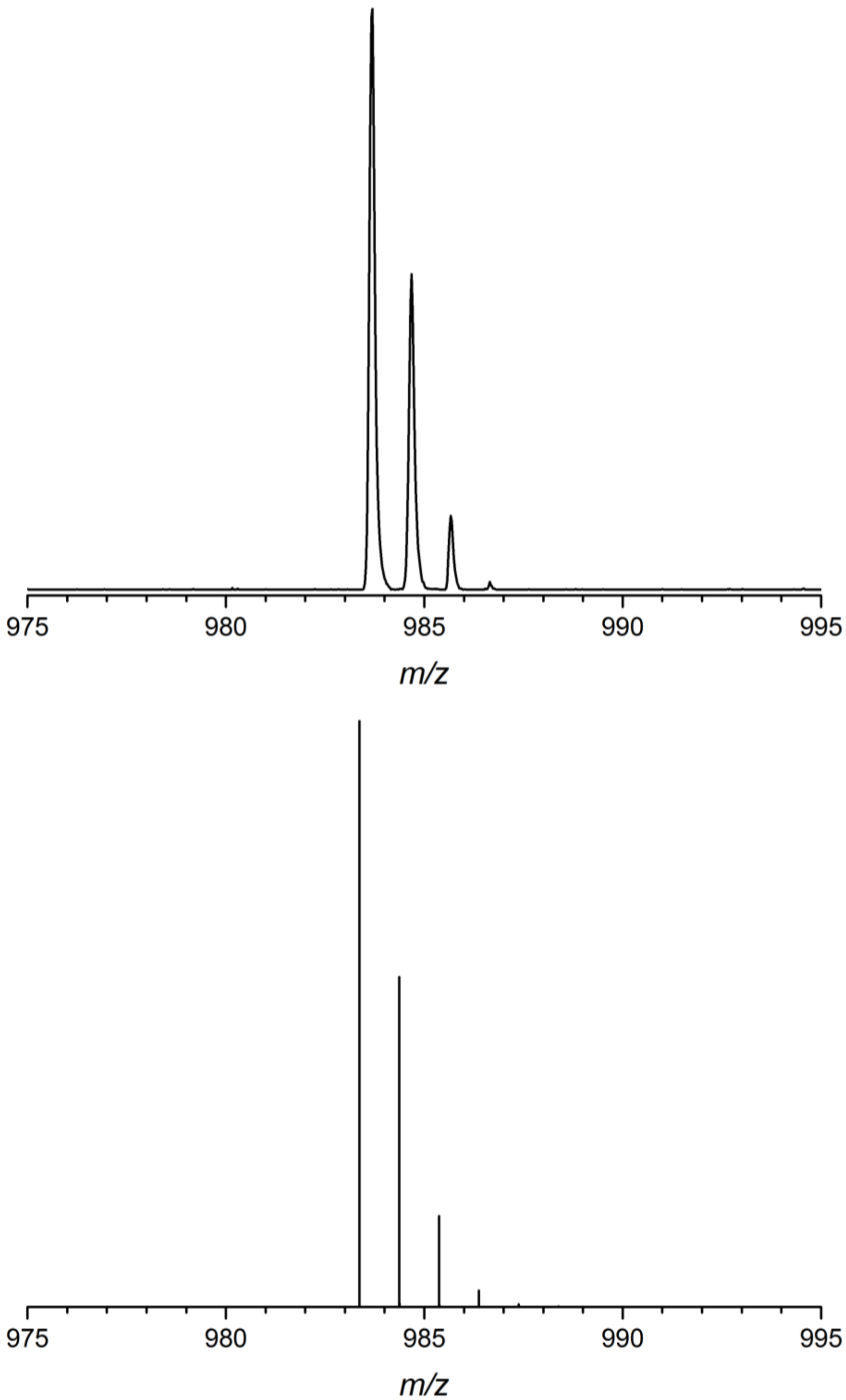

Supplementary Figure 133. MALDI–ToF experimental (top) and calculated (bottom) zoom spectra of **12a**.

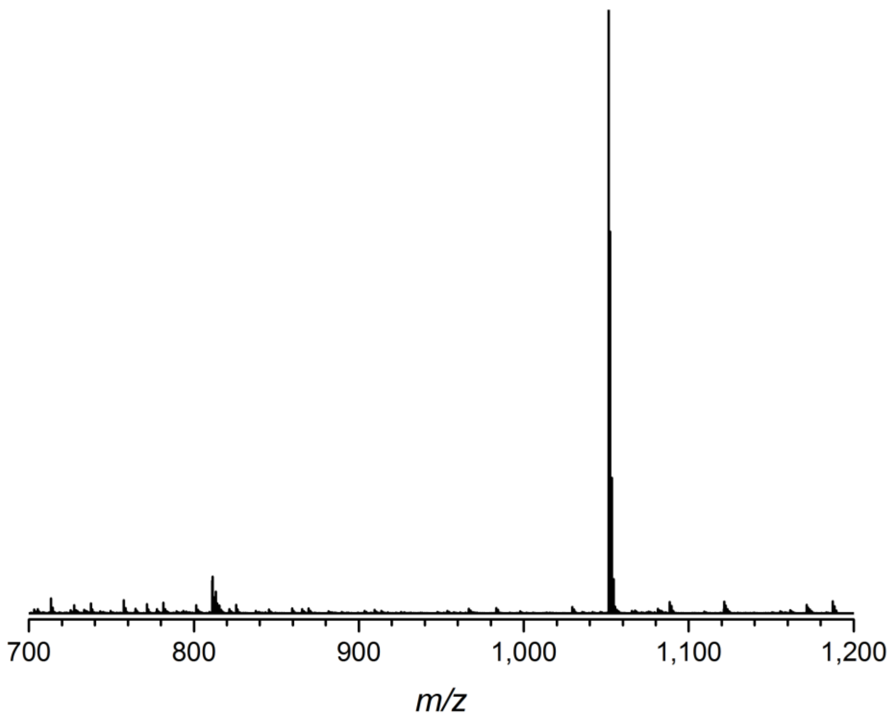

Supplementary Figure 134. ESI-MS overview spectrum of **12a**. All peaks belong to the target molecule with a different counter ion. All peak assignments can be found in Supplementary Table 26.

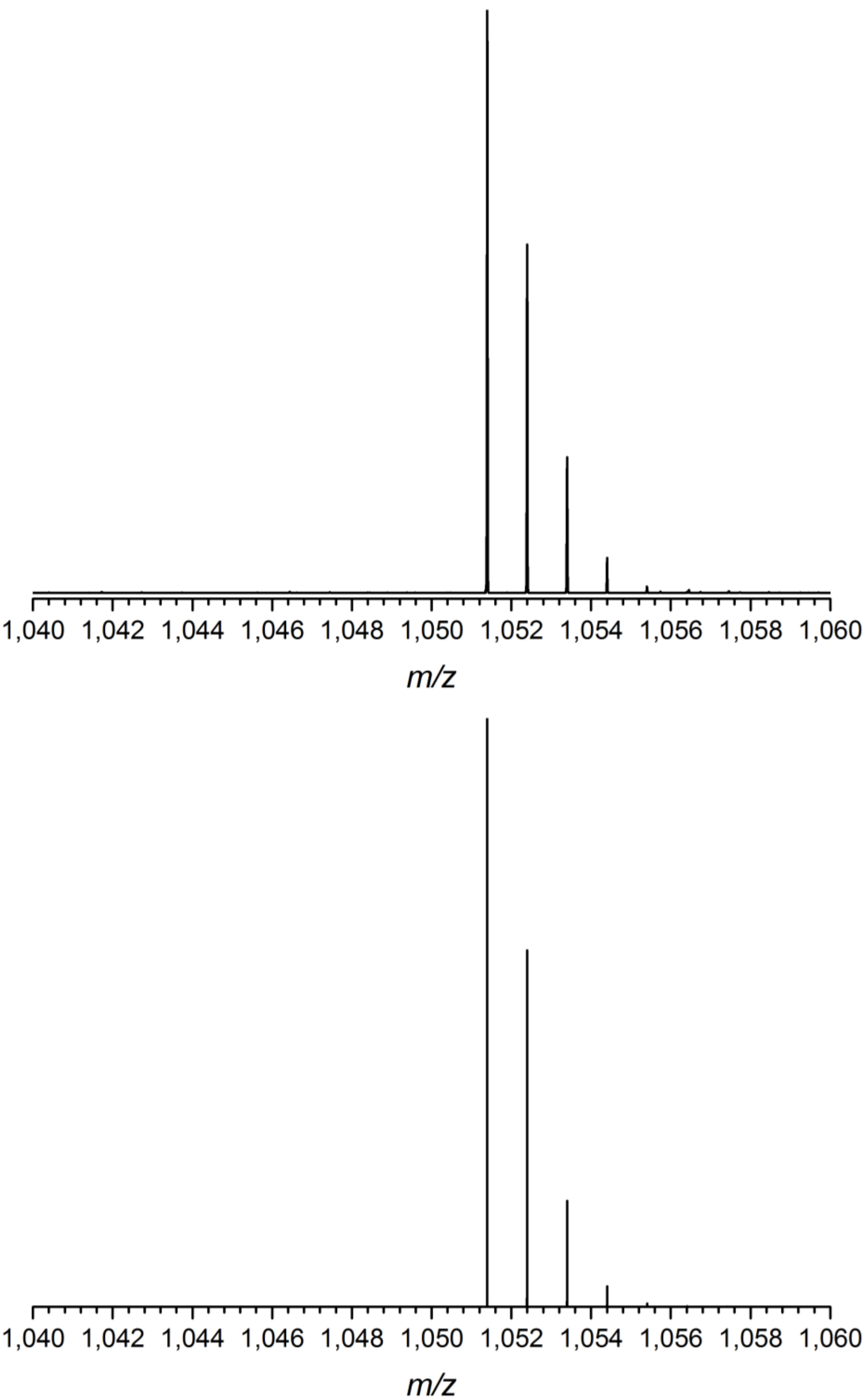

Supplementary Figure 135. ESI-MS experimental (top) and calculated (bottom) zoom spectra of **12a**.

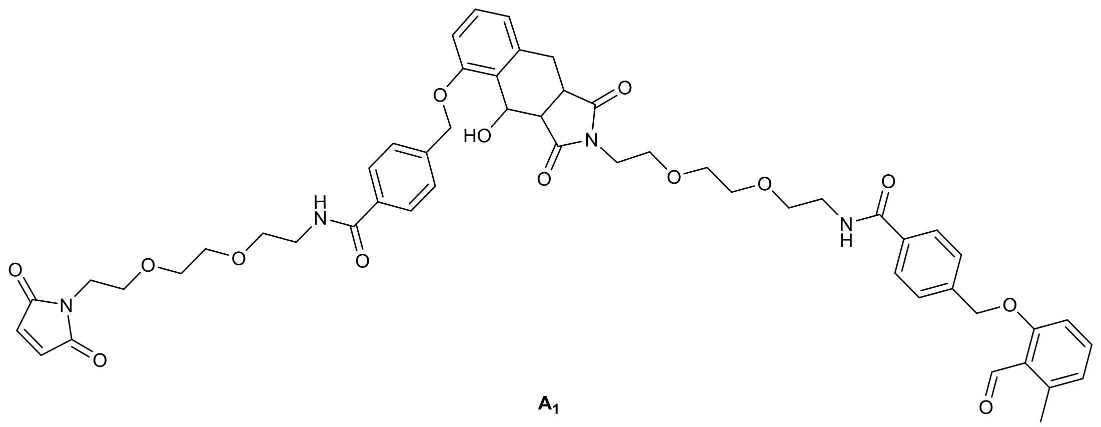

A_1

Supplementary Figure 136. Collation of molecules identified in Supplementary Table 26 (**A_1**).

Characterization of 12b (M$_3$)$_3$

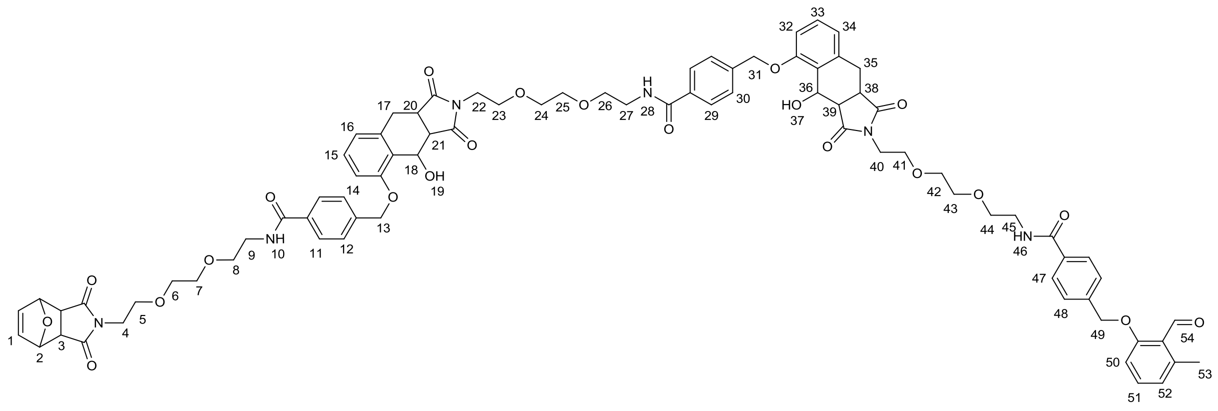

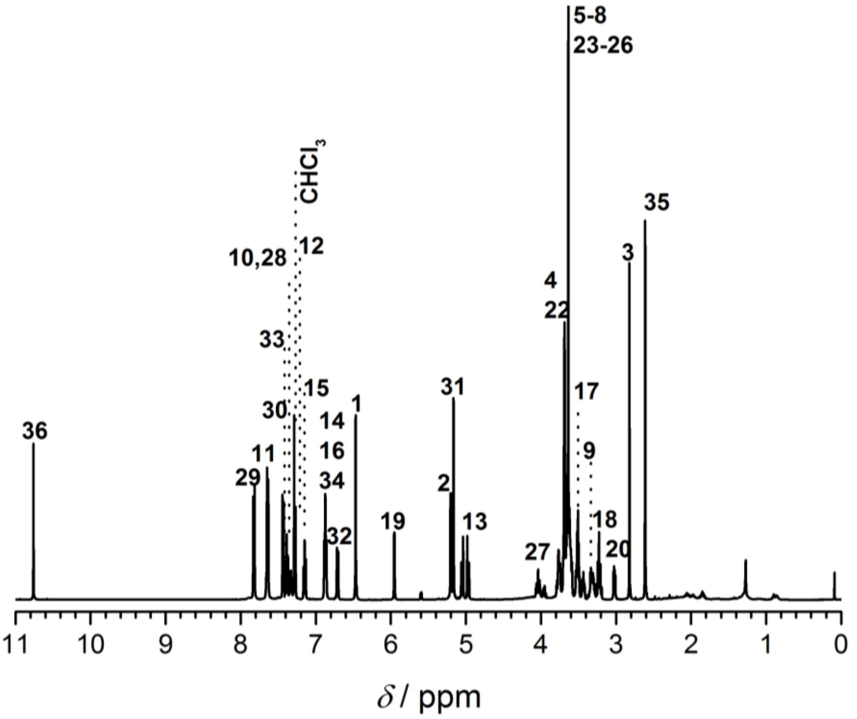

Supplementary Figure 137. ^{1}H NMR spectrum of **12b** ($CDCl_3$).

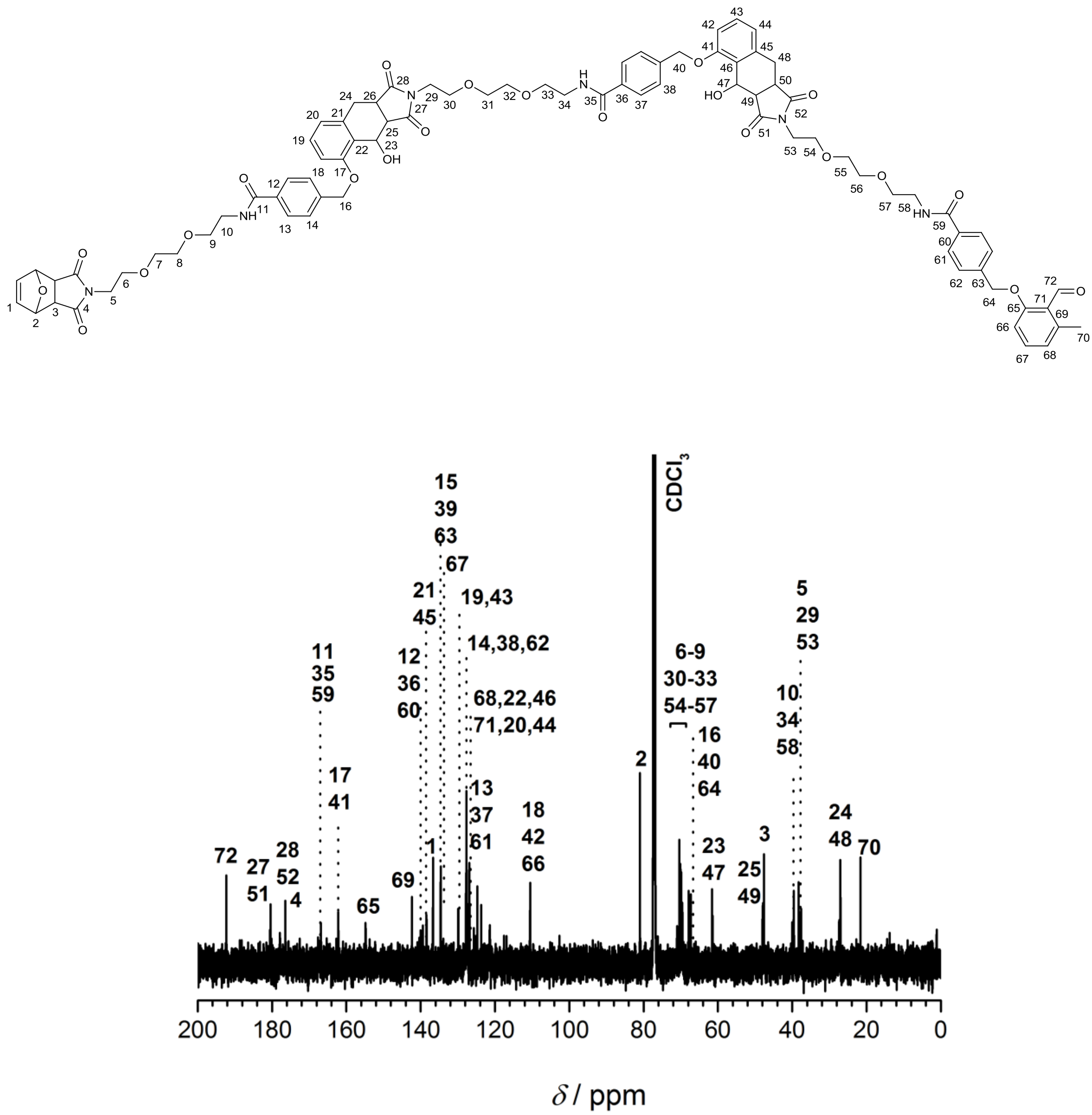

Supplementary Figure 138. ^{13}C NMR spectrum of **12b** ($CDCl_3$).

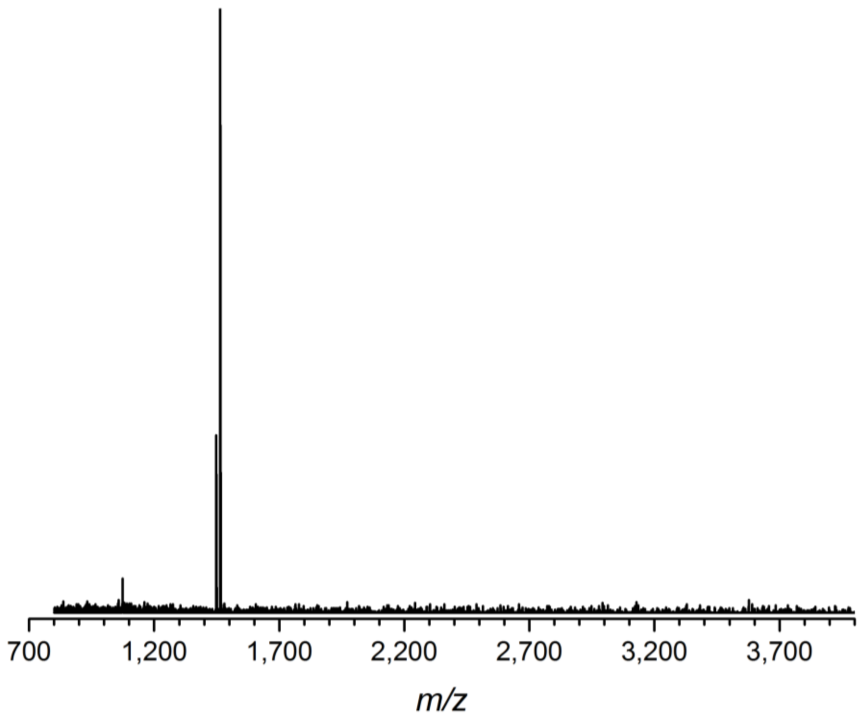

Supplementary Figure 139. MALDI–ToF overview spectrum of **12b**. All major peaks belong to the target molecule with different counter ions. All peak assignments can be found in Supplementary Table 27.

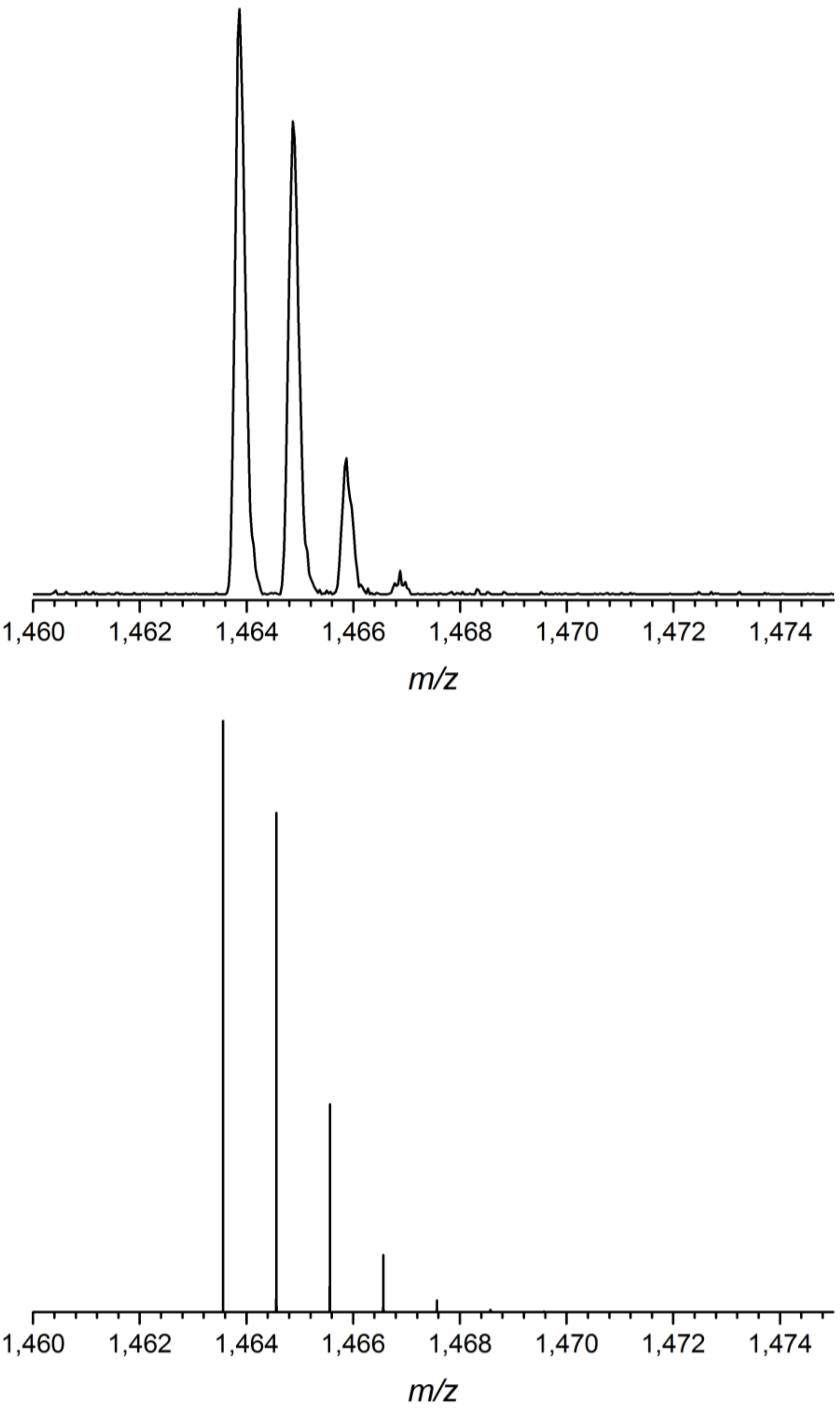

Supplementary Figure 140. MALDI–ToF experimental (top) and calculated (bottom) zoom spectra of **12b**.

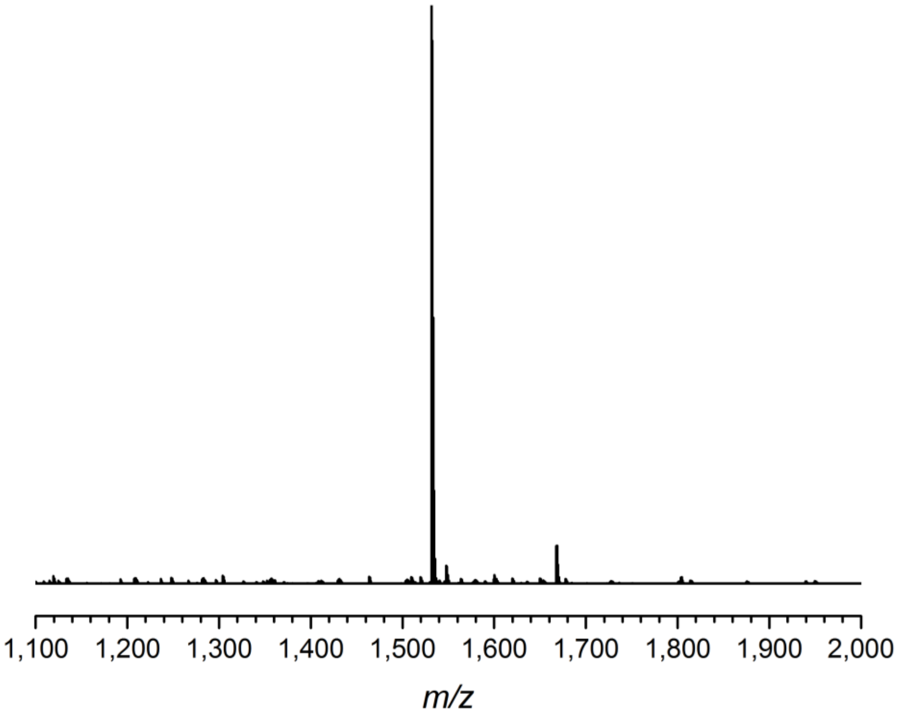

Supplementary Figure 141. ESI-MS overview spectrum of **12b**. All major peaks belong to the target molecule with different counter ions. All peak assignments can be found in Supplementary Table 27.

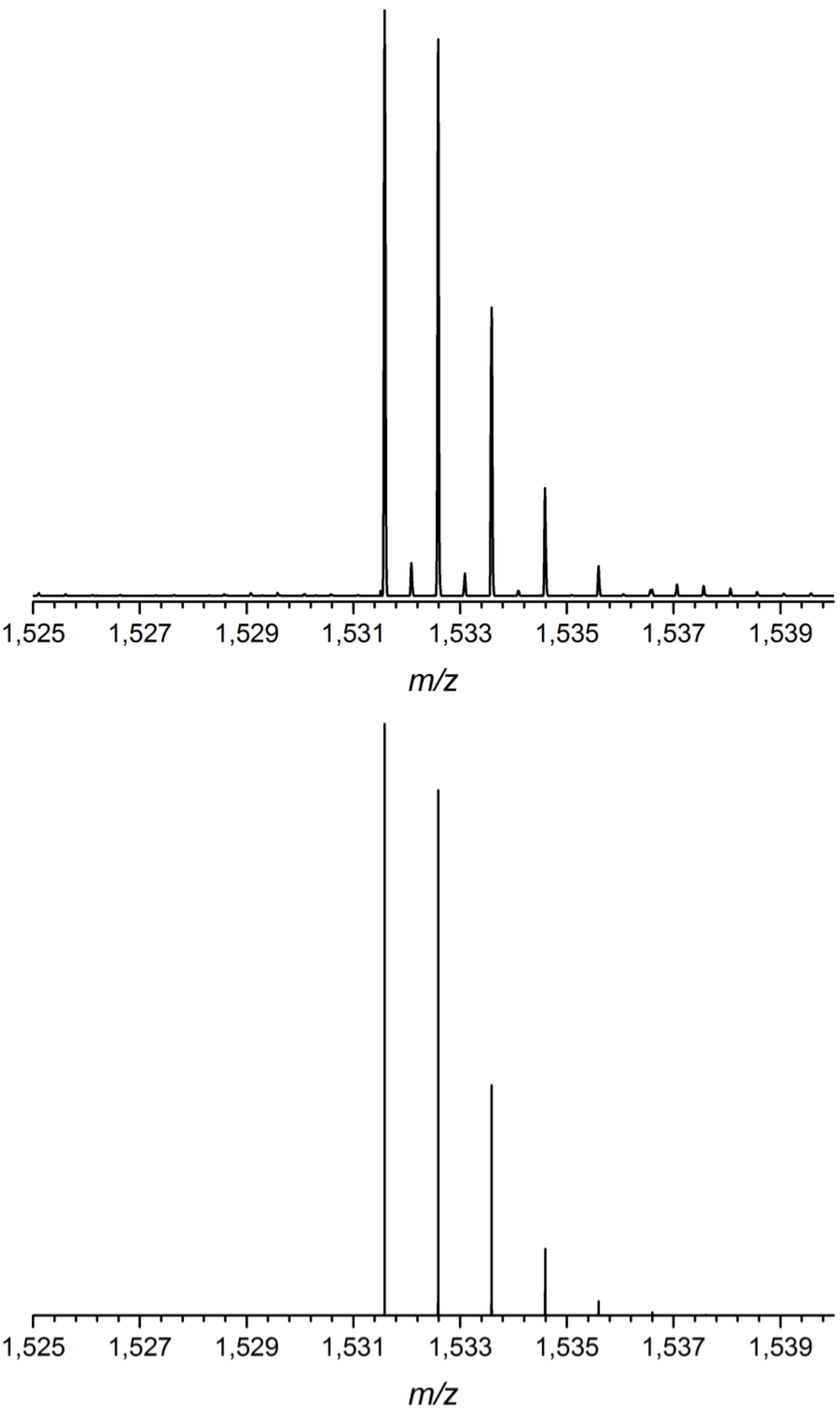

Supplementary Figure 142. ESI-MS experimental (top) and calculated (bottom) zoom spectra of **12b**. The exact mass of the measured sample is matching with the assigned species (see Supplementary Table 27), however there is likely an additional signal from a double charged cluster of two molecules.

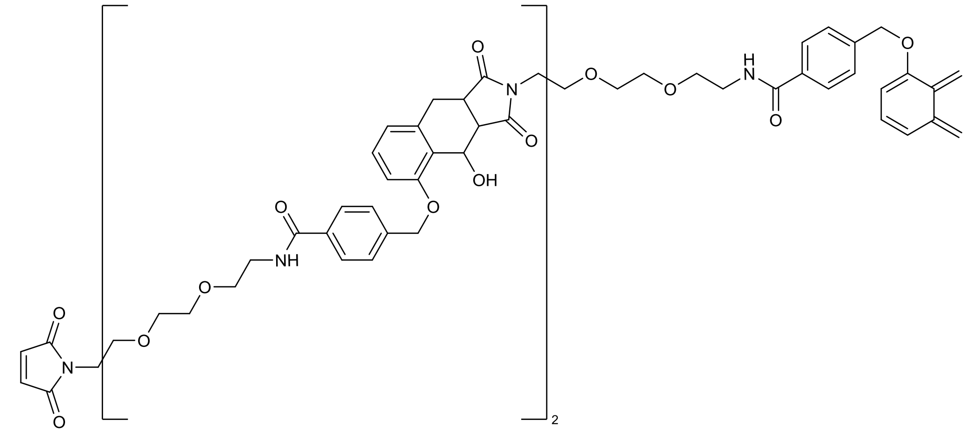

A$_1$

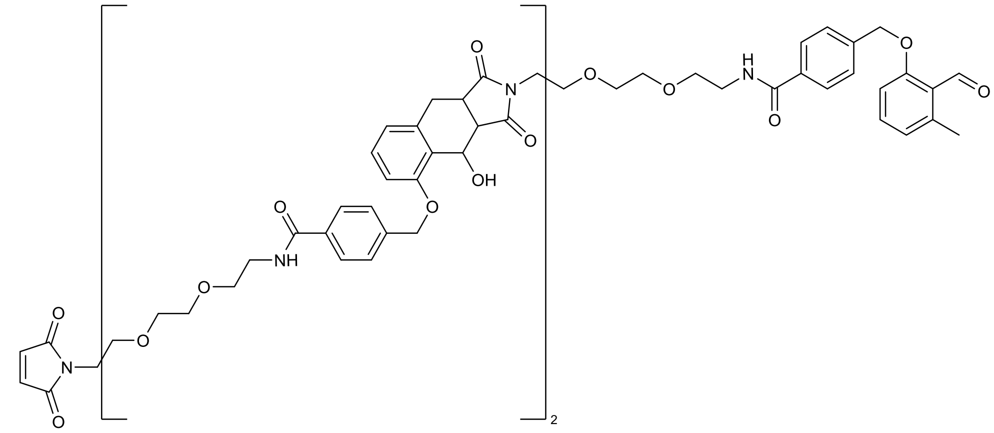

A$_2$

Supplementary Figure 143. Collation of molecules identified in Supplementary Table 27 (**A$_1$-A$_2$**).

Characterization of 12 $(M_3)_5$

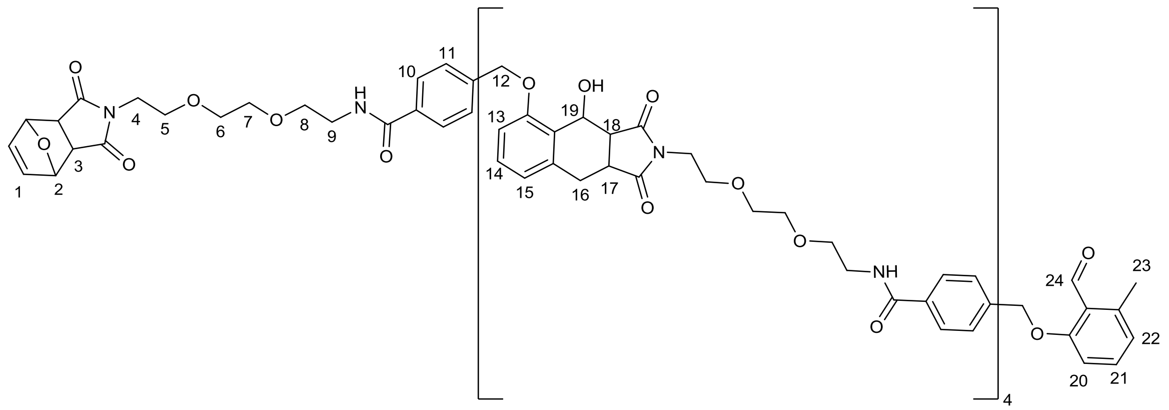

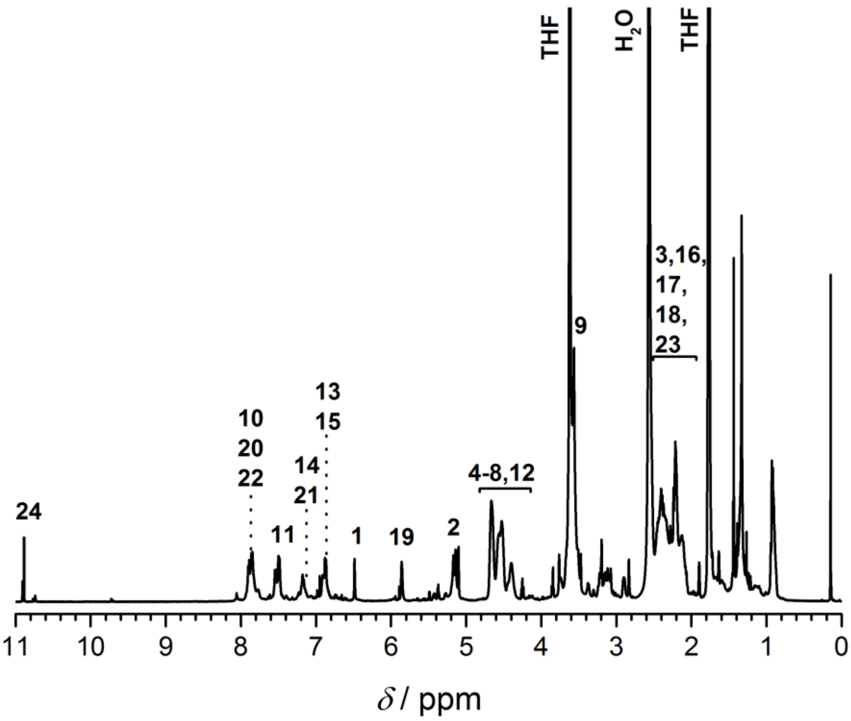

Supplementary Figure 144. ^{1}H NMR spectrum of **12** ($CDCl_3$).

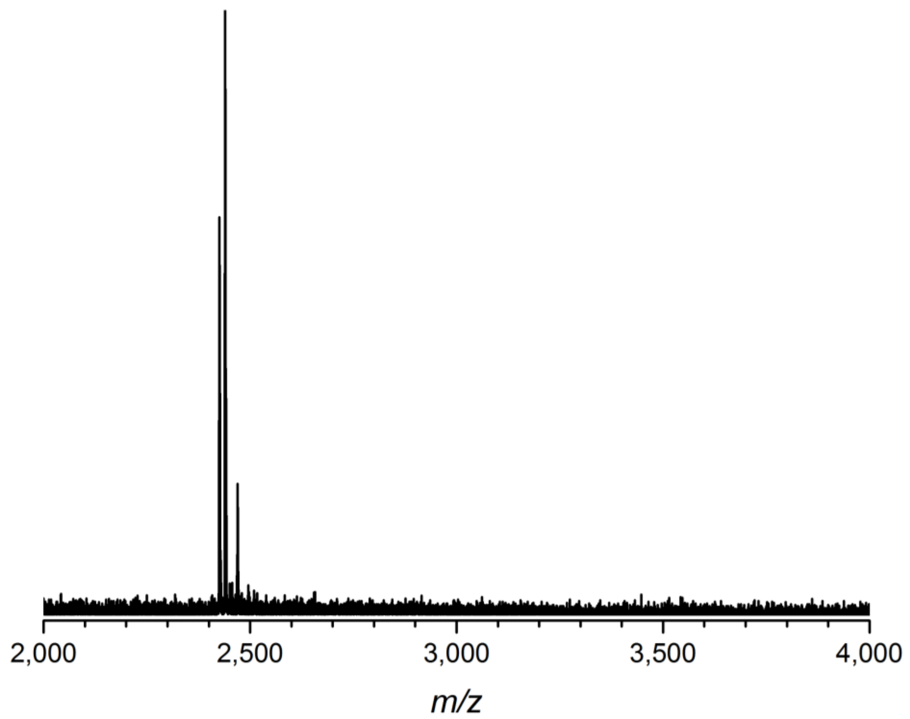

Supplementary Figure 145. MALDI–ToF overview spectrum of **12**. All major peaks belong to the target molecule with different counter ions. All peak assignments can be found in Supplementary Table 28.

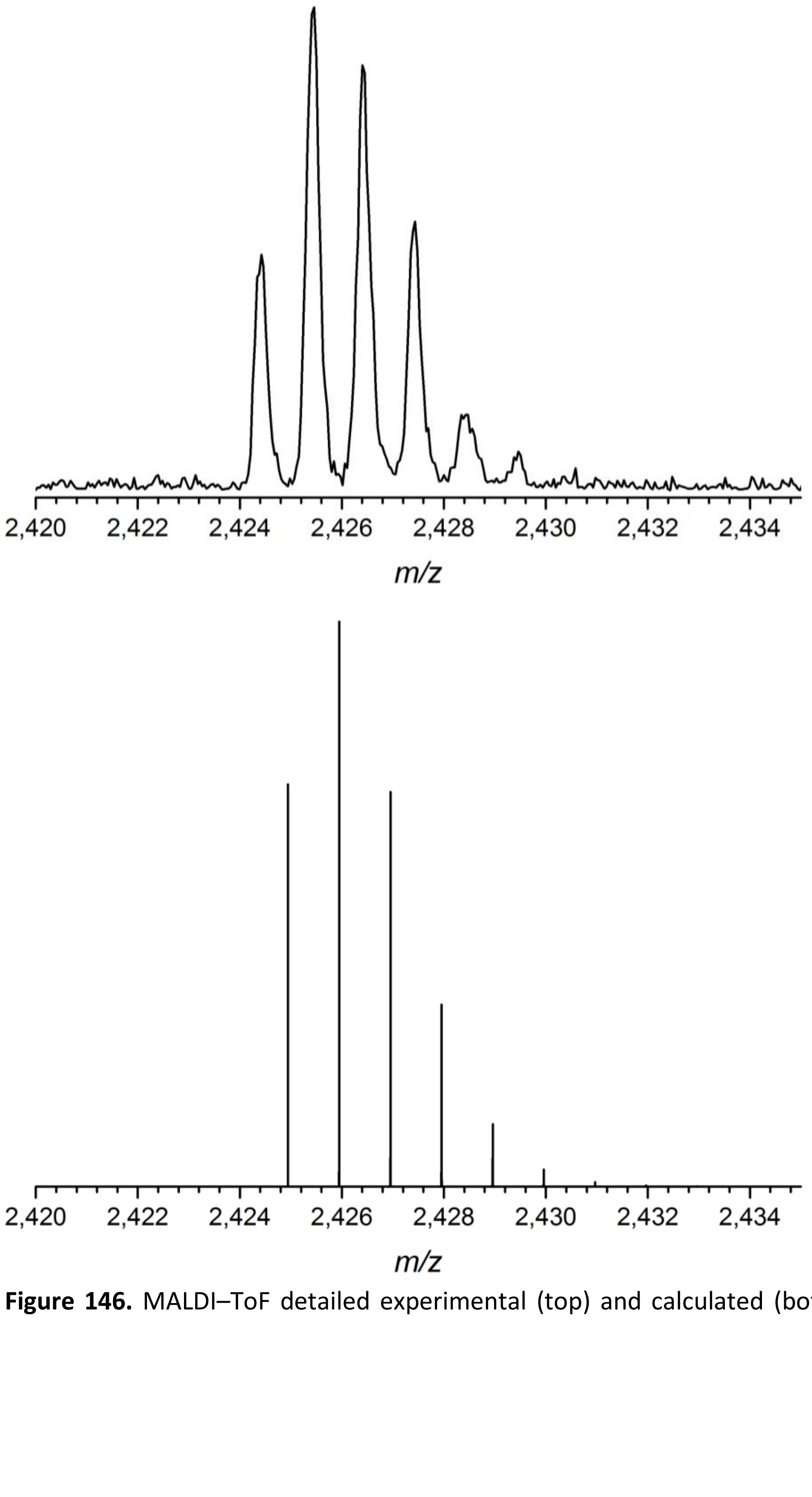

Supplementary Figure 146. MALDI–ToF detailed experimental (top) and calculated (bottom) zoom spectra of **12**.

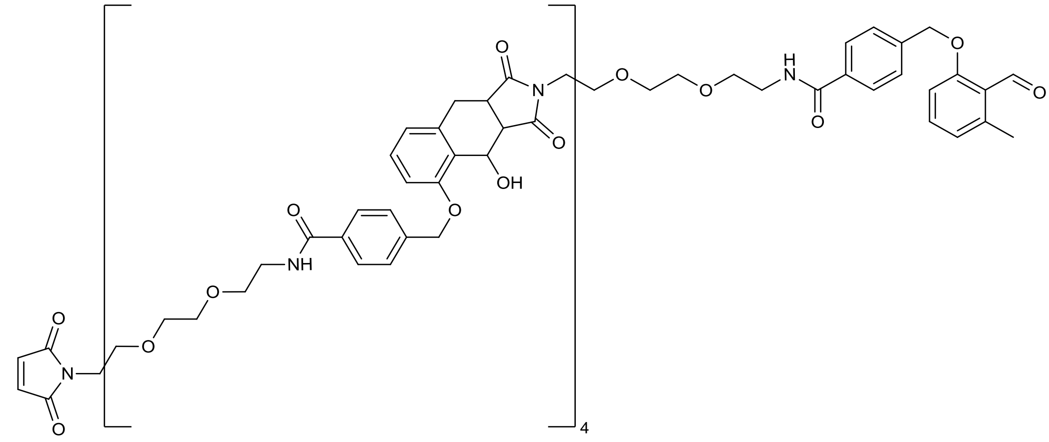

A$_1$

Supplementary Figure 147. Identified molecule reported in Supplementary Table 28 (**A$_1$**).

MALDI–ToF–ToF Mass-Spectrometry Characterization of Compound 7c $(M_1)_3$-X-$(M_1)_3$.

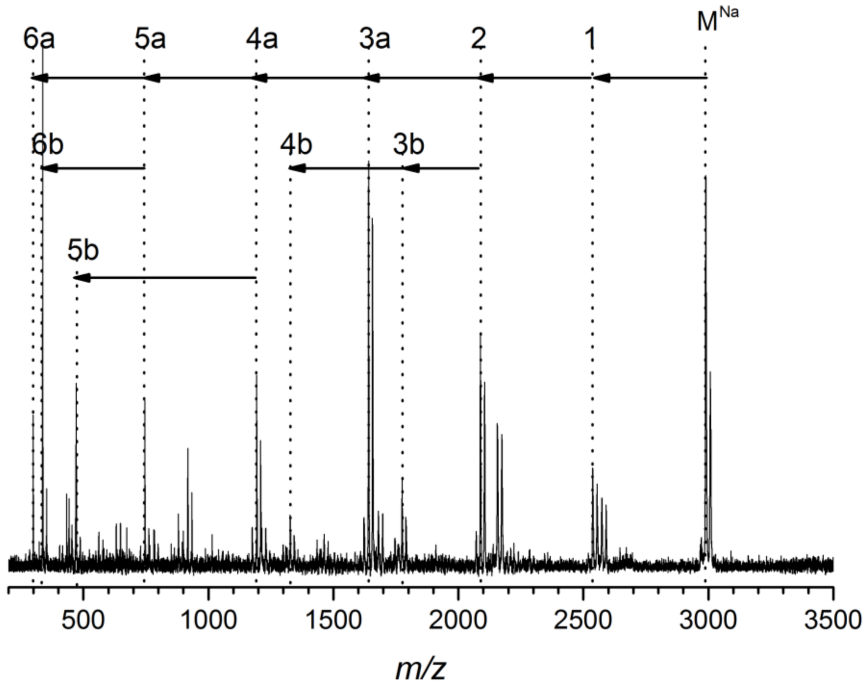

Supplementary Figure 148. MALDI–ToF–ToF spectrum of compound **7c**. Detailed information can be found in Supplementary Figs. 149-150 and in Supplementary Table 29.

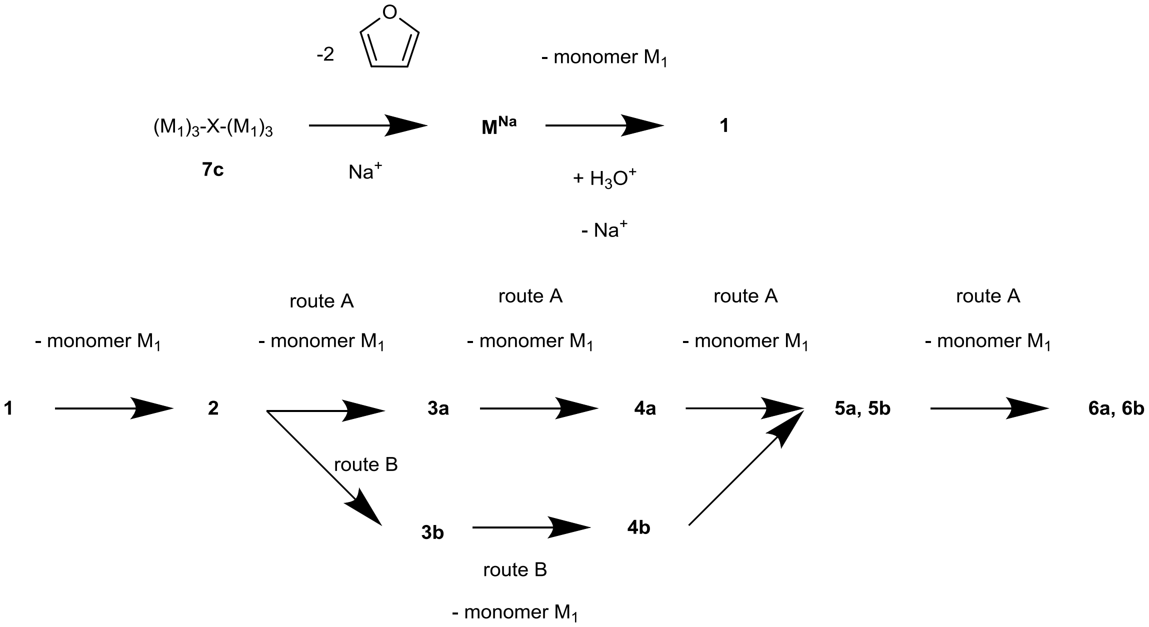

Supplementary Figure 149. Proposed fragmentation pathway of compound **7c**.

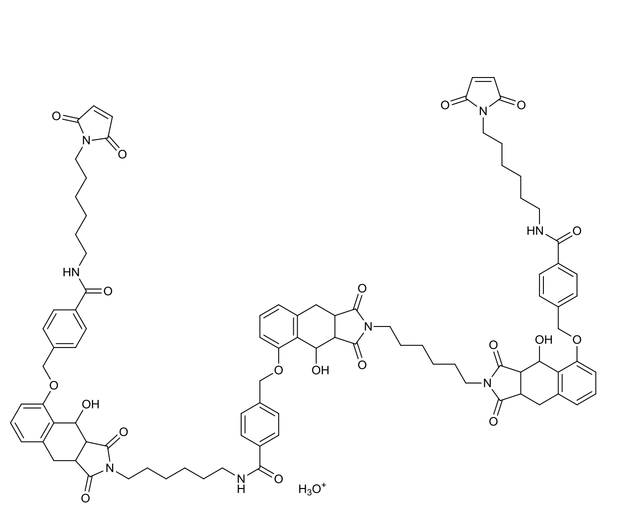

3a

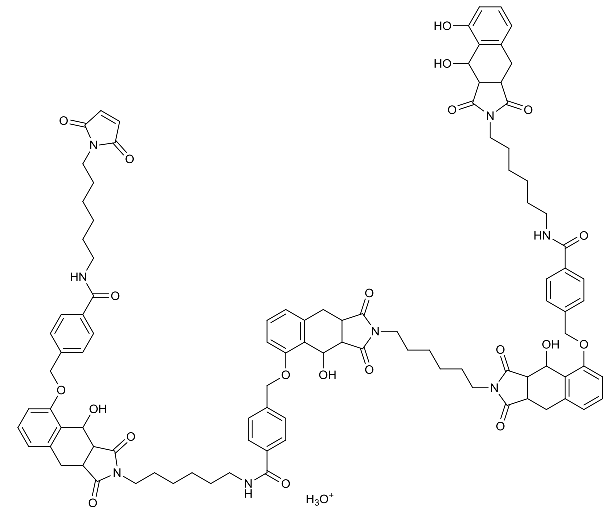

3b

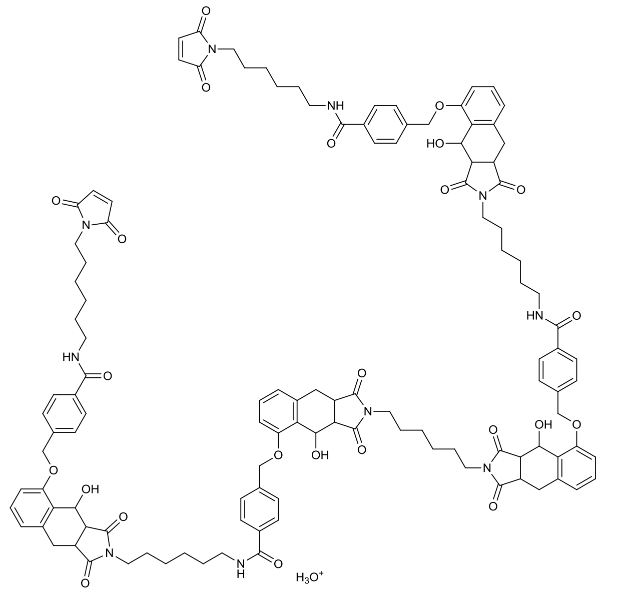

4a

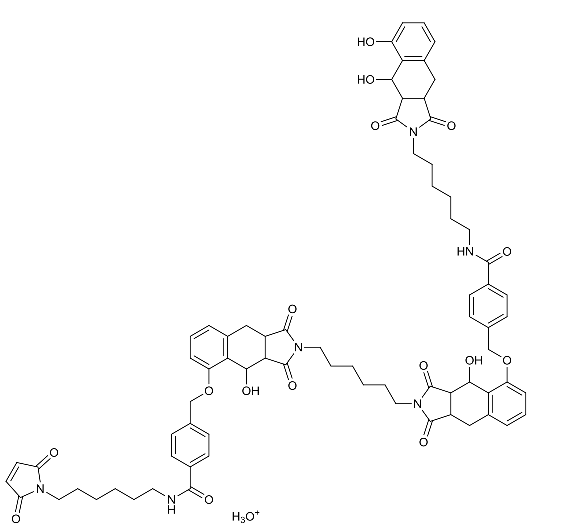

4b

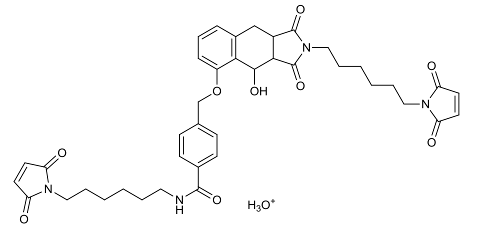

5a

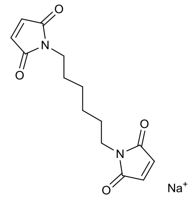

6a

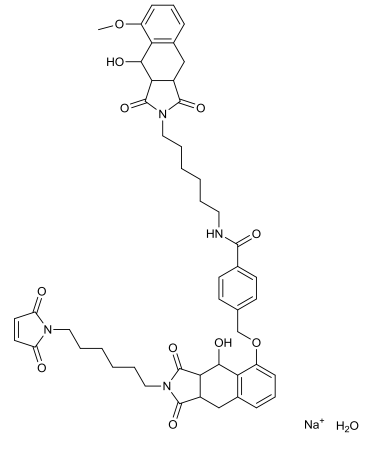

5b

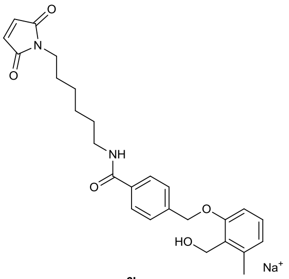

6b

Supplementary Figure 150. Identified structures from MALDI–ToF–ToF spectrum from **7c**.

MALDI–ToF–ToF Mass-Spectrometry Characterization of Compound 8a $(M_2)(M_1)_2$-X-$(M_1)_2(M_2)$

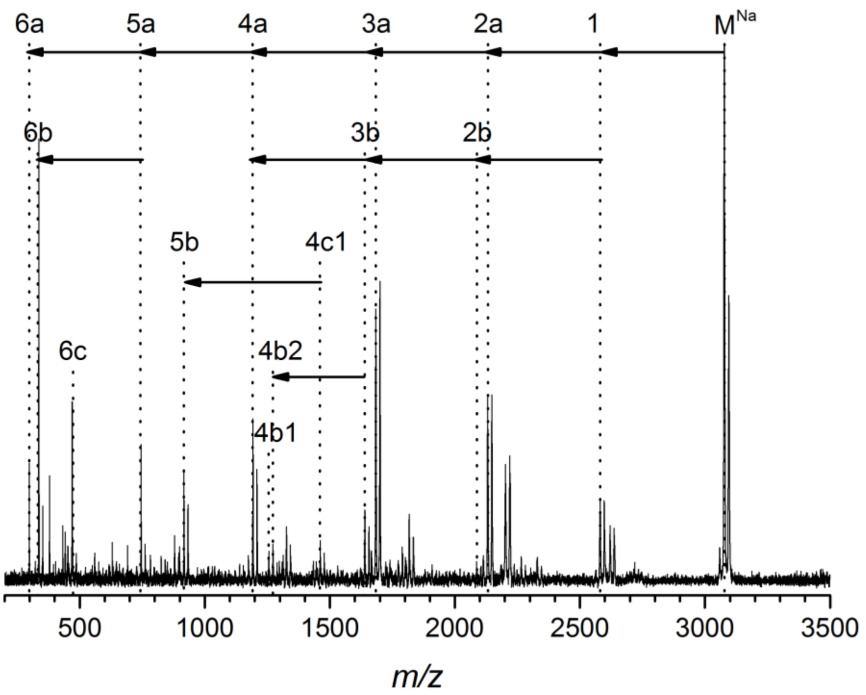

Supplementary Figure 151. MALDI–ToF–ToF spectrum of compound **8a**. Detailed information can be found in Supplementary Figs. 152-153 and in Supplementary Table 30.

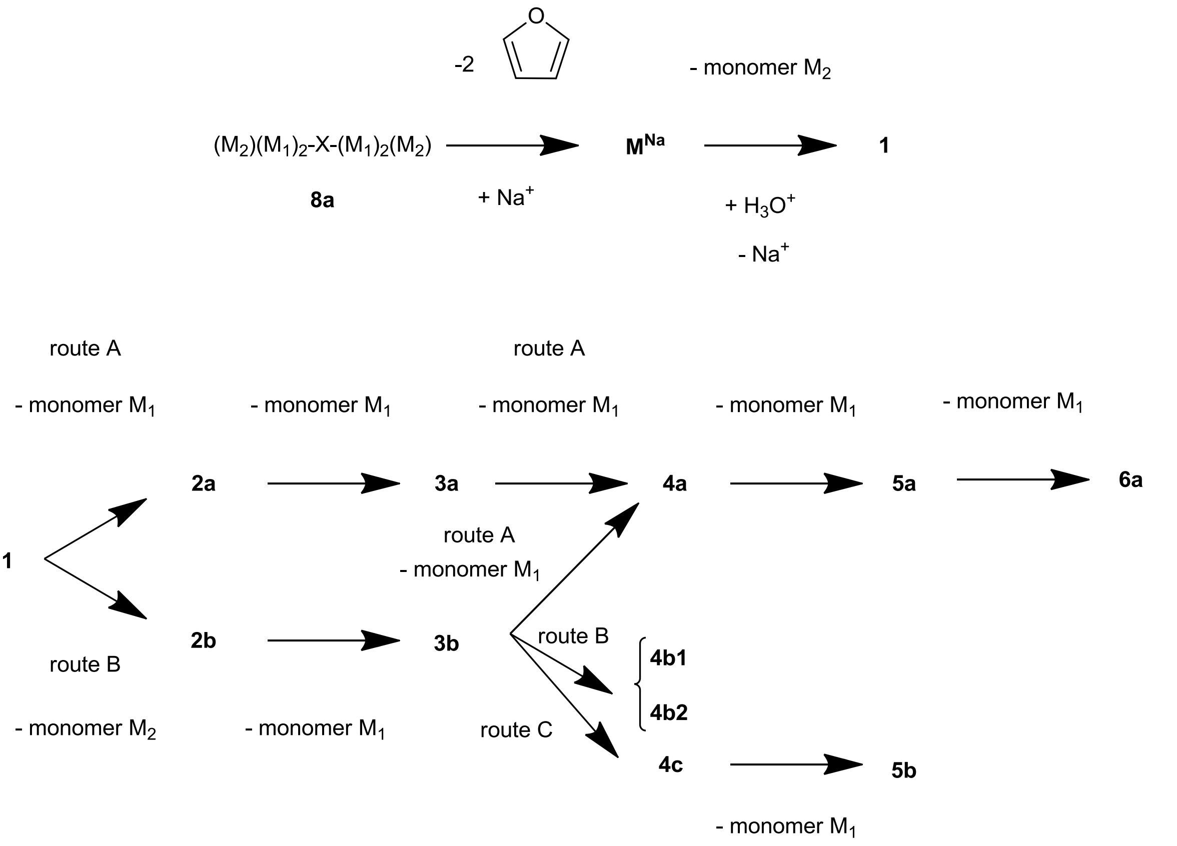

Supplementary Figure 152. Proposed fragmentation pathway of compound **8a**.

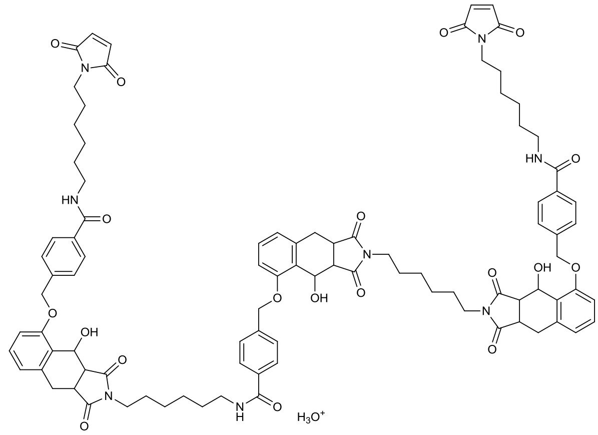

3a

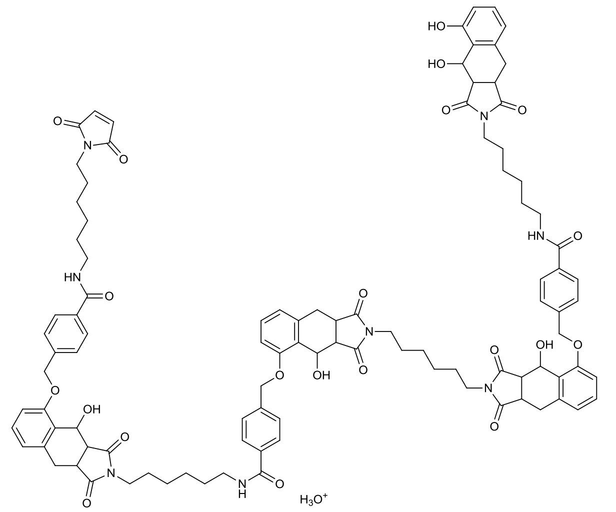

3b

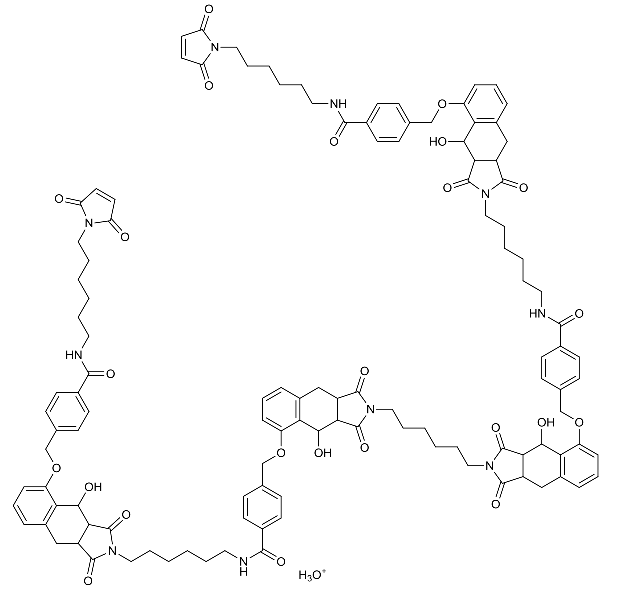

4a

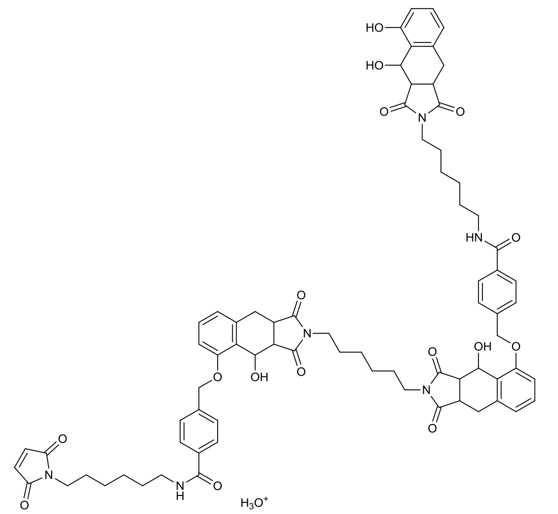

4b

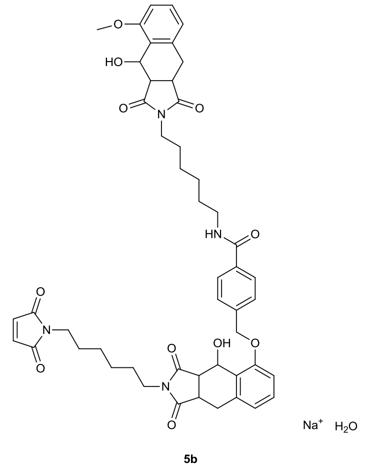

5b

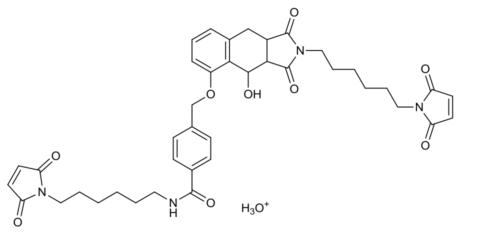

5a

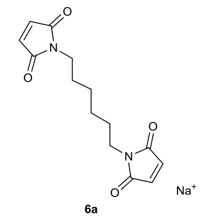

6a

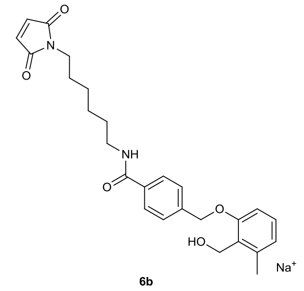

6b

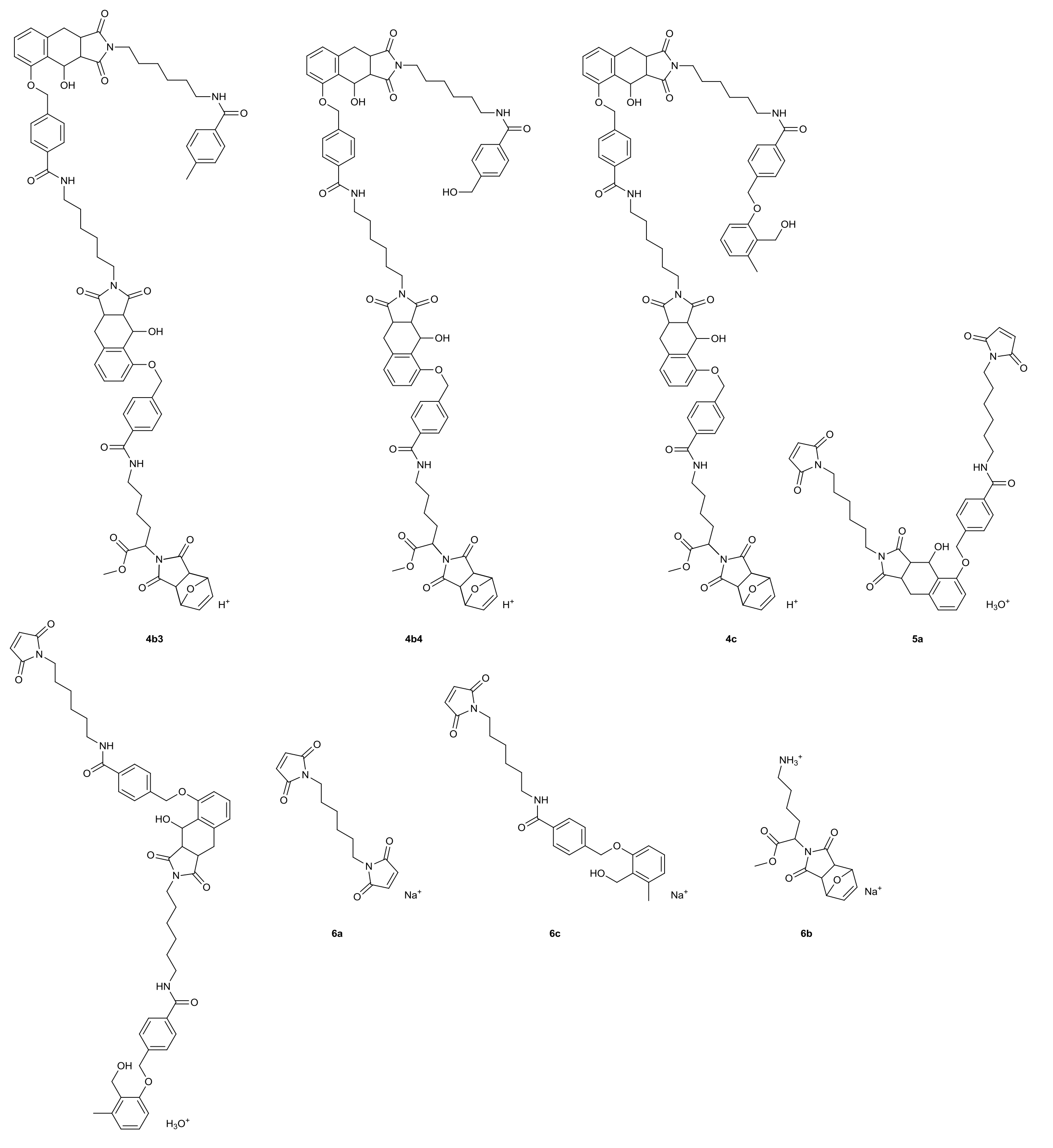

Supplementary Figure 153. Identified structures from MALDI–ToF–ToF spectrum from **8a**.

MALDI–ToF–ToF Mass-Spectrometry Characterization of Compound 9b ($(M_1M_2M_1)$-X-$(M_1M_2M_1)$)

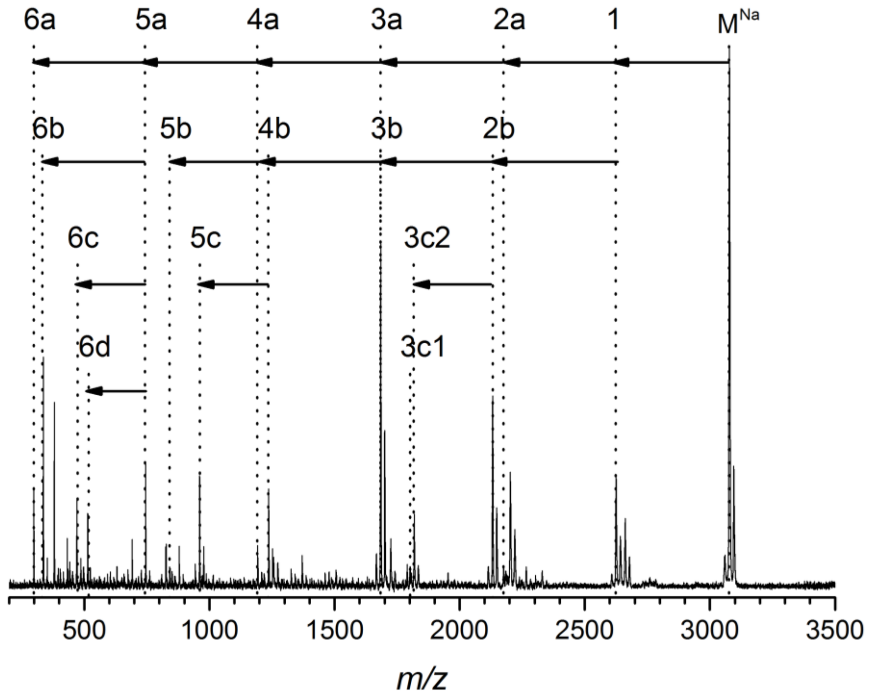

Supplementary Figure 154. MALDI–ToF–ToF spectrum of compound **9b**. Detailed information can be found in Supplementary Figs. 155-156 and in Supplementary Table 31.

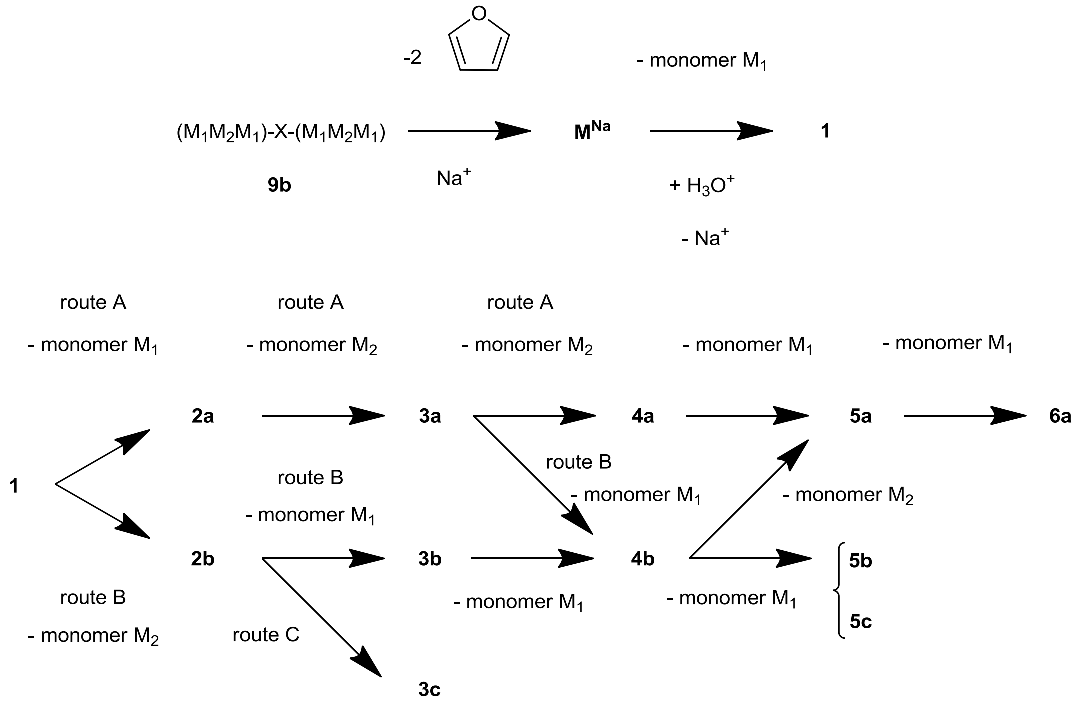

Supplementary Figure 155. Proposed fragmentation mechanism of compound **9b**.

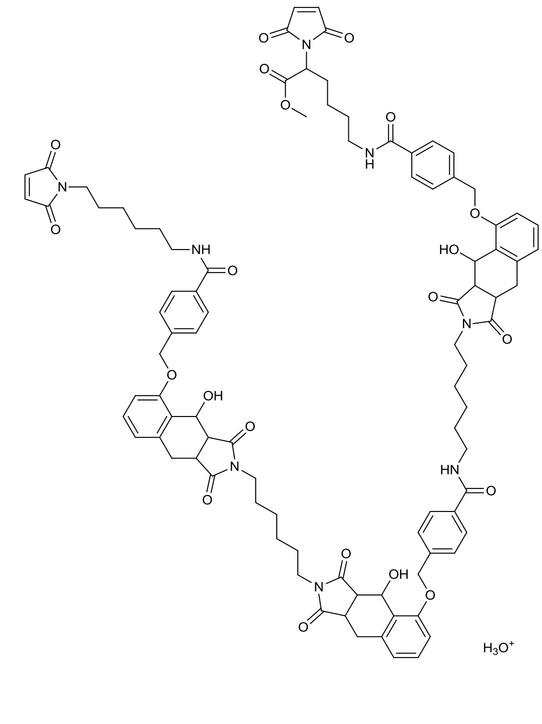

3a

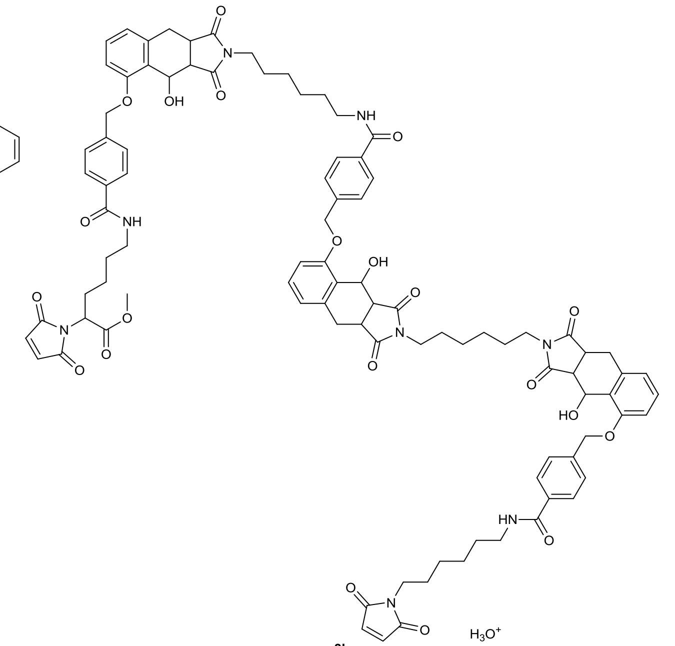

3b

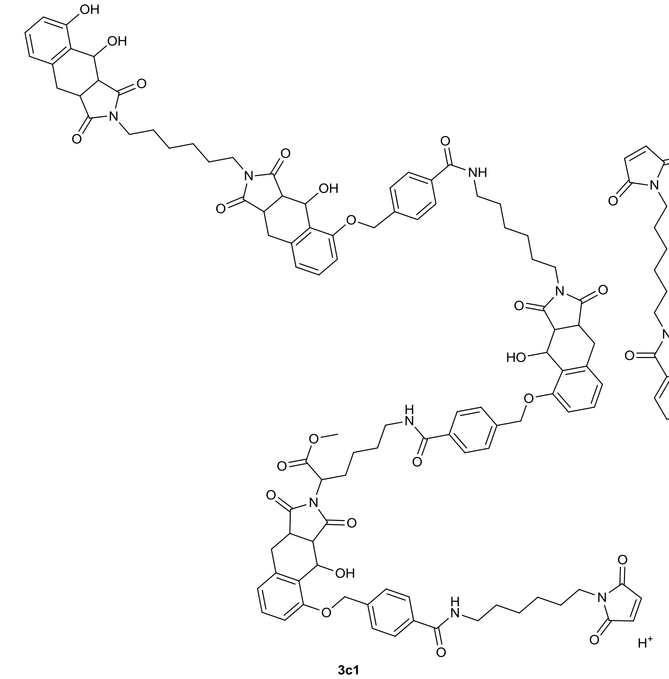

3c1

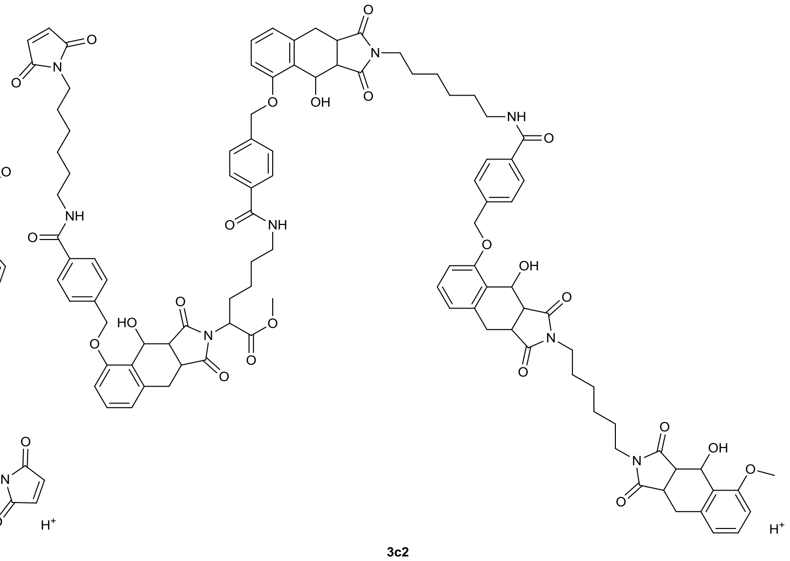

3c2

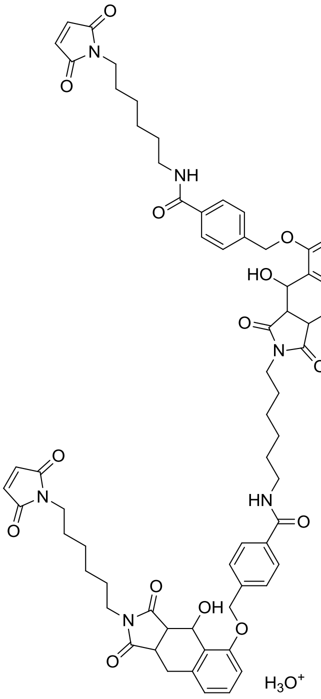

4a

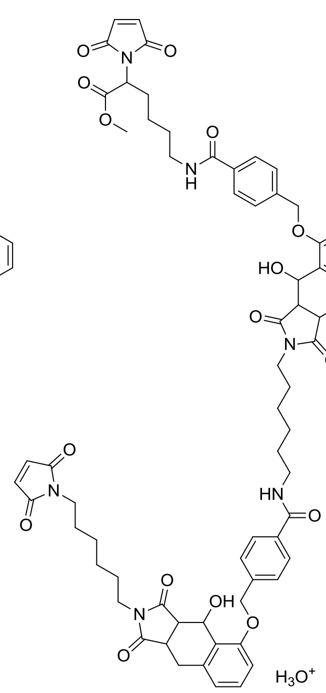

4b

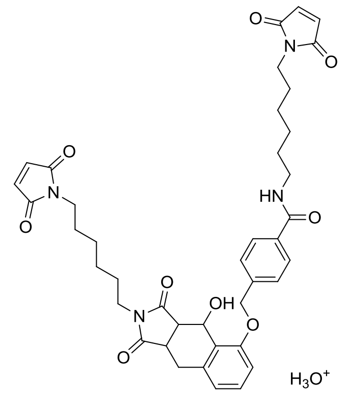

5a

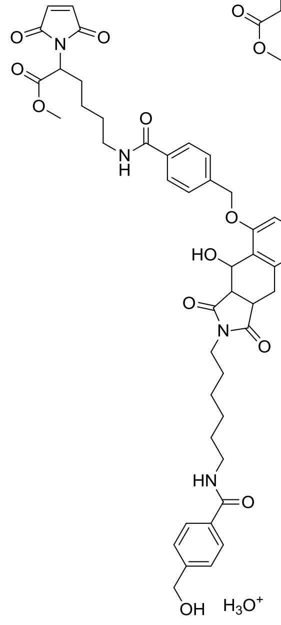

5b

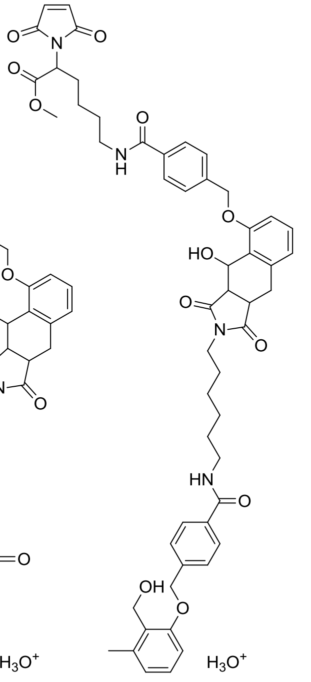

5c

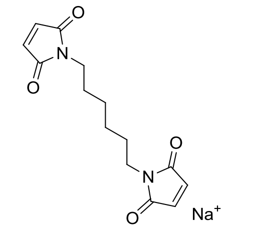

6a

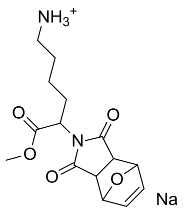

6b

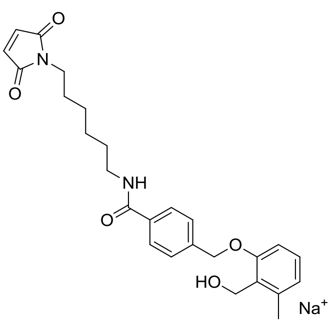

6c

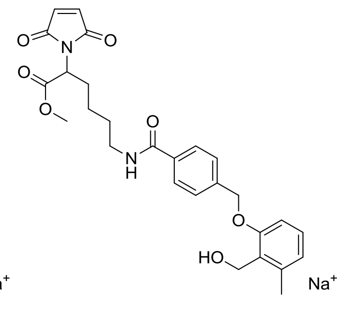

6d

Supplementary Figure 156. Identified structures from MALDI–ToF–ToF spectrum from **9b**.

MALDI–ToF–ToF Mass-Spectrometry Characterization of Compound 10c ($M_2M_1M_2$)-X-($M_2M_1M_2$)

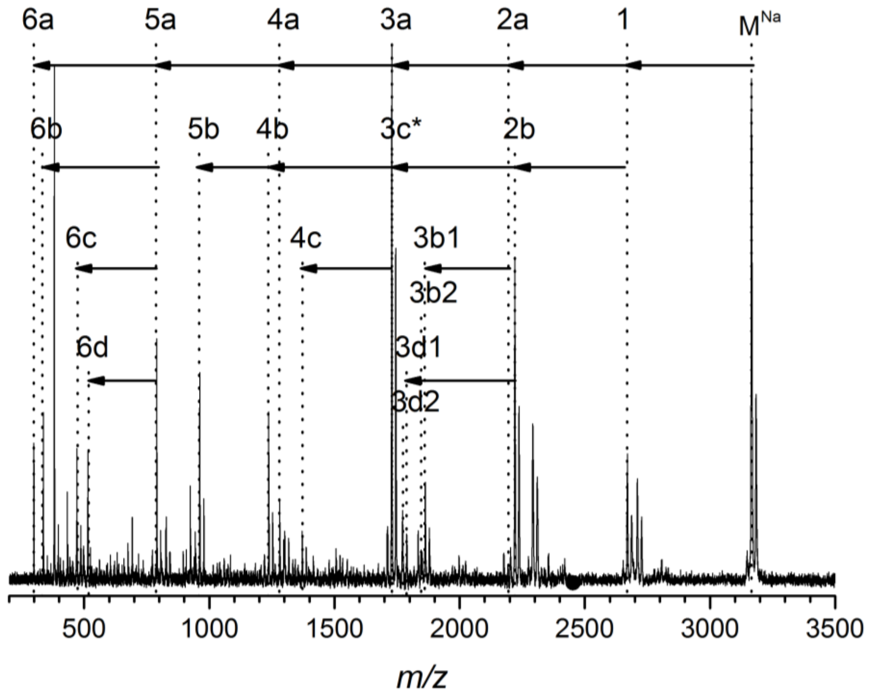

Supplementary Figure 157. MALDI–ToF–ToF spectrum of compound **10c**. Detailed information can be found in Supplementary Figs. 158-159 and in Supplementary Table 32.

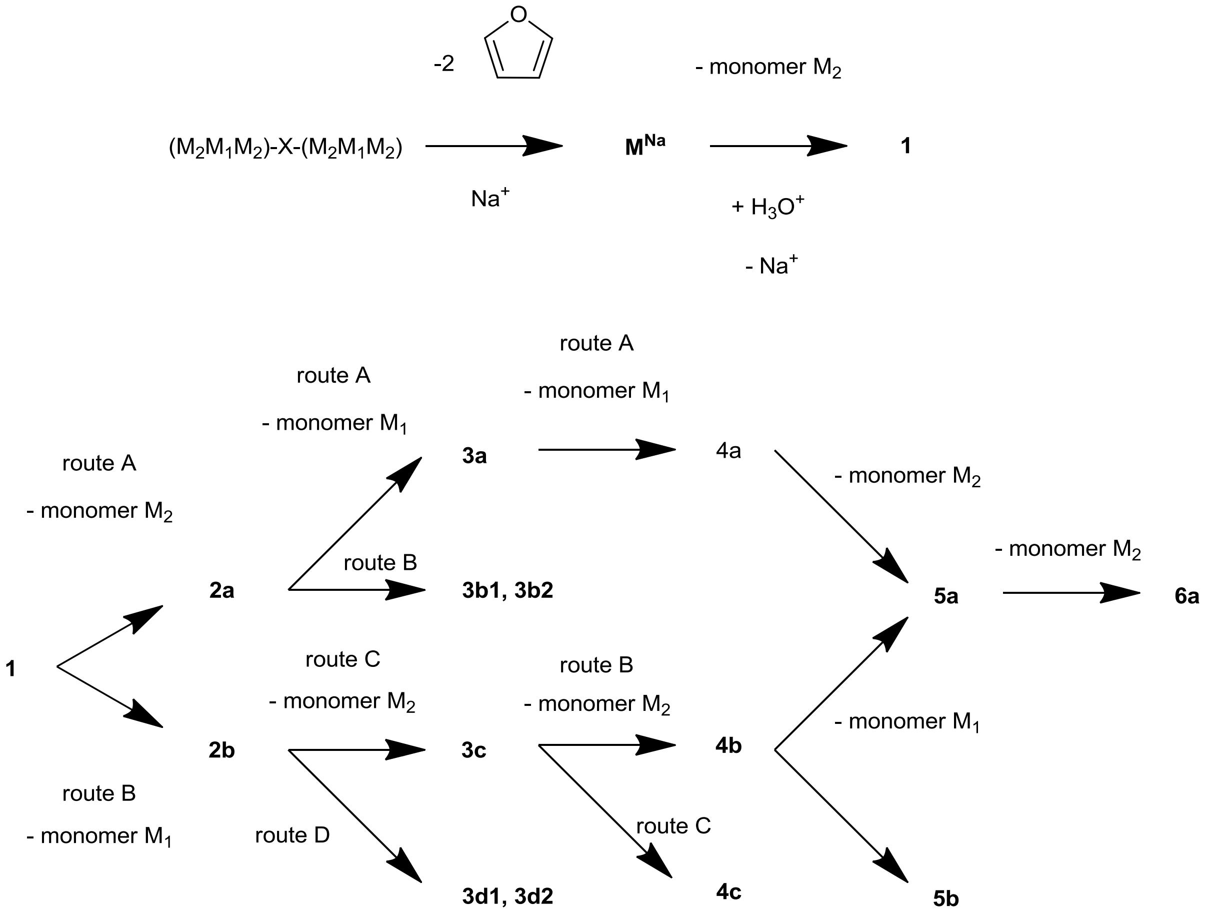

Supplementary Figure 158. Proposed fragmentation mechanism of compound **10c**.

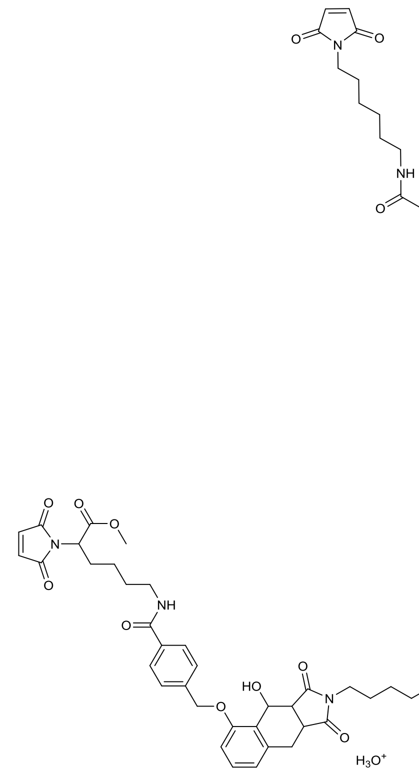

3a

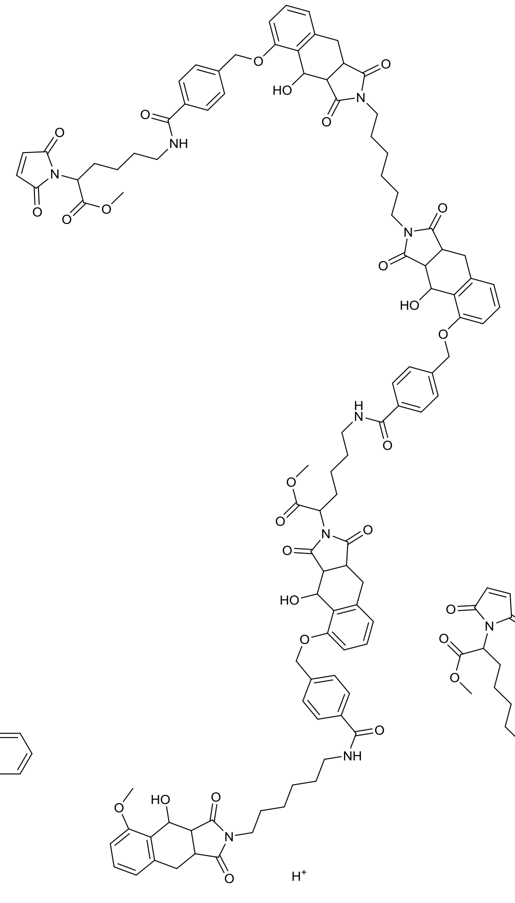

3b$_1$

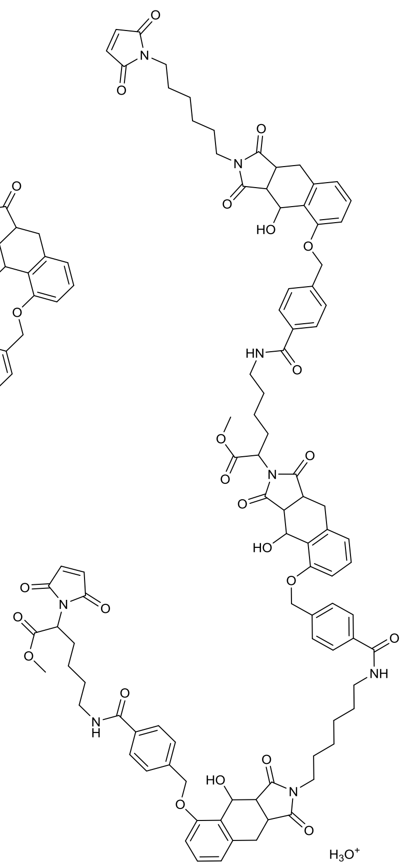

3c

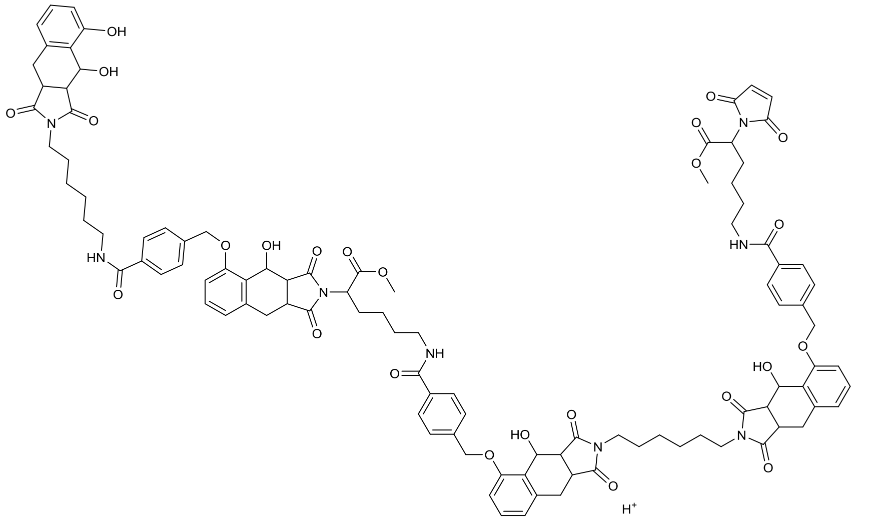

3b$_2$

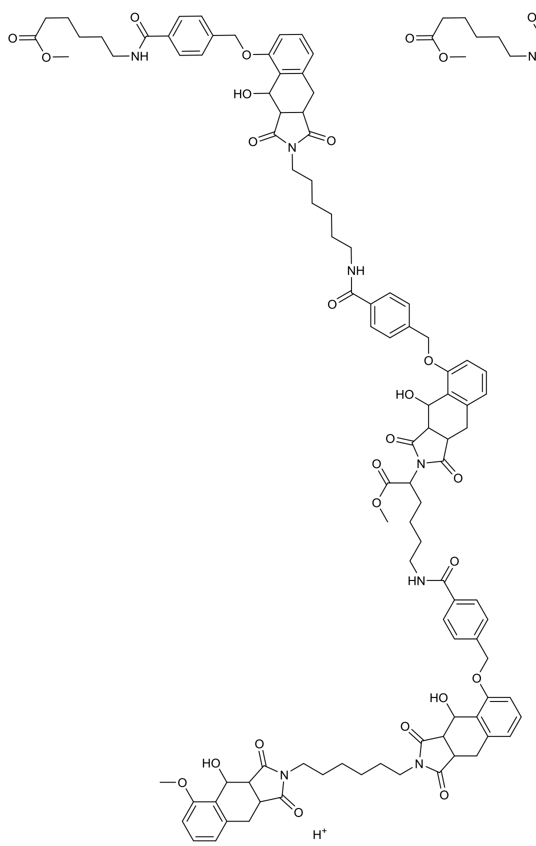

3d1

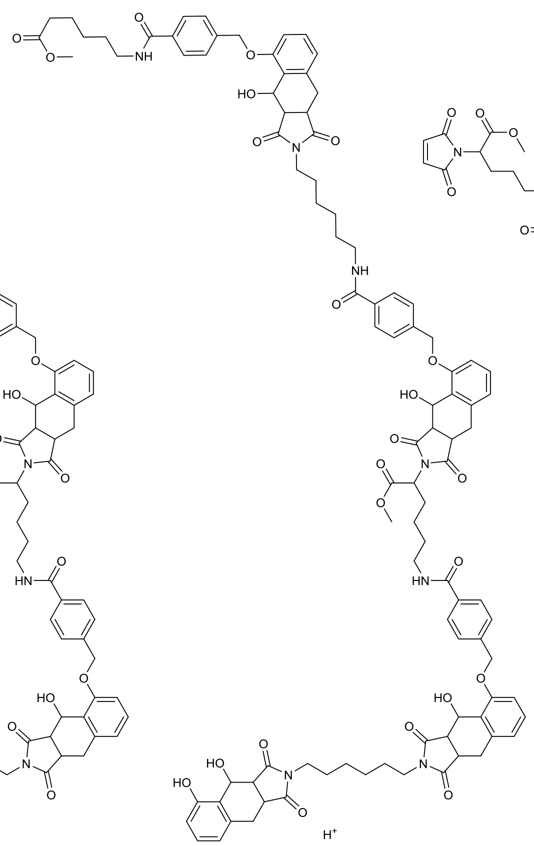

3d2

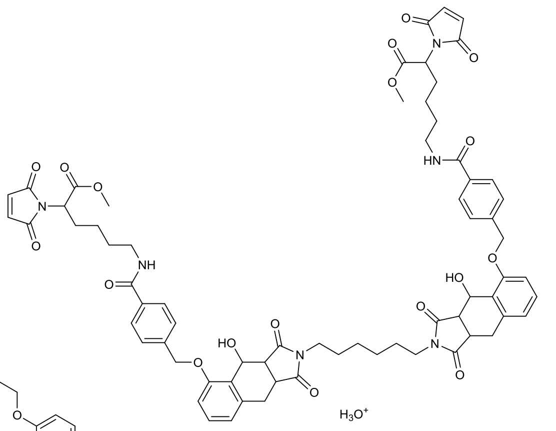

4a

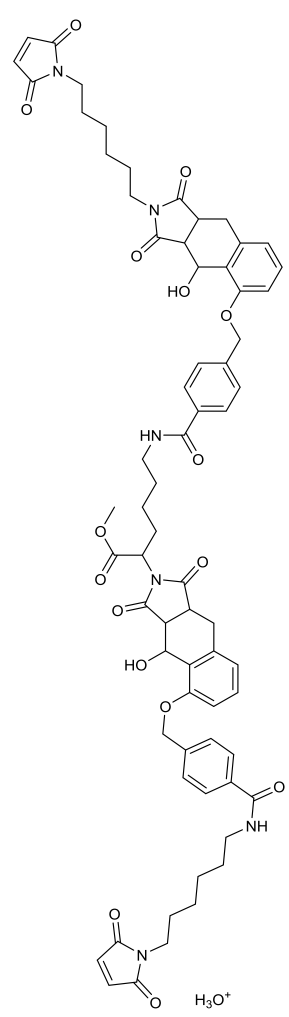

4b

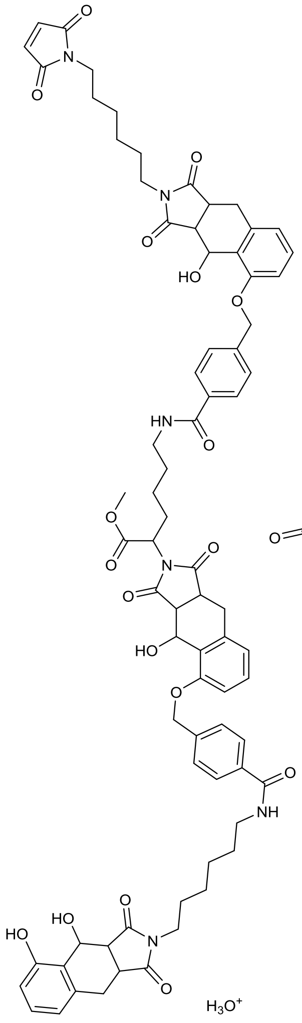

4c

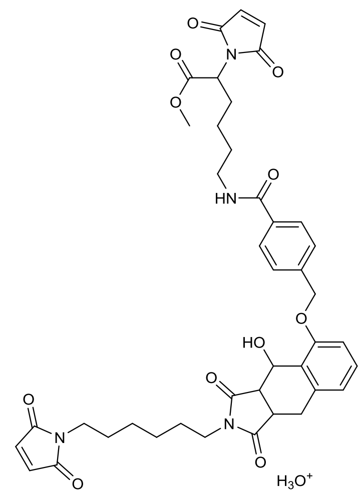

5a

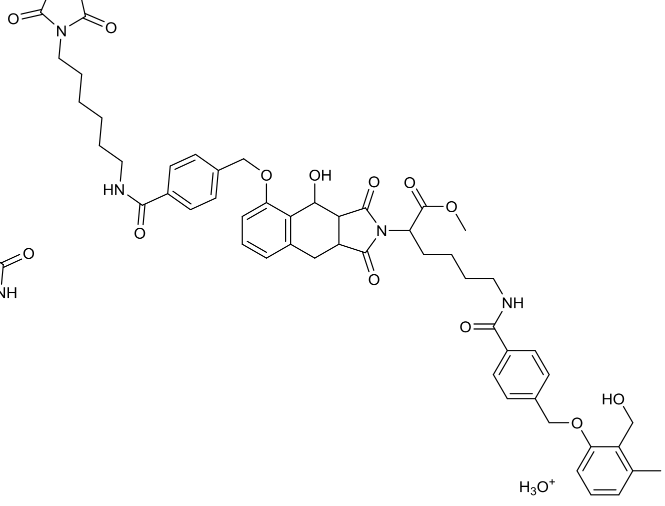

5b

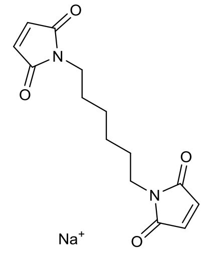

6a

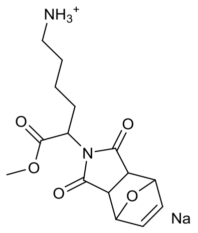

6b

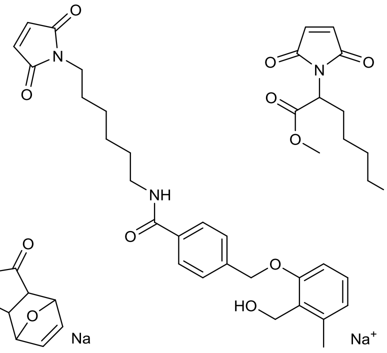

6c

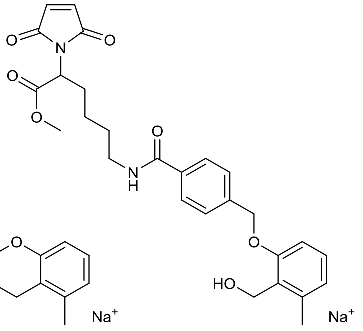

6d

Supplementary Figure 159. Identified structures from MALDI–ToF–ToF spectrum from **10c**.

MS-Characterization of Synthons 1-6 (Monomers M_1 to M_6)

Formula	*m/z* th.	*m/z* exp.	Assignment
			**MALDI–ToF**
**$[C_{26}H_{28}N_2NaO_5]^+$**	**471.189**	**471.189**	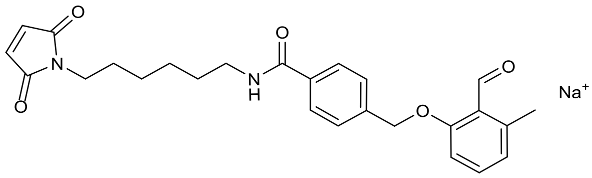
$[C_{26}H_{28}KN_2O_5]^+$	487.163	487.177	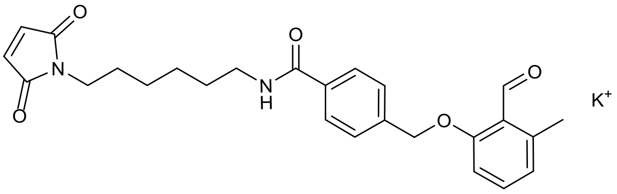
			**ESI-MS**
$[C_{30}H_{32}N_2O_6]^+$	517.2333	517.2367	M_1, H^+
**$[C_{30}H_{32}N_2O_6Na]^+$**	**539.2153**	**539.2189**	**M_1, Na^+**

Supplementary Table 1. Identification of ions observed during the mass spectrometric characterization (MALDI–ToF and ESI-MS) of **1**.

Formula	*m/z* th.	*m/z* exp.	Assignment
		**MALDI–ToF**	
$[C_{27}H_{28}N_2O_7]^+$	492.189	492.236	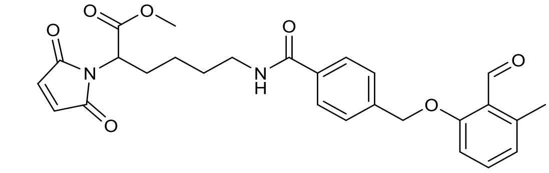
**$[C_{27}H_{28}N_2O_7Na]^+$**	**515.179**	**515.209**	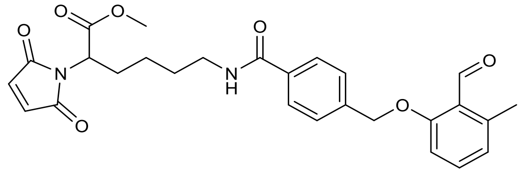 Na^+
$[C_{27}H_{28}N_2O_7K]^+$	531.153	531.197	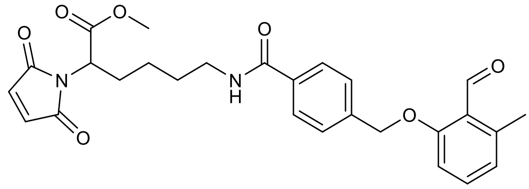 K^+
		**ESI-MS**	
$[C_{27}H_{28}N_2O_7Na]^+$	515.1822	515.1803	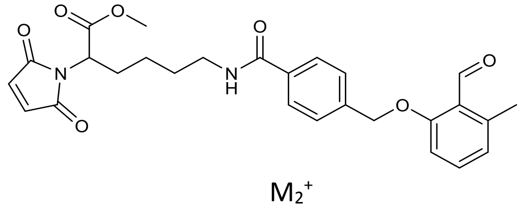 Na^+
$[C_{31}H_{32}N_2O_8]^+$	561.2247	561.2249	M_2^+
**$[C_{31}H_{32}N_2O_8Na]^+$**	**583.2051**	**583.2075**	**M_2, Na^+**

Supplementary Table 2. Identification of ions observed during the mass spectrometric characterization (MALDI–ToF and ESI-MS) of **2**.

Formula	*m/z* th.	*m/z* exp.	Assignment
		**MALDI–ToF**	
**$[C_{26}H_{28}N_2NaO_7]^+$**	**503.179**	**503.197**	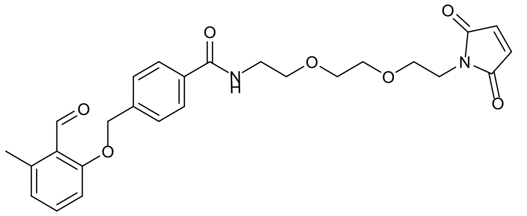 Na^+
$[C_{26}H_{28}KN_2O_7]^+$	519.153	519.178	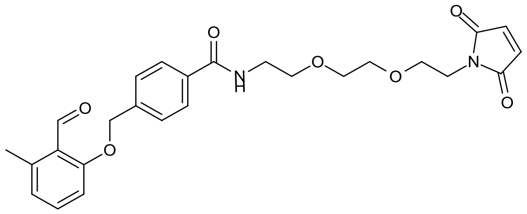 K^+
		**ESI-MS**	
**$[C_{30}H_{32}N_2O_8Na]^+$**	**571.2050**	**571.2089**	**M_3, Na^+**

Supplementary Table 3. Identification of ions observed during the mass spectrometric characterization (MALDI–ToF and ESI-MS) of **3**.

Formula	*m/z* th.	*m/z* exp.	Assignment
		**MALDI–ToF**	
$[C_{36}H_{40}N_3NaO_8]^+$	642.281	642.425	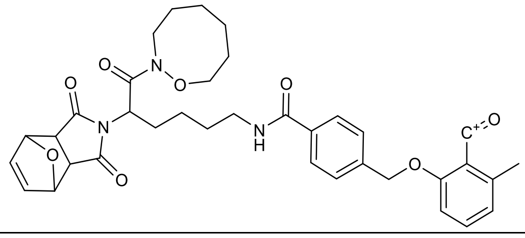
		**ESI-MS**	
$[C_{38}H_{45}N_3O_9Na]^+$	710.3048	710.3055	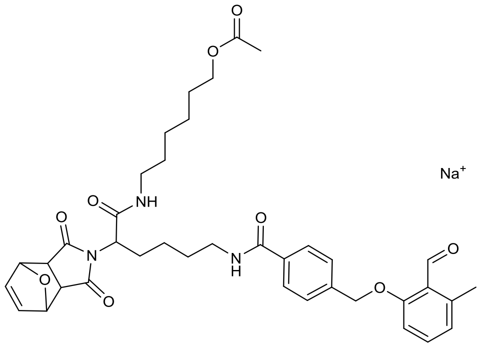

Supplementary Table 4. Identification of ions observed during the mass-spectrometric characterization (MALDI–ToF and ESI-MS) of **4**.

Formula	*m/z* th.	*m/z* exp.	Assignment
	**MALDI–ToF**		
**$[C_{37}H_{43}N_3NaO_6]^+$**	**648.304**	**648.457**	**A_1, Na^+**
$[C_{37}H_{43}N_3NaO_6]^+$	664.278	664.447	A_1, K^+
	**ESI-MS**		
$[C_{41}H_{47}N_3O_7Na]^+$	716.3306	716.3345	M_5, Na^+

Supplementary Table 5. Identification of ions observed in the mass spectrometric characterization (MALDI–ToF and ESI-MS) of **5**. The identified molecules are represented in Supplementary Fig. 25 (**A_1**).

Formula	*m/z* th.	*m/z* exp.	Assignment
	**MALDI–ToF**		
**$[C_{33}H_{32}FN_3NaO_6]^+$**	**608.217**	**608.357**	**A_1, Na^+**
$[C_{33}H_{32}FN_3KO_6]^+$	624.191	624.348	A_1, K^+
	**ESI-MS**		
$[C_{33}H_{32}FN_3O_6]^+$	585.2269	585.5342	A_1^+
$[C_{33}H_{32}FN_3NaO_6]^+$	608.2167	608.2187	A_1, Na^+
**$[C_{37}H_{36}FN_3O_7Na]^+$**	**676.2429**	**676.2446**	**M_6, Na^+**

Supplementary Table 6. Identification of ions observed during the mass spectrometric characterization (MALDI–ToF and ESI-MS) of **6**.

MS-Characterization of Oligomer 7 and Precursors 7a-7d

Formula	*m/z* th.	*m/z* exp.	Assignment
		**MALDI–ToF**	
$[C_{66}H_{72}N_6NaO_{14}]^+$	1195.500	1195.554	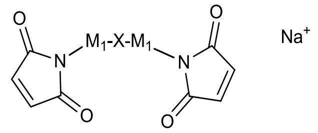
		**ESI-MS**	
$[C_{70}H_{76}N_6NaO_{11}]^+$	1263.5261	1263.5335	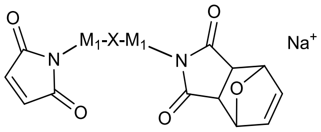
**$[C_{74}H_{80}N_6NaO_{16}]^+$**	**1331.5523**	**1331.5626**	**(M_1)-X-(M_1), Na^+**
$[C_{74}H_{80}N_6KO_{16}]^+$	1347.5262	1347.5229	(M_1)-X-(M_1),K^+
$[C_{74}H_{82}N_6NaO_{18}]^+$	1365.5578	1365.5179	(M_1)-X-(M_1), 2OH, Na^+
$[C_{76}H_{80}N_6F_3Na_2O_{18}]^+$	1467.5271	1467.5339	(M_1)-X-(M_1), NaTFA, Na^+

Supplementary Table 7. Identification of ions observed during the mass-spectrometric characterization (MALDI–ToF and ESI-MS) of **7a**.

Formula	*m/z* th.	*m/z* exp.	Assignment
		**MALDI–ToF**	
$[C_{118}H_{128}N_{10}NaO_{24}]^+$	2091.90<br>(2092.90)	2091.80<br>(2092.80)	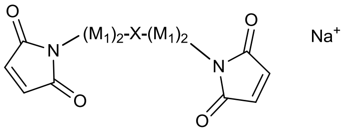
		**ESI-MS**	
$[C_{122}H_{132}N_{10}NaO_{25}]^+$	2159.9252	2159.9416	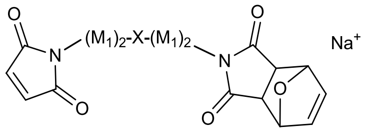
**$[C_{126}H_{136}N_{10}NaO_{26}]^+$**	**2227.9519**	**2227.9552**	**$(M_1)_2$-X-$(M_1)_2$, Na^+**
$[C_{252}H_{272}N_{20}Na_2O_{52}]^{2+}$	2227.9519	2227.9552	2[$(M_1)_2$-X-$(M_1)_2$], $2Na^+$
$[C_{126}H_{136}N_{10}KO_{26}]^{2+}$	2243.9253	2243.9340	$(M_1)_2$-X-$(M_1)_2$, K^+

Supplementary Table 8. Identification of ions observed during the mass-spectrometric characterization (MALDI–ToF and ESI-MS) of **7b**.

Formula	*m/z* th	*m/z* exp	Assignment
		**MALDI–ToF**	
**$[C_{170}H_{184}N_{14}NaO_{34}]^+$**	**2988.30 (2989.30)**	**2988.06 (2990.02)**	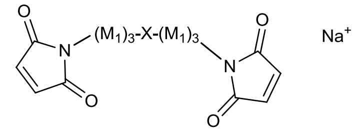
$[C_{174}H_{190}N_{14}NaO_{35}]^+$	3058.34 (3059.34)	3058.08 (3060.12)	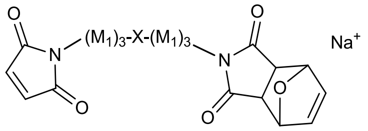
		**ESI-MS**	
**$[C_{178}H_{192}N_{14}Na_2O_{36}]^{2+}$**	**1573.6704**	**1573.6724**	**$(M_1)_3$-X-$(M_1)_3$, 2Na$^+$**
$[C_{178}H_{192}N_{14}K_2O_{36}]^{2+}$	1589.6443	1589.6637	$(M_1)_3$-X-$(M_1)_3$, 2K$^+$
$[C_{180}H_{196}N_{14}F_3Na_2O_{38}]^{2+}$	1608.6848	1608.6910	$(M_1)_3$-X-$(M_1)_3$, TFA, 2H$^+$
$[C_{180}H_{192}N_{14}F_3Na_3O_{38}]^{2+}$	1641.6578	1641.3667	$(M_1)_3$-X-$(M_1)_3$, NaTFA, 2Na$^+$
$[C_{182}H_{194}N_{14}F_6Na_4O_{40}]^{2+}$	1710.6530	1710.6568	$(M_1)_3$-X-$(M_1)_3$, 2NaTFA, 2Na$^+$

Supplementary Table 9. Identification of ions observed during the mass-spectrometric characterization (MALDI–ToF and ESI-MS) of **7c**.

Formula	*m/z* th.	*m/z* exp.	Assignment
	**MALDI–ToF**		
**$[C_{52}H_{56}N_4NaO_{10}]^+$**	**919.389**	**919.459**	**A_1, Na$^+$**
$[C_{56}H_{59}N_4O_9]^+$	931.428	932.531	A_2, H$^+$
$[C_{52}H_{56}KN_4O_{10}]^+$	935.363	935.413	A_1, K$^+$
$[C_{56}H_{60}N_4O_{10}]^+$	948.430	948.652	A_3^+
$[C_{56}H_{60}N_4NaO_{11}]^+$	987.415	987.501	(M_1M_1), Na$^+$
	**ESI-MS**		
$[C_{54}H_{62}N_4NaO_{11}]^+$	965.4307	965.4320	A_4, Na$^+$
$[C_{57}H_{63}N_4NaO_{11}]^+$	979.4488	979.4492	A_5^+
**$[C_{56}H_{60}N_4NaO_{11}]^+$**	**987.4151**	**987.4163**	**(M_1M_1), Na$^+$**
$[C_{56}H_{60}N_4NaO_{11}]^+$	1033.4569	1033.457	A_6, Na$^+$

Supplementary Table 10. Identification of ions observed during the mass-spectrometric characterization (MALDI–ToF and ESI-MS) of **7d**. The identified molecules are represented in Supplementary Fig. 62 (**A_1**).

Formula	*m/z* th.	*m/z* exp.	Assignment
		**MALDI–ToF**	
**$[C_{274}H_{296}N_{22}NaO_{54}]^+$**	**4785.13 (4787.14)**	**- (4787.39)**	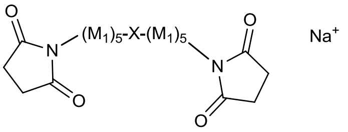
$[C_{274}H_{300}N_{22}KO_{54}]^+$	4801.10 (4804.11)	- (4803.64)	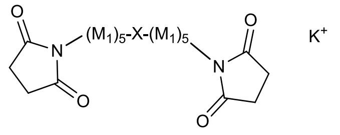
$[C_{278}H_{304}N_{22}NaO_{55}]^+$	4853.16 (4856.16)	- (4857.69)	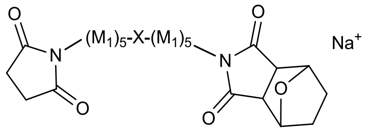
$[C_{278}H_{304}N_{22}KO_{55}]^+$	4869.13 (4872.14)	- (4872.04)	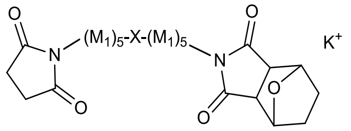
$[C_{282}H_{304}N_{22}Na_2O_{56}]^+$	4940.14 (4943.15)	- (4943.63)	$(M_1)_5$-X-$(M_1)_5$, Na-Na$^+$
$[C_{282}H_{304}N_{22}NaKO_{56}]^+$	4956.11 (4959.13)	- (4959.16)	$(M_1)_5$-X-$(M_1)_5$, Na-K$^+$
		**ESI-MS**	
$[C_{282}H_{304}N_{22}NaO_{56}]^{2+}$	2470.0701	2470.0905	$(M_1)_5$-X-$(M_1)_5$, Na$^+$

Supplementary Table 11. Identification of ions observed during the mass-spectrometric characterization (MALDI–ToF and ESI-MS) of **7**.

MS-Characterization of Oligomer 8 and Precursor 8a

Formula	*m/z* th.	*m/z* exp.	Assignment
		**MALDI–ToF**	
**$[C_{172}H_{184}N_{14}NaO_{38}]^+$**	**3076.28 (3077.28)**	**3076.20 (3078.14)**	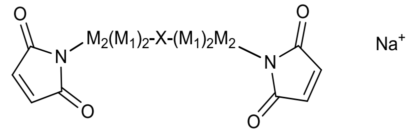
$[C_{176}H_{190}N_{14}NaO_{39}]^+$	3146.32 (3147.32)	3146.22 (31478.18)	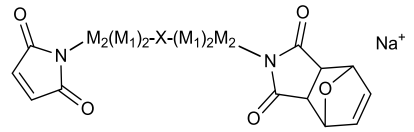
		**ESI-MS**	
**$[C_{180}H_{192}N_{14}Na_2O_{40}]^{2+}$**	**1617.6602**	**1617.6652**	**$(M_2)(M_1)_2$-X-$(M_1)_2(M_2)$, 2Na$^+$**
$[C_{182}H_{194}N_{14}F_3Na_2O_{42}]^{2+}$	1652.6757	1652.6982	$(M_2)(M_1)_2$-X-$(M_1)_2(M_2)$, TFA, 2H$^+$
$[C_{182}H_{192}N_{14}F_3Na_3O_{42}]^{2+}$	1685.6487	1685.6631	$(M_2)(M_1)_2$-X-$(M_1)_2(M_2)$, NaTFA, 2Na$^+$
$[C_{184}H_{195}N_{14}F_6NaO_{42}]^{2+}$	1720.6621	1720.6869	$(M_2)(M_1)_2$-X-$(M_1)_2(M_2)$, 2TFA, H$^+$, Na$^+$

Supplementary Table 12. Identification of ions observed during the mass-spectrometric characterization (MALDI–ToF and ESI-MS) of **8a**.

Formula	*m/z* th.	*m/z* exp.	Assignment
		**MALDI–ToF**	
**$[C_{276}H_{300}N_{22}NaO_{58}]^{+}$**	**4873.11 (4875.12)**	**- (4875.28)**	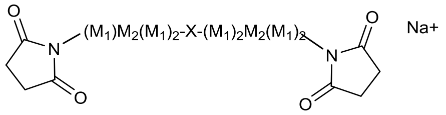
$[C_{276}H_{300}N_{22}KO_{58}]^{+}$	4889.08 (4891.09)	- (4890.09)	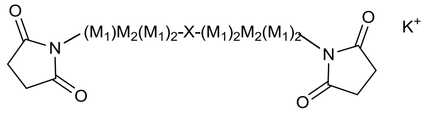
$[C_{280}H_{304}N_{22}NaO_{59}]^{+}$	4957.11 (4944.15)	- (4946.03)	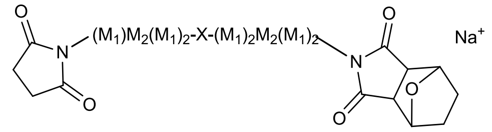
$[C_{280}H_{304}N_{22}KO_{59}]^{+}$	4869.13 (4872.14)	- (4959.90)	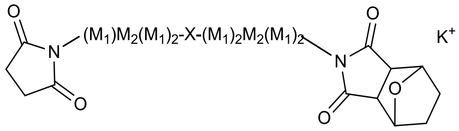
$[C_{284}H_{304}N_{22}Na_2O_{60}]^{+}$	5028.12 (5030.13)	- (5030.71)	$(M_1)_2(M_2)(M_1)_2$-X-$(M_1)_2(M_2)(M_1)_2$, Na-Na^+
$[C_{284}H_{304}N_{22}NaKO_{60}]^{+}$	5044.09 (5046.10)	- (5046.68)	$(M_1)_2(M_2)(M_1)_2$-X-$(M_1)_2(M_2)(M_1)_2$, Na-K^+
		**ESI-MS**	
$[C_{284}H_{304}N_{22}Na_2O_{60}]^{2+}$	2514.0599	2514.0870	$(M_1)_2(M_2)(M_1)_2$-X-$(M_1)_2(M_2)(M_1)_2$, $2Na^+$

Supplementary Table 13. Identification of ions observed during the mass-spectrometric characterization (MALDI–ToF and ESI-MS) of **8**.

MS-Characterization of Oligomer 9 and Precursors 9a-9c

Formula	*m/z* th.	*m/z* exp.	Assignment
		**MALDI–ToF**	
$[C_{120}H_{128}N_{10}NaO_{28}]^{+}$	2179.62 (2180.64)	2179.74 (2180.72)	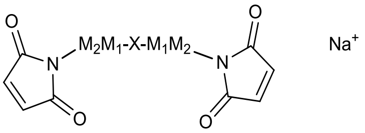
		**ESI-MS**	
$[C_{124}H_{132}N_{10}NaO_{29}]^{+}$	2247.9054	2247.9119	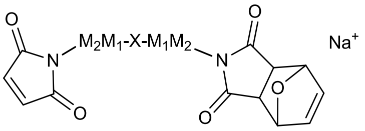
**$[C_{128}H_{136}N_{10}NaO_{30}]^{+}$**	**2315.9316**	**2315.9396**	**(M_2M_1)-X-(M_1M_2), Na^+**
$[C_{256}H_{272}N_{20}Na_2O_{60}]^{2+}$	2315.9316	2315.9396	2[(M_2M_1)-X-(M_1M_2)], $2Na^+$

Supplementary Table 14. Identification of ions observed during the mass-spectrometric characterization (MALDI–ToF and ESI-MS) of **9a**.

Formula	*m/z* th.	*m/z* exp.	Assignment
		**MALDI–ToF**	
$[C_{172}H_{184}N_{14}NaO_{38}]^+$	3076.28 (3077.28)	3076.20 (3078.05)	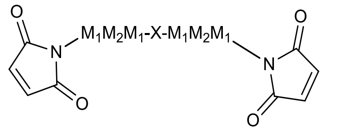 Na^+
		**ESI-MS**	
$\mathbf{[C_{176}H_{188}N_{14}Na_2O_{39}]^{2+}}$	**1583.6471**	**1583.658**	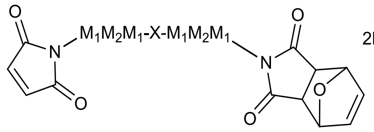 $2Na^+$
$[C_{180}H_{193}N_{14}NaO_{40}]^{2+}$	1606.6693	1606.6936	$(M_1M_2M_1)$-X-$(M_1M_2M_1)$, H^+, Na^+
$[C_{180}H_{192}N_{14}Na_2O_{40}]^{2+}$	1617.6602	1617.6679	$(M_1M_2M_1)$-X-$(M_1M_2M_1)$, $2Na^+$
$[C_{182}H_{192}N_{14}F_3Na_3O_{42}]^{2+}$	1685.6476	1685.1830	$(M_1M_2M_1)$-X-$(M_1M_2M_1)$, NaTFA, $2Na^+$

Supplementary Table 15. Identification of ions observed during the mass spectrometric characterization (MALDI–ToF and ESI-MS) of **9b**.

Formula	*m/z* th.	*m/z* exp.	Assignment
	**MALDI–ToF**		
$\mathbf{[C_{53}H_{56}N_4NaO_{12}]^+}$	**963.379**	**963.583**	**A_1, Na^+**
$[C_{56}H_{56}KN_4O_{12}]^+$	979.353	979.587	A_1, K^+
$[C_{57}H_{60}N_4O_{12}]^+$	992.420	992.612	A_2^+
	**ESI-MS**		
$\mathbf{[C_{57}H_{61}N_4O_{13}]^+}$	**1009.4230**	**1009.4289**	**(M_1M_2), H^+**
$[C_{57}H_{60}N_4NaO_{13}]^+$	1031.4049	1031.4116	(M_1M_2), Na^+
$[C_{59}H_{66}N_4NaO_{14}]^+$	1077.4468	1077.4535	A_3, Na^+

Supplementary Table 16. Identification of ions observed during the mass spectrometric characterization (MALDI–ToF and ESI-MS) of **9c**. The identified molecules are represented in Supplementary Fig. 88 (**A_1-A_3**).

Formula	*m/z* th.	*m/z* exp.	Assignment
		**MALDI–ToF**	
$\mathbf{[C_{278}H_{300}N_{22}NaO_{62}]^+}$	**4961.09 (4964.10)**	**- (4964.34)**	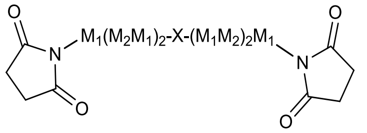 Na^+
$[C_{278}H_{300}N_{22}KO_{62}]^+$	4977.06 (4980.07)	- (4979.90)	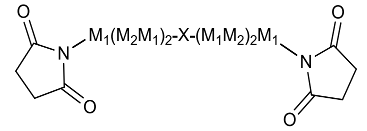 K^+
		**ESI-MS**	
$[C_{286}H_{304}N_{22}Na_2O_{64}]^{2+}$	2558.0497	2558.0698	$(M_1)(M_2M_1)_2$-X-$(M_1M_2)_2(M_1)$, $2Na^+$

Supplementary Table 17. Identification of ions observed during the mass spectrometric characterization (MALDI–ToF and ESI-MS) of **9**.

MS-Characterization of Oligomer 10 and Precursors 10a-10d

Formula	*m/z* th.	*m/z* exp.	Assignment
		**MALDI–ToF**	
$[C_{68}H_{72}N_6NaO_{18}]^+$	1283.480	1283.458	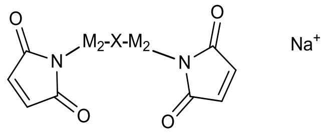
		**ESI-MS**	
$[C_{72}H_{76}N_6NaO_{19}]^+$	1351.5058	1351.5117	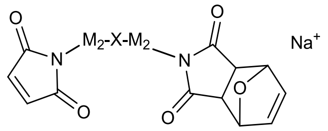
**$[C_{76}H_{80}N_6NaO_{20}]^+$**	**1419.5320**	**1419.5383**	**(M_2)-X-(M_2), Na^+**
$[C_{76}H_{80}N_6KO_{20}]^+$	1435.5059	1435.5112	(M_2)-X-(M_2), K^+
$[C_{78}H_{80}FN_6KNa_2O_{22}]^+$	1555.5068	1555.5116	(M_2)-X-(M_2), NaTFA, Na^+

Supplementary Table 18. Identification of ions observed during the mass-spectrometric characterization (MALDI–ToF and ESI-MS) of **10a**.

Formula	*m/z* th.	*m/z* exp.	Assignment
		**MALDI–ToF**	
$[C_{120}H_{128}N_{10}NaO_{28}]^+$	2179.62 (2180.64)	2179.88 (2180.88)	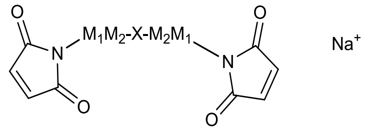
		**ESI-MS**	
$[C_{124}H_{132}N_{10}NaO_{29}]^+$	2247.9054	2247.9068	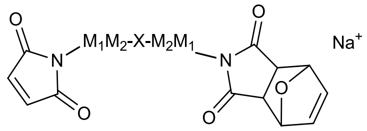
**$[C_{128}H_{136}N_{10}NaO_{30}]^+$**	**2315.9316**	**2315.9315**	**(M_1M_2)-X-(M_2M_1), Na^+**
$[C_{256}H_{272}N_{20}Na_2O_{60}]^{2+}$	2315.9316	2315.9315	2[(M_1M_2)-X-(M_2M_1)], $2Na^+$
$[C_{258}H_{272}N_{20}F_3Na_3O_{62}]^{2+}$	2384.4207	2384.4229	2[(M_1M_2)-X-(M_2M_1)], NaTFA, $2Na^+$
$[C_{260}H_{272}N_{20}F_6Na_4O_{64}]^{2+}$	2451.9064	2451.9110	2[(M_1M_2)-X-(M_2M_1)], 2NaTFA, $2Na^+$

Supplementary Table 19. Identification of ions observed during the mass spectrometric characterization (MALDI–ToF and ESI-MS) of **10b**.

Formula	*m/z* th.	*m/z* exp.	Assignment
		**MALDI–ToF**	
**$[C_{174}H_{184}N_{14}NaO_{42}]^+$**	**3164.26 (3166.26)**	**3164.30 (3166.22)**	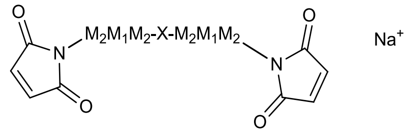
$[C_{174}H_{184}N_{14}KO_{42}]^+$	3180.23 (3182.24)	- (3182.93)	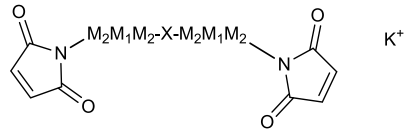
		**ESI-MS**	
$[C_{174}H_{184}N_{14}Na_2O_{42}]^{2+}$	1593.6238	1593.6243	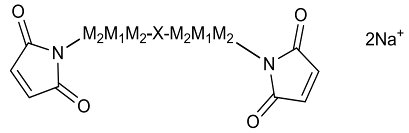
$[C_{178}H_{188}N_{14}Na_2O_{43}]^{2+}$	1627.6397	1627.6386	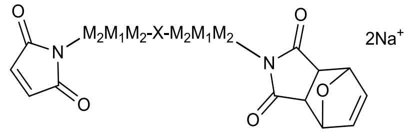
**$[C_{174}H_{184}N_{14}Na_2O_{44}]^{2+}$**	**1661.6501**	**1661.6630**	**$(M_2M_1M_2)$-X-$(M_2M_1M_2)$, $2Na^+$**
$[C_{184}H_{195}N_{14}F_3O_{46}]^{2+}$	1696.6646	1696.6838	$(M_2M_1M_2)$-X-$(M_2M_1M_2)$, TFA, $2H^+$
$[C_{184}H_{192}N_{14}F_3Na_3O_{46}]^{2+}$	1729.6375	1729.6449	$(M_2M_1M_2)$-X-$(M_2M_1M_2)$, NaTFA, $2Na^+$

Supplementary Table 20. Identification of ions observed during the mass spectrometric characterization (MALDI–ToF and ESI-MS) of **10c**.

Formula	*m/z* th.	*m/z* exp.	Assignment
		**MALDI–ToF**	
**$[C_{53}H_{56}N_4NaO_{12}]^+$**	**963.379**	**963.637**	**A_1, Na^+**
$[C_{53}H_{56}KN_4O_{12}]^+$	979.353	979.614	A_1, K^+
$[C_{57}H_{60}N_4O_{12}]^+$	992.420	992.695	A_2^+
		**ESI-MS**	
$[C_{57}H_{61}N_4O_{13}]^+$	1009.4230	1009.4231	(M_2M_1), H^+
**$[C_{57}H_{60}N_4NaO_{13}]^+$**	**1031.4049**	**1031.4067**	**(M_2M_1), Na^+**
$[C_{57}H_{60}KN_4O_{13}]^+$	1047.3788	1047.4019	(M_2M_1), K^+
$[C_{59}H_{61}N_4O_{13}]^+$	1077.4468	1077.4501	A_3, Na^+

Supplementary Table 21. Identification of ions observed during the mass-spectrometric characterization (MALDI–ToF and ESI-MS) of **10d**. The identified molecules are represented in Supplementary Fig. 113 (**A_1**-**A_3**).

Formula	*m/z* th.	*m/z* exp.	Assignment
		**MALDI–ToF**	
**$[C_{280}H_{300}N_{22}NaO_{66}]^{+}$**	**5049.07 (5052.08)**	**- (5052.22)**	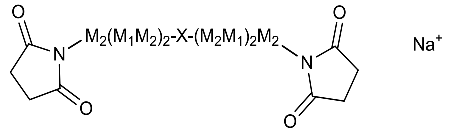
$[C_{280}H_{300}N_{22}KO_{66}]^{+}$	5065.04 (5068.05)	- (5068.05)	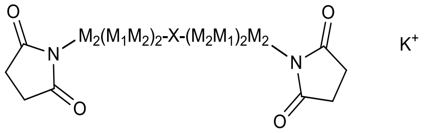
$[C_{284}H_{304}N_{22}KO_{67}]^{+}$	5117.09 (5120.11)	- (5122.87)	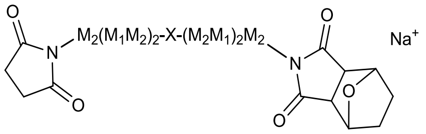
$[C_{284}H_{304}N_{22}KO_{67}]^{+}$	5133.07 (5136.08)	- (5139.56)	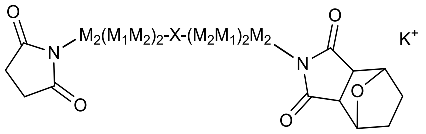
$[C_{288}H_{304}N_{22}Na_2O_{68}]^{+}$	5204.08 (5207.09)	- (5207.34)	$(M_2)(M_1M_2)_2$-X-$(M_2M_1)_2(M_2)$, Na-Na^{+}
$[C_{288}H_{304}N_{22}KNaO_{68}]^{+}$	5220.05 (5223.06)	- (5223.94)	$(M_2)(M_1M_2)_2$-X-$(M_2M_1)_2(M_2)$, Na-K^{+}
		**ESI-MS**	
$[C_{288}H_{304}N_{22}Na_2O_{68}]^{2+}$	2602.0395	2602.0512	$(M_2)(M_1M_2)_2$-X-$(M_2M_1)_2(M_2)$, $2Na^{+}$
$[C_{576}H_{608}N_{44}Na_4O_{136}]^{2+}$	2602.0395	2602.0512	2[$(M_2)(M_1M_2)_2$-X-$(M_2M_1)_2(M_2)$, Na], $2Na^{+}$

Supplementary Table 22. Identification of ions observed during the mass-spectrometric characterization (MALDI–ToF and ESI-MS) of **10**.

MS-Characterization of Oligomer 11 and Precursors 11a-11b

Formula	*m/z* th.	*m/z* exp.	Assignment
	**MALDI–ToF**		
**$[C_{140}H_{161}N_{12}O_{32}]^{+}$**	**2522.13 (2523.14)**	**2522.57 (2523.62)**	**A_1, H^{+}**
$[C_{140}H_{161}N_{12}O_{33}]^{+}$	2538.13 (2539.13)	2538.62 (2539.59)	A_1-OH, H^{+}
	**ESI-MS**		
$[C_{138}H_{157}F_3N_{12}Na_2O_{33}]^{2+}$	1306.5356	1306.5400	A_2, TFA, $2Na^{+}$
$[C_{142}H_{157}F_3N_{12}Na_2O_{36}]^{2+}$	1331.5182	1331.5458	A_3
**$[C_{142}H_{161}F_3N_{12}Na_2O_{34}]^{2+}$**	**1340.5487**	**1340.5551**	**A_1, TFA, $2Na^{+}$**
$[C_{142}H_{161}F_3N_{12}K_2O_{34}]^{2+}$	1357.5305	1357.5314	A_1, TFA, $2K^{+}$

Supplementary Table 23. Identification of ions observed during the mass-spectrometric characterization (MALDI–ToF and ESI-MS) of **11a**. The identified molecules are represented in Supplementary Fig. 121 (**A_1**-**A_3**).

Formula	m/z th.	m/z exp.	Assignment
		MALDI–ToF	
**$[C_{70}H_{75}N_6NaFO_{12}]^+$**	**1233.53**	**1234.06**	**A_1, Na^+**
$[C_{70}H_{75}N_6KFO_{12}]^+$	1249.51	1250.04	A_1, K^+
$[C_{52}H_{56}KN_4O_{14}]^+$	1266.52	1266.06	A_1-OH, K^+
		ESI-MS	
**$[C_{74}H_{79}N_6NaFO_{13}]^+$**	**1301.5581**	**1301.5620**	**(M_6M_5), Na^+**
$[C_{74}H_{79}N_6KFO_{13}]^+$	1317.5321	1317.5615	(M_6M_5), K^+
$[C_{72}H_{76}N_6NaF_4O_{14}]^+$	1347.5248	1347.6006	A_1, TFA, Na^+
$[C_{76}H_{79}N_6Na_2F_4O_{15}]^+$	1437.5329	1437.5425	(M_6M_5), NaTFA, Na^+

Supplementary Table 24. Identification of ions observed during the mass spectrometric characterization (MALDI–ToF and ESI-MS) of **11b**. The identified molecules are represented in Supplementary Fig. 127 (**A_1**).

Formula	m/z th.	m/z exp.	Assignment
		MALDI–ToF	
**$[C_{276}H_{306}F_2N_{24}KO_{57}]^+$**	**4945.14 (4948.15)**	**- (4949.48)**	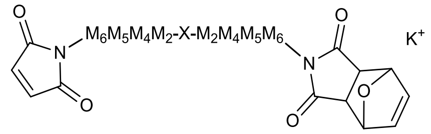
$[C_{276}H_{308}F_2KN_{24}O_{58}]^+$	4963.15 (4965.16)	- (4965.58)	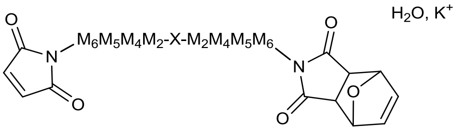
		ESI-MS	
$[C_{282}H_{310}F_5N_{24}KO_{60}]^{2-}$	2563.0754	2563.0762	$[(M_6M_5M_4M_2)$-X-$(M_2M_4M_5M_6)$, K-TFA$]^{2-}$
$[C_{282}H_{311}F_5N_{24}KO_{60}]^{2-}$	2563.5784	2563.5718	$[(M_6M_5M_4M_2)$-X-$(M_2M_4M_5M_6)$, H-K-TFA$]^{2-}$

Supplementary Table 25. Identification of ions observed during the mass-spectrometric characterization (MALDI–ToF and ESI-MS) of **11**.

MS-Characterization of Oligomer 12 and Precursors 12a-12c

Formula	m/z th.	m/z exp.	Assignment
		MALDI–ToF	
**$[C_{52}H_{56}N_4NaO_{14}]^+$**	**983.37**	**983.69**	**A_1, Na^+**
$[C_{52}H_{56}KN_4O_{14}]^+$	999.34	999.67	A_1, K^+
		ESI-MS	
$[C_{56}H_{60}NaN_4O_{15}]^+$	1051.3947	1051.3957	(M_3M_3), Na^+

Supplementary Table 26. Identification of ions observed during the mass-spectrometric characterization (MALDI–ToF and ESI-MS) of **12a**. The identified molecules are represented in Supplementary Fig. 136 (**A_1**).

Formula	*m/z* th.	*m/z* exp.	Assignment
		**MALDI–ToF**	
$[C_{78}H_{84}N_6NaO_{20}]^+$	1447.56	1447.90	A_1, Na^+
**$[C_{78}H_{84}NaN_6O_{21}]^+$**	**1463.56**	**1463.85**	**A_2, Na^+**
		**ESI-MS**	
$[C_{78}H_{84}N_6NaO_{21}]^+$	1463.5582	1463.5572	A_2, Na^+
$[C_{82}H_{89}N_6O_{22}]^+$	1509.6024	1509.6097	$(M_3)_3$, H^+
**$[C_{82}H_{88}NaN_6O_{22}]^+$**	**1531.5844**	**1531.5918**	**$(M_3)_3$, Na^+**
$[C_{164}H_{176}Na_2N_{12}O_{44}]^{2+}$	1531.5844	1531.5918	$2[(M_3)_3]$, $2Na^+$
$[C_{82}H_{88}KN_6O_{22}]^+$	1547.5583	1547.5854	$(M_3)_3$, K^+
$[C_{84}H_{88}F_3N_6Na_2O_{24}]^+$	1667.5592	1667.5779	$(M_3)_3$, NaTFA, Na^+

Supplementary Table 27. Identification of ions observed during the mass spectrometric characterization (MALDI–ToF and ESI-MS) of **12b**. The identified molecules are represented in Supplementary Fig. 143 (**A_1**).

Formula	*m/z* th.	*m/z* exp.	Assignment
		**MALDI–ToF**	
**$[C_{130}H_{140}N_{10}NaO_{35}]^+$**	**2424.95<br>(2425.95)**	**2424.47<br>(2425.46)**	**A_1, Na^+**
$[C_{130}H_{140}N_{10}KO_{35}]^+$	2438.90<br>(2439.91)	2439.39<br>(2440.43)	A_1, K^+
$[C_{134}H_{145}N_{10}O_{36}]^+$	2469.98<br>(2470.99)	2468.41<br>(2467.45)	$(M_5)_5$, H^+
$[C_{134}H_{144}N_{10}O_{37}]^+$	2484.97<br>(2485.97)	2485.12<br>(2486.07)	$[(M_5)_5\text{-}OH]^+$

Supplementary Table 28. Identification of ions observed during the mass spectrometric characterization (MALDI–ToF and ESI-MS) of **12**. The identified molecules are represented in Supplementary Fig. 147 (**A_1**).

MALDI–ToF–ToF Mass-Spectrometry Characterization

Name	Ion	Formula	*m/z* th.	*m/z* exp.
	[Mal-$(M_1)_3$-X-$(M_1)_3$-Mal], K^+	$[C_{170}H_{184}KN_{14}O_{34}]^+$	3004.27 (3005.28)	3007.71
**M^{Na}**	[Mal-$(M_1)_3$-X-$(M_1)_3$-Mal], Na^+	$[C_{170}H_{184}N_{14}NaO_{34}]^+$	2988.30 (2989.30)	2988.96
	[Mal-$(M_1)_3$-X-$(M_1)_3$-Mal], H^+	$[C_{170}H_{185}N_{14}O_{34}]^+$	2966.31 (2967.32)	2967.50
	**1st order fragmentation**			
	[Mal-$(M_1)_2$-X-$(M_1)_3$-Mal], $4H_2O$, H^+	$[C_{144}H_{165}N_{12}O_{33}]^+$	2590.16 (2591.16)	2591.22
	[Mal-$(M_1)_2$-X-$(M_1)_3$-Mal], $3H_2O$, H^+	$[C_{144}H_{163}N_{12}O_{32}]^+$	2572.14 (2573.15)	2573.29
	[Mal-$(M_1)_2$-X-$(M_1)_3$-Mal], $2H_2O$, H^+	$[C_{144}H_{161}N_{12}O_{31}]^+$	2554.139 (2555.14)	2555.37
**1**	[Mal-$(M_1)_2$-X-$(M_1)_3$-Mal], H_3O^+	$[C_{144}H_{159}N_{12}O_3]^+$	2536.13 (2537.13)	2537.15
	[Mal-$(M_1)_2$-X-$(M_1)_3$-Mal], H^+	$[C_{144}H_{157}N_{12}O_{29}]^+$	2518.12 (2519.12)	2520.24
	**2nd order fragmentation**			
	[Mal-M_1-X-$(M_1)_3$-Fm], $2H_2O$, H^+	$[C_{122}H_{137}N_{10}O_{27}]^+$	2173.96 (2174.97)	2173.42
	[Mal-M_1-X-$(M_1)_3$-Fm], H_3O^+	$[C_{122}H_{135}N10O_{26}]^+$	2155.95 (2156.96)	2155.64
	[Mal-M_1-X-$(M_1)_3$-Fm], H^+	$[C_{122}H_{133}N_{10}O_{25}]^+$	2137.94 (2138.95)	2139.18
	[Mal-$(M_1)_2$-X-$(M_1)_2$-Mal], $2H_2O$, H^+	$[C_{118}H_{132}N_{10}O_{26}]^+$	2104.93 (2105.93)	2104.84
**2**	[Mal-$(M_1)_2$-X-$(M_1)_2$-Mal], H_3O^+	$[C_{118}H_{131}N_{10}O_{25}]^+$	2087.93 (2088.93)	2088.42
	[Mal-$(M_1)_2$-X-$(M_1)_2$-Mal], H^+	$[C_{118}H_{129}N_{10}O_{24}]^+$	2069.92 (2070.92)	2071.58
	**3rd order fragmentation, Route A**			
**3b**	3b	$[C_{100}H_{111}N_8O_{22}]^+$	1775.78 1776.78	1774.57
	**3rd order fragmentation, Route B**			
	[Mal-M_1-X-$(M_1)_2$-Mal], H^+, $4H_2O$	$[C_{92}H_{109}N_8O_{23}]^+$	1693.76 (1694.76)	1697.34
	[Mal-M_1-X-$(M_1)_2$-Mal], H^+, $3H_2O$	$[C_{92}H_{107}N_8O_{22}]^+$	1675.75 (1676.75)	1679.68
	[Mal-M_1-X-$(M_1)_2$-Mal], H^+, $2H_2O$	$[C_{92}H_{105}N_8O_{21}]^+$	1657.74 (1658.74)	1655.93
**3a**	[Mal-M_1-X-$(M_1)_2$-Mal], H_3O^+	$[C_{92}H_{103}N_8O_{20}]^+$	1639.73 (1640.73)	1640.86
	[Mal-M_1-X-$(M_1)_2$-Mal], H^+	$[C_{92}H_{101}N_8O_{19}]^+$	1621.72 (1622.72)	1621.948
	**4rd order fragmentation, Route B**			
	4b + H_2O	$[C_{74}H_{85}N_6O_{18}]^+$	1345.59	1342.40
**4b**	4b	$[C_{74}H_{83}N_6O_{17}]^+$	1327.58	1326.46
	4b - H2O	$[C_{74}H_{81}N_6O_{16}]^+$	1309.57	1309.57

	**4rd order fragmentation, Route A**			
	[Mal-M$_1$-X-M$_1$-Mal], 3H$_2$O, H$^+$	$[C_{66}H_{79}N_6O_{17}]^+$	1227.55	1228.60
	[Mal-M$_1$-X-M$_1$-Mal], 2H$_2$O, H$^+$	$[C_{66}H_{77}N_6O_{16}]^+$	1209.54	1209.79
**4a***	[Mal-M$_1$-X-M$_1$-Mal], H$_3$O$^+$	$[C_{66}H_{75}N_6O_{15}]^+$	1191.53	1192.72
	[Mal-M$_1$-X-M$_1$-Mal], H$^+$	$[C_{66}H_{73}N_6O_{14}]^+$	1173.52	1174.87
	**5th order fragmentation**			
	5b + H$_2$O	$[C_{49}H_{59}N_4NaO_{13}]^+$	934.40	933.26
**5b**	5b	$[C_{49}H_{57}N_4NaO_{12}]^+$	916.39	917.41
	5b -H$_3$O$^+$	$[C_{52}H_{58}N_4O_{10}]^+$	898.41	899.36
	[X-M$_1$-Mal], 2H$_2$O. H$^+$	$[C_{40}H_{49}N_4O_{11}]^+$	761.34	761.73
**5a**	[X-M$_1$-Mal], H$_3$O$^+$	$[C_{40}H_{47}N_4O_{10}]^+$	743.33	745.86
	**6th order fragmentation**			
**6b**	Monomer [M$_1$-Mal], Na$^+$	$[C_{26}H_{30}N_2O_5Na]^+$	473.20	470.33
**6a**	Core [X], Na$^+$	$[C_{14}H_{16}N_2O_4Na]^+$	299.10	299.01

(*) *m/z* (**4a**) is similar for [Mal-M$_1$-X-M$_1$-Mal] and [Mal-X-(M$_1$)$_2$-Mal] species.

Supplementary Table 29. Summary of the identified ions from the MALDI–ToF–ToF spectrum for compound **7c** (noted (M$_1$)$_3$-X-(M$_1$)$_3$). Ions terminated with maleimide end-groups are denoted "Mal" and those with a furan protected maleimide end-group "Fm". The theoretical *m/z* value is provided as exact mass as first entry, and the nominal mass in brackets (when not otherwise indicated, exact and nominal value are identical). The identified structures are depicted in Supplementary Fig. 150.

Name	Ion	Formula	*m/z* th.	*m/z* exp.
	[Mal-(M$_2$)(M$_1$)$_2$-X-(M$_1$)$_2$(M$_2$)-Mal], K$^+$	$[C_{172}H_{184}N_{14}O_{38}K]^+$	3092.25 (3093.26)	3094.44
**M^{Na}**	[Mal-(M$_2$)(M$_1$)$_2$-X-(M$_1$)$_2$(M$_2$)-Mal], Na$^+$	$[C_{172}H_{184}N_{14}O_{38}Na]^+$	3076.28 (3077.28)	3076.85
	[Mal-(M$_2$)(M$_1$)$_2$-X-(M$_1$)$_2$(M$_2$)-Mal], H$^+$	$[C_{172}H_{185}N_{14}O_{38}]^+$	3054.30 (3055.30)	3058.46
	**1st order fragmentation**			
	[Mal-(M$_1$)$_2$-X-(M$_1$)$_2$(M$_2$)-Mal], 4H$_2$O, H$^+$	$[C_{145}H_{165}N_{12}O_{35}]^+$	2634.15 (2636.15)	2636.76
	[Mal-(M$_1$)$_2$-X-(M$_1$)$_2$(M$_2$)-Mal], 3H$_2$O, H$^+$	$[C_{145}H_{163}N_{12}O_{34}]^+$	2616.14 (2617.14)	2619.95
	[Mal-(M$_1$)$_2$-X-(M$_1$)$_2$(M$_2$)-Mal], 2H$_2$O, H$^+$	$[C_{145}H_{161}N_{12}O_{33}]^+$	2598.13 (2599.13)	2597.16
**1**	[Mal-(M$_1$)$_2$-X-(M$_1$)$_2$(M$_2$)-Mal], H$_3$O$^+$	$[C_{145}H_{159}N_{12}O_{32}]^+$	2580.12 (2581.12)	2580.67
	[Mal-(M$_1$)$_2$-X-(M$_1$)$_2$(M$_2$)-Mal], H$^+$	$[C_{145}H_{157}N_{12}O_{31}]^+$	2562.11 (2563.11)	2562.50
	**2nd order fragmentation, Route A**			
	[Mal-M$_1$-X-(M$_1$)$_2$(M$_2$)-Fm], 2H$_2$O, H$^+$	$[C_{123}H_{137}N_{10}O_{29}]^+$	2217.95 (2218.96)	2219.83
	[Mal-M$_1$-X-(M$_1$)$_2$(M$_2$)-Fm], H$_3$O$^+$	$[C_{123}H_{135}N_{10}O_{28}]^+$	2199.94 (2200.95)	2202.38
	[Mal-M$_1$-X-(M$_1$)$_2$(M$_2$)-Mal], 2H$_2$O, H$^+$	$[C_{119}H_{133}N_{10}O_{28}]^+$	2149.93 (2150.93)	2147.80
**2a**	[Mal-M$_1$-X-(M$_1$)$_2$(M$_2$)-Mal], H$_3$O$^+$	$[C_{119}H_{131}N_{10}O_{27}]^+$	2131.92	2131.44

			(2132.92)	
	**2nd fragmentation, Route B**			
	[Mal-$(M_1)_2$-X-$(M_1)_2$-Mal], $2H_2O$, H^+	$[C_{118}H_{132}N_{10}O_{26}]^+$	2104.93 (2105.93)	2103.47
**2b**	[Mal-$(M_1)_2$-X-$(M_1)_2$-Mal], H_3O^+	$[C_{118}H_{131}N_{10}O_{25}]^+$	2087.93 (2088.93)	2086.63
	**3rd order fragmentation, Route A**			
	[X-$(M_1)_2(M_2)$-Mal], $2H_2O$, H^+	$[C_{93}H_{105}N_8O_{23}]^+$	1701.73	1700.09
**3a**	[X-$(M_1)_2(M_2)$-Mal], H_3O^+	$[C_{93}H_{103}N_8O_{22}]^+$	1683.72	1683.07
	[X-$(M_1)_2(M_2)$-Mal], H^+	$[C_{93}H_{101}N_8O_{21}]$+	1665.71	1665.06
	**3rd order fragmentation, Route B**			
**3b**	[Mal-M_1-X-$(M_1)_2$-Mal], H_3O^+	$[C_{92}H_{103}N_8O_{20}]^+$	1639.73	1639.09
	[Mal-M_1-X-$(M_1)_2$-Mal], H^+	$[C_{92}H_{101}N_8O_{19}]^+$	1621.72	1621.03
	**4rd order fragmentation, Route C**			
	4c, H_2O	$[C_{83}H_{93}N_6O_{19}]^+$	1477.65	1477.49
**4c**	4c	$[C_{83}H_{91}N_6O_{18}]^+$	1459.64	1461.42
	**4th order fragmentation, Route B**			
	4b4	$[C_{75}H_{83}N_6O_{17}]^+$	1339.58	1341.71
	4b3	$[C_{75}H_{83}N_6O_{16}]^+$	1323.59	1325.83
**4b2**	4b2	$[C_{71}H_{79}N_6O_{16}]^+$	1271.55	1271.88
**4b1**	4b1	$[C_{71}H_{78}N_6O_{15}]^+$	1254.55	1255.90
	**4th order fragmentation, Route A**			
	[Mal-X-$(M_1)_2$-Mal], $2H_2O$, H^+	$[C_{66}H_{77}N_6O_{16}]^+$	1209.54	1208.04
**4a2**	[Mal-X-$(M_1)_2$-Mal], H_3O^+	$[C_{66}H_{75}N_6O_{15}]^+$	1191.53	1192.03
	[Mal-X-$(M_1)_2$-Mal], H^+	$[C_{66}H73_3N_6O_{14}]^+$	1173.52	1174.07
	[Mal-X-$(M_1)_2$-Mal], $2H_2O$, H^+	$[C_{66}H_{77}N_6O_{16}]^+$	1209.54	1208.04
**4a1***	[Mal-M_1-X-M_1-Mal], H_3O^+	$[C_{66}H_{75}N_6O_{15}]^+$	1191.53	1192.03
	[Mal-M_1-X-M_1-Mal], H^+	$[C_{66}H73_3N_6O_{14}]^+$	1173.52	1174.07
	**5th order fragmentation**			
	5b + H_2O	$[C_{52}H_{62}N_4O_{12}]^+$	934.44	932.71
**5b**	5b	$[C_{52}H_{60}N_4O_{11}]^+$	916.43	916.87
	5b - H_2O	$[C_{52}H_{58}N_4O_{10}]^+$	898.41	898.92
	[Mal-X-M_1-Mal], H_3O^+	$[C_{40}H_{49}N_4O_{11}]^+$	761.34	761.26
**5a**	[Mal-X-M_1-Mal], H_3O^+	$[C_{40}H_{47}N_4O_{10}]^+$	743.33	745.24
	**6th order fragmentation**			
**6c**	Monomer [M_1-Mal], Na^+	$[C_{26}H_{30}N_2O_5Na]^+$	473.20	470.07
**6b**	Fragment M_2 Na^+	$[C_{15}H_{21N2}O_5Na]^+$	332.13	336.56
**6a**	Core [X], Na^+	$[C_{14}H_{16}N_2O_4Na]^+$	299.10	298.76

(*) *m/z* (**4a1**) = *m/z* (**4a2**)

Supplementary Table 30. Summary of the identified ions from the MALDI–ToF–ToF spectrum for compound **8a** (noted $(M_2)(M_1)_2$-X-$(M_1)_2(M_2)$). Ions terminated with maleimide end-groups are denoted “Mal” and with furan protected maleimide end-group “Fm”. The theoretical *m/z* value is provided as exact mass as first entry, and the nominal mass in brackets (when not otherwise indicated, exact and nominal value are identical). The identified structures are depicted in Supplementary Fig. 153.

Name	Ion	Formula	*m/z* th.	*m/z* exp.
	[Mal-$M_1M_2M_1$-X-$M_1M_2M_1$-Mal], K^+	$[C_{172}H_{184}KN_{14}O_{38}]^+$	3092.25 (3093.26)	3095.32
**M^{Na}**	[Mal-$M_1M_2M_1$-X-$M_1M_2M_1$-Mal], Na^+	$[C_{172}H_{184}N_{14}NaO_{38}]^+$	3076.28 (3077.28)	3077.62
	[Mal-$M_1M_2M_1$-X-$M_1M_2M_1$-Mal], H_3O^+	$[C_{172}H_{187}N_{14}O_{39}]^+$	3054.30 (3055.30)	3058.34
	**1st order fragmentation**			
	[Mal-M_2M_1-X-$M_1M_2M_1$-Mal], $4H_2O$, H^+	$[C_{146}H_{165}N_{12}O_{37}]^+$	2678.14 (2679.14)	2678.42
	[Mal-M_2M_1-X-$M_1M_2M_1$-Mal], $3H_2O$, H^+	$[C_{146}H_{163}N_{12}O_{36}]^+$	2660.13 (2661.13)	2661.53
	[Mal-M_2M_1-X-$M_1M_2M_1$-Mal], $2H_2O$, H^+	$[C_{146}H_{161}N_{12}O_{35}]^+$	2642.12 (2643.12)	2642.54
**1**	[Mal-M_2M_1-X-$M_1M_2M_1$-Mal], H_3O^+	$[C_{146}H_{159}N_{12}O_{34}]^+$	2624.11 (2625.11)	2625.20
	[Mal-M_2M_1-X-$M_1M_2M_1$-Mal], H^+	$[C_{146}H_{157}N_{12}O_{33}]^+$	2606.10 (2607.10)	2607.86
	**2nd order fragmentation, Route A**			
**2a**	[Mal-M_2M_1-X-M_1M_2-Mal], H_3O^+	$[C_{120}H_{131}N_{10}O_{29}]^+$	2175.91 (2176.91)	2175.92
	**2nd order fragmentation, Route B**			
	[Mal-M_1-X-$M_1M_2M_1$-Fm], $2H_2O$, H^+	$[C_{123}H_{137}N_{10}O_{29}]^+$	2217.95 (2218.96)	2221.11
	[Mal-M_1-X-$M_1M_2M_1$-Fm], H_3O^+	$[C_{123}H_{135}N_{10}O_{28}]^+$	2199.94 (2200.95)	2202.74
	[Mal-M_1-X-$M_1M_2M_1$-Mal], $4H_2O$, H^+	$[C_{119}H_{137}N_{10}O_{30}]^+$	2185.95 (2186.95)	2185.24
	[Mal-M_1-X-$M_1M_2M_1$-Mal], $2H_2O$, H^+	$[C_{119}H_{133}N_{10}O_{28}]^+$	2149.93 (2150.93)	2148.55
**2b**	[Mal-M_1-X-$M_1M_2M_1$-Mal], H_3O^+	$[C_{119}H_{131}N_{10}O_{27}]^+$	2131.92 (2132.92)	2131.96
	[Mal-M_1-X-$M_1M_2M_1$-Mal], H^+	$[C_{119}H_{129}N_{10}O_{26}]^+$	2113.91 (2214.91)	2115.22
	**3rd order fragmentation, Route A**			
	[Mal-M_1-X-M_1M_2-Mal], $4H_2O$, H^+	$[C_{93}H_{109}N_8O_{25}]^+$	1737.75 (1738.75)	1740.64
	[Mal-M_1-X-M_1M_2-Mal], $3H_2O$, H^+	$[C_{93}H_{107}N_8O_{24}]^+$	1719.74 (1720.74)	1724.51
	[Mal-M_1-X-M_1M_2-Mal], $2H_2O$, H^+	$[C_{93}H_{105}N_8O_{23}]^+$	1701.73 (1702.73)	1700.55
**3a**	[Mal-M_1-X-M_1M_2-Mal], H_3O^+	$[C_{93}H_{103}N_8O_{22}]^+$	1683.72 (1684.72)	1684.52
	[Mal-M_1-X-M_1M_2-Mal], H^+	$[C_{93}H_{101}N_8O_{21}]^+$	1665.71 (1666.71)	1666.55
	**3rd order fragmentation, Route B**			
	[X-$M_1M_2M_1$-Mal], $4H_2O$, H^+	$[C_{93}H_{109}N_8O_{25}]^+$	1737.75 (1738.75)	1740.64
	[X-$M_1M_2M_1$-Mal], $3H_2O$, H^+	$[C_{93}H_{107}N_8O_{24}]^+$	1719.74 (1720.74)	1724.51
	[X-$M_1M_2M_1$-Mal], $2H_2O$, H^+	$[C_{93}H_{105}N_8O_{23}]^+$	1701.73 (1702.73)	1700.55

**3b***	[X-$M_1M_2M_1$-Mal], H_3O^+	$[C_{93}H_{103}N_8O_{22}]^+$	1683.72 (1684.72)	1684.52
	[X-$M_1M_2M_1$-Mal], H^+	$[C_{93}H_{101}N_8O_{21}]^+$	1665.71 (1666.71)	1666.55
	**3rd order fragmentation, Route C**			
	3c2 + H_2O	$[C_{102}H_{113}N_8O_{24}]^+$	1833.79 (1834.79)	1834.17
**3c2**	3c2	$[C_{102}H_{111}N_8O_{23}]^+$	1815.78 (1816.78)	1818.26
**3c1**	3c1	$[C_{101}H_{109}N_8O_{23}]^+$	1801.76 (1802.76)	1801.65
	**4th order fragmentation, Route A**			
	[Mal-M_1-X-M_1-Mal], $2H_2O$, H^+	$[C_{66}H_{77}N_6O_{16}]^+$	1209.54	1208.75
**4a**	[Mal-M_1-X-M_1-Mal], H_3O^+	$[C_{66}H_{75}N_6O_{15}]^+$	1191.53	1192.57
	**4th order fragmentation, Route B**			
	[X-M_1M_2-Mal], $3H_2O$, H^+	$[C_{67}H_{79}N_6O_{19}]^+$	1271.54	1272.41
	[X-M_1M_2-Mal], $2H_2O$, H^+	$[C_{67}H_{77}N_6O_{18}]^+$	1253.53	1252.46
**4b**	[X-M_1M_2-Mal], H_3O^+	$[C_{67}H_{75}N_6O_{17}]^+$	1235.52	1236.43
	[X-M_1M_2-Mal], H^+	$[C_{67}H_{73}N_6O_{16}]^+$	1217.51	1218.38
	**5th order fragmentation**			
	5c + H_2O	$[C_{53}H_{63}N_4O_{14}]^+$	979.43	977.10
**5c**	5c	$[C_{53}H_{61}N_4O_{13}]^+$	961.42	961.08
	5c - H_2O	$[C_{53}H_{59}N_4O_{12}]^+$	943.41	943.19
	5b + $2H_2O$	$[C_{45}H_{57}N_4O_{14}]^+$	877.39	879.18
	5b + H_2O	$[C_{45}H_{55}N_4O_{13}]^+$	859.38	851.32
**5b**	5b	$[C_{45}H_{53}N_4O_{12}]^+$	841.37	841.35
	5b - H_2O	$[C_{45}H_{51}N_4O_{11}]^+$	823.35	825.46
	[X-M_1-Mal], $2H_2O$, H^+	$[C_{40}H_{49}N_4O_{11}]^+$	761.34	761.62
**5a**	[X-M_1-Mal], H_3O^+	$[C_{40}H_{47}N_4O_{10}]^+$	743.33	745.67
	[X-M_1-Mal], H^+	$[C_{40}H_{45}N_4O_9]^+$	725.32	728.04
	**6th order fragmentation**			
**6d**	Monomer [M_2-Mal], Na^+	$[C_{27}H_{30}N_2NaO_7]^+$	517.19	514.27
**6c**	Monomer [M_1-Mal], Na^+	$[C_{26}H_{30}N_2NaO_5]^+$	473.20	470.35
**6b**	Fragment M_2 Na^+	$[C_{15}H_{21}N_2NaO_5]^+$	332.13	336.86
**6a**	Core [X], Na^+	$[C_{14}H_{16}N_2O_4Na]^+$	299.10	299.00

(*) *m/z* (**3a**) = *m/z* (**3b**)

Supplementary Table 31. Summary of the identified ions from the MALDI–ToF–ToF spectrum for compound **9b** (noted ($M_1M_2M_1$)-X-($M_1M_2M_1$)). Ions terminated with maleimide end-groups are denoted "Mal" and with furan protected maleimide end-group "Fm". The theoretical *m/z* value is provided as exact mass as first entry, and the nominal mass in brackets (when not otherwise indicated, exact and nominal value are identical). The identified structures are depicted in Supplementary Fig. 156.

Name	Ion	Formula	*m/z* th.	*m/z* exp.
	[Mal-$M_2M_1M_2$-X-$M_2M_1M_2$-Mal], K^+	$[C_{174}H_{184}N_{14}KO_{42}]^+$	3180.23 (3181.24)	3183.73
**M^{Na}**	[Mal-$M_2M_1M_2$-X-$M_2M_1M_2$-Mal], Na^+	$[C_{174}H_{184}N_{14}NaO_{42}]^+$	3164.26 (3165.26)	3166.08

	[Mal-$M_2M_1M_2$-X-$M_2M_1M_2$-Mal], H^+	$[C_{174}H_{185}N_{14}O_{42}]^+$	3142.27 (3143.28)	3148.69
	**1st order fragmentation**			
	[Mal-M_1M_2-X-$M_2M_1M_2$-Mal], $4H_2O$, H^+	$[C_{147}H_{165}N_{12}O_{39}]^+$	2722.13 (2723.13)	2727.08
	[Mal-M_1M_2-X-$M_2M_1M_2$-Mal], $3H_2O$, H^+	$[C_{147}H_{163}N_{12}O_{38}]^+$	2704.12 (2705.12)	2710.30
	[Mal-M_1M_2-X-$M_2M_1M_2$-Mal], $2H_2O$, H^+	$[C_{147}H_{161}N_{12}O_{37}]^+$	2686.11 (2687.11)	2686.73
**1**	[Mal-M_1M_2-X-$M_2M_1M_2$-Mal], H_3O+	$[C_{147}H_{159}N_{12}O_{36}]^+$	2668.10 (2669.10)	2670.41
	[Mal-M_1M_2-X-$M_2M_1M_2$-Mal], H^+	$[C_{147}H_{157}N_{12}O_{35}]^+$	2650.09 (2651.09)	2652.926
	**2nd order fragmentation, Route A**			
**2a**	[Mal-M_1M_2-X-M_2M_1-Mal], H_3O^+	$[C_{120}H_{131}N_{10}O_{29}]^+$	2193.92 (2194.92)	2192.01
	[Mal-M_1M_2-X-M_2M_1-Mal], H^+	$[C_{120}H_{133}N_{10}O_{30}]^+$	2175.91 (2176.91)	2175.74
	**2nd order fragmentation, Route B**			
	[Mal-M_2-X-$M_2M_1M_2$-Fm], K^+	$[C_{125}H_{132}N_{10}KO_{31}]^+$	2307.87 (2308.87)	2309.33
	[Mal-M_2-X-$M_2M_1M_2$-Fm], Na^+	$[C_{125}H_{132}N_{10}NaO_{31}]^+$	2291.90 (2292.90)	2291.58
	[Mal-M_2-X-$M_2M_1M_2$-Mal], $4H_2O$, H^+	$[C_{121}H_{137}N_{10}O_{34}]^+$	2273.93 (2274.93)	2274.72
	[Mal-M_2-X-$M_2M_1M_2$-Mal], $2H_2O$, H^+	$[C_{121}H_{133}N_{10}O_{32}]^+$	2237.91 (2238.91)	2227.08
**2b**	[Mal-M_2-X-$M_2M_1M_2$-Mal], H_3O^+	$[C_{121}H_{131}N_{10}O_{31}]^+$	2219.90 (2220.90)	2220.49
	[Mal-M_2-X-$M_2M_1M_2$-Mal], H^+	$[C_{121}H_{129}N_{10}O_{30}]^+$	2201.89 2202.89	2203.16
	**3rd order fragmentation, Route A**			
	[Mal-M_2-X-M_2M_1-Mal], $2H_2O$, H^+	$[C_{94}H_{105}N_8O_{25}]^+$	1745.72 (1746.72)	1745.01
**3a**	[Mal-M_2-X-M_2M_1-Mal], H_3O^+	$[C_{94}H_{103}N_8O_{24}]^+$	1727.71 (1728.71)	1728.87
	[Mal-M_2-X-M_2M_1-Mal], H^+	$[C_{94}H_{100}N_8O_{23}]^+$	1708.69 (1709.62294)	1710.06
	**3rd order fragmentation, Route B**			
	3b1, H_2O	$[C_{103}H_{113}N_8O_{26}]^+$	1877.78 (1878.78)	1878.81
**3b1**	3b1	$[C_{103}H_{111}N_8O_{25}]^+$	1859.76 (1860.77)	1862.76
**3b2**	3b2	$[C_{102}H_{108}N_8O_{25}]^+$	1844.74 (1845.75)	1845.79
	**3rd order fragmentation, Route C**			
	[X-$M_2M_1M_2$-Mal], $2H_2O$, H^+	$[C_{94}H_{105}N_8O_{25}]^+$	1745.72 (1746.72)	1745.01
**3c***	[X-$M_2M_1M_2$-Mal], H_3O^+	$[C_{94}H_{103}N_8O_{24}]^+$	1727.71 (1728.71)	1728.87

	[X-$M_2M_1M_2$-Mal], H^+	$[C_{94}H_{100}N_8O_{23}]^+$	1708.69 (1709.62)	1710.06
	**3rd order fragmentation, Route D**			
**3d1**	3d1	$[C_{99}H_{109}N_7NaO_{23}]^+$	1786.75 (1787.75)	1788.39
**3d2**	3d2	$[C_{98}H_{107}N_7NaO_{23}]^+$	1772.73 (1773.73)	1771.73
	**4th order fragmentation, Route A**			
	[Mal-M_2-X-M_2-Mal], $4H_2O$, H^+	$[C_{68}H_{81}N_6O_{22}]^+$	1333.54	1333.80
	[Mal-M_2-X-M_2-Mal], $3H_2O$, H^+	$[C_{68}H_{79}N_6O_{21}]^+$	1315.53	1316.72
	[Mal-M_2-X-M_2-Mal], $2H_2O$, H^+	$[C_{68}H_{77}N_6O_{20}]^+$	1297.52	1296.73
**4a**	[Mal-M_2-X-M_2-Mal], H_3O^+	$[C_{68}H_{75}N_6O_{19}]^+$	1279.51	1280.85
	[Mal-M_2-X-M_2-Mal], H^+	$[C_{68}H_{73}N_6O_{18}]^+$	1261.50	1262.89
	**4th order fragmentation, Route B**			
	[X-M_2M_1-Mal], $2H_2O$, H^+	$[C_{67}H_{77}N_6O_{18}]^+$	1253.53	1252.88
**4b**	[X-M_2M_1-Mal], H_3O^+	$[C_{67}H_{75}N_6O_{17}]^+$	1235.52	1236.95
	[X-M_2M_1-Mal], H^+	$[C_{67}H_{73}N_6O_{16}]^+$	1217.51	1219.12
	**4th order fragmentation, Route C**			
	4c + H_2O	$[C_{75}H_{85}N_6O_{20}]^+$	1389.58	1386.69
**4c**	4c	$[C_{75}H_{83}N_6O_{19}]^+$	1371.57	1370.59
	4c - H2O	$[C_{75}H_{81}N_6O_{18}]^+$	1353.56	1354.46
	**5th order fragmentation**			
	[X-M_2-Mal], $4H_2O$, H^+	$[C_{41}H_{53}N_4O_{15}]^+$	841.35	841.64
	[X-M_2-Mal], $3H_2O$, H^+	$[C_{41}H_{51}N_4O_{14}]^+$	823.34	827.67
	[X-M_2-Mal], $2H_2O$, H^+	$[C_{41}H_{49}N_4O_{13}]^+$	805.33	805.85
**5a**	[X-M_2-Mal], H_3O^+	$[C_{41}H_{47}N_4O_{12}]^+$	787.32	789.90
	[X-M_2-Mal], H^+	$[C_{41}H_{45}N_4O_{11}]^+$	769.31	772.95
	5b + H_2O	$[C_{53}H_{61}N_4O_{14}]^+$	977.42	977.38
**5b**	5b	$[C_{53}H_{59}N_4O_{13}]^+$	959.41	961.49
	5b - H_2O	$[C_{53}H_{57}N_4O_{12}]^+$	941.40	943.39
	**6th order fragmentation**			
**6d**	Monomer [M_2-Mal], Na+	$[C_{27}H_{30}N_2NaO_7]^+$	517.19	514.46
**6c**	Monomer [M_1-Mal], Na^+	$[C_{26}H_{30}N_2NaO_5]^+$	473.20	470.66
**6b**	Fragment M_1, Na^+	$[C_{15}H_{21}N_2NaO_5]^+$	332.13	337.11
**6a**	Core [X], Na^+	$[C_{14}H_{16}N_2O_4Na]^+$	299.10	299.24

(*) *m/z* (**3a**) = *m/z* (**3c**)

Supplementary Table 32. Summary of the identified ions from the MALDI–ToF–ToF spectrum for compound **10c** (noted ($M_2M_1M_2$)-X-($M_2M_1M_2$)). Ions terminated with maleimide end-groups are denoted "Mal" and with furan protected maleimide end-group "Fm". The theoretical *m/z* value is provided as exact mass as first entry, and the nominal mass in brackets (when not otherwise indicated, exact and nominal value are identical). The identified structures are depicted in Supplementary Fig. 159.

Supplementary Methods

Chemicals

1,6-hexanediamine (synthetical grade, Merck), chloroform ($CHCl_3$, AnalaR Normapur, VWR), magnesium sulfate ($MgSO_4$, anhydrous, 99.5 % synthetical grade, Alfa Aesar), ethyl acetate (EA, AnalaR Normapur, VWR), 1,4-dioxane (analytical grade, VWR), triethylamine (Et_3N, 99 % for synthesis, Sigma Aldrich), furan (99 %, ABCR), acetic anhydride (Ac_2O, >99 %, Merck), sodium acetate (NaAc, 99 %, Alfa Aesar), *n*-hexane (Hex, AnalaR Normapur, for analysis, VWR), trifluoroacetic acid (TFA, 99 %, ABCR), dichloromethane (DCM, AnalaR Normapur, VWR), *N,N*-dimethylformamide (DMF, extra pure, Acros Organics), diethylether (Et_2O, analytical grade, VWR), methanol (MeOH, AnalaR Normapur, VWR), toluene (Normapur, VWR), petroleum ether (AnalaR Normapur, VWR), 1-ethyl-3-(3-dimethylaminopropyl)-carbodiimid hydrochloride (EDC·HCl, 98 %, Alfa Aesar), 1-hydroxybenzotriazole (HOBt, 97 %, Sigma-Aldrich), 1-hydroxy-7-azabenzotriazole (HOAT, 99 %, Sigma-Aldrich), tetrahydrofuran (THF, analytical grade, VWR), 32 % HCl (for synthesis, Acros Organics), lysine-Fmoc-NBoc (98 %, Sigma-Aldrich), piperidine (98 % for synthesis, Merck), 3,4,6,7,8,9-hexahydro-2*H*-pyrimido[1,2-a]pyrimidine (99 %, Sigma Aldrich), trimethyl orthoformate (TMOF, 99 %, Sigma-Aldrich), toluene sulfonic acid (TosOH, 97 %, Merck), 6-amino-1-hexanol (98 %, Sigma-Aldrich), 1-adamantanemethylamine (98 %, Acros Organics), 4-fluorobenzylamine (98 %, Alfa Aesar), *N,N*-diisopropylethylamine (DIPEA, 99.5 % biotech. grade, Sigma-Aldrich), 2,3-dimethylanisole (97 %, Alfa Aesar), di-tert-butyl dicarbonate (Boc_2O, 98 %, Novabiochem), 2,2-(Ethylenedioxy)bis(ethylamine) (98 %, Sigma Aldrich), copper sulfate pentahydrate ($CuSO_4 \cdot 5H_2O$, 99 %, Acros), potassium peroxodisulfate ($K_2S_2O_8$, 97 %, Sigma Aldrich), aluminium chloride ($AlCl_3$, anhydrous, 99 %, Roth), methyl-4-(bromomethyl)benzoate (97 %, ABCR), 18-crown-6 (95 %, VWR), maleic anhydride (99 %, Merck), acetonitrile (ACN, 99 %, Acros Organics), sodium chloride (NaCl, 99.5 %, Fluka) were employed without further purification. Dichloromethane (DCM, 99.8 %, extra dry, AcroSeal, Acros Organics), 1,4-dioxane (99.5 %, extra dry, AcroSeal, Acros Organics), methanol (MeOH, 99.9 %, extra dry, AcroSeal, Acros Organics), *N,N*-dimethylformamide (DMF, 99.8 %, extra dry, AcroSeal, Acros Organics), acetonitrile (ACN, 99.9 %, extra dry, AcroSeal, Acros Organics) and ethanol (EtOH, 99.5 %, extra dry, AcroSeal, Acros Organics) were purchased and employed as dry solvents.

4-((2-Formyl-3-methylphenoxy)methyl)-benzoic acid (1), 3a,4,7,7a-tetrahydro-4,7-epoxyisobenzo-furan-1,3-dione (2), and 1,6-hexanebismaleimide (3) were synthesized according to the literature. The synthesis method of the Boc-protected compound **1a** was applied as reported and adapted to **3a**, (4) as well as the transformation to **1b** and **3b** into furan-protected maleimide functional molecules.(5) This method was adapted furthermore for the synthesis of **2b**, **4b**, **5b** and **6b**. Boc-deprotection reaction was conducted via protocol reported from Burkart *et al.* and applied for all monomer intermediates (**1c**-**6c**).(6) The lysine-Fmoc-NBoc esterification and Fmoc-deprotection was reported (7) for the compounds **2a** and **2b**, and adapted to the intermediates **4a, 4b**, **5a, 4b**, **6a** and **6b**.

The mass-spectrometric data of the current study are depicted in several spectra and tables, firstly to evidence the monodisperse character of the synthesized macromolecules, secondly because different fragments and counter ions were identified. For MALDI–ToF–ToF experiments, a different numbering is introduced for clarity at the end of the Supporting Information Section, and should not be confused with the numbering followed within the rest of the document. In the MALDI–ToF data collation tables, the theoretical and experimental exact mass of the identified molecules are compared. However, for higher *m/z*-values, only the nominal mass (provided in brackets) is experimentally accessible and is comparable to the calculated theoretical nominal mass (also in brackets).

Sequence-Defined Synthesis

Principle of Photo-caged Diene (Photoenol) Ligation

The benzaldehyde (photo-caged diene) entity absorbs UV-light, being excited to a higher energy level (excited triplet state, unpaired electrons with parallel spins) after intersystem crossing (ISC). A biradical is formed and is subsequently stabilized in the form of an ortho-quinodimethane. The reaction of this diene with an ene leads to the generation of an aromatic ring which is the driving force of the cycloaddition.

Reactions Performed in the Flow Reactor

For the sequential synthesis of molecules for the first and second sequence order, the photoreaction was performed in a flow reactor designed by the authors (refer to Supplementary Fig. 38). The reactor is made of a quartz spiral (length 2 m, inner diameter 7 mm) and the reaction mixture was pumped by a peristaltic pump 5201 Heidolph. The solution was pumped at 14 rpm via a 0.7 mm Viton tube corresponding to a flow of 5 mL min^{-1}, resulting in a residence time of approx. 45 minutes. The irradiation was performed by a PL-L lamp (36 W) with a total emission in UV-A range of 91.8 W m^{-2} (1.4 W m^{-2} in UV-B range, 1.8 W m^{-2} in UV-C range) at a distance of ca. 5 cm from the spiral. The emission spectrum can be found in Supplementary Fig. 39. Purging with inert atmosphere and filling of the reactor with dry DCM was a prerequisite prior to irradiating the actual reaction mixture. The reaction mixture was preliminary prepared with dried compounds, stored under inert atmosphere and dissolved in dry DCM at a concentration of 1,6-hexanebismaleimide of 5 mmol L^{-1} (first sequence) and of 2.5 mmol L^{-1} of the previously synthesized dimer (second sequence), and of 1.25 mmol L^{-1} of previously synthesized tetramer (third sequence).

Reactions Performed in Batch

For smaller amounts, the reaction mixture was irradiated in Pyrex vials filled with 5.0 mL solution after purging with inert gas and exposed to irradiation over 45 minutes with the same PL-L lamp (36 W, emission spectrum available in Supplementary Fig. 39) at a distance of ca. 5.0 cm. The concentration was similar as for the photoreactions performed under flow conditions, i.e. 5.0 mmol L^{-1} for the first sequence order, 2.5 mmol L^{-1} for the second, 1.25 mmol L^{-1} for the third and 0.325 mmol L^{-1} for the fifth. The reaction was performed while the vials were placed on the rotating support without stirring.

NMR Characterization of the Copolymers

Due to the complexity of the synthesized molecules at high sequence orders and to enhance the accessibility of the NMR spectra for the reader, the proton chemical shift assignments of the NMR spectra are clearly identified for dimer in an exemplary analysis. NMR spectra of the homopolymer series **7a-7** from monomer **1** constitute reference spectra for the polymer chain backbone. For other copolymers, only characteristic peaks from introduced side-functions or chain termini are presented. Due to the symmetrical geometry of the 1,6-bismaleimide based polymers, only one arm of the molecule is represented, the other arm being strictly identical.

MALDI–ToF–ToF Mass-Spectrometry

Each hexamer **7c**, **8a**, **9b** and **10c** was analyzed via MALDI–ToF–ToF mass-spectrometry in order to decode the sequence order. Due to the complexity of the obtained spectra, a different numbering from the rest of the Support Information Section of the identified ionic species is introduced. Considering the first identified peak (noted $\mathbf{M^{Na}}$ in the spectrum) with the highest *m/z*-value, one can identify the molecule of interest after the loss of two furan moieties – the end-groups is denoted in that case “-Mal” – and different counter ions (K^+, Na^+ and H^+). The molecule undergoes different fragmentation orders

corresponding to the loss of one monomer unit classified in the corresponding table. In some cases, the defragmentation route can be divided in different pathways and generate specific ions which either did not undergo the loss of a furan end-group (noted in that case “-Fm”) or result from a partial fragmentation of the monomer unit. For clarity, a mechanism for the different fragmentation pathways is given with the spectrum, as well as the identified structures for ions generated from the third to the sixth fragmentation. For clarity, only species ionized with a H_3O^+ counter ion are given a specific name and are represented in the spectra and the fragmentation pathway. Thus, identical molecules ionized with water molecules are only indicated in the analytic tables to fulfill the complete characterization of the MALDI–ToF–ToF spectra.

Synthesis of Synthon 1 (Monomer M_1)

Synthesis of *tert*-Butyl (6-aminohexyl)carbamate 1a

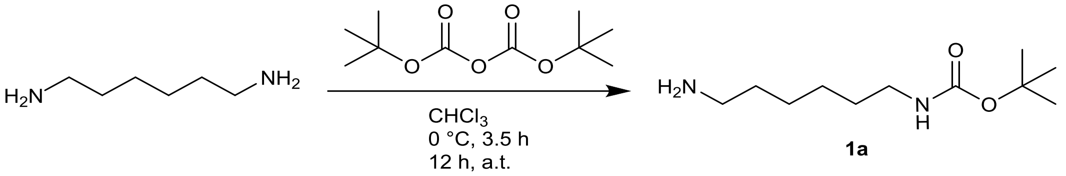

To a solution of 1,6-hexanediamine (11.5 g, 395.1 mmol, 7.50 eq.) dissolved in $CHCl_3$ (400 mL) and cooled to 0 °C, Boc_2O (45.9 g, 52.7 mmol, 1.00 eq.) dissolved in $CHCl_3$ (200 mL) was added dropwise over the course of 3 h. Afterwards, the reaction mixture was stirred for 30 minutes at 0 °C and stirred overnight at ambient temperature. After removal of the solvent under reduced pressure, the residue was dissolved in 1.5 N Na_2CO_3 (700 mL) and extracted twice with DCM (60 mL). The combined organic layers were washed three times with brine (100 mL) and dried over $MgSO_4$. After removal of the solvent under reduced pressure, the crude product was purified by recrystallization of residual 1,6-hexanediamine from toluene (-20 °C). The target compound was obtained as a yellow oil from the filtrate after evaporation of the solvent. (Yield: 33 %, 7.6 g). ^1H NMR (500 MHz, $CDCl_3$): δ 4.56 (s, 1H), 3.09 (dd, J = 12.4, 6.1 Hz, 2H), 2.66 (td, J = 7.0, 4.0 Hz, 2H), 1.48 – 1.36 (m, 12H), 1.35 – 1.25 (m, J = 3.6 Hz, 4H), 1.20 (s, 2H). ^{13}C NMR (125 MHz, $CDCl_3$): δ 156.1, 79.1, 42.3, 40.6, 33.9, 30.2, 28.5, 26.9, 26.7.

Synthesis of *tert*-Butyl (6-(1,3-dioxo-1,3,3*a*,4,7,7*a*-hexahydro-2*H*-4,7-epoxyisoindol-2-yl)hexyl)-carbamate 1b

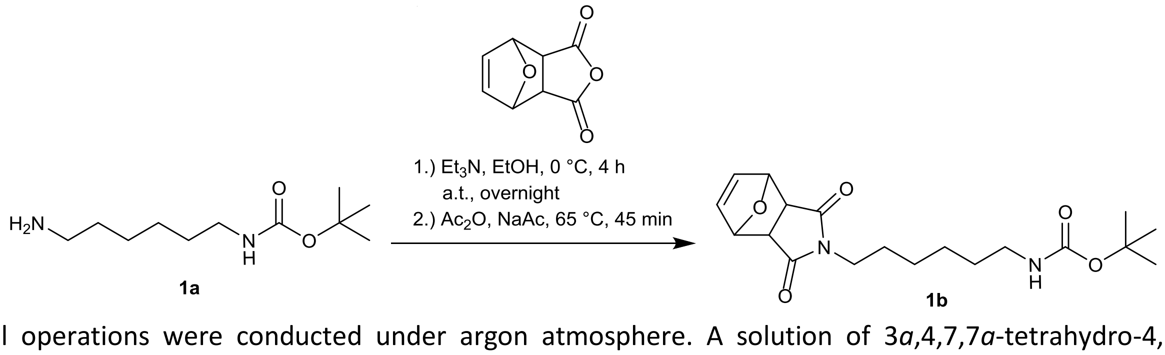

All operations were conducted under argon atmosphere. A solution of 3*a*,4,7,7*a*-tetrahydro-4,7-epoxyisobenzofuran-1,3-dione (3.66 g, 16.90 mmol, 1.20 eq.) and Et_3N (3.51 mL, 25.36 mmol, 1.80 eq.) in dry EtOH (150 mL) was slowly added to a solution of **1a** (2.34 g, 14.09 mmol, 1.00 eq.) dissolved in dry 1,4-dioxane (40 mL) at 0 °C. Subsequently, the reaction mixture was stirred for 4 h at 0 °C and overnight at ambient temperature. The solvent was removed *in vacuo* and the residue was diluted with acetic anhydride (50 mL). NaAc (1.42 g, 17.3 mmol, 1.23 eq.) was added and the mixture was heated for 45 minutes at 65 °C. Afterwards, the reaction mixture was dried *in vacuo* to eliminate the excess of Ac_2O. The residue was then cooled to 0 °C and neutralized with saturated aqueous solution of $NaHCO_3$. The resulting solution was extracted 5 times with EA (80 mL) and the collected organic

phases were washed twice with saturated $NaHCO_3$ (80 mL), to be finally dried over Na_2SO_4. After filtration and removal of the solvent, the product was purified by column chromatography (EA:Hex:MeOH 47.5:47.5:5 after solid deposition from DCM, R_f = 0.45) to provide a yellow solid. (Yield: 53 %, 4.11 g). ^{1}H NMR (500 MHz, $CDCl_3$): δ 6.44 (s, 2H), 5.19 (s, 2H), 4.49 (s, 1H), 3.38 (t, J = 7.3 Hz, 2H), 3.01 (s, 2H), 2.77 (s, 2H), 1.53 – 1.44 (m, 4H), 1.37 (dd, J = 16.9, 3.5 Hz, 9H), 1.29 – 1.15 (m, 4H). ^{13}C NMR (125 MHz, $CDCl_3$): δ 176.5, 156.2, 136.6, 80.9, 78.9, 47.4, 40.4, 38.8, 29.8, 28.4, 27.5, 27.4, 26.2.

Synthesis of 6-(1,3-Dioxo-1,3,3*a*,4,7,7*a*-hexahydro-2*H*-4,7-epoxyisoindol-2-yl)hexan-1-aminium 2,2,2-trifluoroacetate 1c

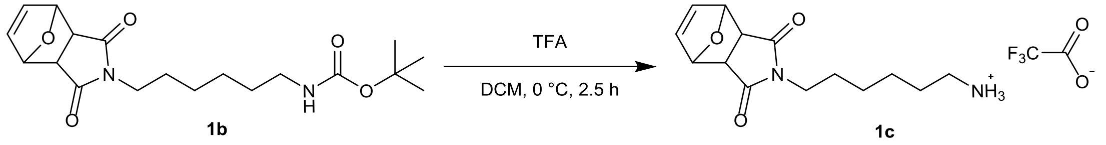

All operations were conducted under argon atmosphere. **1b** (1.82 g, 5 mmol, 1.00 eq.) was dissolved in dry DCM (225 mL) and cooled to 0 °C. TFA (11.55 mL, 149.92 mmol, 32 eq.) was slowly added and the reaction mixture was stirred for 2.5 h until thin layer chromatography (TLC) showed the absence of starting material. The solvent and TFA were subsequently removed *in vacuo* at 20 °C by threefold dissolution of the residue in DCM and subsequent evaporation. The resulting residue was weighed in order to quantify the excess amount of residual TFA to determine the excess amount of Et_3N necessary for the next synthetic step. (Yield: 100 %, 2.05 g). ^{1}H NMR (500 MHz, $CDCl_3$): δ 7.83 (s, 3H), 6.44 (s, 2H), 5.17 (s, 2H), 3.39 (t, J = 7.0 Hz, 2H), 2.84 – 2.78 (m, 4H), 1.62 – 1.52 (m, 4H), 1.34 – 1.11 (m, 4H). ^{13}C NMR (125 MHz, $CDCl_3$): δ 176.6, 136.5, 80.9, 47.4, 39.6, 38.4, 27.1, 27.0, 25.5, 25.3.

Synthesis of *N*-(6-(1,3-Dioxo-1,3,3*a*,4,7,7a-hexahydro-2*H*-4,7-epoxyisoindol-2-yl)hexyl)-4-((2-formyl-3-methylphenoxy)methyl)benzamide 1

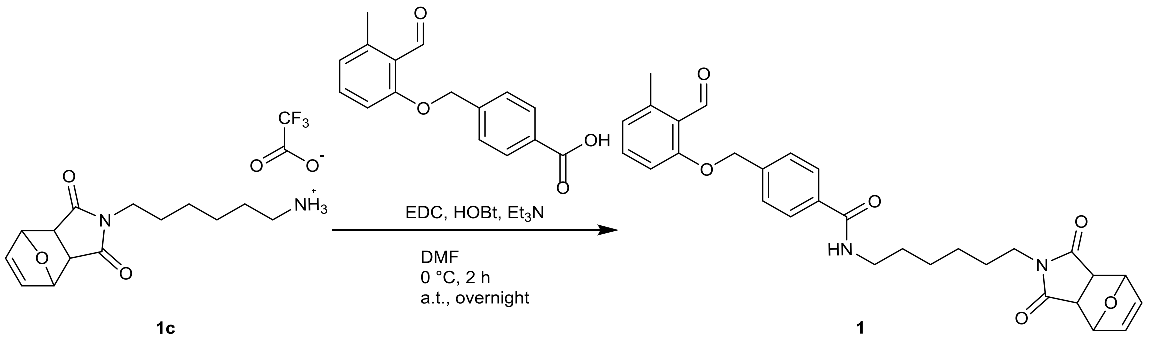

All operations were conducted under argon atmosphere. **1c** (1.90 g, 5.01 mmol, 1.00 eq.), 4-((2-formyl-3-methylphenoxy)methyl)-benzoic acid (1.49 g, 5.51 mmol, 1.10 eq.) and HOBt (1.02 g, 7.62 mmol, 1.50 eq.) were dissolved in DMF (60 mL) and cooled to 0 °C. Et_3N (1.60 mL, 11.53 mmol, 2.30 eq.) was added dropwise to the mixture. Subsequently EDC·HCl (1.11 g, 3.75 mmol, 1.15 eq.) was added and the reaction mixture was stirred for 2 h at 0 °C and overnight at ambient temperature. The mixture was diluted with EA (250 mL), washed twice with saturated $NaHCO_3$ (25 mL) and brine (50 mL). Afterwards, the organic layer was dried over $MgSO_4$ and the solvent was evaporated *in vacuo*. Purification was carried out by column chromatography (EA, R_f = 0.45, DCM:MeOH 98:2, R_f = 0.3) to provide the product as a white solid. (Yield: 65.2 %, 869.9 mg). The product is light-sensitive and was stored protected from light. ^{1}H NMR (500 MHz, $CDCl_3$): δ 10.66 (s, 1H), 7.74 (d, J = 8.2 Hz, 2H), 7.40 (d, J = 8.2 Hz, 2H), 7.29 (t, J = 8.0 Hz, 1H), 6.82 – 6.73 (m, 2H), 6.48 – 6.38 (m, 3H), 5.17 (s, 2H), 5.13 (s, 2H), 3.41 (t, J = 7.1 Hz, 2H), 3.35 (dd, J = 12.9, 6.8 Hz, 2H), 2.76 (s, 2H), 2.51 (s, 3H), 1.52 (d, J = 7.5 Hz, 4H), 1.47 – 1.37 (m, 2H), 1.37 – 1.25 (m, 2H). ^{13}C NMR (125 MHz, $CDCl_3$): δ 192.1, 176.4, 167.0, 162.0, 142.3, 139.6, 136.5, 134.7, 134.5, 127.4, 127.1, 124.6, 123.6, 110.4, 81.0, 70.0, 47.4, 39.6, 38.6, 29.3, 27.3, 26.0, 25.8, 21.5. UV-VIS: λ_{max} 319 nm. ESI-MS (*m/z*): $[M-Na]^+$ calcd. for $C_{30}H_{32}N_2O_6Na$, 539.2153; found,

539.2189. Details of the NMR chemical shift assignments and the mass spectrometric assignments can be found in Supplementary Figs. 1-5 and Supplementary Table 1.

Synthesis of Synthon 2 (Monomer M_2)

Synthesis of Methyl 2-((((9*H*-fluoren-9-yl)methoxy)carbonyl)amino)-6-((*tert*-butoxycarbonyl)-amino)hexanoate 2a

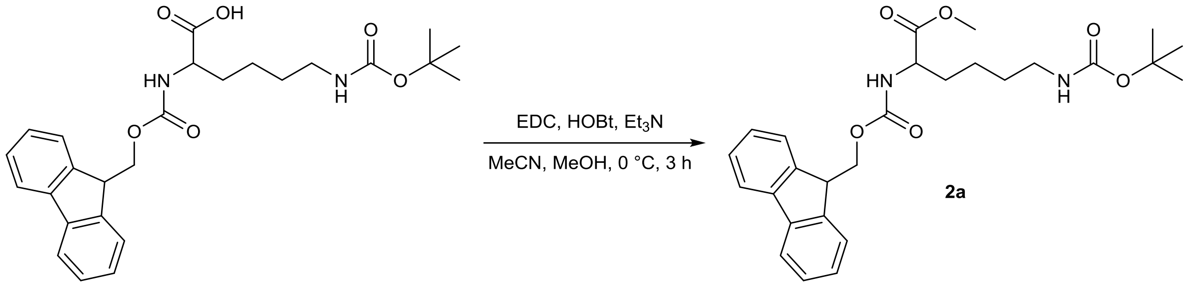

All operations were conducted under argon atmosphere. Lysine-Fmoc-NBoc (5.8 g, 12.4 mmol, 1.00 eq.) and HOBt (2.5 g, 18.5 mmol, 1.50 eq.) were dissolved in dry MeCN (60 mL) and cooled to 0 °C. Dry MeOH (50 mL) and Et_3N (2.6 mL, 20.5 mmol, 1.65 eq.) were added dropwise to the mixture. EDC·HCl (2.7 g, 14.1 mmol, 1.15 eq.) was dissolved in dry MeCN (40 mL) and dry MeOH (10 mL) and was subsequently added dropwise to the mixture. The reaction mixture was stirred for 3 h at 0 °C. Subsequently, the reaction mixture was allowed to warm to ambient temperature and was diluted with DCM (100 mL). The mixture was acidified with 0.25 N HCl (50 mL) and dispersed in saturated $NaHCO_3$ (50 mL) in order to precipitate a white solid. After filtration, the filtrate was washed once with saturated $NaHCO_3$ (50 mL) and the aqueous layer was extracted twice with DCM (50 mL). The combined organic layers were washed twice with brine (50 mL) and dried over $MgSO_4$. After filtration, the solvent was removed *in vacuo* to provide the product as a yellow oil. (Yield: 97.3 %, 5.81 g). ^{1}H NMR (500 MHz, $CDCl_3$): δ 7.69 (d, J = 7.5 Hz, 2H), 7.53 (dd, J = 7.1, 3.6 Hz, 2H), 7.33 (t, J = 7.4 Hz, 2H), 7.24 (t, J = 6.9 Hz, 2H), 5.36 (d, J = 7.6 Hz, 1H), 4.51 (s, 1H), 4.39 – 4.24 (m, 3H), 4.15 (t, J = 7.0 Hz, 1H), 3.67 (s, 3H), 3.04 (d, J = 6.0 Hz, 2H), 1.78 (ddd, J = 15.5, 10.7, 5.5 Hz, 2H), 1.49 – 1.10 (m, 13H). ^{13}C NMR (125 MHz, $CDCl_3$): δ 173.1, 156.1, 156.0, 143.9, 141.3, 128.0, 127.1, 125.1, 120.0, 79.3, 67.0, 53.7, 52.5, 47.2, 40.1, 32.1, 29.6, 28.4, 22.4.

Synthesis of Methyl 2-amino-6-((tert-butoxycarbonyl)amino)hexanoate 2b

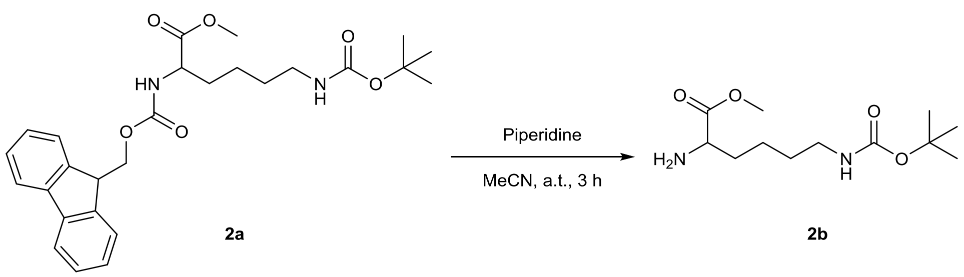

All operations were conducted under argon atmosphere. **2a** (5.81 g, 12.0 mmol, 1.00 eq.) was dissolved in dry MeCN (100 mL) under inert atmosphere. Piperidine (10 mL, 8.62 g, 101 mmol, 8.40 eq.) was added dropwise to the solution and the mixture was stirred for 3 h at ambient temperature. After removal of the solvent under reduced pressure, the crude product was purified by flash chromatography after solid deposition from DCM employing different eluents to remove side-products (Hex:EA 4:1, EA, DCM). The target compound was collected employing a gradient of DCM:MeOH from 98:2 to 95:5 (stained via $KMnO_4$). After evaporation of the solvent, the product was obtained as a yellow oil and was employed for the next reaction step without any further purification. (Yield: 84.0 %, 2.63 g). ^{1}H NMR (500 MHz, $CDCl_3$): δ 4.63 (s, 1H), 3.71 (s, 3H), 3.45 (dd, J = 7.4, 5.5 Hz, 1H), 3.17 – 3.03 (m, 2H), 1.82 – 1.45 (m, 6H). ^{13}C NMR (125 MHz, $CDCl_3$): δ 176.3, 156.0, 79.0, 54.3, 52.0, 40.3, 34.3, 29.8, 28.4, 22.8.

Synthesis of Methyl 6-((tert-butoxycarbonyl)amino)-2-(1,3-dioxo-3*a*,4,7,7*a*-tetrahydro-1*H*-4,7-epoxyisoindol-2(3*H*)-yl)hexanoate 2c

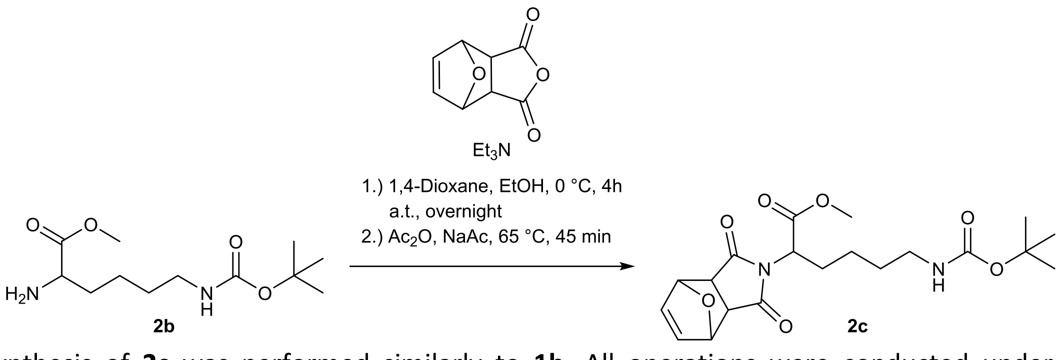

The synthesis of **2c** was performed similarly to **1b**. All operations were conducted under argon atmosphere. 3a,4,7,7a-Tetrahydro-4,7-epoxyisobenzofuran-1,3-dione (2.01 g, 12.12 mmol, 1.20 eq.) was dissolved in a mixture of dry EtOH and 1,4-dioxane (4:1, 150 mL) and compound **2b** (2.63 g, 10.10 mmol, 1.00 eq.) in 30 mL of the same solvent mixture separately. The solutions were cooled to 0 °C and **2b** was subsequently transferred dropwise to the 3a,4,7,7a-tetrahydro-4,7-epoxyisobenzofuran-1,3-dione solution. Et_3N (1.34 mL, 18.2 mmol, 1.80 eq.) was added dropwise and the reaction mixture was stirred for 4 h at 0 °C and overnight at ambient temperature. The target compound was obtained after purification via column chromatography (EA:Hex 50:50, solid deposition from DCM). Once the yellow fractions were separated, the eluent mixture was changed to EA:Hex (60:40, R_f = 0.5, stained via $KMnO_4$). (Yield: 41.0 %, 1.7 g). ^{1}H NMR (500 MHz, $CDCl_3$): δ 6.46 (s, 2H), 5.23 (d, J = 3.7 Hz, 2H), 4.64–4.50 (m, 2H), 3.65 (s, 3H), 2.98 (s, 1H), 2.84 (dd, J = 24.2, 6.5 Hz, 2H), 2.13 – 1.94 (m, 2H), 1.50 – 1.25 (m, 11H), 1.24 – 1.06 (m, 2H). ^{13}C NMR (125 MHz, $CDCl_3$): δ 175.7, 175.4, 155.9, 136.5, 81.1, 79.0, 52.7, 52.5, 47.6, 47.1, 40.0, 29.0, 28.4, 27.3, 22.8.

Synthesis of 5-(1,3-Dioxo-3*a*,4,7,7*a*-tetrahydro-1*H*-4,7-epoxyisoindol-2(3*H*)-yl)-6-methoxy-6-oxohexan-1-aminium 2,2,2-trifluoroacetate 2d

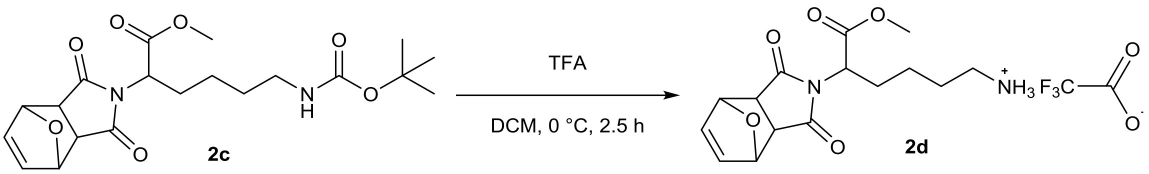

The synthesis of **2d** was performed similarly to **1c**. All operations were conducted under argon atmosphere. **2c** (1.7 g, 4.16 mmol, 1.00 eq.) was dissolved in dry DCM (150 mL) and cooled to 0 °C. TFA (10.2 mL, 133.12 mmol, 32 eq.) dissolved in dry DCM (50 mL) was added dropwise (total concentration of TFA in the reaction mixture was 5 %) to **2c** and the reaction mixture was stirred for 3 h at 0 °C. The solvent and TFA were subsequently removed *in vacuo* at ambient temperature by threefold dissolution of the residue in DCM (100 mL) and subsequent evaporation. The amount of TFA residue was determined gravimetrically. (Yield: 100 %, 1.76 g). ^{1}H NMR (500 MHz, $CDCl_3$): δ 7.16 (s, 2H), 6.46 (s, 2H), 5.20 (s, 2H), 4.56 (t, J = 7.6 Hz, 1H), 3.65 (s, 3H), 3.11 – 2.76 (m, 4H), 2.90 (s, 2H), 2.13 – 1.88 (m, 2H), 1.59 (s, 2H), 1.31 – 1.12 (m, 3H). ^{13}C NMR (125 MHz, $CDCl_3$): δ 176.3, 169.2, 136.5, 81.2, 53.0, 52.2, 47.6, 47.1, 40.1, 26.9, 26.2, 22.0.

Synthesis of Methyl 2-(1,3-dioxo-3*a*,4,7,7a-tetrahydro-1*H*-4,7-epoxyisoindol-2(3*H*)-yl)-6-(4-((2-formyl-3-methylphenoxy)methyl)benzamido)hexanoate 2

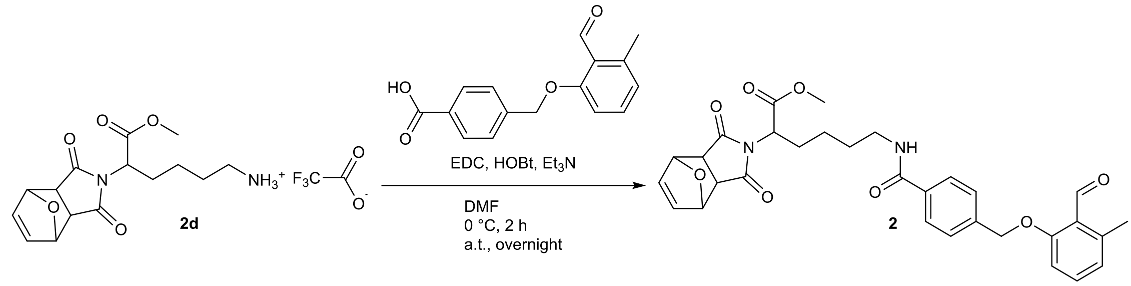

The synthesis of **2** was performed similarly to **1**. All operations were conducted under argon atmosphere. **2d** (1.76 g, 4.16 mmol, 1.00 eq.) was dissolved in dry DMF (30 mL), cooled to 0 °C and Et_3N (0.97 g, 9.56 mmol, 2.30 eq.) was added. 4-((2-formyl-3-methylphenoxy)methyl)-benzoic acid (1.24 g, 4.58 mmol, 1.10 eq.) and HOBt (846 mg, 6.24 mmol, 1.50 eq.) were dissolved in dry DMF (30 mL), cooled to 0 °C and were added to the reaction mixture afterwards. Subsequently, EDC·HCl (916 mg, 4.78 mmol, 1.15 eq.) dissolved in dry DMF (20 mL), cooled to 0 °C was added dropwise and the reaction mixture was stirred for 2 h at 0 °C and overnight at ambient temperature. The reaction mixture was diluted with DCM (250 mL), washed twice with saturated $NaHCO_3$ (50 mL) and brine (100 mL). The organic layer was dried over $MgSO_4$ and the solvent was evaporated *in vacuo*. The target compound was obtained as a yellow oil after purification by column chromatography (DCM:MeOH 98:2, R_f = 0.14). (Yield: 62 %, 1.45 g). ^1H NMR (500 MHz, $CDCl_3$): δ 9.41 (s, 1H), 7.72 (d, J = 8.3 Hz, 2H), 7.37 (d, J = 8.2 Hz, 2H), 7.29 (t, J = 8.0 Hz, 1H), 6.85 – 6.73 (m, 2H), 6.41 (s, 2H), 6.34 – 6.22 (m, 1H), 5.12 (s, 4H), 4.60 (dd, J = 10.4, 5.3 Hz, 1H), 3.65 (s, 3H), 3.35 (dd, J = 12.3, 6.4 Hz, 2H), 2.81 (dd, J = 29.6, 6.5 Hz, 2H), 2.51 (s, 3H), 2.21 – 2.00 (m, 2H), 1.71 – 1.36 (m, 2H), 1.33 – 1.09 (m, 2H). ^{13}C NMR (125 MHz, $CDCl_3$): δ 192.0, 175.7*, 175.4*, 169.0, 167.0, 161.9, 142.3, 139.5, 136.5, 134.6, 134.5, 127.5, 127.0, 124.7, 123.7, 110.4, 81.1, 70.0, 52.7, 52.5, 47.6*, 47.1*, 39.6, 28.5, 27.4, 23.0, 21.5. UV-VIS: λ_{max} 318 nm. ESI-MS (*m/z*): $[M-Na]^+$ calcd. for $C_{31}H_{32}N_2O_8Na$, 583.2051; found, 583.2075. The signal marked with a * are split (refer to comment in the method description). Details of the NMR chemical shift assignments and the mass spectrometric assignments can be found in Supplementary Figs. 6-10, and Supplementary Table 2.

Synthesis of Synthon 3 (Monomer M_3)

The synthesis of **3** was performed similar to compound **1** using 2-(2-(2-aminoethoxy)ethoxy)ethylamine instead of 1,6-hexanediamine.

Synthesis of *tert*-Butyl (2-(2-(2-aminoethoxy)ethoxy)ethyl)carbamate 3a

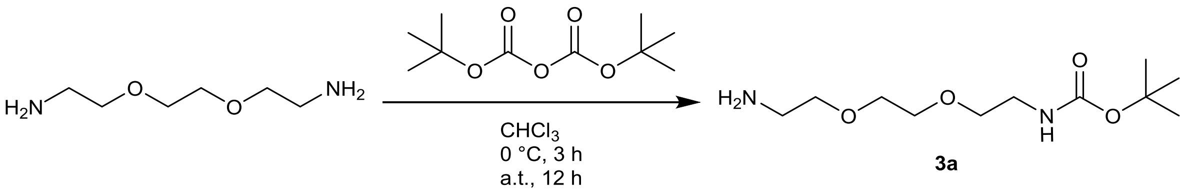

The synthesis of **3a** was performed similarly to **1a**. To a solution of 2-(2-(2-aminoethoxy)ethoxy)ethylamine (27.3 g, 184.36 mmol, 7.50 eq.) dissolved in $CHCl_3$ (400 mL) and cooled to 0 °C, Boc_2O (0.57 g, 24.6 mmol, 1.00 eq.) dissolved in $CHCl_3$ (200 mL) was added dropwise over the course of 3 h. Afterwards, the reaction mixture was stirred for 30 minutes at 0 °C and overnight at ambient temperature. After removal of the solvent under reduced pressure, the residue was dissolved in of 3 N Na_2CO_3 (400 mL) and extracted twice with DCM (60 mL). The combined organic layers were washed three times with brine (100 mL) and dried over $MgSO_4$. After removal of the solvent under reduced pressure, the target compound was obtained as a yellow oil. (Yield: 90.0 %, 5.5 g). ^1H NMR (500 MHz, $CDCl_3$): δ 5.17 (s, 1H), 3.66 – 3.56 (m, 4H), 3.52 (dd, J = 11.1, 5.9 Hz, 4H), 3.29

(s, 2H), 2.92 – 2.80 (m, 2H), 1.72 (s, 2H), 1.41 (s, 9H). ^{13}C NMR (125 MHz, $CDCl_3$): δ 156.1, 79.3, 77.2, 73.4, 70.3, 41.8, 40.4, 28.5.

Synthesis of *tert*-Butyl (2-(2-(2-(1,3-dioxo-1,3,3*a*,4,7,7*a*-hexahydro-2*H*-4,7-epoxyisoindol-2-yl)ethoxy)ethoxy)ethyl)carbamate 3b

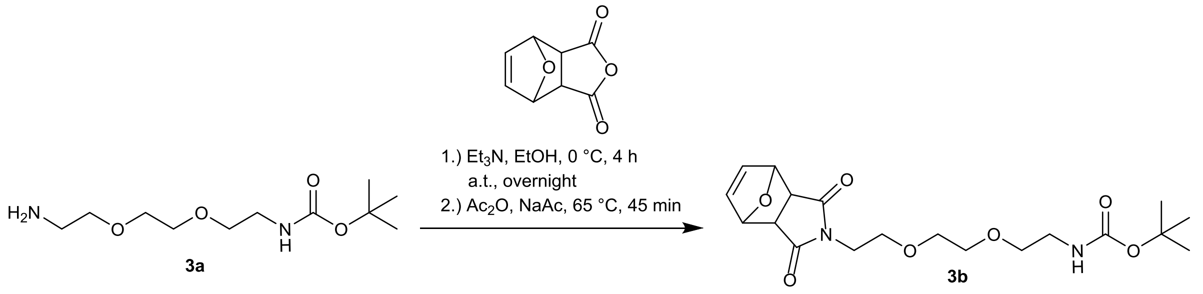

The synthesis of **3b** was performed similarly to **1b**. All operations were conducted under argon atmosphere. To a solution of **3a** (4.20 g, 16.90 mmol, 1.20 eq.) and Et_3N (3.52 mL, 25.36 mmol, 1.80 eq.) dissolved in dry EtOH (150 mL), 3*a*,4,7,7*a*-tetrahydro-4,7-epoxyisobenzofuran-1,3-dione (2.34 g, 14.09 mmol, 1.00 eq.) dissolved in dry 1,4-dioxane (40 mL) was slowly added at 0 °C. Subsequently, the mixture was stirred for 4 h at 0 °C and overnight at ambient temperature. The solvent was removed *in vacuo* and the residue was diluted with acetic anhydride (50 mL). NaAc (1.42 g, 17.32 mmol, 1.23 eq.) was added and the mixture was heated for 45 minutes to 65 °C. Afterwards, the excess of acetic anhydride was removed under reduced pressure. The residue was then cooled to 0 °C and neutralized with saturated $NaHCO_3$. The mixture was extracted 5 times with EA (80 mL) and the combined organic layers were washed twice with saturated $NaHCO_3$ (80 mL), to be finally dried over Na_2SO_4. After filtration and the removal of the solvent, the product was purified by column chromatography to provide the target compound as a yellow oil (EA:petroleum ether 10:1 after solid deposition from DCM, R_f = 0.4). (Yield: 66.7 %, 4.46 g.) ^{1}H NMR (500 MHz, $CDCl_3$): δ 6.45 (s, 2H), 5.20 (s, 2H), 5.03 (s, 1H), 3.60 (dt, J = 11.2, 5.9 Hz, 4H), 3.51 (qd, J = 6.0, 2.8 Hz, 4H), 3.44 (t, J = 5.1 Hz, 2H), 3.23 (d, J = 3.4 Hz, 2H), 2.80 (s, 2H), 1.37 (s, 9H). ^{13}C NMR (125 MHz, $CDCl_3$): δ 176.0, 156.0, 136.6, 80.9, 79.3, 70.3, 70.0, 69.8, 67.2, 47.6, 40.5, 38.2, 28.4.

Synthesis of 2-(2-(2-(1,3-Dioxo-1,3,3*a*,4,7,7*a*-hexahydro-2*H*-4,7-epoxyisoindol-2-yl)ethoxy)ethoxy)-ethan-1-aminium 2,2,2-trifluoroacetate 3c

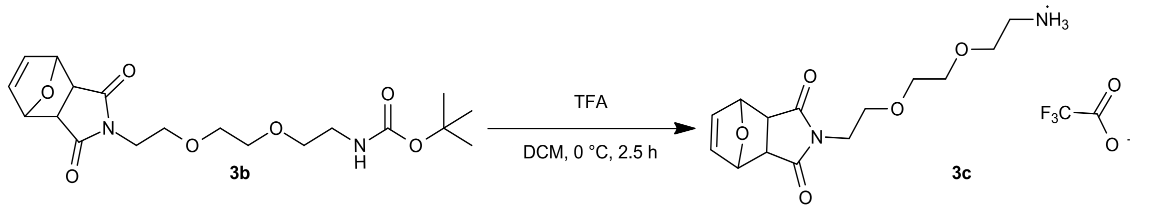

The Boc-deprotection of **3b** succeeded employing exactly the same conditions as for **1c** with **3b** (1.98 g, 5.00 mmol, 1.00 eq.), DCM (230 mL) and TFA (14.6 mL, 160 mmol, 32 eq.). The conversion and the residue of TFA was determined via TLC. The product was directly employed for the next reaction without any further characterization.

Synthesis of *N*-(2-(2-(2-(1,3-Dioxo-1,3,3a,4,7,7*a*-hexahydro-2*H*-4,7-epoxyisoindol-2-yl)ethoxy) ethoxy)ethyl)-4-((2-formyl-3-methylphenoxy)methyl)benzamid 3

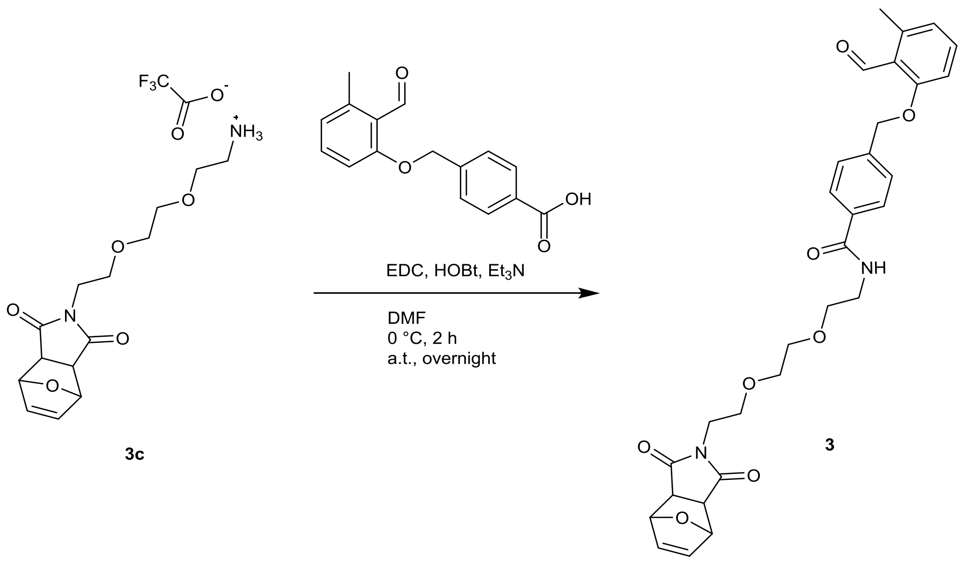

The synthesis of **3** was performed similarly to **1**. All operations were conducted under argon atmosphere. **3c** (2.06 g, 5.01 mmol, 1.00 eq.), 4-((2-formyl-3-methylphenoxy)methyl)-benzoic acid (1.49 g, 5.51 mmol, 1.10 eq.) and HOBt (1.02 g, 7.62 mmol, 1.50 eq.) were dissolved in DMF (60 mL) and cooled to 0 °C. Subsequently, Et_3N (1.6 mL, 11.53 mmol, 2.30 eq.) and EDC·HCl (1.11 g, 3.75 mmol, 1.15 eq.) were added, the reaction mixture was stirred for 2 h at 0 °C and overnight at ambient temperature. Subsequently, the reaction mixture was diluted with EA (250 mL), washed twice with 1 N HCl (20 mL), twice with saturated $NaHCO_3$ (25 mL) and brine (50 mL). The organic layer was dried over $MgSO_4$ and the solvent was evaporated *in vacuo*. Purification was carried out by column chromatography to provide the product as a white solid (EA:Hex:MeOH 47.5:47.5:5, R_f = 0.36). (Yield: 80.3 %, 2.2 g). ^{1}H NMR (500 MHz, $CDCl_3$): δ 10.68 (s, 1H), 7.82 (d, J = 8.3 Hz, 2H), 7.44 (d, J = 8.3 Hz, 2H), 7.30 (t, J = 8.0 Hz, 1H), 6.96 (s, 1H), 6.81 (d, J = 8.4 Hz, 1H), 6.77 (d, J = 7.4 Hz, 1H), 6.38 (s, 2H), 5.20 – 5.06 (m, 4H), 3.67 – 3.44 (m, 12H), 2.73 (s, 2H), 2.52 (s, 3H). ^{13}C NMR (125 MHz, $CDCl_3$): δ 192.1, 176.2, 167.0, 162.0, 142.3, 139.7, 136.5, 134.5, 134.4, 127.6, 127.1, 124.6, 123.7, 110.4, 80.9, 70.3, 70.0, 69.7, 69.7, 67.2, 47.5, 39.8, 38.2, 21.5. UV-VIS: λ_{max} 317 nm. ESI-MS (*m/z*): $[M-Na]^+$ calcd. for $C_{30}H_{32}N_2O_8Na$, 571.2050; found, 571.2089. Details of the NMR chemical shift assignments and the mass spectrometric assignments can be found in Supplementary Figs. 11-15 and Supplementary Table 3.

Synthesis of Synthon 4 (Monomer M_4)

The synthesis of **4** was conducted in a similar fashion as for **2**, except for the first amide reaction where 6-hydroxyhexylamine was employed instead of methanol.

Synthesis of (9*H*-Fluoren-9-yl)methyl *tert*-butyl (6-((6-hydroxyhexyl)amino)-6-oxohexane-1,5-diyl)dicarbamate 4a

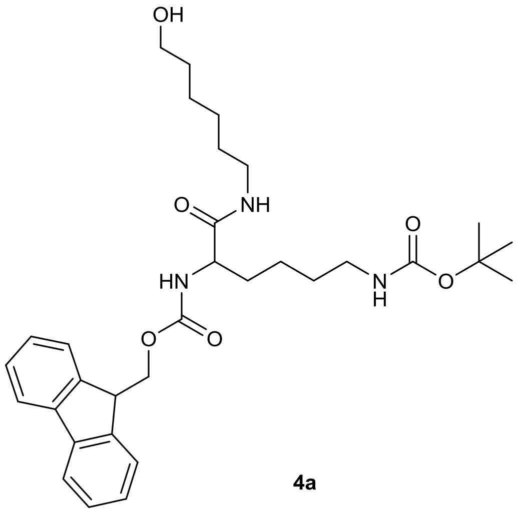

4a

The synthesis of **4a** was performed similarly to **2a**. All operations were conducted under argon atmosphere. Lysine-Fmoc-NBoc (2.30 g, 4.9 mmol, 1.05 eq.), 6-hydroxyhexylamine (547 mg, 4.67 mmol, 1.00 eq.), HOBt (840 mg, 5.60 mmol, 1.10 eq.) and *N,N*-diisopropylethylamine (0.119 mL, 0.06 g, 0.47 mmol, 0.10 eq.) were dissolved in dry DMF (75 mL) and cooled to 0 °C. EDC·HCl (980 mg, 5.13 mmol, 1.10 eq.) was added and the reaction mixture was stirred for 2 h at 0 °C and overnight at ambient temperature. Afterwards, the reaction mixture was diluted with EA (250 mL) and washed two times with 1 N HCl (50 mL), two times with saturated $NaHCO_3$ (50 mL) and brine (80 mL). The organic layer was dried over $MgSO_4$ and the solvent was removed *in vacuo*. The target compound was employed for the next reaction step without any further purification. (Yield: 99.1 %, 2.65 g). ^{1}H NMR (500 MHz, $CDCl_3$): δ 7.75 (t, J = 9.6 Hz, 2H), 7.59 (d, J = 7.2 Hz, 2H), 7.40 (t, J = 7.4 Hz, 2H), 7.31 (t, J = 7.4 Hz, 2H), 6.25 (s, 1H), 5.58 (s, 1H), 4.64 (s, 1H), 4.39 (d, J = 6.7 Hz, 2H), 4.09 – 4.03 (m, 1H), 3.59 (t, J = 6.4 Hz, 2H), 3.25 (d, J = 6.1 Hz, 2H), 3.09 (s, 2H), 1.72 – 1.57 (m, 2H), 1.56 – 1.46 (m, 8H), 1.43 (s, 9H), 1.40 – 1.27 (m, 6H).

Synthesis of *tert*-Butyl (5-amino-6-((6-hydroxyhexyl)amino)-6-oxohexyl)carbamate 4b

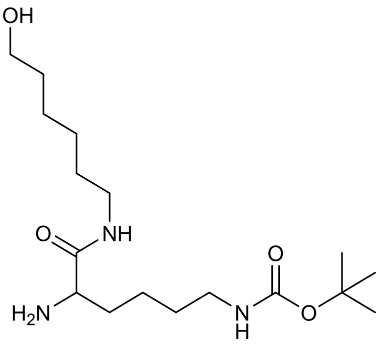

4b

The synthesis of **4b** was performed similarly to **2b**. The crude compound **4a** (2.65 g, 4.67 mmol, 1.00 eq.) was dissolved in dry MeCN (75 mL). Piperidine (7.5 mL, 6.47 g, 75.0 mmol, 8.40 eq.) was added dropwise to the solution and the reaction mixture was stirred for 3 h at ambient temperature. After removal of the solvent, purification was carried out by column chromatography (DCM:MeOH

gradient 20:1 – 10:1, solid deposition from DCM) to provide the target compound as a yellow oil (DCM:MeOH 10:1, R_f = 0.34). (Yield: 99 %, 1.60 g). ^{1}H NMR (500 MHz, $CDCl_3$): δ 7.40 (s, 1H), 4.60 (s, 1H), 3.36 (dd, J = 7.7, 4.1 Hz, 1H), 3.11 (d, J = 6.0 Hz, 2H), 2.92 (qd, J = 13.3, 6.5 Hz, 4H), 1.96 (s, 2H), 1.85 (tt, J = 10.1, 5.5 Hz, 2H), 1.74 – 1.57 (m, 4H), 1.56 – 1.45 (m, 3H), 1.42 (s, 15H). ^{13}C NMR (125 MHz, $CDCl_3$): δ 174.4, 156.3, 79.3, 62.7, 55.18, 40.3, 38.9, 34.8, 32.7, 30.1, 29.7, 28.6, 26.6, 25.4, 22.9.

Synthesis of *tert*-Butyl (5-(1,3-dioxo-1,3,3*a*,4,7,7*a*-hexahydro-2*H*-4,7-epoxyisoindol-2-yl)-6-((6-hydroxyhexyl)amino)-6-oxohexyl)carbamate 4c

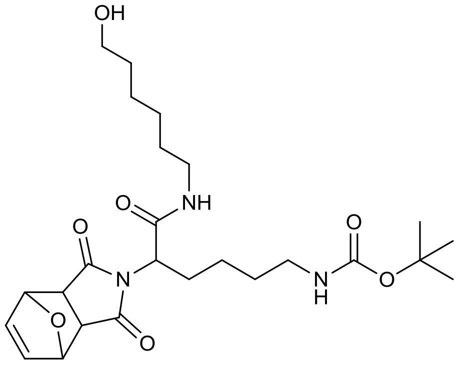

4c

The synthesis of **4c** was performed similarly to **2c**. All operations were conducted under argon atmosphere. 3*a*,4,7,7*a*-tetrahydro-4,7-epoxyisobenzofuran-1,3-dione (931 mg, 5.60 mmol, 1.20 eq.) was dissolved in a mixture of dry EtOH:1,4-dioxane (4/1, v/v) and **4b** (1.61 g, 4.67 mmol, 1.00 eq.) was dissolved in 60 mL of the same solvent mixture and cooled to 0 °C separately. Subsequently, the solution containing **4b** was transferred to the 3*a*,4,7,7*a*-tetrahydro-4,7-epoxyisobenzofuran-1,3-dione solution dropwise. Et_3N (0.62 mL, 8.41 mmol, 1.80 eq.) was added to the mixture and the reaction was stirred at 0 °C for 4 h and overnight at ambient temperature. After removal of the solvent under reduced pressure, an excess of Ac_2O (21 mL) and NaAc (471 mg, 5.74 mmol, 1.23 eq.) were added to the residue. The mixture was heated to 65 °C for 45 minutes, cooled afterwards to 0 °C and neutralized with saturated aqueous solution of $NaHCO_3$. The resulting solution was extracted 5 times with $CHCl_3$ (50 mL) and the combined organic layers were washed twice with saturated $NaHCO_3$ (30 mL), to be finally dried over $MgSO_4$. The target compound was obtained after purification via column chromatography to provide product as a yellow solid (EA:Hex 1:1, R_f = 0.54). (Yield: 56.4 %, 1.30 g). ^{1}H NMR (500 MHz, $CDCl_3$): δ 6.55 (s, 2H), 6.10 (s, 1H), 5.31 (s, 1H), 5.28 (s, 1H), 4.68 – 4.49 (m, 2H), 4.03 (dd, J = 6.6, 5.8 Hz, 2H), 3.32 – 3.19 (m, 1H), 3.19 – 3.01 (m, 3H), 2.92 (q, J = 6.4 Hz, 2H), 2.16 (dd, J = 15.3, 7.8 Hz, 2H), 1.63 – 1.55 (m, 2H), 1.53 – 1.38 (m, 14H), 1.36 – 1.19 (m, 6H). ^{13}C NMR (125 MHz, $CDCl_3$): δ 176.27, 175.86*, 167.94, 156.12, 136.57, 136.45*, 81.90, 81.78*, 79.17, 64.49, 54.99, 47.28, 47.26*, 40.16, 39.76, 29.35, 29.22, 28.59, 27.10, 26.40, 25.61, 23.47, 21.15. The signal marked with a * are split (refer to comment in the method description).

Synthesis of 5-(1,3-Dioxo-3*a*,4,7,7*a*-tetrahydro-1*H*-4,7-epoxyisoindol-2(3*H*)-yl)-6-((6-hydroxyhexyl)-amino)-6-oxohexan-1-aminium 2,2,2-trifluoroacetate 4d

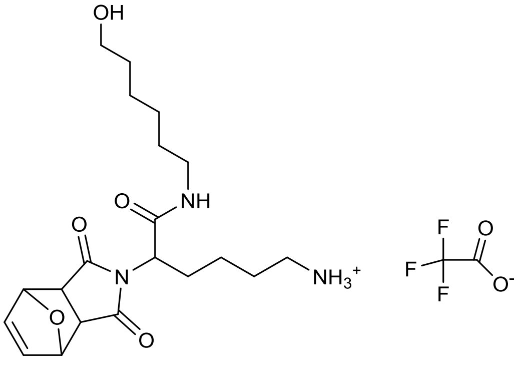

4d

The Boc-deprotection was performed under similar conditions as for **1c** with **4c** (1.84 g, 3.73 mmol), DCM (94 mL) and TFA (4.5 mL). The conversion was determined via TLC. The product was directly employed for the next reaction without any further characterization.

Synthesis of *N*-(5-(1,3-Dioxo-1,3,3*a*,4,7,7*a*-hexahydro-2*H*-4,7-epoxyisoindol-2-yl)-6-((6-hydroxyhex-yl)-amino)-6-oxohexyl)-4-((2-formyl-3-methylphenoxy)methyl)benzamide 4

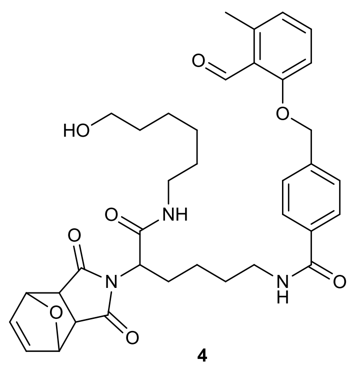

4

The synthesis of **4** was performed similarly to **1** without further purification of compound **4d**. All operations were conducted under argon atmosphere. **4d** (1.84 g, 3.73 mmol, 1.00 eq.) was dissolved in dry DMF (30 mL), cooled to 0 °C and Et_3N (1.19 mL, 868 mg, 6.13 mmol, 2.30 eq.) was added. 4-((2-formyl-3-methylphenoxy)methyl)-benzoic acid (687 mg, 4.10 mmol, 1.10 eq.) and HOAt (539 mg, 5.60 mmol, 1.50 eq.) were dissolved in dry DMF (30 mL), cooled to 0 °C and were added to the reaction mixture afterwards. Subsequently, EDC·HCl (582 mg, 4.28 mmol, 1.15 eq.) dissolved in dry DMF (15 mL), cooled to 0 °C and was added dropwise. The reaction mixture was initially stirred for 2 h at 0 °C and then overnight at ambient temperature. Subsequently, the reaction mixture was diluted with DCM (250 mL) and washed twice with saturated $NaHCO_3$ (50 mL) and brine (100 mL). The organic layer was dried over $MgSO_4$ and the solvent was evaporated *in vacuo*. The target compound was obtained as a colorless solid after purification by column chromatography (EA, R_f = 0.20). (Yield: 58.6 %, 1.31 g). ^{1}H NMR (500 MHz, $CDCl_3$): δ 10.71 (s, 1H), 7.79 (d, J = 8.1 Hz, 2H), 7.45 (d, J = 8.0 Hz, 2H), 7.35 (t, J = 8.0 Hz, 1H), 6.85 (d, *J* = 8.4 Hz, 1H), 6.83 (d, *J* = 7.6 Hz, 1H), 6.55 (s, 1H), 6.51 (s, 2H), 6.20 (s, 1H), 5.23 (s, 1H), 5.21 (s, 1H), 5.18 (s, 2H), 4.56 (dd, J = 8.8, 6.7 Hz, 1H), 4.01 (t, J = 6.6 Hz, 2H), 3.41 (q, J = 6.3 Hz, 2H), 3.23 (td, J = 12.8, 6.6 Hz, 1H), 3.10 (td, J = 12.8, 6.6 Hz, 1H), 2.90 (s, 2H), 2.56 (s, 3H), 2.23 – 2.12 (m, 2H), 2.01 (s, 1H), 1.67 – 1.52 (m, 4H), 1.46 – 1.36 (m, 2H), 1.35 – 1.21 (m, 6H). ^{13}C NMR (125 MHz, $CDCl_3$): δ 192.2, 176.3, 175.9*, 171.4, 168.0, 167.2, 162.1, 142.3, 139.7, 136.5, 136.4*, 134.6, 127.5, 127.2, 124.7, 123.7, 110.5, 81.8, 81.7*, 70.0, 64.5, 54.8, 47.3, 47.3*, 39.8, 29.2, 28.7, 28.5, 27.1, 26.4, 25.5, 23.5, 21.6, 21.1. UV-VIS: λ_{max} 317 nm. ESI-MS (*m/z*): $[M-Na]^+$ calcd. for $C_{38}H_{45}N_3O_9Na$, 710.3048; found, 710.3055. The resonances marked with a * are split (refer to comment in the method

description). Details of the NMR chemical shift assignments and the mass spectrometric assignments can be found in Supplementary Figs. 16-20 and Supplementary Table 4.

Synthesis of Synthon 5 (Monomer M_5)

The synthesis of **5** was conducted in a similar fashion as for **2** except 6-(((-adamantan-2-yl)methyl)amine was employed instead of methanol.

Synthesis of (9*H*-Fluoren-9-yl)methyl *tert*-butyl (6-(((-adamantan-2-yl)methyl)amino)-6-oxohexane-1,5-diyl)dicarbamate 5a

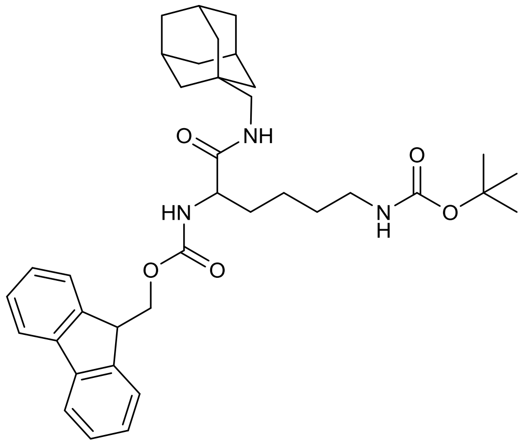

5a

The synthesis of **5a** was performed similarly to **2a**. All operations were conducted under argon atmosphere. Lysine-Fmoc-NBoc (2.30 g 4.9 mmol, 1.05 eq.), 6-(((-adamantan-2-yl)methyl)amine (725 mg, 4.67 mmol, 1.00 eq.), HOAt (840 mg, 5.60 mmol, 1.10 eq.) and *N,N*-diisopropylethylamine (0.119 mL, 0.06 g, 0.47 mmol, 0.10 eq.) were dissolved in dry DMF (75 mL) and cooled to 0 °C. EDC·HCl (980 mg, 5.13 mmol, 1.10 eq.) was added and the reaction mixture was stirred for 2 h at 0 °C and additionally overnight at ambient temperature. The reaction mixture was diluted with EA (250 mL), washed two times with 1 N HCl (50 mL), two times with saturated $NaHCO_3$ solution (50 mL) and with brine (80 mL). The organic layer was dried over $MgSO_4$ and the solvent was removed *in vacuo* to provide the target compound as a white solid. The compound was employed for the next reaction step without any further purification. (Yield: 98.3 %, 2.86 g). ^{1}H NMR (500 MHz, $CDCl_3$): δ 7.78 (dd, J = 12.5, 7.5 Hz, 2H), 7.62 (d, J = 7.2 Hz, 2H), 7.47 – 7.38 (m, 2H), 7.34 (t, J = 7.4 Hz, 2H), 6.19 (s, 1H), 5.59 (s, 1H), 4.64 (s, 1H), 4.41 (dd, J = 14.7, 8.6 Hz, 2H), 4.24 (t, J = 7.0 Hz, 1H), 4.05 – 3.98 (m, 1H), 3.23 – 3.03 (m, 2H), 2.00 –1.92 (m, 3H), 1.74 – 1.68 (m, 6H), 1.65 – 1.57 (m, 8H), 1.45 (t, J = 14.9 Hz, 15H). The product was directly employed for the next reaction without any further characterization.

Synthesis of *tert*-Butyl (6-(((-adamantan-2-yl)methyl)amino)-5-amino-6-oxohexyl)carbamate 5b

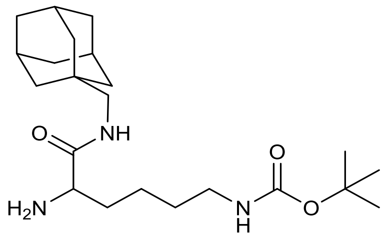

5b

The synthesis of **5b** was performed similarly to **2b**. All operations were conducted under argon atmosphere. The crude compound **5a** (2.86 g, 4.67 mmol, 1.00 eq.) was dissolved in dry MeCN (75 mL). Piperidine (7.5 mL, 6.47 g, 75.0 mmol, 8.40 eq.) was added dropwise to the solution and the reaction mixture was stirred for 3 h at ambient temperature. After removal of the solvent, purification was

carried out by column chromatography (DCM:MeOH 10:1, after solid deposition from DCM) to provide the target compound as a colorless oil (DCM:MeOH 10:1, R_f = 0.62). (Yield: 93 %, 1.71 g). ^{1}H NMR (500 MHz, $CDCl_3$): δ 7.41 (s, 1H), 4.56 (s, 1H), 3.39 (dd, J = 7.7, 4.0 Hz, 1H), 3.17 – 3.06 (m, 2H), 3.02 – 2.87 (m, 2H), 1.97 (s, 2H), 1.90 – 1.82 (m, 2H), 1.73 – 1.58 (m, 11H), 1.57 – 1.28 (m, 17H). ^{13}C NMR (125 MHz, $CDCl_3$): δ 174.4, 156.3, 79.3, 62.7, 55.2, 40.3, 38.9, 34.8, 32.7, 30.1, 28.6, 26.6, 25.4, 22.9.

Synthesis of *tert*-Butyl (6-(((adamantan-1-yl)methyl)amino)-5-(1,3-dioxo-1,3,3*a*,4,7,7*a*-hexahydro-2*H*-4,7-epoxyisoindol-2-yl)-6-oxohexyl)carbamate 5c

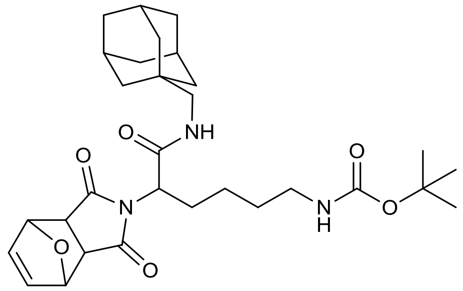

5c

The synthesis of **5c** was performed similarly to **2c**. All operations were conducted under argon atmosphere. 3*a*,4,7,7*a*-tetrahydro-4,7-epoxyisobenzofuran-1,3-dione (865 mg, 5.20 mmol, 1.20 eq.) was dissolved in a mixture of dry EtOH:1,4-dioxane (4:1) and **5b** (1.71 g, 4.34 mmol, 1.00 eq.) was dissolved in 55 mL of the same solvent mixture and cooled to 0 °C separately. Subsequently, the solution containing **5b** was transferred to the 3*a*,4,7,7*a*-tetrahydro-4,7-epoxyisobenzofuran-1,3-dione solution dropwise. Et_3N (576 µL, 7.82 mmol, 1.80 eq.) was added to the mixture and the reaction was stirred at 0 °C for 4 h and overnight at ambient temperature. After removal of the solvent under reduced pressure, an excess of Ac_2O (19.5 mL) and NaAc (437 mg, 5.33 mmol, 1.23 eq.) were added to the residue. The mixture was heated to 65 °C for 45 minutes, cooled afterwards to 0 °C and neutralized with saturated aqueous solution of $NaHCO_3$. The resulting solution was extracted 5 times with $CHCl_3$ (50 mL) and the combined organic layers were washed twice with saturated $NaHCO_3$ (30 mL), to be finally dried over $MgSO_4$. The target compound was obtained after purification via column chromatography to provide the product as a yellow solid (EA:Hex 1:1, R_f = 0.67). (Yield: 64.7 %, 1.82 g). ^{1}H NMR (500 MHz, $CDCl_3$): δ 6.63 – 6.41 (m, 2H), 6.14 (s, 1H), 5.31 (s, 1H), 5.29 (s, 1H), 4.59 (dd, J = 10.0, 5.9 Hz, 2H), 3.14 – 2.98 (m, 3H), 2.93 (s, 2H), 2.77 (dd, J = 13.4, 5.6 Hz, 1H), 2.24 – 2.10 (m, 2H), 1.94 (s, 3H), 1.73 – 1.56 (m, 6H), 1.58 – 1.35 (m, 17H), 1.32 – 1.17 (m, 2H). ^{13}C NMR (125 MHz, $CDCl_3$): δ 176.3, 175.9*, 168.2, 156.1, 136.6, 136.4*, 81.7, 81.6*, 79.2, 55.3, 51.4, 47.3, 47.3*, 40.2, 40.1, 37.0, 33.9, 29.3, 28.5, 28.3, 27.3, 23.5. The signal marked with a * are split (refer to comment in the method description).

Synthesis of 6-((Adamantan-2-ylmethyl)amino)-5-(1,3-dioxo-3a,4,7,7a-tetrahydro-1H-4,7-epoxyisoindol-2(3H)-yl)-6-oxohexan-1-aminium 2,2,2-trifluoroacetate 5d

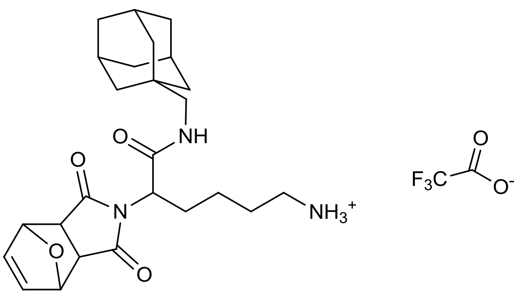

5d

The Boc-deprotection was performed in similar conditions as for **1c** with **5c** (1.82 g, 3.36 mmol, 1.00 eq.), DCM (66 mL) and TFA (9.8 mL, 107.52 mmol, 32 eq.) to provide the target compound as a

dark-green oil. The product was directly employed for the next reaction without any further characterization.

Synthesis of *N*-(6-(((Adamantan-1-yl)methyl)amino)-5-(1,3-dioxo-1,3,3*a*,4,7,7*a*-hexahydro-2*H*-4,7-epoxyisoindol-2-yl)-6-oxohexyl)-4-((2-formyl-3-methylphenoxy)methyl)benzamide 5

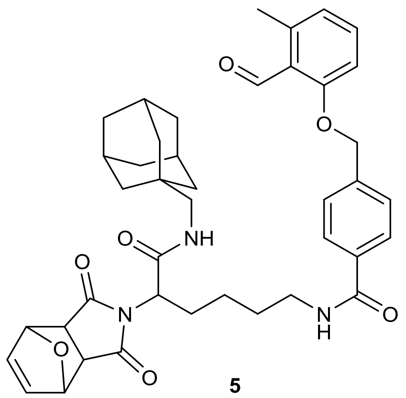

5

The synthesis of **5** was performed similarly to **1** without further purification of compound **5d**. All operations were conducted under argon atmosphere. **5d** (1.86 g, 3.36 mmol, 1.00 eq.) was dissolved in dry DMF (30 mL), cooled to 0 °C and Et_3N (1.07 mL, 781 mg, 7.73 mmol, 2.30 eq.) was added. 4-((2-formyl-3-methylphenoxy)methyl)-benzoic acid (547 mg, 3.70 mmol, 1.10 eq.) and HOAt (376 mg, 5.00 mmol, 1.50 eq.) were dissolved in dry DMF (30 mL), cooled to 0 °C and were added to the reaction mixture afterwards. Subsequently, EDC·HCl (582 mg, 4.28 mmol, 1.15 eq.) dissolved in dry DMF (15 mL), cooled to 0 °C was added dropwise and the reaction mixture was stirred for 2 h at 0 °C and overnight at ambient temperature. The reaction mixture was diluted with DCM (250 mL) and washed twice with saturated $NaHCO_3$ (50 mL) and brine (100 mL). The organic layer was dried over $MgSO_4$ and the solvent was evaporated *in vacuo*. The target compound was obtained as a colorless solid after purification by column chromatography (gradient EA:Hex 70:30 – pure EA, R_f = 0.24 in EA). (Yield: 73.9 %, 1.72 g). ^{1}H NMR (500 MHz, $CDCl_3$): δ 10.73 (s, 1H), 7.80 (d, J = 8.2 Hz, 2H), 7.45 (d, J = 8.2 Hz, 2H), 7.35 (t, J = 8.0 Hz, 1H), 6.85 (d, *J* = 8.4 Hz, 1H), 6.83 (d, *J* = 7.6 Hz, 1H), 6.51 (s, 2H), 6.47 (t, J = 5.6 Hz, 1H), 6.18 (t, J = 6.1 Hz, 1H), 5.25 (s, 2H), 5.19 (s, 2H), 4.61 (dd, J = 9.4, 6.2 Hz, 1H), 3.49 – 3.36 (m, 2H), 3.01 (dd, J = 13.4, 6.8 Hz, 1H), 2.95 – 2.87 (m, 2H), 2.77 (dd, J = 13.4, 5.7 Hz, 1H), 2.57 (s, 3H), 2.29 – 2.13 (m, 2H), 1.94 (s, 3H), 1.72 – 1.53 (m, 8H), 1.40 (d, J = 1.9 Hz, 6H), 1.37 – 1.30 (m, 2H). ^{13}C NMR (125 MHz, $CDCl_3$): δ 192.2, 176.4, 175.9*, 168.2, 167.2, 162.1, 142.4, 139.7, 136.6, 136.4*, 134.6, 134.6, 127.6, 127.2, 124.7, 123.7, 110.5, 81.7, 81.6*, 70.1, 55.2, 51.4, 47.3, 47.3*, 40.1, 39.6, 37.0, 33.9, 28.7, 28.3, 27.3, 23.6, 21.6. UV-VIS: λ_{max} 318 nm. ESI-MS (*m/z*): $[M\text{-}Na]^+$ calcd. for $C_{41}H_{47}N_3O_7Na$, 716.3306; found, 716.3345. The signal marked with a * are split (refer to comment in the method description). Details of the NMR chemical shift assignments and the mass spectrometric assignments can be found in Supplementary Figs. 21-25 and Supplementary Table 5.

Synthesis of Synthon 6 (Monomer M_6)

The synthesis of **6** was conducted under same conditions as for **2**, except for the first amide reaction carried out with 4-fluorobenzyl)amine instead of methanol.

Synthesis of (9*H*-Fluoren-9-yl)methyl *tert*-butyl (6-((4-methoxybenzyl)amino)-6-oxohexane-1,5-di-yl)dicarbamate 6a

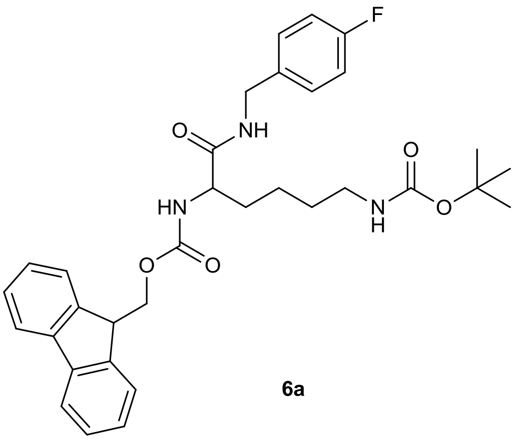

6a

The synthesis of **6a** was performed similarly to **2a**. All operations were conducted under argon atmosphere. Lysine-Fmoc-NBoc (2.30 g, 4.9 mmol, 1.05 eq.), 4-fluorobenzyl)amine (4.67 mmol, 1.00 eq.), HOBt (840 mg, 5.60 mmol, 1.10 eq.) and *N,N*-diisopropylethylamine (0.119 mL, 0.06 g, 0.47 mmol, 0.10 eq.) were dissolved in DMF (75 mL) and cooled to 0 °C. EDC·HCl (980 mg, 5.13 mmol, 1.10 eq.) was added and the reaction mixture was stirred at 0 °C for 2 h and additionally overnight at ambient temperature. The reaction mixture was diluted with EA (50 mL), washed two times with 1 N HCl (50 mL), two times with saturated $NaHCO_3$ solution (50 mL) and with brine (80 mL). The organic layer was dried over $MgSO_4$ and the solvent was removed *in vacuo* to provide the target compound as a white solid. The crude product was used for the next reaction step without any further purification. (Yield: 98.5 %, 2.72 g). ^{1}H NMR (500 MHz, $CDCl_3$): δ 7.76 (d, J = 7.7 Hz, 2H), 7.57 (t, J = 7.1 Hz, 2H), 7.39 (t, J = 7.5 Hz, 2H), 7.33 – 7.28 (m, 2H), 7.16 (d, J = 8.0 Hz, 2H), 6.80 (t, J = 9.8 Hz, 2H), 6.39 (s, 1H), 5.51 (s, 1H), 4.59 (s, 1H), 4.49 – 4.30 (m, 4H), 4.18 (t, J = 6.7 Hz, 1H), 4.13 (d, J = 5.4 Hz, 1H), 3.74 (s, 3H), 3.09 (s, 2H), 1.7-1.46(m, 4H), 1.45 – 1.37 (m, 11H). The product was directly employed for the next reaction without any further characterization.

Synthesis of *tert*-Butyl (5-amino-6-((4-fluorobenzyl)amino)-6-oxohexyl)carbamate 6b

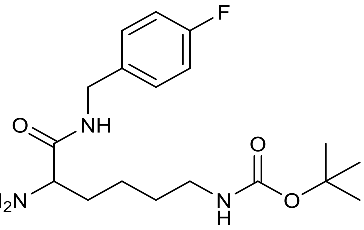

6b

The synthesis of **6b** was performed similarly to **2b**. All operations were conducted under argon atmosphere. The crude compound **6a** (2.65 g, 4.67 mmol, 1.00 eq.) was dissolved in dry MeCN (75 mL). Piperidine (7.5 mL, 6.47 g, 75.0 mmol, 8.40 eq.) was added dropwise to the solution and the reaction mixture was stirred for 3 h at ambient temperature. After removal of the solvent, purification was carried out by column chromatography (DCM:MeOH gradient 20:1 – 10:1, after solid deposition from DCM) to provide the target compound as a yellow oil (DCM:MeOH 10:1, R_f = 0.54). (Yield: 99 %, 1.64 g). ^{1}H NMR (500 MHz, $CDCl_3$): δ 7.70 (s, 1H), 7.25 – 7.20 (m, 2H), 7.03 – 6.97 (m, 2H), 4.67 – 4.50 (m, 1H), 4.39 (d, J = 6.0 Hz, 2H), 3.39 (dd, J = 8.0, 4.2 Hz, 1H), 3.11 (dd, J = 12.5, 6.2 Hz, 2H), 1.88 (tdd, J = 9.7, 6.5, 4.3 Hz, 2H), 1.64 (s, 2H), 1.59 – 1.34 (m, 13H). ^{13}C NMR (125 MHz, $CDCl_3$): δ 175.0, 162.2 (d, $^1J_{CF}$ = 245.5 Hz), 156.2, 134.5 (d, $^4J_{CF}$ = 3.2 Hz), 129.5 (d, $^3J_{CF}$ = 8.1 Hz), 115.6 (d, $^2J_{C}$ = 21.4 Hz), 79.3, 55.1, 42.5, 40.2, 34.7, 30.0, 28.5, 23.0.

Synthesis of *tert*-Butyl (5-(1,3-dioxo-1,3,3*a*,4,7,7*a*-hexahydro-2*H*-4,7-epoxyisoindol-2-yl)-6-((4-fluorobenzyl)amino)-6-oxohexyl)carbamate 6c

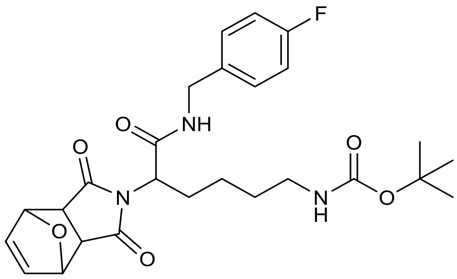

6c

The reaction conditions are similar as for the synthesis of **2c**. All operations were conducted under argon atmosphere. 3*a*,4,7,7*a*-tetrahydro-4,7-epoxyisobenzofuran-1,3-dione (925 mg, 5.57 mmol, 1.20 eq.) was dissolved in a mixture of dry EtOH:1,4-dioxane (4/1, v/v) and **6b** (1.64 g, 4.64 mmol, 1.00 eq.) was dissolved in 60 mL of the same solvent mixture and cooled to 0 °C separately. Subsequently, the solution containing **6b** was transferred to the 3*a*,4,7,7*a*-tetrahydro-4,7-epoxyisobenzofuran-1,3-dione solution dropwise. Et_3N (1.16 mL, 8.4 mmol, 1.80 eq.) was added to the mixture and the reaction was stirred at 0 °C for 4 h and overnight at ambient temperature. After removal of the solvent under reduced pressure, an excess of Ac_2O (25 mL) and NaAc (468 mg, 5.71 mmol, 1.23 eq.) were added to the residue. The mixture was heated to 65 °C for 45 minutes, cooled afterwards to 0 °C and neutralized with saturated aqueous solution of $NaHCO_3$. The resulting solution was extracted 5 times with $CHCl_3$ (50 mL) and the combined organic layers were washed twice with saturated $NaHCO_3$ (30 mL), to be finally dried over $MgSO_4$. The target compound was obtained after purification via column chromatography to provide the product as a yellow solid (EA:Hex 1:1, R_f = 0.59). (Yield: 62.3 %, 1.46 g). ^{1}H NMR (500 MHz, $CDCl_3$): δ 7.23 – 7.14 (m, 2H), 7.03 – 6.94 (m, 2H), 6.67 – 6.42 (m, 3H), 5.25 (s, 1H), 5.03 (s, 1H), 4.82 – 4.53 (m, 2H), 4.49 – 4.43 (m, 1H), 4.35 (dd, J = 14.8, 5.6 Hz, 1H), 3.19 – 2.97 (m, 2H), 2.88 (dd, J = 17.4, 6.3 Hz, 2H), 2.28 – 2.13 (m, 2H), 1.52 – 1.34 (m, 11H), 1.31 – 1.18 (m, 2H). ^{13}C NMR (125 MHz, $CDCl_3$): δ 176.2, 175.8*,168.9, 168.1*, 162.2 (d, $^1J_{CF}$ = 245.5 Hz), 156.1, 136.4, 134.4, 133.9 (d, $^4J_{CF}$ = 3.2 Hz), δ 129.5 (d, $^3J_{CF}$ = 8.1 Hz), 115.7 (d, $^2J_{CF}$ = 21.4 Hz), 115.5* (d, $^2J_{CF}$ = 21.4 Hz), 81.8, 81.6*, 79.2, 54.8, 54.7*, 47.3, 43.2, 43.1*, 40.1, 29.3, 28.5, 28.5*, 27.1, 23.6, 23.4*. The signal marked with a * are split (refer to comment in the method description).

Synthesis of 5-(1,3-Dioxo-3a,4,7,7a-tetrahydro-1H-4,7-epoxyisoindol-2(3H)-yl)-6-((4-fluorobenzyl) amino)-6-oxohexan-1-aminium 2,2,2-trifluoroacetate 6d

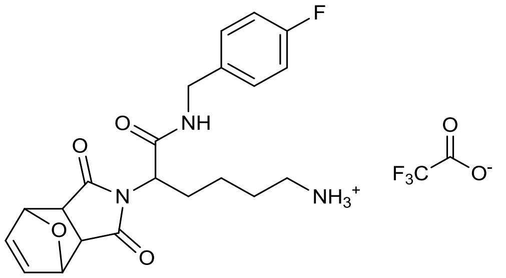

6d

The Boc-deprotection was performed under similar conditions as for **1c** with **6c** (1.46 g, 2.91 mmol, 1.00 eq.), DCM (100 mL) and TFA (8.56 mL, 93.12 mmol, 32 eq.) to provide the target compound as a dark-green oil. The product was directly employed for the next reaction without any further characterization.

Synthesis of *N*-(5-(1,3-Dioxo-1,3,3*a*,4,7,7*a*-hexahydro-2*H*-4,7-epoxyisoindol-2-yl)-6-((4-fluorobenzyl)amino)-6-oxohexyl)-4-((2-formyl-3-methylphenoxy)methyl)benzamide 6

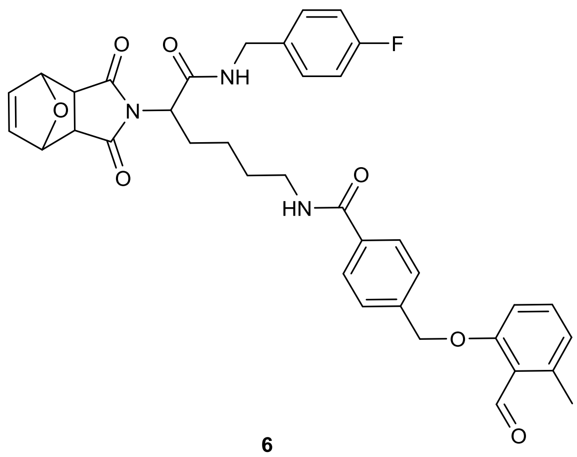

6

The synthesis of **6** was performed similarly to **1** without further purification of compound **6d**. All operations were conducted under argon atmosphere. **6d** (1.50 g, 2.91 mmol, 1.00 eq.) was dissolved in dry DMF (30 mL), cooled to 0 °C and Et_3N (934 µL, 681 mg, 6.71 mmol, 2.30 eq.) was added. 4-((2-formyl-3-methylphenoxy)methyl)-benzoic acid (856 mg, 3.20 mmol, 1.10 eq.) and HOAt (549 mg, 4.36 mmol, 1.50 eq.) were dissolved in dry DMF (30 mL), cooled to 0 °C and were added to the reaction mixture afterwards. Subsequently, EDC·HCl (642 mg, 3.34 mmol, 1.15 eq.) dissolved in dry DMF (15 mL), cooled to 0 °C was added dropwise and the reaction mixture was stirred for 2 h at 0 °C and overnight at ambient temperature. The reaction mixture was diluted with DCM (250 mL) and washed twice with saturated $NaHCO_3$ (50 mL) and brine (100 mL). The organic layer was dried over $MgSO_4$ and the solvent was evaporated *in vacuo*. The target compound was obtained as a colorless solid after purification by column chromatography (gradient EA:Hex 70:30 – pure EA, R_f = 0.36 in EA). (Yield: 69.9 %, 1.33 g). ^{1}H NMR (500 MHz, $CDCl_3$): δ 10.71 (s, 1H), 7.77 (d, J = 8.2 Hz, 2H), 7.44 (d, J = 8.1 Hz, 2H), 7.36 (t, J = 8.0 Hz, 1H), 7.17 (dd, J = 8.5, 5.4 Hz, 2H), 6.96 (t, J = 8.7 Hz, 2H), 6.85 (d, *J* = 8.4 Hz, 1H), 6.83 (d, *J* = 7.6 Hz, 1H), 6.57 (t, J = 5.7 Hz, 1H), 6.50 – 6.43 (m, 3H), 5.19 (m, 3H), 5.00 (s, 1H), 4.61 (dd, J = 9.8, 5.7 Hz, 1H), 4.48 (dd, J = 14.9, 6.4 Hz, 1H), 4.21 (dd, J = 14.8, 5.1 Hz, 1H), 3.41 (q, J = 6.5 Hz, 2H), 2.88 – 2.82 (m, 2H), 2.57 (s, 3H), 2.26 – 2.14 (m, 2H), 1.59 (dtq, J = 27.2, 13.6, 6.7 Hz, 2H), 1.40 – 1.29 (m, 2H). ^{13}C NMR (125 MHz, $CDCl_3$): δ 192.2, 176.2, 175.9*, 168.1, 167.2, 162.2 (d, $^1J_{CF}$ = 245.5 Hz), 162.1, 142.4, 139.7, 136.4, 134.6, 134.5, 133.8 (d, $^4J_{CF}$ = 3.2 Hz), 129.5 (d, $^3J_{CF}$ = 8.1 Hz), 127.5, 127.2, 124.8, 123.7, 115.5 (d, $^2J_{CF}$ = 21.4 Hz), 110.5, 81.8, 81.6*, 70.0, 54.7, 47.3, 47.3*, 43.1, 39.5, 28.8, 27.2, 23.5, 21.6. ^{19}F NMR (470 MHz, $CDCl_3$): δ -115.12, -115.13*. UV-VIS: λ_{max} 317 nm. ESI-MS (*m/z*): $[M-Na]^+$ calcd. for $C_{37}H_{36}FN_3O_7Na$, 676.2429; found, 676.2446. The signal marked with a * are split (refer to comment in the method description). Details of the NMR chemical shift assignments and the mass spectrometric assignments can be found in Supplementary Figs. 27-32 and Supplementary Table 6.

Synthesis of 1d, 2e, 3d, 5e

Synthesis of 4-((2-(Dimethoxymethyl)-3-methylphenoxy)methyl)-*N*-(6-(2,5-dioxo-2,5-dihydro-1*H*-pyrrol-1-yl)hexyl)benzamide 1d

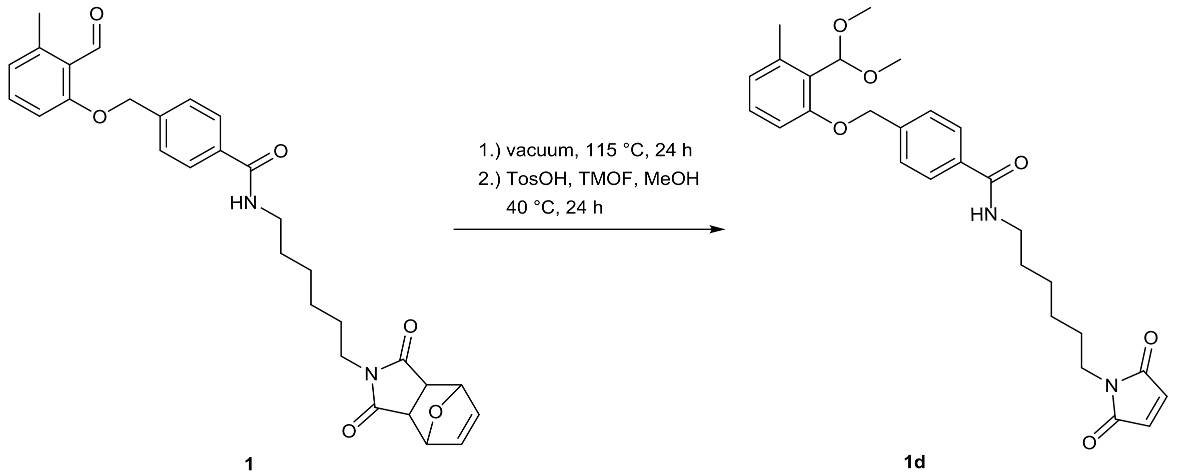

All operations were conducted under argon atmosphere and protected from light. **1** (210.6 mg, 0.41 mmol, 1.00 eq.) was heated at 115 °C for 24 h under vacuum to quantitatively remove the furan protecting group. The product was employed without any further purification. TosOH (6.2 mg, 32.6 µmol, 0.08 eq.), TMOF (180 µL, 1.63 mmol, 4.00 eq.) and anhydrous MeOH (4.5 mL) were added to the furan deprotected compound. The suspension was heated to 40 °C and stirred for 24 h. The crude reaction mixture was filtrated over silica (DCM, 1 % Et_3N) and the solvent was removed under reduced pressure to provide a pink oil. ^{1}H NMR (500 MHz, $CDCl_3$): δ 7.80 (d, J = 8.3 Hz, 2H), 7.50 – 7.46 (m, 2H), 7.14 – 7.10 (m, 1H), 6.80 (d, J = 7.6 Hz, 1H), 6.73 (d, J = 8.3 Hz, 1H), 6.68 (s, 2H), 6.24 – 6.18 (m, 1H), 5.11 (s, 1H), 3.53 (t, J = 7.1 Hz, 2H), 3.44 (q, J = 7.1, 5.8 Hz, 2H), 3.41 (s, 6H), 3.33 (s, 3H), 1.66 – 1.56 (m, 4H), 1.46 – 1.37 (m, 2H), 1.37 – 1.23 (m, 2H).

Synthesis of 4-((2-(Dimethoxymethyl)-3-methylphenoxy)methyl)-*N*-(2-(2-(2-(2,5-dioxo-2,5-dihydro-1*H*-pyrrol-1-yl)ethoxy)ethoxy)ethyl)benzamide 2e

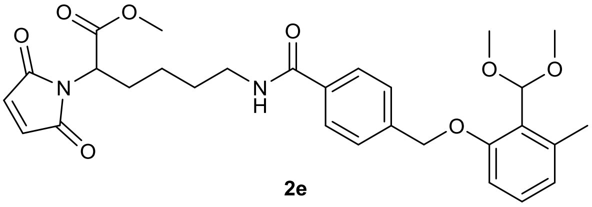

2e

The reaction was performed in a similar fashion as for **1d** with **2** (52.4 mg, 95.5 µmol, 1.00 eq.), TosOH (1.5 mg, 7.6 µmol, 0.08 eq.), TMOF (42 µL, 0.62 mmol, 4.00 eq.) and anhydrous MeOH (1.0 mL) to provide the product as an orange oil. ^{1}H NMR (500 MHz, $CDCl_3$): δ 7.87 – 7.69 (m, 3H), 7.49 (d, J = 8.1 Hz, 2H), 7.20 – 7.11 (m, 1H), 6.82 (d, J = 7.6 Hz, 1H), 6.75 (d, J = 8.3 Hz, 1H), 6.71 (s, 2H), 6.33 – 6.28 (m, 1H), 5.88 (s, 1H), 4.99 (s, 1H), 4.71 – 4.64 (m, 2H), 3.74 (d, J = 3.4 Hz, 1H), 3.43 (s, 3H), 3.34 (s, 6H), 2.52 (s, 3H), 2.23 – 2.05 (m, 2H), 1.78 – 1.56 (m, 2H), 1.40 – 1.29 (m, 2H).

Synthesis of Methyl 6-(4-((2-(dimethoxymethyl)-3-methylphenoxy)methyl)benzamido)-2-(2,5-dioxo-2,5-dihydro-1*H*-pyrrol-1-yl)hexanoate 3d

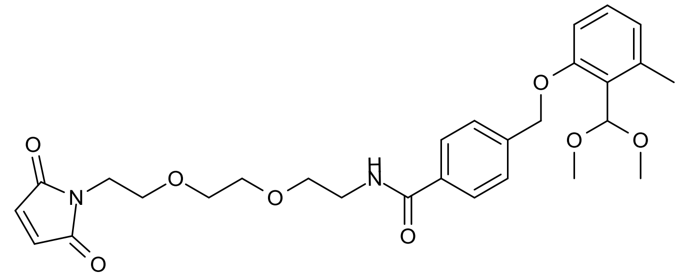

3d

The reaction was performed in a similar fashion as for **1d** with **3** (63.6 mg, 11.0 µmol, 1.00 eq.), TosOH (1.7 mg, 9.1 µmol, 0.08 eq.), TMOF (50 µL, 0.45 mmol, 4.00 eq.) and anhydrous MeOH (1.0 mL) to provide the product as a pink oil. ^{1}H NMR (500 MHz, $CDCl_3$): δ 7.84 (d, J = 8.2 Hz, 2H), 7.47 (d, J = 8.2 Hz, 2H), 7.10 (t, J = 7.9 Hz, 2H), 6.86 – 6.81 (m, 1H), 6.78 (d, J = 7.6 Hz, 1H), 6.72 (d, J = 8.1 Hz, 1H), 6.61 (s, 2H), 5.86 (s, 1H), 5.10 (s, 2H), 3.70 (t, J = 5.6 Hz, 2H), 3.67 – 3.56 (m, 10H), 3.39 (s, 6H), 2.48 (s, 3H).

Synthesis of *N*-(6-((Adamantan-1-ylmethyl)amino)-5-(2,5-dioxo-2,5-dihydro-1*H*-pyrrol-1-yl)-6-oxohexyl)-4-((2-(dimethoxymethyl)-3-methylphenoxy)methyl)benzamide 5e

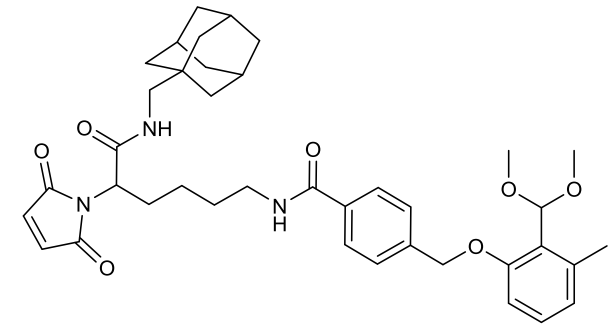

5e

The reaction was performed in a similar fashion as for **1d** with **5** (58.7 mg, 84.6 µmol, 1.00 eq.), TosOH (1.3 mg, 6.8 µmol, 0.08 eq.), TMOF (35 µL, 0.34 mmol, 4.00 eq.) and anhydrous MeOH (1.0 mL) to provide the product as a pink oil. Due to the complexity of the NMR spectrum, an indication of the proton assignment to the different chemical shift is visualized in Supplementary Fig. 36 where the expected resonances of protons 1, 20 and 22 are expected as observed for the related compounds.

Synthesis of symmetric Homopolymer 7 ($(M_1)_5$-X-$(M_1)_5$) and precursors 7a-d (7a: (M_1)-X-(M_1), 7b: $(M_1)_2$-X-$(M_1)_2$, 7c: $(M_1)_3$-X-$(M_1)_3$, 7d: $(M_1)_2$)

Synthesis of 7a (M_1)-X-(M_1)

1 (1.03 g, 2.0 mmol, 2.00 eq.) and 1,6-hexanebismaleimide (345.0 mg, 1.25 mmol, 1.25 eq.) were evacuated and purged with inert atmosphere prior to be dissolved in dry DCM (500 mL) and irradiation under flow conditions. The conversion was quantitative (as determined via NMR spectroscopy) regarding monomer **1** (characterized by the disappearance of the aldehyde signal at low field strength). Due to side reactions of the 1,6-hexansbismaleimide, the reaction mixture was precipitated in Et_2O and purified via flash chromatography by normal phase separation. The material was deposited on a dry short precolumn and separated over a SNAP Ultra column (25 g) from DCM with a MeOH gradient (25 mL min^{-1}). The solvent mixture started with DCM:MeOH 98:2 to 97:3 for 12 column volumes (CV); isocratic step 97:3 for 21 CV; gradient to 96:4 for 6 CV; isocratic step 96:4. Compound **7a** was collected between 62 and 78 CV. The purity of the different fraction was controlled via TLC (DCM:MeOH 96:4). (Yield: 68.3 %, 1.12 g). ^{1}H NMR (500 MHz, $CDCl_3$): δ 7.74 (dd, J = 8.1, 4.2 Hz, 2H), 7.43 (dd, J = 8.1, 2.4 Hz,

2H), 7.22 (td, J = 7.9, 4.1 Hz, 1H), 6.83 – 6.78 (m, 3H), 6.51 (s, 2H), 5.91 (dd, J = 4.0, 1.7 Hz, 1H), 5.19 (dd, J = 9.6, 3.3 Hz, 2H), 5.11 – 5.02 (m, 2H), 3.54 (ddd, J = 21.2, 13.9, 7.1 Hz, 2H), 3.46 (t, J = 7.1 Hz, 2H), 3.38 (dd, J = 12.3, 6.3 Hz, 2H), 3.22 – 3.04 (m, 3H), 2.91 (ddd, J = 21.5, 9.3, 4.1 Hz, 1H), 2.83 (s, 2H), 2.72 (s, 2H), 1.66 – 1.50 (m, 6H), 1.42 – 1.23 (m, 6H). ^{13}C NMR (125 MHz, $CDCl_3$): δ 180.3, 177.7, 176.5, 167.4, 154.7, 140.0, 138.5, 136.5, 134.4, 129.6, 127.3, 127.2, 126.4, 121.5, 110.9, 80.9, 70.0, 61.1, 47.4, 46.6, 39.8, 38.7, 38.6, 38.1, 29.2, 27.7, 27.3, 27.2, 26.1, 25.9, 25.7. ESI-MS (*m/z*): $[M\text{-}Na]^+$ calcd. for $C_{74}H_{80}N_6NaO_{16}$, 1331.5523; found, 1331.5626. Details of the NMR chemical shift assignments and the mass spectrometric assignments can be found in Supplementary Figs. 40-45 and in Supplementary Table 7.

Synthesis of 7b $(M_1)_2$-X-$(M_1)_2$

7a (540.5 mg, 0.413 mmol, 1.00 eq.) was thermally treated under vacuum to induce the furan deprotection of the maleimide end-groups. The reaction was carried out at 115 °C under vacuum overnight protected from light. Monomer **1** (265.7 mg, 0.867 mmol, 2.10 eq.) was added, the solids were dried and the reaction flask was purged with nitrogen prior to dissolution in dry DCM (165 mL). The reaction was performed under flow conditions and the conversion was determined via NMR spectroscopy. **7b** was precipitated in Et_2O and purified via preparative chromatography. A gradient was performed with eluent A (MQ water:THF 75:25, 20 mL min^{-1}) and eluent B (THF). From the initial isocratic condition (100 % A, 3 CV), the gradient was increased stepwise with 10 % B over 1 CV. After an isocratic step with 10 % B for 3 CV, the gradient was increased to 20 % B over 1 CV. **7b** was collected for the next gradient (20 - 30 % B). (Yield: 50 %, 455 mg). ESI-MS (*m/z*): $[M\text{-}Na]^+$ calcd. for $C_{126}H_{136}N_{10}NaO_{26}$, 2227.9519; found, 2227.9572. Details of the NMR chemical shift assignments and the mass spectrometric assignments can be found in Supplementary Figs. 46-50 and in Supplementary Table 8.

Synthesis of 7c $(M_1)_3$-X-$(M_1)_3$

7c was synthesized under similar conditions as for the sequence extension from **7a** to **7b.** After thermal treatment, **7b** (288.1 mg, 0.131 mmol, 1.00 eq.) and **1** (141.6 mg, 0.274 mmol, 2.10 eq.) were dissolved in dry DCM (105 mL) and let to react under flow conditions. **7c** was precipitated in Et_2O and isolated via preparative chromatography. A gradient was performed with eluent A (MQ water:THF 90:10, 20 mL min^{-1}) and eluent B (THF). From the initial isocratic condition (100 % A, 1 CV), the gradient was increased to 30 % B over 3 CV. **7c** was collected at peak maximum for 30 % B. (Yield: 10.7 %, 43.3 mg). ESI-MS (*m/z*): $[M\text{-}2Na]^{2+}$ calcd. for $C_{178}H_{192}N_{14}Na_2O_{36}$, 1573.6704; found, 1573.6724. All major peaks belong to the target molecule with different counter ions can be found in Supplementary Figs. 51-55 and in Supplementary Table 9.

Synthesis of 7d $(M_1)_2$

All operations were conducted under argon atmosphere. To a solution of the furan deprotected and acetal protected monomer **1d** (47.1 mg, 95.3 µmol, 1.00 eq.) dissolved in anhydrous DCM (15 mL), monomer **1** (51.7 mg, 0.1 mmol, 1.05 eq.) dissolved in anhydrous DCM (25 mL) was added (5.0 mmol L^{-1}). The monomer solution was subsequently filled into sealed and previously degassed head space vials (5 mL) and purged for 5 minutes with argon, to be finally exposed to UV light (PL-L, 355 nm) for 45 minutes (batch conditions). After irradiation, the single fractions were gathered and the solvent was removed under reduced pressure. The purification was performed via reverse phase flash chromatography (SNAP C18 12 g, 12 mL min^{-1}) with MQ water and a THF gradient of 1.0 % per CV. Isocratic phases of a constant THF content have been applied at 20, 25, 30, 35 % (5 CV respectively), and 40 % (35 CV). The product was eluted as the THF content reached 40 % in the mobile phase. An acidic workup was not performed since the aldehyde functionality was regenerated after the purification. (Yield: 58.2 %, 53.6 mg). ^{1}H NMR (500 MHz, $CDCl_3$): δ 10.71 (s, 1H), 7.77 (dd, J = 21.2, 8.1 Hz, 4H), 7.45 (t, J = 7.0 Hz, 4H), 7.38 (t, J = 8.0 Hz, 1H), 7.22 (t, J = 7.9 Hz, 1H), 6.94 – 6.77 (m, 4H), 6.68 – 6.59 (m, 2H), 6.51 (s, 2H), 5.97 (d, J = 4.0 Hz, 1H), 5.60 (dd, J = 6.0, 1.8 Hz, 1H), 5.24 (s, 2H), 5.19

(s, 2H), 5.11 (s, 2H), 3.67 – 3.56 (m, 4H), 3.45 – 3.32 (m, 4H), 3.16 (d, J = 27.1 Hz, 3H), 2.98 (dd, J = 9.1, 4.0 Hz, 1H), 2.85 (s, 2H), 2.59 (s, 3H), 2.19 – 1.60 (m, 16H). ^{13}C NMR (125 MHz, $CDCl_3$) δ 192.2, 180.4, 177.7, 176.5, 167.2, 162.0, 154.8, 142.3, 140.0, 138.48, 139.6, 136.5, 134.6, 134.5, 129.6, 127.4, 127.3, 127.2, 127.1, 127.1, 126.5, 124.6, 123.6, 121.5, 111.1, 110.4, 80.9, 70.0, 69.9, 67.7, 61.4, 50.8, 47.4, 46.7, 39.7, 39.4, 38.7, 38.4, 29.2, 29.2, 27.4, 27.3, 26.1, 25.9, 25.7, 25.7, 25.3, 21.5. ESI-MS (*m/z*): [M-Na]$^+$ calcd. for $C_{56}H_{60}N_4NaO_{11}$, 987.4151; found, 987.4163. Details of the NMR chemical shift assignments and the mass spectrometric assignments can be found in Supplementary Figs. 56-62 and Supplementary Table 10.

Synthesis of 7 $(M_1)_5$-X-$(M_1)_5$

The decamer **7** was synthesized in batch conditions from the hexamer **7c** (10.3 mg, 3.31 µmol, 1.00 eq.) and the dimer **7d** (6.7 mg, 6.97 mmol, 2.10 eq.) in dry DCM (25 mL). The purification was performed via successive precipitation from THF, Hex and MeOH mixtures. Therefore, the reaction mixture was dried under reduced pressure. The residue was diluted in THF (3 mL) and MeOH (4.5 mL) was slowly added until the solution turned turbid. The solution was let to settle and filtered (precipitation 1). After removal of the solvent under reduced pressure, the filtrate was precipitated in a second cycle, after dissolution in THF (3 mL) by addition of MeOH (2.5 mL) (precipitation 2). The filtrate underwent a subsequent precipitation cycle after removal of the solvent and dissolution in THF (3 mL) with Hex (2.5 mL) (precipitation 3). After dissolution of the solid in THF (3 mL), the expected product was found in the filtrate after addition of MeOH (4.5 mL) (precipitation 4). (Yield: 49.2 %, 8.0 mg). ESI-MS (*m/z*): [M-Na]$^+$ calcd. for $C_{126}H_{136}N_{10}NaO_{26}$, 2470.0701; found, 2470.0905. Details for mass-spectra can be found in Supplementary Figs. 63-64 and in Supplementary Table 11.

Synthesis of symmetric Copolymer 8 ($(M_1)_2(M_2)(M_1)_2$-X-$(M_1)_2(M_2)(M_1)_2$) and precursor 8a ($(M_2)(M_1)_2$-X-$(M_1)_2(M_2)$)

Synthesis of 8a $(M_2)(M_1)_2$-X-$(M_1)_2(M_2)$

8a was synthesized under similar conditions as **7c** with **7b** (247.8 mg, 0.112 mmol, 1.00 eq.) and **2** (132.2 mg, 0.236 mmol, 2.10 eq.) in dry DCM (90 mL) and let to react under flow conditions. **8a** was precipitated in Et_2O and isolated via preparative chromatography. A gradient was performed with eluent A (MQ water:THF 90:10, 20 mL min^{-1}) and eluent B (THF). From the initial isocratic condition (100 % A, 1 CV), the gradient was increased stepwise with 10 % B over 1 CV. After an isocratic step with 10 % B for 3 CV, the gradient was increased to 20 % B over 1 CV, then to 30 % over 1 CV. **7b** was collected from the next gradient (20 – 30 % B) and **8a** was collected at peak maximum for 30 % B. (Yield: 5.7 %, 20.6 mg). ESI-MS (*m/z*): [M-2Na]$^{2+}$ calcd. for $C_{180}H_{192}N_{14}Na_2O_{40}$, 1617.6602; found, 1617.6652. Details of the NMR chemical shift assignments and the mass spectrometric assignments can be found in Supplementary Figs. 65-69 and in Supplementary Table 12.

Synthesis of 8 $(M_1)_2(M_2)(M_1)_2$-X-$(M_1)_2(M_2)(M_1)_2$

The decamer **8** was synthesized in batch conditions from the hexamer **8a** (7.4 mg, 3.32 µmol, 1.00 eq.) and the dimer **7d** (4.7 mg, 4.87 mmol, 2.10 eq.) in dry DCM (25 mL). The purification was performed as for **7**. The reaction mixture was dried under reduced pressure. The solid was diluted in THF (4 mL), and MeOH (7.5 mL) was slowly added until the solution turned turbid. The solution was let to decant and filtered (precipitation 1). After removal of the solvent under reduced pressure, the filtrate underwent another precipitation cycle, after dissolution in THF (4 mL) by addition of Hex (0.5 mL) (precipitation 2). The solid underwent a subsequent precipitation cycle after removal of the solvent and dissolution in THF (4 mL) by addition of Hex (0.5 mL) (precipitation 3). After dissolution of the residue in THF (4 mL) and addition of MeOH (7.5 mL) (precipitation 4), the expected product settled as a solid and was collected via filtration. (Yield: 33.7 %, 3.9 mg). ESI-MS (*m/z*): [M-2Na]$^{2+}$ calcd. for $C_{284}H_{304}N_{22}Na_2O_{60}$, 2514.0599; found, 2514.0870. Details for mass-spectra can be found in Supplementary Figs. 70-71 and in Supplementary Table 13.

Synthesis and Characterization of symmetric Copolymer 9 ((M_1)(M_2M_1)$_2$-X-(M_1M_2)$_2$(M_1)) and precursors 9a-c (9a: (M_2M_1)-X-(M_1M_2), 9b: ($M_1M_2M_1$)-X-($M_1M_2M_1$), 9c: (M_1M_2))

Synthesis and Characterization of 9a (M_2M_1)-X-(M_1M_2)

9a was synthesized under similar conditions as **7b** with **7a** (484.21 mg, 0.370 mmol, 1.00 eq.) and **2** (435.3 mg, 0.777 mmol, 2.10 eq.) in dry DCM (148 mL). The reaction was performed under flow conditions. **9a** was precipitated in Et_2O and isolated via preparative chromatography. An elution gradient was established with eluent A (MQ water:THF 75:25, 20 mL min^{-1}) and eluent B (THF). From the initial isocratic condition (25 % A, 9 CV), the gradient was increased to 30 % B over 5 CV. **9a** was collected during the isocratic step for 25 % B after 5 CV. (Yield: 54.5 %, 462.7 mg). ESI-MS (*m/z*): [M-Na]$^+$ calcd. for $C_{128}H_{136}N_{10}NaO_{30}$, 2315.9316; found, 2315.9396. Details of the NMR chemical shift assignment and the mass spectrometric assignments can be found in Supplementary Figs. 72-76 and in Supplementary Table 14.

Synthesis of 9b ($M_1M_2M_1$)-X-($M_1M_2M_1$)

9b was synthesized under similar conditions in batch as **7c**. **9a** (168.0 mg, 73 µmol, 1.00 eq.) and **1** (79.4 mg, 0.154 mmol, 2.10 eq.) were dissolved in dry DCM (59 mL). **9b** was precipitated in Et_2O and isolated via preparative chromatography. A gradient was established with eluent A (MQ water:THF 90:10, 20 mL min^{-1}) and eluent B (THF). From the initial isocratic condition (100 % A, 3 CV), the gradient was stepwise increased with 10 % B over 1 CV. After an isocratic step with 10 % B for 3 CV, the gradient was increased to 20 % B over 1 CV and kept isocratic for 3 CV. Another increase of the gradient to 30 % B for 1 CV enabled the collection of **9b** at the peak maximum for 30 % B under isocratic conditions. (Yield: 17.2 %, 40.3 mg). ESI-MS (*m/z*): [M-2Na]$^{2+}$ calcd. for $C_{180}H_{192}N_{14}Na_2O_{40}$, 1617.6602; found, 1617.6679. Details of the NMR chemical shift assignments and the mass spectrometric assignments can be found in Supplementary Figs. 77-81 and in Supplementary Table 15.

Synthesis of 9c (M_1M_2)

All operations were conducted under argon atmosphere. The synthesis of **9c** was performed similarly to **7d** with the furan deprotected and acetal protected monomer **2e** (63.6 mg, 0.11 mmol, 1.00 eq.) and monomer **1** (61.5 mg, 0.12 mmol, 1.05 eq.) dissolved in anhydrous DCM (40 mL). After irradiation and the removal of the solvent, **9c** was diluted with DCM (30 mL) and treated with aqueous 0.5 N HCl (30 mL) for 2 h. Subsequently, the layers were separated and the aqueous layer was extracted twice with DCM (20 mL). The purification was performed via reverse phase flash chromatography (SNAP C18 12 g, 12 mL min^{-1}) with MQ water and a THF gradient of 1.0 % per column volume. Isocratic phases of a constant THF content have been applied at 20 and 25 % (9 CV respectively), 30 % (23 CV), 35 % (5 CV) and 40 % (35 CV). The product was eluted as the THF content reached 40 % in the mobile phase. The fractions were gathered, the solvent was removed under reduced pressure and the residue was precipitated in Hex at ambient temperature. (Yield: 45 %, 52 mg). ^{1}H NMR (500 MHz, $CDCl_3$): δ 10.70 (d, J = 5.6 Hz, 1H), 7.87 – 7.63 (m, 4H), 7.59 – 7.42 (m, 3H), 7.42 – 7.34 (m, 2H), 7.34 – 7.26 (m, 2H), 7.26 – 7.14 (m, 1H), 6.95 – 6.77 (m, 4H), 6.52 (s, 3H), 6.06 – 5.89 (m, 1H), 5.25 (s, 2H), 5.21 – 5.04 (m, 4H), 4.89 – 4.72 (m, 1H), 3.82 – 3.72 (m, 3H), 3.55 – 2.94 (m, 8H), 2.85 (s, 2H), 2.60 (s, 3H), 2.43 – 1.79 (m, 4H), 1.72 – 1.50 (m, 6H), 1.42 – 1.20 (m, 6H). ^{13}C NMR (125 MHz, $CDCl_3$): δ 192.1, 179.6, 176.9, 176.5, 169.7, 169.4, 167.2, 162.0, 154.7, 142.3, 138.2, 136.6, 134.5, 127.4, 127.4, 127.1, 127.0, 126.6, 124.7, 123.6, 121.6, 111.4, 110.5, 81.0, 70.2, 70.0, 61.4, 61.2, 52.8, 52.5, 47.4, 47.2, 46.3, 40.0, 39.8, 39.7, 38.7, 38.1, 37.6, 29.5, 29.2, 28.8, 27.8, 27.4, 27.4, 26.1, 25.9, 22.9, 21.5. ESI-MS (*m/z*): [M-Na]$^+$ calcd. for $C_{57}H_{60}N_4NaO_{13}$, 1031.4049; found, 1031.4116. Details of the NMR chemical shift assignments and the mass spectrometric assignments can be found in Supplementary Figs. 82-88 and in Supplementary Table 16.

Synthesis of 9 ((M_1)(M_2M_1)$_2$-X-(M_1M_2)$_2$(M_1))

The decamer **9** was synthesized in batch conditions from the hexamer **9b** (12.0 mg, 3.76 µmol, 1.00 eq.) and the dimer **9c** (7.97 mg, 7.90 mmol, 2.10 eq.) in dry DCM (25 mL). The purification was performed as for **7**. The reaction mixture was dried under reduced pressure. The solid was diluted in THF (4 mL), and MeOH (6 mL) was slowly added until the solution turned turbid. The solution was let to settle and filtered (precipitation 1). After removal of the solvent under reduced pressure, the filtrate underwent another precipitation cycle, followed by subsequent dissolution in THF (4 mL) by addition of MeOH (9 mL) (precipitation 2). The filtrate underwent a subsequent precipitation cycle after removal of the solvent and dissolution in THF (4 mL) by addition of Hex (0.3 mL) (precipitation 3). The expected product was obtained as a solid. (Yield: 47.7 %, 9.1 mg). ESI-MS (*m/z*): $[M\text{-}Na]^+$ calcd. for $C_{286}H_{304}N_{22}Na_2O_{64}$, 2558.0497; found, 2558.0698. Details of the mass spectrometric characterization can be found in Supplementary Figs. 89-90 and in Supplementary Table 17.

Synthesis and Characterization of symmetric Copolymer 10 ((M_2)(M_1M_2)$_2$-X-(M_2M_1)$_2$(M_2)) and precursors 10a-d (10a: (M_2)-X-(M_2), 10b: (M_1M_2)-X-(M_2M_1), 10c: ($M_2M_1M_2$)-X-($M_2M_1M_2$), 10d: (M_2M_1))

Synthesis and Characterization of 10a (M_2)-X-(M_2)

10a was synthesized under similar conditions as **7a** with **2** (1.06 g, 1.88 mmol, 2.00 eq.) and 1,6-hexanebismaleimde (325.0 mg, 1.18 mmol, 1.25 eq.) in dry DCM (235 mL) and let to react under flow conditions. The reaction mixture was precipitated in Et_2O and purified via flash chromatography by normal phase separation. Therefore, the material was deposited on a dry short pre-column and separated over a SNAP Ultra column (25 g) with eluent A (DCM:MeOH 96:4) and eluent B (DCM:MeOH 90:10 MeOH, 25 mL min^{-1}) under isocratic conditions (A:B 97:3). The target compound **10a** was collected between 12 and 36 CV. The purity of the different fractions was controlled via TLC (DCM:MeOH 96:4). (Yield: 83.0 %, 1.09 g). ^1H NMR (500 MHz, $CDCl_3$): δ 7.73 (t, J = 7.8 Hz, 2H), 7.41 (d, J = 7.9 Hz, 2H), 7.25 – 7.19 (m, 1H), 6.91 – 6.79 (m, 2H), 6.63 (d, J = 9.5 Hz, 1H), 6.56 – 6.48 (m, 2H), 5.96 – 5.90 (m, 1H), 5.18 (dd, J = 9.6, 3.3 Hz, 2H), 5.06 (d, J = 13.5 Hz, 2H), 4.69 – 4.61 (m, 1H), 3.73 (s, 3H), 3.63 – 3.47 (m, 2H), 3.43 – 3.33 (m, 2H), 3.22 – 3.07 (m, 3H), 2.91 (ddd, J = 26.8, 13.5, 9.1 Hz, 3H), 2.46 – 2.22 (m, 2H), 2.21 – 2.05 (m, 2H), 1.72 – 1.42 (m, 4H), 1.40 – 1.19 (m, 4H). ^{13}C NMR (125 MHz, $CDCl_3$): δ 180.3, 177.6, 175.8, 169.1, 167.3, 154.7, 139.9, 138.5, 136.5, 134.3, 129.6, 127.5, 127.2, 126.4, 121.5, 111.0, 81.1, 69.8, 61.1, 52.8, 52.4, 47.6, 46.7, 39.6, 38.5, 38.0, 28.4, 27.6, 27.3, 27.2, 25.7, 23.0. ESI-MS (*m/z*): $[M\text{-}Na]^+$ calcd. for $C_{76}H_{80}N_6NaO_{20}$, 1419.5320; found, 1419.5383. Details of the NMR chemical shift and mass spectrometric assignments can be found in Supplementary Figs. 91-96 and in Supplementary Table 18.

Synthesis of 10b (M_1M_2)-X-(M_2M_1)

10b was synthesized under similar conditions as **7c** with **10a** (417.8 mg, 0.299 mmol, 1.00 eq.) and **1** (324.3 mg, 0.628 mmol, 2.10 eq.) in dry DCM (120 mL). **10b** was precipitated in Et_2O and isolated via preparative chromatography. A gradient was established with eluent A (MQ water:THF 75:25, 20 mL min^{-1}) and eluent B (THF). From the initial isocratic condition (25 % A, 9 CV), the gradient was increased to 30 % B over 5 CV. **10a** was collected during the gradient increase from 25 – 30 % B. (Yield: 35.3 %, 242.3 mg). ESI-MS (*m/z*): $[M\text{-}Na]^+$ calcd. for $C_{128}H_{136}N_{10}NaO_{30}$, 2315.9316; found, 2315.9315. Details for of the NMR chemical shift and mass spectrometric assignments can be found in Supplementary Figs. 97-101 and in Supplementary Table 19.

Synthesis of 10c ($M_2M_1M_2$)-X-($M_2M_1M_2$)

10c was synthesized under similar conditions as **7c**. **10b** (118.4 mg, 51.6 µmol, 1.00 eq.) and **2** (60.7 mg, 0.108 mmol, 2.10 eq.) were dissolved in dry DCM (41 mL) in batch. **10c** was precipitated in Et_2O and

isolated via preparative chromatography. A gradient was established with eluent A (MQ water:THF 90:10, 20 mL min^{-1}) and eluent B (THF). From the initial isocratic condition (100 % A, 3 CV), the gradient was increased stepwise with 10 % B over 1 CV. After an isocratic step of 10 % B for 3 CV, the gradient was increased to 20 % B over 1 CV and kept isocratic for 3 CV. Another increase of the gradient to 30 % for 1 CV enabling the collection **10c** at peak maximum for 30 % B. (Yield: 38.6 %, 65.3 mg). ESI-MS (*m/z*): $[M\text{-}2Na]^{2+}$ calcd. for $C_{174}H_{184}N_{14}Na_2O_{44}$, 1661.6501; found, 1661.6630. Details of the NMR chemical shift and mass spectrometric assignments can be found in Supplementary Figs. 102-106 and in Supplementary Table 20.

Synthesis of 10d (M_2M_1)

All operations were conducted under argon atmosphere. The synthesis of **10d** was performed under batch conditions similarly to **9c** with the furan deprotected and acetal protected monomer **1d** (210.6 mg, 0.41 mmol, 1.00 eq.) and monomer **2** (240.0 mg, 0.43 mmol, 1.05 eq.) dissolved in anhydrous DCM (170 mL). The purification was performed via reverse phase flash chromatography (SNAP C18 column 12 g, 12 mL min^{-1}) with MQ water and a THF gradient of 1.0 % per CV. Isocratic phases of a constant THF content have been applied at 20 % (5 CV), 25 % (10 CV), 30 % (18 CV), 35 % (6 CV) and 40 % (35 CV). The product was eluted as the THF content reached 40 % in the mobile phase. The fractions were gathered, the solvent was removed under reduced pressure and the residue was precipitated in Hex at ambient temperature. An acidic workup was not performed since the aldehyde functionality was regenerated after the purification. (Yield: 45 %, 52 mg). ^{1}H NMR (500 MHz, $CDCl_3$): δ 10.73 (s, 1H), 7.79 (d, J = 7.9 Hz, 2H), 7.73 (d, J = 7.9 Hz, 2H), 7.48 – 7.41 (m, 4H), 7.38 (t, J = 8.0 Hz, 1H), 7.24 (t, J = 7.9 Hz, 1H), 6.94 – 6.81 (m, 4H), 6.56 – 6.44 (m, 4H), 5.98 (d, J = 4.1 Hz, 1H), 5.24 – 5.17 (m, 4H), 5.13 (s, 2H), 4.72 – 4.64 (m, 1H), 3.74 (s, 3H), 3.68 – 3.57 (m, 2H), 3.49 – 3.34 (m, 4H), 3.31 – 3.10 (m, 3H), 3.04 – 2.83 (m, 3H), 2.59 (s, 3H), 2.23 – 2.05 (m, 1H), 1.74 – 1.50 (m, 6H), 1.50 – 1.22 (m, 6H). ^{13}C NMR (125 MHz, $CDCl_3$): δ 192.2, 180.3, 177.6, 175.8, 167.2, 139.7, 138.5, 136.5, 134.5, 129.7, 127.4, 127.2, 127.1, 126.5, 124.6, 121.6, 111.1, 110.4, 81.1, 69.9, 61.3, 52.8, 52.5, 47.6, 47.1, 46.7, 39.6, 39.3, 38.4, 38.1, 29.0, 28.5, 27.8, 27.4, 27.3, 25.7, 25.3, 23.0, 21.5. ESI-MS (*m/z*): $[M\text{-}Na]^{+}$ calcd. for $C_{57}H_{60}N_4NaO_{13}$, 1031.4049; found, 1031.4067. Details of the NMR chemical shift and mass spectrometric assignments can be found in Supplementary Figs. 107-113 and in Supplementary Table 21.

Synthesis of 10 (M_2)(M_1M_2)$_2$-X-(M_2M_1)$_2$(M_2)

The decamer **10** was synthesized in batch conditions from the hexamer **10c** (11.0 mg, 3.45 µmol, 1.00 eq.) and the dimer **10d** (7.3 mg, 7.23 mmol, 2.10 eq.) in dry DCM (25 mL). The purification was performed as for **7**. The reaction mixture was dried under reduced pressure. The residue was dissolved in THF (3 mL) and MeOH (3.5 mL) was slowly added until the solution turned turbid. The solution was let to decant and filtered (precipitation 1). After removal of the solvent under reduced pressure, the filtrate underwent another precipitation cycle after dissolution in THF (4 mL) by addition of MeOH (6.5 mL) (precipitation 2). The filtrate underwent a subsequent precipitation cycle after removal of the solvent and dissolution in THF (3 mL) by addition of Hex (1 mL) (precipitation 3). The target product was obtained as a solid. (Yield: 79.3 %, 14.1 mg). ESI-MS (*m/z*): $[M\text{-}2Na]^{2+}$ calcd. for $C_{288}H_{304}N_{22}Na_2O_{68}$, 2602.0395; found, 2602.0512. Details for mass-spectra can be found in Supplementary Figs. 114-115 and in Supplementary Table 22.

Synthesis of symmetric Copolymer 11 (($M_6M_5M_4M_2$)-X-($M_2M_4M_5M_6$)) and precursors 11a-b (11a: (M_4M_2)-X-(M_2M_4), 11b: (M_5M_6))

Synthesis of 11a (M_4M_2)-X-(M_2M_4)

11a was synthesized under similar conditions as **7c** with **10a** (383.5 mg, 0.2746 mmol, 1.00 eq.) and **4** (372.4 mg, 0.549 mmol, 2.10 eq.) in dry DCM (110 mL) and let to react under flow conditions. **11a** was precipitated in Et_2O and isolated via preparative chromatography by reverse phase separation. The

material was dissolved in MQ water:THF mixture to give a clear solution. The solution was injected on a C18 column (25 g). A gradient was performed with eluent A (MQ water:THF 75:25, 20 mL min^{-1}, 25 % B) and eluent B (THF) with stepwise 10 % B. **11a** was collected during the gradient increase 25 - 30 % B. (Yield: 3.7 %, 26 mg). Details of the NMR chemical shift and the mass spectrometric assignments can be found in Supplementary Figs. 116-121 and in Supplementary Table 23.

Synthesis of 11b (M_6M_5)

All operations were conducted under argon atmosphere. The synthesis of **11b** was performed under batch conditions similarly to **7d** with the furan deprotected and acetal protected monomer **5e** (54.2 mg, 80.6 µmol, 1.00 eq.) and monomer **6** (55.3 mg, 84.6 µmol, 1.05 eq.) dissolved in anhydrous DCM (40 mL). The purification was performed via reverse phase flash chromatography (SNAP C18 12 g, 12 mL min^{-1}) with MQ water and a THF gradient of 1.0 % per CV. Isocratic phases of a constant THF content have been applied at 20, 25, 30, 35 % (5, 10, 40 and 31 CV respectively) and 40 % (35 CV). The product was eluted as the THF content reached 45 % in the mobile phase. An acidic workup was not performed since the aldehyde functionality was regenerated after the purification. (Yield: 50 %, 51.6 mg). ^{19}F NMR (470 MHz, $CDCl_3$): δ -115.19, -115.20. ESI-MS (*m/z*): $[M\text{-}Na]^+$ calcd. for $C_{74}H_{79}N_6NaFO_{13}$, 1301.5581; found, 1301.5620. Details of the NMR chemical shift and the mass spectrometric assignments can be found in Supplementary Figs. 122-127 and in Supplementary Table 24.

Synthesis of 11 ($M_6M_5M_4M_2$)-X-($M_2M_4M_5M_6$)

The octamer **11** was synthesized under batch conditions from the tetramer **11a** (10.4 mg, 4.06 µmol, 1.00 eq.) and the dimer **11b** (10.9 mg, 8.55 mmol, 2.10 eq.) in dry DCM (20 mL). The reaction mixture was dried under reduced pressure and purified via flash chromatography in reverse mode with a SNAP Ultra C18 (12 g) column. The separation was conducted with MQ water and THF, with a THF gradient conducted 10 % stepwise. The expected product was eluted at 60 % THF. (Yield: 6.7 %, 3.4 mg). ESI-MS (*m/z*): $[M\text{-}K\text{-}TFA]^{2-}$ calcd. for $C_{282}H_{310}F_5N_{24}KO_{60}$, 2563.0754; found, 2063.0762. Details of the mass spectrometric characterization can be found in Supplementary Figs. 128-129 and in Supplementary Table 25.

Synthesis of Homopolymer 12 ($(M_3)_5$) and precursors 12a-b (12a: $(M_3)_2$, 12b: $(M_3)_3$)

Synthesis of 12a $(M_3)_2$

All operations were conducted under argon atmosphere. The synthesis of **12a** was performed under batch conditions similarly to **9c** with the furan deprotected and acetal protected monomer **3d** (52.4 mg, 95.5 µmol, 1.00 eq.) and monomer **3** (55.0 mg, 0.1 mmol, 1.05 eq.) dissolved in anhydrous DCM (40 mL). After irradiation, the single fractions were gathered and the solvent removed under reduced pressure. The residue was diluted with DCM (30 mL) and treated with aqueous 0.5 N HCl (30 mL) for 2 h. Subsequently, the layers were separated and the aqueous layer was extracted twice with DCM (20 mL). The purification was performed via reverse phase flash chromatography (SNAP C18 12 g column 12 mL min^{-1}) with MQ water and a THF gradient of 1.0 % per CV. Isocratic phases of a constant THF content have been applied at 20 % and 25 % (5 CV) and 30 % (20 CV). The product was eluted as the THF content reached 30 % in the mobile phase. The fractions were gathered, the solvent was removed under reduced pressure and the residue was precipitated in Hex at ambient temperature. (Yield: 45 %, 44.3 mg). ^{1}H NMR (500 MHz, $CDCl_3$): δ 10.76 (s, 1H), 7.82 (d, J = 8.2 Hz, 2H), 7.64 (d, J = 8.1 Hz, 2H), 7.43 (d, J = 7.9 Hz, 2H), 7.39 (t, J = 8.0 Hz, 1H), 7.36 – 7.31 (m, 2H), 7.28 (d, J = 8.8 Hz, 2H), 7.15 (t, J = 7.9 Hz, 1H), 6.91 – 6.84 (m, 3H), 6.71 (d, J = 8.3 Hz, 1H), 6.47 (s, 2H), 5.95 (d, J = 3.3 Hz, 1H), 5.20 (d, J = 4.0 Hz, 2H), 5.16 (s, 2H), 5.09 – 4.93 (m, 2H), 4.11 – 3.91 (m, 2H), 3.82 – 3.73 (m, 2H), 3.73 – 3.57 (m, 18H), 3.56 – 3.47 (m, 2H), 3.47 – 3.40 (m, 1H), 3.39 – 3.27 (m, 2H), 3.27 – 3.18 (m, 2H), 3.06 – 2.98 (m, 1H), 2.82 (s, 2H), 2.61 (s, 3H). ^{13}C NMR (125 MHz, $CDCl_3$): δ 192.3, 180.4, 177.9, 176.4, 167.6, 166.9, 162.1, 154.8, 142.3, 139.8, 139.4, 138.4, 136.6, 134.8, 134.6, 133.9, 129.8, 127.7,

127.6, 126.8, 125.4, 124.7, 123.7, 121.3, 110.5, 110.4, 81.0, 71.0, 70.3, 70.1, 70.00, 69.9, 69.5, 67.9, 67.3, 61.5, 48.0, 47.6, 39.9, 39.4, 38.3, 38.0, 37.6, 27.3, 21.2. ESI-MS (*m/z*): $[M\text{-}Na]^+$ calcd. for $C_{56}H_{60}NaN_4O_{15}$, 1051.3947; found, 1051.3957. Details of the NMR chemical shift assignments and the mass spectrometric assignments can be found in Supplementary Figs. 130-136 and Supplementary Table 26.

Synthesis of 12b $(M_3)_3$

All operations were conducted under argon atmosphere. The reaction was conducted under batch conditions similarly to the synthesis of dimer **12a**. The furan deprotected and acetal protected monomer **3d** (36.0 mg, 65.6 µmol, 1.50 eq.) and dimer **12a** (45.0 mg, 43.7 µmol, 1.00 eq.) were mixed and dissolved in anhydrous DCM (25 mL). The purification was carried out via reverse phase flash chromatography (SNAP C18 12 g column 12 mL min^{-1}) with water and a THF gradient of 1.0 % per CV. Isocratic phases of a constant THF content have been applied at 20 and 25 % (9 CV respectively), 30 % (15 CV), 35 % (24 CV) and 40 % (12 CV). The product was eluted as the THF content reached 40 % in the mobile phase. The fractions were gathered, the solvent was removed under reduced pressure and the residue was precipitated in Hex at ambient temperature. An acidic workup was not performed since the aldehyde functionality was regenerated after the purification. (Yield: 61 %, 28 mg). ^1H NMR (500 MHz, $CDCl_3$): δ 10.76 (s, 1H), 8.00 – 7.77 (m, 2H), 7.78 – 7.70 (m, 2H), 7.70 – 7.59 (m, 2H), 7.59 – 7.52 (m, 2H), 7.51 – 7.33 (m, 3H), 7.33 – 7.21 (m, 3H), 7.21 – 7.10 (m, 2H), 6.98 – 6.82 (m, 4H), 6.81 – 6.63 (m, 2H), 6.47 (s, 2H), 5.96 (s, 2H), 5.27 – 5.12 (m, 4H), 5.12 – 4.86 (m, 4H), 4.10 – 3.93 (m, 2H), 3.84 – 3.59 (m, 30H), 3.58 – 3.46 (m, 4H), 3.47 – 3.28 (m, 2H), 3.29 – 3.18 (m, 4H), 3.03 (s, 2H), 2.82 (s, 2H), 2.62 (s, 3H). ^{13}C NMR (125 MHz, $CDCl_3$): δ 192.3, 180.4, 176.4, 166.8, 162.2, 154.9, 142.4, 140.0, 139.4, 138.4, 136.7, 134.6, 129.9, 127.7, 127.6, 127.6 127.4, 126.9, 124.7, 123.7, 110.5, 110.5, 81.0, 70.4, 70.1, 69.9, 69.6, 69.5, 67.9, 67.3, 61.6, 61.4, 48.0, 47.9, 47.6, 40.0, 39.6, 38.3, 38.0, 37.6, 27.3, 27.0, 21.6. ESI-MS (*m/z*): $[M\text{-}Na]^+$ calcd. for $C_{82}H_{88}NaN_6O_{22}$, 1531.5844; found, 1531.5918. Details of the NMR chemical shift assignments and the mass spectrometric assignments can be found in Supplementary Figs. 137-143 and Supplementary Table 27.

Synthesis of the Dimer Acetal 12c

All operations were conducted under argon atmosphere and protected from light. The synthesis of **12c** was performed similarly to **1d**. Dimer **12a** (25.6 mg, 16.6 µmol, 1.00 eq.) was heated at 115 °C for 8 h under vacuum and was kept under vacuum at ambient temperature overnight for two cycles to quantitatively remove the furan protecting group. The product was employed without any further purification. Furan deprotected dimer **12a** (24.0 mg, 23.3 µmol, 1.00 eq.), TosOH (0.4 mg, 1.9 µmol, 0.08 eq.) and TMOF (10 µL, 93.3 µmol, 4.00 eq.) were suspended in anhydrous MeOH (1.0 mL). The reaction suspension was heated to 40 °C and the reaction mixture was stirred for 24 h. Afterwards, the crude reaction mixture was filtrated over silica (anhydrous DCM/MeOH 4:1, 1.0 % Et_3N) and the solvent was removed under reduced pressure to provide the target compound as an orange oil. **12c** was immediately employed for the synthesis of **12**.

Characterization of 12 $(M_3)_5$

The reaction was conducted under batch conditions similarly to the synthesis of dimer **12a**. Under inert atmosphere, furan deprotected and acetal protected dimer **12c** (24.0 mg, 23.3 µmol, 1.50 eq.) and trimer **12b** (23.5 mg, 15.5 µmol, 1.00 eq.) were mixed and dissolved in anhydrous DCM (10 mL). Purification was carried out via reverse phase flash chromatography (SNAP C18 12 g column 12 mL min^{-1}) with MQ water and a THF gradient of 1.0 % per CV. Isocratic phases of a constant THF content have been applied at 25 % (9 CV), 30 % (15 CV), 35 % (21 CV) and 40 % (18 CV). The product was eluted as the THF content reached 40 % in the mobile phase. The fractions were gathered, the solvent was removed under reduced pressure and the residue was precipitated in Hex at ambient temperature. An acidic workup was not performed since the aldehyde functionality was regenerated after the purification. (Yield: 20 %, 7 mg). ESI-MS ($C_{37}H_{36}FN_3NaO_7^+$) calculated: 676.243 found: 676.243

m/z. Details of the NMR chemical shift and mass spectrometric assignments can be found in Supplementary Figs. 144-147 and Supplementary Table 28.

Supplementary References

(1) Oehlenschlaeger, K., Mueller, J. O., Heine, N. B., Glassner, M., Guimard, N. K., Delaittre, G., Schmidt, F. G., Barner-Kowollik, C. Light-induced modular ligation of conventional RAFT polymers. *Angew. Chem. Int. Ed.* **52**, 762–766 (2013).

(2) Gramlich, W. M., Robertson, M. L. & Hillmyer, M. A. Reactive Compatibilization of poly(l-lactide) and conjugated soybean oil. *Macromolecules* **43,** 2313–2321 (2010).

(3) Zydziak, N.; Feist, F.; Huber, B.; Mueller, J. O.; Barner-Kowollik, C. Photo-induced sequence defined macromolecules via hetero bifunctional synthons. *Chem. Comm.* ***51***, 1799–1802 (2015).

(4) Richter, M., Chakrabarti, A., Ruttekolk, I. R., Wiesner, B., Beyermann, M., Brock, R., Rademann, J. Multivalent design of apoptosis-inducing Bid-BH3 peptide-oligosaccharides boosts the intracellular activity at identical overall peptide concentrations. *Chem. Eur. J.*, **18,** 16708–16715 (2012).

(5) Liu, F., Soh Yan Ni, A., Lim, Y., Mohanram, H., Bhattacharjya, S., Xing, B. Lipopolysaccharide neutralizing peptide-porphyrin conjugates for effective photoinactivation and intracellular imaging of gram-negative bacteria strains. *Bioconjugate Chem*., **23,** 1637–1647 (2012).

(6) Burkart, M. D., Foley, T. L., Garcia, C. J., Foley, T. L, Jadhav, A., Simeonov, A., Yasgar, A. Preparation of FRET reporters to support chemical probe development. *Org. Biomol. Chem.* **20,** 4601–4606 (2010).

(7) Robertson, M., Bremner, J. B., Coates, J., Deadman, J., Keller, P. A., Pyne, S. G., Somphol, K., Rhodes, D. I. Synthesis and antibacterial activity of C2-symmetric binaphtyl scaffolded amino acid derivatives. *Eur. J. Med. Chem.* **46,** 4201–4211 (2011).
